# Diversity and Eco-Evolutionary Associations of Endosymbiotic Astome Ciliates With Their Lumbricid Earthworm Hosts

**DOI:** 10.3389/fmicb.2021.689987

**Published:** 2021-06-18

**Authors:** Tomáš Obert, Ivan Rurik, Peter Vd’ačný

**Affiliations:** Department of Zoology, Faculty of Natural Sciences, Comenius University in Bratislava, Bratislava, Slovakia

**Keywords:** Astomatia, coevolution, host switch, host-driven diversification, Lumbricidae, phylogenetic interaction-adjusted index

## Abstract

Coevolution of endosymbionts with their hosts plays an important role in the processes of speciation and is among the most fascinating topics in evolutionary biology. Astome ciliates represent an interesting model for coevolutionary studies because they are so tightly associated with their host organisms that they completely lost the cell oral apparatus. In the present study, we used five nuclear markers (18S rRNA gene, ITS1–5.8S–ITS2 region, and 28S rRNA gene) and two mitochondrial genes (16S rRNA gene and cytochrome *c* oxidase subunit I) to explore the diversity of astomes inhabiting the digestive tract of lumbricid earthworms at temperate latitudes in Central Europe and to cast more light on their host specificity and coevolution events that shaped their diversification. The present coevolutionary and phylogenetic interaction-adjusted similarity analyses suggested that almost every host switch leads to speciation and firm association with the new host. Nevertheless, the suggested high structural host specificity of astomes needs to be tested with increased earthworm sampling, as only 52 out of 735 lumbricid earthworms (7.07%) were inhabited by ciliates. On the other hand, the diversification of astomes associated with megascolecid and glossoscolecid earthworms might have been driven by duplication events without host switching.

## Introduction

Astome ciliates are obligate endosymbionts of a wide range of invertebrates and some lower tetrapods such as newts and frogs (e.g., Heidenreich, 1935; de Puytorac, 1954, 1969, 1972; McAllister et al., 1993; McAllister and Trauth, 1996; Rataj and Vd’ačný, 2018). These eukaryotic microbes are so tightly associated with their host organisms that they completely lost the whole oral apparatus (i.e., the oral ciliary structures including a paroral membrane and adoral organelles, cytopharynx, and cytostome) during the course of their evolution (Jankowski, 2007; Lynn, 2008). Despite the morpho-evolutionary significance of astomes, they are nowadays among the most neglected ciliate groups. The comparatively low interest in astomes is due to their little economical and parasitological importance on the one hand and due to their relatively rare occurrence and low abundances in the temperate latitudes on the other hand (Obert and Vd’ačný, 2019, 2020a, 2021). Nevertheless, their taxonomy, molecular phylogeny, and ecology attained some attention in the past decade (Fokam et al., 2011, 2012; Sauvadet et al., 2017; Nana et al., 2018; Rataj and Vd’ačný, 2018, 2019; Obert and Vd’ačný, 2019, 2020a, 2021). Although these studies robustly determined the phylogenetic home of astomes within the highly diverse class Oligohymenophorea, the monophyletic origin of these mouthless ciliates remained open for further testing, as astomes isolated from planarians do not group with astomes isolated from annelids (Rataj and Vd’ačný, 2018; Antipa et al., 2020). Likewise, the coevolution of astomes with their hosts is an exciting but almost unexplored topic that needs to be addressed with molecular phylogenetic methods.

Coevolution of endosymbionts with their hosts plays an important role in the processes of speciation and represents one of the most fascinating topics in evolutionary biology (Poulin, 2007). Indeed, the host–endosymbiont relationship can be one of considerable dynamism, comprising a continuum of biological associations. Humphery-Smith (1989) demarcated the extremes in the continua of host specificity and host pathogenicity. He also recognized four groups of symbionts—poorly host specific and highly pathogenic, poorly host specific and non-pathogenic, highly host specific and highly pathogenic, and highly host specific and non-pathogenic. Astomes most likely belong to the last group, as indicated by no distinct pathological changes of their hosts and by molecular phylogenies in which astomes cluster according to the higher taxonomic groups of their host organisms (Fokam et al., 2011; Sauvadet et al., 2017; Rataj and Vd’ačný, 2018, 2019; Obert and Vd’ačný, 2019, 2020a). Nevertheless, the host specificity of astomes and their eco-evolutionary trends need to be assessed with a broad taxon sampling and extensive molecular data. Whether any of the principles concerning host–endosymbiont coevolution (for a review, see Brooks, 1979) holds also for astomes needs to be explored with modern phylogenetic methods as well. As suggested in our previous study (Obert and Vd’ačný, 2021), astome ciliates may at least partially conform to the Fahrenholz and Szidat coevolutionary rules. The former rule assumes that symbiont phylogeny mirrors host phylogeny, and hence, coevolution drives host–endosymbiont cospeciation (Stammer, 1957; Brooks and McLennan, 1993). On the other hand, the Szidat rule proposes that the more primitive hosts harbor the more primitive endosymbionts (Szidat, 1956, 1960).

In the present study, we focused on astome ciliates inhabiting the digestive tract of lumbricid earthworms sampled at temperate latitudes in Central Europe. The evolution of earthworms has been largely geographically structured, and climate changes along with the sea-level fluctuations have been invoked to explain the geographic distribution of lumbricids (Pérez-Losada et al., 2011; Fernández et al., 2012; Domínguez et al., 2015). Since astomes are intimately connected with their earthworm hosts and adapted to the particular physicochemical conditions of their digestive tract (Nana et al., 2012, 2014), it might be assumed that the evolution and distribution of astomes have been significantly shaped by the diversification of their earthworm hosts. James et al. (2021) suggested that the distribution of earthworms follows the Rapoport rule, which says that species latitudinal ranges are narrower in low latitudes than in high latitudes (Stevens, 1989). Interestingly, Benovics et al. (2018) proposed that the diversity of symbionts associated with hosts having small distribution areas is lower compared with the diversity of symbionts whose hosts exhibit a large distribution area. If these assumptions are correct and general enough, the geographical ranges of earthworms might have a strong impact on the diversification dynamics of their astome symbionts.

The main goal of the present paper is to cast more light on the structural and phylogenetic host specificity of astome ciliates and on the coevolution with their earthworm hosts using multigene data. We shall also test whether the Fahrenholz and Szidat rules hold for astomes and which coevolution events are among the most important drivers of the astome evolution.

## Materials and Methods

### Material Collection and Sample Processing

Lumbricid earthworms were collected at 25 localities in western Slovakia (Central Europe) especially in the capital city and its vicinity (Supplementary Table 1). Earthworms were determined based on the features of their external morphology (Pižl, 2002). The morphological identification was verified using NADH-ubiquinone oxidoreductase chain 1 (ND1) sequences, which were blasted against the National Center for Biotechnology Information (NCBI) database. The determination of earthworms from which astome ciliates had been isolated was further confirmed using the barcoding cytochrome *c* oxidase subunit I (COI). Primers and PCR conditions used for the amplification of ND1 and COI genes of earthworms are provided in Supplementary Tables 2, 3. The molecular assignment of examined earthworms to species is shown in Supplementary Figures 1, 2.

Earthworms were processed and dissected as described by Obert and Vd’ačný (2019, 2020a). Detected astome ciliates were manually isolated from the gut content of their earthworm hosts with Pasteur micropipettes adjusted as described by Foissner (2014). Living ciliates were investigated *in vivo* at low (50–400 ×) and high (1000 ×, oil immersion) magnifications with bright field and differential interference contrast under a Leica DM2500 optical microscope (Leica Microsystems GmbH, Wetzlar, Germany).

### Molecular Methods

Single cells of astome ciliates were placed in 180 μl of cell lysis buffer (Promega, Fitchburg, WI, United States), and their genomic DNA was isolated using the ReliaPrep^TM^ Blood gDNA Miniprep System (Promega, Fitchburg, WI, United States). Altogether, two mitochondrial (16S rRNA gene and COI gene) and three nuclear (18S, 5.8S, and 28S rRNA genes) genes as well as their internal transcribed spacers (ITS1 and ITS2) were PCR amplified. Primers and PCR conditions are provided in Supplementary Tables 2, 3. PCRs were carried out with the GoTaq^®^ Long PCR Master Mix (Promega, Fitchburg, WI, United States), following the protocol described in our previous studies (Obert and Vd’ačný, 2019, 2020a). Sequencing was conducted in Macrogen Europe B.V. (Amsterdam, Netherlands) on an ABI 3730 automatic sequencer. Newly acquired sequences were examined in Chromas ver. 2.6.6 (Technelysium Pty Ltd., South Brisbane, Australia), and high-quality sequence fragments were assembled into contigs in BioEdit ver. 7.2.5 (Hall, 1999).

### Phylogenetic Analyses

Four multigene datasets containing various combinations of nuclear and mitochondrial molecular markers were prepared. The first dataset contained five markers of the rDNA cistron (i.e., 18S rRNA gene, ITS1–5.8S–ITS2 region, and D1/D2-domains of the 28S rRNA gene). The second dataset contained mitochondrial 16S rRNA gene and nuclear rDNA cistron sequences. In the third dataset, rDNA cistron sequences were combined with the mitochondrial COI gene sequences. Finally, the rDNA cistron was concatenated with the two mitochondrial markers. Because nucleotide COI sequences were highly divergent among astome species, they were translated into amino acids using the invertebrate genetic code in MEGA X ver. 10.2 (Kumar et al., 2018) for the purposes of MrBayes and IQ-Tree analyses. Individual molecular markers were aligned on the MAFFT ver. 7 server^1^ (Katoh et al., 2019). The alignment strategy included the following: the progressive G-INS-1 method with an accurate guide tree, the unaligned level at 0.2 for mitochondrial sequences, the gap opening penalty at 1.53, the 200PAM/κ = 2 scoring matrix for nucleotide sequences, and the BLOSUM62 scoring matrix for amino acid sequences.

Phylogenetic trees were constructed with Bayesian inference, the maximum likelihood (ML) approach, and the distance neighbor-joining (NJ) algorithm. As concerns Bayesian analyses, they were conducted in Phycas ver. 2.2 (Lewis et al., 2015) as implemented in Python ver 2.7 and in MrBayes on XSEDE ver. 3.2.7 (Ronquist et al., 2012). Prior parameters of evolutionary models of individual molecular markers were estimated in jModelTest ver. 2.1.10 (Darriba et al., 2012) on the CIPRES portal ver. 3.3^2^ (Miller et al., 2010). Settings in both Bayesian analyses and convergence diagnostics followed our previous protocols (Obert and Vd’ačný, 2019, 2020a). ML phylogenies were estimated in IQTREE (Nguyen et al., 2015) on XSEDE ver. 1.6.10 on the CIPRES portal. The mixed data type option was selected for datasets containing nucleotide and amino acid sequences. Each molecular partition was assigned the best evolutionary substitution model, as chosen by the in-built program under the Bayesian information criterion. The ML search started from a BioNJ tree. The branching pattern of ML trees was assessed with 1,000 ultrafast bootstrap replicates, whereby the bnni algorithm was employed to reduce overestimating nodal support (Hoang et al., 2018). When spurious results were obtained with the three aforementioned algorithms, distance trees were built in MEGA X (Kumar et al., 2018) with the following settings: the maximum composite likelihood method, gamma-distributed rates among sites, a heterogeneous pattern among lineages, a pairwise deletion option to exclude alignment gaps, and 5,000 bootstrap replicates.

Species delimitation was carried out jointly with the estimation of a species tree using BP&P ver. 2.2 (Yang, 2015). Bayesian coalescent analyses were based on four molecular markers (18S rRNA gene, ITS region, D1/D2 domains of the 28S rRNA gene, and 16S rRNA gene) and had the following settings: (1) a gamma prior for population size at *G*(2, 1495); (2) a gamma prior for divergence time at the root at *G*(14, 470), all other divergence times were estimated using the Dirichlet prior with the equation 2 in Yang and Rannala (2010); (3) rates among individual markers, as estimated from the Dirichlet distribution; (4) heredity scalars of individual markers, as estimated from the data using a gamma prior *G*(4, 4); (5) rjMCMC simulations with burn-in of 100,000 samples and one million generations, whereby every second iteration was taken and 500,000 samples were saved; and (6) a large fine-tuning parameter (ε = 15) to guarantee a good mixing in the reversible jump algorithm. The maximum clade credibility tree was calculated in TreeAnnotator ver. 2.6.0 (Bouckaert et al., 2014).

The putative secondary structures of the astome ITS2 molecules were modeled in mfold ver. 3.0 (Zuker, 2003), following the pipeline described in our previous study (Obert and Vd’ačný, 2020a). The single difference was that the hybridized 5.8S–28S rRNA imperfect helix was much longer; that is, it contained 16 instead of five nucleotide pairs (Coleman, 2005; Keller et al., 2009). Secondary structures were graphically prepared in Varna ver. 3.93 (Darty et al., 2009). The 50%-majority rule consensus ITS2 models were proposed using the package 4SALE ver. 1.7.1 (Seibel et al., 2006). Nucleotide frequencies at individual positions in helices were computed and visualized with the online program WebLogo ver. 2.8.2 (Crooks et al., 2004). Compensatory base changes (CBCs) were detected within helices using the CBCAnalyzer option (Wolf et al., 2005), as implemented in 4SALE. The numbers of base pairs and unpaired bases were counted for each structural domain of the ITS2 molecules. The guanine–cytosine (GC) content was estimated on the webpage http://www.endmemo.com/bio/gc.php. The thermodynamic energy Δ*G* of the individual ITS2 molecules was calculated using the program RNAeval ver. 2.4.13 (Lorenz et al., 2016).

Pairwise *p*-distances were computed separately for the 18S rRNA gene, ITS region, 28S rRNA gene, 16S rRNA gene, and COI gene in MEGA X (Kumar et al., 2018). The pairwise *p*-distances served to construct ordination diagrams with the metric multidimensional scaling (MDS) method in Python ver. 3.6.6, using the scikit-learn package (Pedregosa et al., 2011), the iterative SMACOF algorithm, 20,000 iterations, 250 initializations, and ε at 10^–8^ to guarantee convergence.

### Cophylogenetic Analyses

An event-based tree reconciliation approach, as implemented in Jane ver. 4.01 (Conow et al., 2010), was used to analyze the evolutionary associations among astome ciliates and their earthworm hosts. Given the host and endosymbiont phylogenies, occurrences of five possible cophylogenetic events (cospeciation, duplication, duplication followed by host switching, loss, and failure to diverge) were reconstructed in a parsimony framework. Terminology of cophylogenetic events follows Conow et al. (2010). Host phylogeny was obtained by pruning the earthworm tree that was based on two nuclear genes, four mitochondrial genes, and seven mitochondrial tRNAs (Domínguez et al., 2015). Phylogenetic relationships among astome ciliates were based on our previous single- and multigene analyses (Obert and Vd’ačný, 2019, 2020a, 2021), considering also the results of the present coalescent-based phylogenetic analyses. To examine the effect of costs for each of the five cophylogenetic events, 10 different scenarios were tested following the strategy proposed by Benovics et al. (2019) and references cited therein. All cophylogenetic analyses were conducted with 10,000 generations and a population size of 1,000. To statistically test whether the global reconstruction cost was significantly lower than expected by chance, 500 samples were simulated using random endosymbiont trees.

A distance-based approach was used to analyze the cophylogeny of *Anoplophrya*, *Subanoplophrya*, *Maupasella*, and *Metaradiophrya* with their lumbricid hosts. African astomes could not be included in these global fit analyses, because no gene sequences are available from their megascolecid and glossoscolecid earthworm hosts in the NCBI database. Pairwise *p*-distances of the 18S rRNA gene of 12 astome species and pairwise *p-*distances of the ND1 gene of their eight lumbricid earthworm hosts were calculated in MEGA X. Global fit estimates and individual coevolutionary links were estimated with the parafit function (Legendre et al., 2002), as implemented in the ape package ver. 3.4 in R. Statistical significance of the results was assessed with 999 permutations, and the Lingoes correction was employed to avoid negative eigenvalues.

### Phylogenetic Interaction-Adjusted Similarity Analyses

The similarity of individual earthworm species was analyzed in light of phylogenetic relationships of their ciliate endosymbionts, using the unweighted phylogenetic interaction-adjusted (PINA) index proposed by Schmidt et al. (2017). However, the original unweighted PINA index violates the basic axiom for metrics—the identity of indiscernibles. In other words, the similarity of identical samples must equal 1. To meet the three basic axioms for metrics, we corrected the formula for calculation of unweighted PINA index, as follows:

$$P\,U=\frac{\sum_{i\in A}\sum_{j\in B}\Phi_{i\,j}}{\sqrt{\left(\sum_{i\in A}\sum_{j\in A}\Phi_{i\,j}\right)\,\left(\sum_{i\in B}\sum_{j\in B}\Phi_{i\,j}\right)}}$$

where *A* and *B* are individual samples (i.e., earthworm host species), *i* and *j* are individual taxa (i.e., ciliate endosymbiont species), and *Φ*_*ij*_ is the phylogenetic association matrix between taxa *i* and *j*. Two different strategies were used to calculate the phylogenetic association matrix Φ. The first approach included the estimation of pairwise *p*-distances of the 18S rRNA gene of 21 astome species (Supplementary Table 4). The rationale for this approach was based on computer simulations, which show that evolutionary processes leave almost the same information in pairwise distances between species as they leave in high-order combinations of character states (Felsenstein, 2004, p. 147). The second approach included the construction of a BioNJ tree from the 18S rRNA gene alignment in MEGA X. Following Schmidt et al. (2017), the BioNJ tree was interpreted as a phylogenetic association network. Consequently, cophenetic phylogenetic distances were extracted from the tree and transformed into an association matrix Φ. The unweighted PINA index was first derived from the pairwise *p*-distances and then from the cophenetic distances. Then, the similarity of earthworm species with respect to phylogenetic relationships of their astome endosymbionts was assessed by the metric MDS approach with the aforementioned settings.

## Results

### Alpha-Diversity of Astome Endosymbionts in Lumbricid Earthworms

The diversity of astomes in lumbricid earthworms was studied using five macronuclear markers (18S rRNA gene, ITS1–5.8S–ITS2 region, and the first two barcoding domains of the 28S rRNA gene) and two mitochondrial genes (16S rRNA gene and the gene encoding for cytochrome *c* oxidase, subunit I). In total, 95 new sequences were obtained during this study, including 16 new 18S rRNA gene sequences, 14 new ITS1–5.8S–ITS2 region sequences along with the D1/D2 domains of the 28S rRNA gene, 35 new 16S rRNA gene sequences, and 30 new sequences coding for COI. The corresponding GenBank accession numbers are summarized in Table 1.

**TABLE 1:** Characterization and origin of nuclear and mitochondrial sequences of astome ciliates analyzed in the present study.

Species	Specimen^a^	Host species	Locality^b^	18S rRNA gene	ITS region and 28S rRNA gene	16S rRNA gene	COI gene
*Anoplophrya allolobophorae*	JA-3 37 ACH^c^	*Allolobophora chlorotica*	JA-3	**MZ048824**	**MZ048775**	**MZ048789**	**MZ044303**
*Anoplophrya aporrectodeae*	PUz 17 AT	*Aporrectodea tuberculata*	PUz	**MZ048825**	**MZ048776**	**MZ048790**	–
	PUz 40 AT^d^	*A. tuberculata*	PUz	**MZ048826**	**MZ048777**	**MZ048791**	–
	PUz 41 AT	*A. tuberculata*	PUz	**MZ048827**	**MZ048778**	**MZ048792**	–
*Anoplophrya octolasionis*	MU 56 OL^e^	*Octolasion lacteovicinum*	MU	**MZ048828**	**MZ048779**	**MZ048793**	**MZ044304**
	MU 57 OL	*O. lacteovicinum*	MU	**MZ048829**	**MZ048780**	**MZ048794**	**MZ044305**
	MU 58 OL	*O. lacteovicinum*	MU	**MZ048830**	**MZ048781**	**MZ048795**	**MZ044306**
*Anoplophrya lumbrici*	KR 9 LT	*Lumricus terrestris*	KR	**MZ048831**	**MZ048782**	**MZ048796**	**MZ044307**
	KR 11 LT	*L. terrestris*	KR	MN121061	MN897871	**MZ048797**	**MZ044308**
	RZ 6 LT	*L. terrestris*	RZ	MN121062	MN897872	**MZ048798**	**MZ044309**
*Anoplophrya vulgaris*	JA-1 18 EF	*Eisenia andrei*^g^	JA-1	**MZ048832**	**MZ048783**	**MZ048799**	–
	JA-1 20 EF	*E. andrei*^g^	JA-1	MN121065	MN897875	**MZ048800**	–
	JA-1 21 EF	*E. andrei*^g^	JA-1	MN121066	MN897876	**MZ048801**	–
	NG 27 DV	*Dendrobaena veneta*	NG	**MZ048833**	MN897877	**MZ048802**	**MZ044310**
	NG 28 DV	*D. veneta*	NG	MN121067	MN897878	**MZ048803**	**MZ044311**
	BZ 13 EF	*E. andrei*^g^	BZ	**MZ048834**	MN897879	**MZ048804**	–
*Maupasella mucronata*	KDo 33 ET	*Eiseniella tetraedra*	KDo	MW182008	MW181992	–	**MZ044312**
	KDo 34 ET	*E. tetraedra*	KDo	MW182009	MW181993	–	**MZ044313**
	KDo 35 ET	*E. tetraedra*	KDo	MW182010	MW181994	–	**MZ044314**
	KDo 36 ET	*E. tetraedra*	KDo	MW182011	MW181995	–	**MZ044315**
*Metaradiophrya chlorotica*	JA-2 1M ACH	*A. chlorotica*	JA-2	**MZ048835**	**MZ048784**	**MZ048805**	**MZ044316**
	JA-2 2M ACH	*A. chlorotica*	JA-2	**MZ048836**	**MZ048785**	**MZ048806**	**MZ044317**
	JA-2 3M ACH	*A. chlorotica*	JA-2	**MZ048837**	**MZ048786**	**MZ048807**	**MZ044318**
*Metaradiophrya lumbrici*	JA-2 25 LT	*L. terrestris*	JA-2	MN121068	MN897880	**MZ048808**	**MZ044319**
	JA-2 26 LT	*L. terrestris*	JA-2	MN121069	MN897881	**MZ048809**	**MZ044320**
	KR 8 LT	*L. terrestris*	KR	MN121070	MN897882	**MZ048810**	**MZ044321**
	KR 10/1 LT	*L. terrestris*	KR	MN121071	MN897883	**MZ048811**	**MZ044322**
	RZ 4 LT	*L. terrestris*	RZ	MN121074	MN897884	**MZ048812**	**MZ044323**
	RZ 5 LT	*L. terrestris*	RZ	MN121075	MN897885	**MZ048813**	**MZ044324**
*Metaradiophrya varians*	BZ 12 EF	*E. andrei*^g^	BZ	MN121076	MN897886	**MZ048814**	**MZ044325**
	BZ 14 EF	*E. andrei*^g^	BZ	MN121077	MN897887	**MZ048815**	**MZ044326**
	JA-1 19 EF	*E. andrei*^g^	JA-1	MN121078	MN897888	**MZ048816**	**MZ044327**
	JA-1 22 EF	*E. andrei*^g^	JA-1	MN121079	MN897889	**MZ048817**	**MZ044328**
	BZkv 31 EF	*E. andrei*	BZkv	**MZ048838**	**MZ048787**	**MZ048818**	**MZ044329**
	BZkv 32 EF	*E. andrei*	BZkv	**MZ048839**	**MZ048788**	**MZ048819**	**MZ044330**
*Metaradiophrya speculorum*	HkD 59 AT	*A. tuberculata*	HkD	MW182012	MW181996	**MZ048820**	**MZ044331**
	HkD 60 AT	*A. tuberculata*	HkD	MW182013	MW181997	**MZ048821**	**MZ044332**
*Subanoplophrya nodulata*	PU 29 OT^f^	*Octolasion tyrtaeum*	PU	MN121063	MN897873	**MZ048822**	–
	PU 30 OT	*O. tyrtaeum*	PU	MN121064	MN897874	**MZ048823**	–

*^*a*^ Specimen code consists of a locality code as specified in Supplementary Table 1, an isolate code, and an abbreviation of host species name.*

*^*b*^ For locality codes and further details, see Supplementary Table 1.*

*^*c*^ Genomic DNA of the holotype specimen of *A. allolobophorae* has been deposited in the Natural History Museum in Bratislava, Slovakia (ID Collection Code 01427576).*

*^*d*^ Genomic DNA of the holotype specimen of *A. aporrectodeae* has been deposited in the Natural History Museum in Bratislava, Slovakia (ID Collection Code 01427577).*

*^*e*^ Genomic DNA of the holotype specimen of *A. octolasionis* has been deposited in the Natural History Museum in Bratislava, Slovakia (ID Collection Code 01427578).*

*^*f*^ Genomic DNA of the holotype specimen of *S. nodulata* has been deposited in the Natural History Museum in Bratislava, Slovakia (ID Collection Code 01427579).*

*^*g*^ Identified as *Eisenia fetida* by Obert and Vd’ačný (2019, 2020a) on the basis of morphological data. However, the identification within the *E. fetida* complex was specified to *E. andrei* based on the mitochondrial COI and ND1 sequences.*

*New sequences are in boldface.*

**FIGURE 1:**
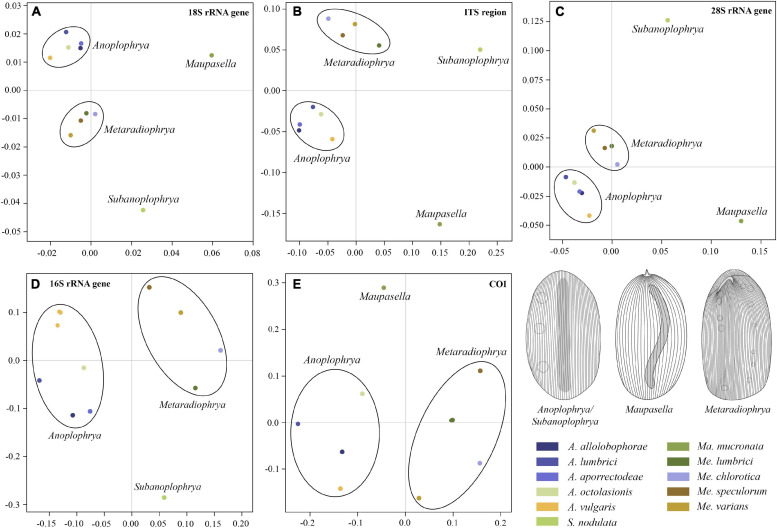
Multidimensional scaling based on pairwise *p-*distances of the 18S rRNA gene **(A)**, the ITS1–5.8S–ITS2 region **(B)**, the first two barcoding domains of the 28S rRNA gene **(C)**, the mitochondrial 16S rRNA gene **(D)**, and the mitochondrial gene encoding for cytochrome *c* oxidase, subunit I **(E)**. For pairwise *p-*distances, see Supplementary Tables 5–9.

According to the present phylogenetic and barcoding analyses, we distinguished 11 astome species belonging to four genera: *Anoplophrya* Stein, 1860; *Maupasella* Cépède, 1910; *Metaradiophrya* Heidenreich, 1935; and *Subanoplophrya* Obert and Vd’ačný, 2020a. Their intra- and interspecific genetic *p-*distances are summarized in Supplementary Tables 5–9. MDS analyses of pairwise genetic distances showed that each molecular marker enables unambiguous identification of one *Maupasella* and one *Subanoplophrya* species as well as of four *Metaradiophrya* and five *Anoplophrya* species. Apparently, the two mitochondrial genes provide the highest resolution among the astome species (Figures 1A–E). rDNA cistron sequences were identical within individual species (Supplementary Tables 5–7), while 16S rRNA gene sequences had 0.00%–6.60% intraspecific divergence and 10.62%–20.45% divergence between congeneric species pairs. However, when the two *Anoplophrya vulgaris* lineages, which were isolated from different hosts, are considered to be distinct taxa, the maximum intraspecific divergence drops only to 0.57% (Supplementary Table 8). COI sequences have 0.00%–1.11% intraspecific divergence and more than 18.47% divergence between congeneric species pairs (Supplementary Table 9). Unfortunately, all our attempts to obtain COI sequences from the *A. vulgaris* isolated from *Eisenia andrei* failed, and hence, we could not further test the species status of both lineages.

**FIGURE 2:**
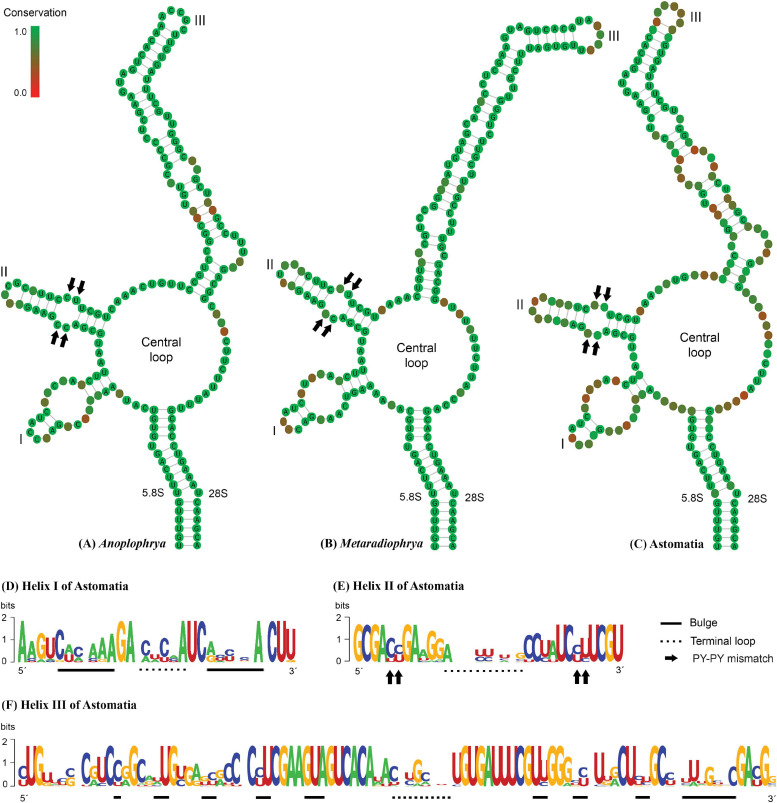
**(A–C)** Putative consensus secondary structure of the ITS2 molecules of five *Anoplophrya* species, four *Metaradiophrya* species, and 15 astome species. **(D–F)** The structure logos of three helices of 15 astome species. The height of a base is proportional to its frequency in the multiple sequence alignment. For further details, see Table 2 and Supplementary Figures 3–18.

The power of the ITS2 molecules for species discrimination was analyzed as well. The putative ITS2 secondary structures, including those isolated from megascolecid and glossoscolecid earthworms, are shown in Supplementary Figures 3–18. Their main properties are collated in Table 2. The consensus ITS2 model of astome ciliates and structure logo of individual helices are shown in Figures 2A–F. To summarize, each astome species has its own primary and secondary ITS2 structures, whereby the two *A. vulgaris* lineages shared the same ribotype. The distinctness of multiple species within the genera *Anoplophrya* and *Metaradiophrya* is further corroborated by compensatory and hemi-compensatory base changes (Tables 3, 4).

**TABLE 2:** Characterization of ITS2 molecules of astome ciliates.

Taxon	Total length	GC content (%)	Length of helix			Number of unpaired ribonucleotides in							No. of bulges in helix III	No. of GU pairings	Δ*G* (37°C, kcal/mol)
			I	II	III	Central loop	Terminal loop of helix I	Terminal loop of helix II	Terminal loop of helix III	Bulge of helix I	Bulge(s) of helix II	Bulges of helix III			
*Almophrya bivacuolata*	154	44.81	19	26	75	34	9	4	3	–	4	18	5	4	−43.2
*Anoplophrya allolobophorae*	160	57.50	25	27	84	24	6	5	5	5	4	21	6	4	−49.6
*Anoplophrya aporrectodeae*	160	56.25	25	27	84	24	4	3	5	9	4	21	6	5	−50.5
*Anoplophrya lumbrici*	163	47.85	21	26	80	36	13	4	5	–	4	19	5	3	−43.9
*Anoplophrya octolasionis*	161	49.07	25	26	80	30	4	4	5	9	4	19	5	3	−44.6
*Anoplophrya vulgaris*	156	54.49	24	24	78	30	4	4	5	8	4	19	5	5	−41.0
*Eudrilophrya complanata*	158	42.41	19	26	76	37	11	4	5	–	4	25	5	3	−29.5
*Metaracoelophrya* sp.	157	33.12	20	26	74	37	5	4	3	1	4	21	5	3	−35.3
*Metaradiophrya chlorotica*	164	49.39	25	28	85	26	4	4	7	7	4	25	7	7	−42.9
*Metaradiophrya lumbrici*	160	43.13	25	24	89	22	4	4	7	5	4	18	6	8	−41.6
*Metaradiophrya speculorum*	162	45.68	25	26	85	26	4	4	7	9	4	18	6	8	−40.4
*Metaradiophrya varians*	165	42.42	25	29	89	22	5	5	7	6	4	18	6	9	−43.3
*Maupasella mucronata*	162	43.83	27	27	92	16	4	5	7	9	4	25	6	6	−35.5
*Njinella prolifera*	160	48.75	23	26	83	28	13	4	3	–	6	26	6	3	−32.9
*Paraclausilocola constricta*	155	47.10	19	25	74	37	13	3	4	–	4	20	5	3	−34.5
*Subanoplophrya nodulata*	166	34.94	–	28	81	57	–	4	3	–	4	18	5	4	−42.7

Minimum	154	33.12	19	24	74	16	4	3	3	1	4	18	5	3	−29.5
Maximum	166	57.50	27	29	92	57	13	5	7	9	6	26	7	9	−50.5
Arithmetic mean	160.2	46.30	23.1	26.3	81.8	30.4	6.9	4.1	5.1	6.8	4.1	20.7	5.6	4.9	−40.7
Standard deviation	3.5	6.68	2.7	1.4	5.6	9.5	3.8	0.6	1.6	2.6	0.5	2.9	0.6	2.1	−5.8

*For further details, see Figure 2 and Supplementary Figures 3–18. GC, guanine–cytosine.*

**TABLE 3:** Numbers of CBCs (below diagonal) and hemi-CBCs (above diagonal) between ITS2 molecules of five *Anoplophrya* species isolated from lumbricid earthworms.

No.	Taxon	1.	2.	3.	4.	5.
**1.**	*Anoplophrya allolobophorae*	–	0	0	1	1
**2.**	*Anoplophrya aporrectodeae*	0	–	0	1	1
**3.**	*Anoplophrya lumbrici*	2	2	–	0	1
**4.**	*Anoplophrya octolasionis*	2	2	0	–	1
**5.**	*Anoplophrya vulgaris*	1	1	2	2	–

*CBCs, compensatory base changes.*

**TABLE 4:** Numbers of CBCs (below diagonal) and hemi-CBCs (above diagonal) between ITS2 molecules of four *Metaradiophrya* species isolated from lumbricid earthworms.

No.	Taxon	1.	2.	3.	4.
**1.**	*Metaradiophrya chlorotica*	–	2	3	3
**2.**	*Metaradiophrya lumbrici*	0	–	0	2
**3.**	*Metaradiophrya speculorum*	0	0	–	2
**4.**	*Metaradiophrya varians*	1	0	0	–

*CBCs, compensatory base changes.*

### Distribution and Prevalence of Astomes in Lumbricid Earthworms

Altogether 735 specimens belonging to 16 earthworm species from three ecological (anecic, epigeic, and endogeic) groups were collected at 25 localities in western Slovakia, Central Europe (Supplementary Table 1). In total, 11 astome species were detected in the earthworm digestive tract. The distribution of astome species is summarized in Supplementary Table 10. The anecic earthworm *Lumbricus terrestris* was the most abundant, with 221 specimens examined for the presence of intestinal astome ciliates. However, only 18 earthworms (8.14%), which had been sampled at three localities, contained two astome species, *Anoplophrya lumbrici* (Stein, 1854) Stein, 1860, and *Metaradiophrya lumbrici* (Dujardin, 1841) Heidenreich, 1935. The second most intensively studied earthworm species was the epigeic *E. andrei* from the *Eisenia fetida* complex altogether accounting for 170 exemplars. It was also inhabited by two astome species, *A. vulgaris* de Puytorac, 1954, and *Metaradiophrya varians* de Puytorac, 1954. As in *L. terrestris*, only about 8.82% of *E. andrei* specimens contained astome ciliates. The third most numerous species was the endogeic *Aporrectodea tuberculata*, with 120 collected individuals. However, only 4.16% of the examined earthworms were associated with astomes. Again, only two astome species were detected: *Anoplophrya aporrectodeae* sp. nov. was found in four earthworms, while *Metaradiophrya speculorum* Obert and Vd’ačný, 2021, was noticed in only one earthworm.

Other less frequent earthworm species inhabited by astomes were as follows: *Eiseniella tetraedra* (30 exemplars examined) with its endosymbiont *Maupasella mucronata* (Cépède, 1910) de Puytorac, 1954, which was detected in only one earthworm (prevalence 3.3%); *Allolobophora chlorotica* (30 exemplars) with *Metaradiophrya chlorotica* Williams, 1942, which was found in two earthworms all collected at the same locality (prevalence 6%), and *Anoplophrya allolobophorae* sp. nov., which was isolated only from a single earthworm (prevalence 3.3%); *Octolasion lacteovicinum* (20 exemplars) with *Anoplophrya octolasionis* sp. nov., which was noticed in three earthworms (prevalence 15%); *Octolasion tyrtaeum* (five exemplars) with *Subanoplophrya nodulata* sp. nov., which was found in two earthworms (prevalence 40%); and *Dendrobaena veneta* (25 exemplars) with *A. vulgaris*, which was found in six earthworms (prevalence 24%). There were as many as seven earthworm species in which no endosymbionts were detected: *Aporrectodea rosea* (five exemplars), *Aporrectodea trapezoides* (30 exemplars), *Dendrodrilus rubidus* (15 exemplars), *Dendrobaena octaedra* (three exemplars), *Fitzingeria platyura* (only one exemplar), *Lumbricus rubellus* (20 exemplars), *Octolasion lacteum* (10 exemplars), and *Octolasion* sp. (30 exemplars).

To summarize, the presence of astome ciliates was detected in eight out of the 16 earthworm species studied. However, only 8.37% of specimens of the positive earthworm species carried astome ciliates. When all 735 examined earthworms were considered, the prevalence of astomes slightly decreased to 7.07%.

### Phylogenetic Analyses

**FIGURE 3:**
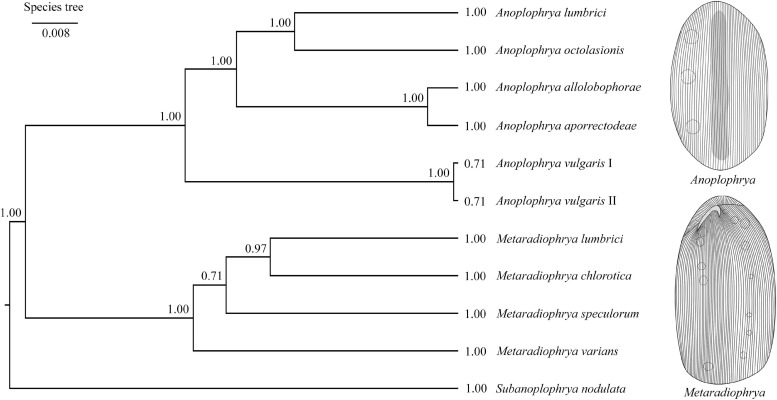
Coalescent species tree based on 16S, 18S, 28S rRNA, and ITS region sequences of astome ciliates isolated from lumbricid earthworms. Posterior probabilities of clades are provided along internal branches and posterior probabilities for the presence of individual species are provided behind the terminal branches. Scale bars denote the fraction of substitutions per site.

Bayesian inference, the ML approach, and the distance NJ algorithm were used to determine the phylogenetic positions of all newly acquired sequences within the order Astomatida based on two mitochondrial (16S rRNA gene and COI gene) and three nuclear (18S, 5.8S, and 28S rRNA genes) genes and their spacers (ITS1 and ITS2). The respective gene trees are shown in Supplementary Figures 19–21. The best resolution was obtained when all seven molecular markers were concatenated (Supplementary Figure 21). The genera *Metaradiophrya* and *Anoplophrya* were recognized as monophyletic groups with maximum support also in the multispecies coalescent tree (Figure 3). Species originated from epigeic earthworms, i.e., *M. varians* and *A. vulgaris*, branched off first within their genera. Relationships among *Anoplophrya* species were completely resolved: *A. lumbrici* was depicted in a sister position to *A. octolasionis*, and *A. allolobophorae* in a sister position to *A. aporrectodeae*. As concerns *Metaradiophrya*, *M. lumbrici* and *M. chlorotica* clustered together with strong support (0.97), and *M*. *speculorum* was shown as their nearest relative though with poor support (0.71). All 10 astome species received maximum statistical support in Bayesian species delimitation analyses. However, the taxonomic reliability of the two *A. vulgaris* lineages originating from different earthworms was only very poorly supported (Figure 3).

### Cophylogenetic Analyses

**FIGURE 4:**
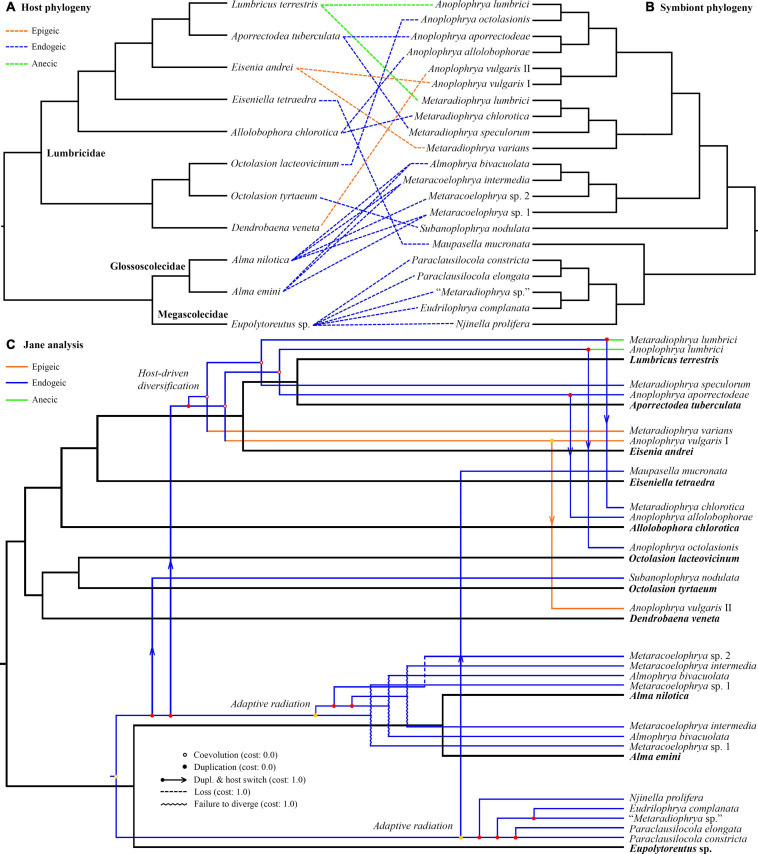
**(A,B)** Tanglegram showing associations between earthworm hosts and their astome symbionts. **(C)** One of the most parsimonious cophylogenetic scenarios between earthworm hosts and their astome symbionts constructed with Jane under default settings. Black branches represent the host phylogeny, and colored branches represent the symbiont phylogeny. Color denotes the ecological group of earthworms.

Jane reconstructions were conducted under 10 different cost scenarios. However, only five models yielded statistically significant results (*p* < 0.05): the Jane default, the TreeMap default, the TreeFitter default, the codivergence-adjusted TreeFitter, and the host switch-adjusted TreeFitter model (Table 5). The lowest total cost (11) was achieved with the latter scenario. All statistically significant models except for the codivergence-adjusted cost scheme suggested the same numbers of coevolutionary events: five cospeciations, eight duplications, seven duplications followed by host switching, one loss, and three times failure to diverge. The highest total cost (58) was obtained under the host switch prohibited model that unrealistically suggested as many as 35 loss events. Equalizing all event costs or extreme penalization of cospeciation, loss, and failure to diverge increased the number of duplications followed by host switching (11–13) and decreased the occurrence of cospeciation events (0–2). Jane reconstructions also indicated that the evolution of African astome ciliates, which are associated with the megascolecid and glossoscolecid earthworms, might have been primarily driven by duplication events. On the other hand, the diversification of European astome ciliates, which inhabit the digestive tube of lumbricid earthworms, might have been governed by duplication followed by host switching (Figure 4C). Interestingly, also coevolution events were detected at the base of the genera *Anoplophrya* and *Metaradiophrya*. The deepest-branching species *A. vulgaris* and *M. varians* are associated with the epigeic earthworms *E. andrei* or *D. veneta*, while *A. lumbrici* and *M. lumbrici* isolated from the anecic earthworm *L. terrestris* are placed within the endogeic clusters (Figures 4A–C). The distance-based analysis using parafit did not reject the null hypothesis of the global test that the evolution of astomes and their lumbricid hosts has been independent (*p* = 0.436). Nevertheless, such a result of the global test is to be expected, as within-group host switches are frequent in astomes associated with lumbricid hosts.

**TABLE 5:** Cophylogenetic analyses of earthworms and their ciliate endosymbionts using 10 different cost schemes.

Cost scheme	Event costs	Total cost	Cospeciation	Duplication	Dupl. and host switch	Loss	Failure to diverge
Jane default^†^	0 1 2 1 1	26	5	8	7	1	3
TreeMap default^†^	0 1 1 1 1	19	5	8	7	1	3
TreeFitter default^†^	0 0 2 1 1	18	5	8	7	1	3
Codivergence-adjusted TreeFitter model^†^	1 0 1 1 1	16	0–2–4	8	8–10–12	1	3
Host switch-adjusted TreeFitter model^†^	0 0 1 1 1	11	5	8	7	1	3
Cospeciation prohibited	10 1 1 1 1	24	0	7–8	12–13	1	3
Host switch prohibited	1 1 10 1 1	58	6	14	0	35	3
Sorting prohibited	1 1 1 10 1	33	0–1–2	7–8	11–12–13	1	3
FTD prohibitive	1 1 1 1 10	51	0–1–2	7–8	11–12–13	1	3
Equal weights	1 1 1 1 1	24	0–1–2	7–8	11–12–13	1	3

*Event costs are in the following order: cospeciation, duplication, duplication and host switch, loss, and failure to diverge. Total cost is the sum of occurrence of each evolutionary event multiplied by its cost. Statistically significant scenarios are marked by cross symbol (†).*

### Phylogenetic Interaction-Adjusted Similarity Analyses

**FIGURE 5:**
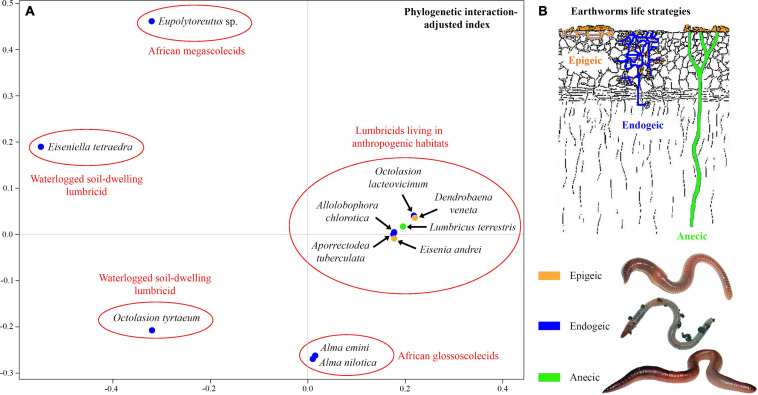
**(A)** Metric multidimensional scaling plot of 11 earthworm species based on phylogenetic relationships of their astome endosymbionts. The similarity was measured with the unweighted phylogenetic interaction-adjusted (PINA) index. **(B)** Earthworm life strategies. Colors denote the individual ecological group of earthworms.

The unweighted PINA index was calculated from *p*-distances of 18S rRNA gene sequences of 21 astome taxa and also from the cophenetic distances extracted from a respective BioNJ tree. As expected, both approaches brought very similar results, and therefore, only the MDS diagram inferred from the unweighted PINA index based on *p*-distances is presented here. MDS classified the 11 earthworm species into five well-separated groups (Figures 5A,B). Three groups contained only a single earthworm species each, documenting that their astome endosymbionts are phylogenetically highly distant and they do not co-occur. The African megascolecid earthworm *Eupolytoreutus* carries a comparatively diverse gut community of a deep-branching astome monophylum, which comprises *Eudrilophrya complanata*, “*Metaradiophrya* sp.,” *Njinella prolifera*, and *Paraclausilocola* spp. The lumbricid earthworms from waterlogged soils are also inhabited by deep-branching astomes, but they do not group together and represent orphan astome lineages in both single and multigene phylogenies. Specifically, the semi-aquatic earthworm *E. tetraedra* was associated exclusively with the astome *M. mucronata*, and the earthworm *O. tyrtaeum* only with the astome *S. nodulata*. The fourth group united African glossoscolecid earthworms belonging to the genus *Alma*. Glossoscolecids share a monophylum of phylogenetically closely related astomes belonging to the genera *Almophrya* and *Metaracoelophrya*. Finally, the fifth group contained a comparatively diverse assemblage of six lumbricid earthworm species. These earthworms have different lifestyles (epigeic, anecic, and endogeic) and are even not closely phylogenetically related, but they live in anthropogenic habitats and share astomes of the genera *Anoplophrya* and *Metaradiophrya*. Four of these six earthworm species lived in symbioses with one *Anoplophrya* and one *Metaradiophrya* species. Specifically, the epigeic earthworm *E. andrei* carried *A. vulgaris* and *M. varians*, the anecic earthworm *L. terrestris* carried *A. lumbrici* and *M. lumbrici*, the endogeic earthworm *A. tuberculata* carried *A. aporrectodeae* and *M. speculorum*, and the endogeic earthworm *A. chlorotica* carried *A. allolobophorae* and *M. chlorotica*. Two earthworm species were inhabited by only one astome species: the epigeic earthworm *D. veneta* by *A. vulgaris* and the endogeic earthworm *O. lacteovicinum* by *A. octolasionis* (Figures 4A,B). The present phylogenetic-adjusted similarity analyses thus suggested that each earthworm group is associated with either phylogenetically closely related astome endosymbionts (African megascolecids and glossoscolecids, European lumbricids living in anthropogenic habitats) or with orphan astome lineages (European lumbricid earthworms from waterlogged soils). African megascolecids and glossoscolecids have comparatively species-rich gut communities, whose diversification has been very likely shaped mostly by duplications followed by the coexistence of related astome species and genera (Figures 4A–C). On the other hand, the intestine communities of individual European lumbricid species typically include only a pair of astome species, whereby one species belongs to the genus *Anoplophrya* and the other one to *Metaradiophrya* (Figures 4A,B). The present data also suggest that host switching dominates in the evolution of these two astome genera, and almost every host switch is followed by speciation (Figure 4C). As a consequence, individual lumbricid species carry only a few endosymbiont species, but the whole lumbricid gut community in anthropogenic habitats is rather diverse and comparable with that of megascolecid and glossoscolecid earthworms.

## Discussion

### Molecular Taxonomy of Astome Ciliates Isolated From Lumbricids

The quality of species identification affects all subsequent evolutionary and ecological implications. There are several dozens of species in *Anoplophrya* and *Metaradiophrya* (de Puytorac, 1972) whose identification is very difficult because of comparatively few diagnostic morphological features and lack of information about their intraspecific variability (Obert and Vd’ačný, 2019). In the present study, we employed as many as five nuclear markers (18S rRNA gene, ITS1–5.8S–ITS2 region, and the first two barcoding domains of the 28S rRNA gene) and two mitochondrial genes (16S rRNA gene and the gene encoding for COI) along with the coalescent-based Bayesian statistical approach to delimiting species boundaries in astome ciliates. According to the present barcoding analyses, each molecular marker enables unambiguous identification of all astome species (Figures 1A–E). The single exception is *A. vulgaris*, which comprises two lineages each isolated from a different epigeic earthworm. Both lineages share identical rDNA cistron sequences but differ by as much as 6.48–6.60% in the mitochondrial 16S rRNA gene (Supplementary Table 8). However, when the two *A. vulgaris* lineages are considered to be separate taxa, the maximum intraspecific divergence drops only to 0.57%. Since the intraspecific distances range from 0.00 to 0.11% in nine other astome species, we suggest that divergences > 1% in the 16S rRNA gene are suggestive of different taxa. Bayesian delimitation analyses did not provide statistically significant support (posterior probability only 0.71) for this hypothesis (Figure 3), very likely due to the 100% identity in the much longer rDNA cistron. To cast more light on the species status of both lineages, COI sequences will be needed. The present distance analyses based on the remaining astomes revealed that COI sequences have 0.00–1.11% intraspecific divergence and more than 18.47% divergence between congeneric species pairs (Supplementary Table 9). This matches very well the results of barcoding analyses in the hymenostome genus *Tetrahymena* (Chantangsi et al., 2007; Doerder, 2014, 2019; Rataj and Vd’ačný, 2020) and various animal groups (e.g., Hebert et al., 2003a, b). Therefore, the 3% divergence threshold suggested for animals (Hebert et al., 2003a) and the 4% threshold suggested for *Tetrahymena* (Doerder, 2019) seem to be a reasonable barcoding gap in astomes as well. It is also important to mention that each astome species delimited by the Bayesian coalescent approach, exhibits unique primary and secondary structures of the ITS2 molecule (Table 2 and Supplementary Figures 3–18). The distinctness of each species within the genera *Anoplophrya* and *Metaradiophrya* is further strengthened by CBCs and hemi-CBCs in the ITS2 helices (Tables 3, 4). The occurrence of a single CBC within a helix can differentiate two species with a probability of 0.93, but the probability decreases to 0.76 when there is no CBC (Müller et al., 2007). Very likely the presence of two hemi-CBCs can unambiguously separate two species (Amato et al., 2007; Teng et al., 2015; Shazib et al., 2016).

### Comments on the Putative ITS2 Secondary Structure Model of Astomes

In our previous study, we proposed for the first time a secondary structure model for the astome ITS2 molecules (Obert and Vd’ačný, 2020a). Unfortunately, we used only five pairs to constrain the 5.8S–28S rRNA imperfect helix, which led to incorrect determination of the 3’-end of the ITS2 molecules. Here, we applied the strategy of Coleman (2005) and Keller et al. (2009); that is, we considered 16 pairs in the hybridized 5.8S–28S rRNA stem. This approach affected the putative secondary structure of the 5’-end of the ITS2 molecule in all astomes except for *Metaradiophrya*. The secondary structure of the 3’-end of the ITS2 molecules was also significantly modified causing the lack of helix IV in all astomes. Nevertheless, the structure of the second and third helices remained unchanged. To summarize, all astomes share a loop model radiating three helices except for *S. nodulata*, which exhibits only two helices (Supplementary Figures 3–18). To avoid imprecise determination of the ITS2 boundaries, we recommend modeling the whole 5.8S–28S rRNA stem. For an updated and corrected ITS2 model of astomes, the reader is referred to Figures 2A–C.

### Coevolution of Astomes With Their Earthworm Hosts

Despite the minimal number of coevolutionary studies about ciliates and their host organisms, associations between different taxonomic/ecological groups of hosts and genetically distinct lineages of symbiotic ciliates have been detected multiple times, for instance, between apostomes and shrimps (Lynn et al., 2014), astomes and earthworms (Obert and Vd’ačný, 2019, 2020a, 2021), trichostomes and mammals (Vallo et al., 2012; Moon-van der Staay et al., 2014; Vd’ačný, 2018), tetrahymenids and planarians (Rataj and Vd’ačný, 2020), and clevelandellids and cockroaches (Vd’ačný et al., 2019; Pecina and Vd’ačný, 2020a, b). However, our knowledge about coevolution events that are among the most important drivers of ciliate evolution is still rather insufficient. Moreover, it has not been hitherto tested whether symbiotic ciliates follow the Fahrenholz and Szidat coevolutionary rules. Fortunately, these issues can be statistically assessed using the information contained in phylogenetic trees and genetic distances between species pairs (Conow et al., 2010; Poulin et al., 2011). In the present study, we used a multifaceted approach to address these outstanding questions on a model of astome ciliates inhabiting the digestive tract of lumbricid earthworms at temperate latitudes in Central Europe. The present cophylogenetic and PINA similarity analyses suggested that (1) the evolution of astomes and their lumbricid hosts has been very likely independent, (2) diversification of astome ciliates associated with lumbricid earthworms might have been primarily driven by host switching and that of astomes associated with megascolecid and glossoscolecid earthworms by duplications, and (3) astomes very likely have a high structural and phylogenetic host specificity.

Thus, the present distance-based analyses suggested that the evolution of astomes and their lumbricid hosts has been independent. In other words, the genetic distances between species pairs of symbionts and hosts are not correlated. Astome ciliates thus very likely do not conform to the Fahrenholz rule, which assumes that symbiont phylogeny mirrors host phylogeny (Stammer, 1957; Brooks and McLennan, 1993). The lack of cospeciation signal is also in line with the present Jane analyses, which showed host switching as the main motor of the astome diversification in lumbricid earthworms (Figure 4C). Even when various cost scenarios were considered, the number of duplications followed by host switching always prevailed over the number of cospeciation events (Table 5). Bayesian coalescent-based delimitation analyses indicated with maximum statistical support that almost each host switch resulted in symbiont speciation (Figures 3, 4C). The single exception was *A. vulgaris*, which comprises two distinct mitochondrial lineages—one was isolated from *E. andrei* while the other one from *D. veneta*. This host switch occurred very likely only recently, as both lineages still share identical rDNA cistron sequences. However, they differ by as much as 6.48–6.60% in the mitochondrial 16S rRNA gene, indicating that they indeed represent two distinct taxa (Supplementary Table 8). It is important to mention that astomes isolated from African glossoscolecids and megascolecids exhibit a very different diversification mode in which duplications overdominate (Figure 4C). Such contrasting diversification patterns between European and African astomes might be ascribed to the different ranges of their hosts, which very likely follow the Rapoport rule (James et al., 2021). Earthworm ranges are narrower in low latitudes, which in turn decreases the chance for host switching after duplication. This speculation, however, needs to be thoroughly tested with a much broader sampling especially in tropical Africa and South America in the future.

### Host Specificity of Astomes

One may assume that astomes have a weak host specificity, as it was allegedly supposed for all symbiotic ciliates in the pre-molecular era or when only the highly conservative 18S rRNA gene was employed (Moon-van der Staay et al., 2014). However, the measurement of host specificity goes well beyond counting how many host species can successfully be used by a symbiont (Poulin et al., 2011). Nowadays, the structural, phylogenetic, and geographic host specificity is recognized, and sophisticated statistical tools are available for their evaluation. The comparison of African and European astomes revealed a very high structural and phylogenetic host specificity. Except for *A. vulgaris*, we have constantly detected each astome species only in one earthworm species (Obert and Vd’ačný, 2019, 2020a, 2021). Only a slightly broader host spectrum was reported for some African astomes, which were detected in multiple *Alma* species (Fokam et al., 2011, 2012; Nana et al., 2018). It is important to mention that many astome species were reported to occur in a variety of earthworm hosts in the past (for a review, see de Puytorac, 1972), a fact that might weaken the high structural specificity of ciliates. However, these literature data need to be taken with caution because they were often not accompanied by detailed morphological data, and most importantly, their reliability has not been hitherto tested using multigene data.

As concerns the phylogenetic host specificity, all molecularly studied astomes from megascolecid earthworms have been never isolated from glossoscolecid or lumbricid earthworms and vice versa. Even when literature data are considered, astomes isolated from earthworms have been never reported from any other invertebrate or vertebrate group (e.g., Cépède, 1910; Heidenreich, 1935; de Puytorac, 1954, 1972). Recent phylogenetic studies have also suggested that astome ciliates cluster according to associations with higher taxa of their hosts (Fokam et al., 2011; Sauvadet et al., 2017; Rataj and Vd’ačný, 2018, 2019; Obert and Vd’ačný, 2019, 2020a, 2021). These findings indicate that astomes may conform to the Szidat rule, which says that the more primitive hosts harbor the more primitive endosymbionts (Szidat, 1956, 1960). Interestingly, astomes isolated from planarians branched off first (Rataj and Vd’ačný, 2018, 2019), astomes isolated from polychaetes are in a sister position to astomes isolated from oligochaetes (Sauvadet et al., 2017), astomes isolated from megascolecid and glossoscolecid earthworms are placed relatively deep in the phylogenetic trees, and it seems that astomes isolated from lumbricid earthworms represent the crown radiation (Obert and Vd’ačný, 2019, 2020a, 2021). However, this “nice” picture was disrupted when *Maupasella* and *Subanoplophrya*, which had been isolated from waterlogged soil-dwelling lumbricids, were included in phylogenetic analyses. These two genera were classified as deep-branching orphan lineages nearby the clusters of astomes isolated from megascolecids and glossoscolecids. This, in turn, indicates that the validity of the Szidat rule needs to be explored with a much broader taxon sampling.

Finally, it is important to mention that the prevalence of astomes in lumbricid earthworms was very low; i.e., astomes were detected only in 52 out of the 735 earthworms studied (7.07%). Because of the rare occurrence of astomes, we cannot exclude that individual ciliate species might occur also in other earthworm species. Increased earthworm sampling is, therefore, needed to confirm or reject the hypothesis of the high structural host specificity of astomes.

### Chaos in Taxonomy and Nomenclature of Astomes Isolated From Lumbricid Earthworms

The type concept, according to which each nominal taxon actually or potentially has a name-bearing type (Article 61.1 of the International Commission on Zoological Nomenclature, 1999), provides an objective standard for the application of scientific names. However, typification problems concern the two most common astome genera isolated from lumbricid earthworms, *Anoplophrya* Stein, 1860, and *Metaradiophrya* Heidenreich, 1935. Stein (1860, p. 56) established *Anoplophrya* with three nominal species: “*Opalina lumbrici* St., *Opalina inermis* St., and *Naidum* Schmidt., *branchiarum* St.” He did not fix a type species. According to the Catalog of the generic names of ciliates, no type species has been fixed for *Anoplophrya* (Aescht, 2001). Therefore, Jankowski (2007, p. 901), referring to Aescht’s Catalog, fixed “*O. lumbrici* Schrank, 1803” as type species of *Anoplophrya*. Jankowski (2007), however, overlooked that already de Puytorac (1954, p. 202) mentioned “*A. lumbrici* (Schrank) (1803)” as type species, and hence no later designation is valid (Article 70.2 of the International Commission on Zoological Nomenclature, 1999). Nonetheless, both typifications are invalid because only nominal species originally included in a nominal genus are eligible for type fixation (Article 67.2 of the International Commission on Zoological Nomenclature, 1999). Neither “*O. lumbrici* Schrank, 1803” nor “*A. lumbrici* (Schrank) (1803)” [basionym *Leucophra lumbrici* Schrank, 1803] was originally included in *Anoplophrya*, and they are not conspecific with “*O. lumbrici* St.” (i.e., *O. lumbrici* Stein, 1854). Stein (1854, p. 183) first considered *L. lumbrici* Schrank, 1803, to be a *Bursaria* species, but later Stein (1867) recognized its assignment to the genus *Plagiotoma* Dujardin, 1841. According to molecular phylogenetic analyses, *Plagiotoma lumbrici* (Schrank, 1803) Dujardin, 1841, is not an astome but a hypotrich ciliate (Affa’a et al., 2004; Obert and Vd’ačný, 2020b). As concerns the three originally included species in *Anoplophrya*, only *O. lumbrici* Stein, 1854, remained classified in that genus after the revision of Heidenreich (1935). Thus, *O. inermis* Stein, 1859 (misclassified as “*Ophryoglena inermis* Stein, 1859” by Aescht, 2001, p. 17) became the type species of the astome genus *Acanthophrya* Heidenreich, 1935, by monotypy (Article 68.3 of the International Commission on Zoological Nomenclature, 1999). “*Naidum branchiarum*” was transferred to the genus *Collinia* Cépède, 1910, which nowadays belongs to a different subclass, Apostomatia (Lynn, 2008). Because the elimination of all but one of the originally included nominal species from a nominal genus does not in itself constitute type fixation (Article 69.4 of the International Commission on Zoological Nomenclature, 1999), we follow Recommendation 69A.3 of the International Commission on Zoological Nomenclature (1999) and fix *O. lumbrici* Stein, 1854, as the type species of *Anoplophrya*.

Heidenreich (1935) established *Metaradiophrya* with two species, *M. lumbrici* (Dujardin, 1841) and *M. falcifera* (Stein, 1861). Aescht (2001) assumed that Heidenreich (1935) did not fix a type species and proposed that *Metaradiophrya* Heidenreich, 1935, is a *nomen nudum* according to Article 13.3 of the International Commission on Zoological Nomenclature (1999). Therefore, Jankowski (2007, p. 897), referring to Aescht (2001), re-established *Metaradiophrya* and fixed *O. lumbrici* Dujardin, 1841, as its type species. However, H. Berger (pers. comm.) recognized that Heidenreich (1935) typified *Metaradiophrya* and designated *O. lumbrici* Dujardin, 1841, as its type species. This nomenclatural act was overlooked by Aescht (2001) and Jankowski (2007), as Heidenreich (1935) did not designate the type in the “Description” section on pages 335–338 but in the “Systematics” section on page 378. Thus, *Metaradiophrya* Jankowski, 2007, is a junior objective synonym and homonym of *Metaradiophrya* Heidenreich, 1935, because they share the same type species.

Finally, there are also some taxonomical and nomenclatural problems with “*Leucophrys nodulata* Dujardin, 1841,” the type species of *Subanoplophrya* Obert and Vd’ačný, 2020a, by original designation (H. Berger, pers. comm.). We ascribed the type species to Dujardin (1841, p. 460), who, however, considered his species to be conspecific with “*Leucophra nodulata*, Müller, Zool. dan. fasc. 2, tab. 80, fig. *a*⋅1. — Infus. p. 153.” *Leucophra* Müller, 1776 was placed on the Official Index of Rejected and Invalid Generic Names in Zoology (International Commission on Zoological Nomenclature, 1970), *Leucophra* Müller, 1780 is a *nomen dubium* (Aescht, 2001), and *Leucophrys* Ehrenberg, 1830 is considered a senior synonym of *Tetrahymena* Furgason, 1940, which is a *nomen conservandum* placed on the Official List of Generic Names in Zoology (International Commission on Zoological Nomenclature, 1970). *Tetrahymena* is now classified in the subclass Hymenostomatia (Lynn, 2008). Not only the generic assignment but also the conspecificity of Müller’s and Dujardin’s species are problematic since they originated from very different host groups. Specifically, Müller (1779, p. 124, 1786, p. 153) isolated *Leucophra nodulata* from *Nais littoralis* Müller, 1780 [ = *Paranais litoralis* (Müller, 1784)], which lives in mud and sand of the inter- and subtidal zone of the North Atlantic Ocean and belongs to the family Naididae. Although Dujardin (1841, p. 460) did not specify the host organism, he mentioned that it was different from that reported by Müller (1786). Moreover, Dujardin (1841) explicitly referred to lumbricids and not to naidids. We argue that Dujardin’s and Müller’s species cannot be conspecific because they were isolated from ecologically and phylogenetically highly distant oligochaete families. Because the type species of *Subanoplophrya* was misidentified, we apply here Article 70.3 of the International Commission on Zoological Nomenclature (1999). To promote stability and universality, we state the following: The type species of *Subanoplophrya* Obert and Vd’ačný, 2020a, is now fixed under Article 70.3 of the Code, as *S. nodulata* nov. spec., misidentified as *Leucophrys nodulata* Dujardin, 1841 (basionym *Leucophra nodulata* Müller, 1779) in the original designation by Obert and Vd’ačný (2020a). A new species needs to be established for the misidentified and dubious *Leucophrys nodulata*, which is done in the *Taxonomic Summary* section. Our proposal is also deemed to serve best the spirit of the Code, especially, of Articles 11.10, 67.13, and 69.2.4 of the International Commission on Zoological Nomenclature (1999).

## Taxonomic Summary

We use molecular data to diagnose the new species, following the barcoding strategy of Doerder (2019). When diagnostic molecular signatures were searched for, the query group contained all specimens of the species in question and the reference group included all remaining congeners. In the case of *S. nodulata*, the reference group contained all remaining astomes isolated from lumbricids. Since *S. nodulata* is genetically highly distant from all other astomes studied, we used only the rDNA cistron to diagnose it. There are more than 600 positions in the 16S rRNA gene alignment, which can be included in the diagnosis of *S. nodulata* and therefore are not listed below. Only nucleotide states that were shared by all members of the query group and were different from the states in the reference group were used as diagnostic characters. The reference alignments are provided in Supplementary Material. We interpret the isolated DNA as type material of new species, which is in accordance with Article 72.5.1 of the International Commission on Zoological Nomenclature (1999).

### Zoobank Registration Number of Work

urn:lsid:zoobank.org:pub:4A7904EB-282D-4CA1-8F12-029C6FC1B0C7

Phylum Ciliophora Doflein, 1901

Class Oligohymenophorea de Puytorac et al., 1974

Subclass Astomatia Schewiakoff, 1896

Order Astomatida Schewiakoff, 1896

Family Anoplophryidae Cépède, 1910

Genus *Anoplophrya* Stein, 1860 (type species: *O. lumbrici* Stein, 1854).

### *A. allolobophorae* Nov. Spec.

#### Zoobank Registration Number of New Species

urn:lsid:zoobank.org:act:48245C20-5CB6-4AF0-83B0-8DBF9B75367F

#### Diagnosis

ITS1–5.8S–ITS2 region: 252 G, 289 C. 16S rRNA gene: 14 G, 23 C, 30 A, 47 C, 60 G, 94 T, 98 T, 109 T, 126 C, 133 C, 168 G, 175 G, 177 G, 186 A, 187 A, 193 A, 199 A, 270 A, 293 T, 299 T, 302 G, 317 C, 324 G, 417 G, 446 G, 447 G, 451 G, 463 C, 581 C, 583 C, 588 A, 843 T, 871 G, 889 C, 894 G, 895 C, 896 T, 897 A, 902 A, 903 C, 914 G, 942 T, 943 G, 948 T. Cytochrome *c* oxidase, subunit I: 33 C, 39 A, 57 C, 72 C, 87 G, 99 G, 123 C, 125 C, 129 A, 130 A, 134 A, 140 A, 141 C, 159 G, 160 G, 164 C, 168 G, 171 G, 172 C, 174 A, 175 G, 176 C, 177 C, 180 C, 185 A, 188 A, 190 G, 195 G, 196 T, 199 T, 200 A, 206 T, 213 A, 216 T, 224 C, 238 G, 240 G, 260 T, 274 A, 282 C, 293 C, 357 A, 366 T, 369 G, 372 G, 381 C, 393 C, 417 T, 423 G, 471 G, 489 G, 495 C, 505 T, 507 A, 522 G, 531 C, 540 G, 567 C, 573 G, 597 C, 621 A, 630 G, 647 C, 648 A.

#### Type Locality

Soil from a garden, Jakubská ulica street, Rača, Bratislava, Slovakia (48°12′11.4″N, 17°09′05.3″E).

#### Type Host

*A. chlorotica* (Savigny, 1826).

#### Type Material

A DNA sample of holotype specimen has been deposited in the Natural History Museum, Vajanského nábrežie 2, 810 06 Bratislava, Slovakia (ID Collection Code 01427576).

#### Gene Sequences

The 18S rRNA gene, ITS1–5.8S–ITS2-28S rRNA gene, 16S rRNA gene, and COI sequences of the holotype specimen have been deposited in GenBank under accession nos. MZ048824, MZ048775, MZ048789, and MZ044303, respectively.

#### Etymology

The specific epithet is a singular genitive case of the Latin noun *allolobophor*⋅*a*, ⋅*ae* [f], meaning an *Anoplophrya* from *Allolobophora*. The species-group name is to be treated as an adjective used as a substantive in the genitive case, because of its derivation from the host’s generic name (Article 11.9.1.4. of the International Commission on Zoological Nomenclature, 1999).

### *A. aporrectodeae* Nov. Spec.

#### Zoobank Registration Number of New Species

urn:lsid:zoobank.org:act:E2F81BAB-E67B-42D6-8466-CD0F0C34EBA2

#### Diagnosis

18S rRNA gene: 409 G, 718 A. ITS1–5.8S–ITS2 region: 289 T. 16S rRNA gene: 40 C, 51 T, 52 G, 92 C, 109 C, 116 G, 126 G, 164 G, 186 G, 187 C, 193 G, 270 G, 273 C, 274 G, 290 C, 293 C, 400 C, 413 G, 436 C, 443 A, 444 G, 446 A, 451 A, 545 A, 550 G, 570 C, 846 G, 894 A, 900 G, 902 C, 903 G, 905 A, 940 G, 943 T, 944 T.

#### Type Locality

Agricultural soil from a garden, Spodná ulica street, Pusté Úl’any village, Galanta district, Slovakia (48°13′41.0″N, 17°34′48.6″E).

#### Type Host

*A. tuberculata* (Eisen, 1874).

#### Type Material

A DNA sample of holotype specimen has been deposited in the Natural History Museum, Vajanského nábrežie 2, 810 06 Bratislava, Slovakia (ID Collection Code 01427577).

#### Gene Sequences

The 18S rRNA gene, ITS1–5.8S–ITS2-28S rRNA gene, and 16S rRNA gene sequences of the holotype specimen have been deposited in GenBank under accession nos. MZ048826, MZ048777, and MZ048791, respectively.

#### Etymology

The specific epithet is a singular genitive case of the Latin noun *aporrectode*⋅*a*, ⋅*ae* [f], meaning an *Anoplophrya* from *Aporrectodea*. The species-group name is to be treated as an adjective used as a substantive in the genitive case, because of its derivation from the host’s generic name (Article 11.9.1.4. of the International Commission on Zoological Nomenclature, 1999).

### *A. octolasionis* Nov. Spec.

#### Zoobank Registration Number of New Species

urn:lsid:zoobank.org:act:8D517D9C-D2B1-4D08-8809-A25DFBAA77D3

#### Diagnosis

18S rRNA gene: 271 A, 276 T, 729 T. ITS1–5.8S–ITS2 region: 77 C, 78 A, 80 A, 269 A. 28S rRNA gene: 214 G, 493 A, 501 T, 553 A, 693 T, 771 C. 16S rRNA gene: 12 C, 30 C, 49 C, 53 A, 54 A, 91 G, 94 G, 137 G, 153 T, 166 T, 190 C, 202 T, 261 A, 273 A, 286 T, 415 G, 418 G, 434 G, 437 G, 448 T, 502 G, 530 G, 541 G, 547 G, 554 G, 558 -, 560 G, 567 T, 576 G, 613 A, 709 G, 764 C, 778 G, 779 T, 790 A, 801 C, 840 T, 844 A, 846 T, 869 G, 876 C, 890 T, 901 A, 909 A, 917 G, 918 T, 920 C, 928 C, 934 C, 937 T, 958 A, 960 G. Cytochrome *c* oxidase, subunit I: 18 T, 27 C, 28 A, 75 T, 87 T, 96 C, 124 G, 136 G, 141 T, 147 T, 154 G, 162 T, 168 A, 173 C, 177 G, 181 A, 182 G, 185 T, 186 A, 188 C, 190 A, 192 T, 195 T, 198 A, 200 C, 202 T, 203 C, 204 C, 206 C, 209 T, 217 G, 219 T, 224 A, 229 T, 245 T, 258 A, 259 A, 275 A, 283 C, 291 C, 293 G, 301 A, 303 A, 304 T, 305 C, 342 T, 360 C, 396 C, 399 G, 414 G, 417 C, 441 G, 456 C, 465 T, 468 T, 492 A, 496 A, 498 A, 499 C, 501 C, 504 G, 507 G, 510 C, 516 G, 519 T, 520 C, 522 C, 534 G, 538 C, 540 T, 543 A, 550 A, 555 G, 568 C, 577 A, 591 T, 597 G, 604 C, 606 T, 612 A, 615 T, 633 C, 639 G, 642 C, 646 T, 669 T, 672 C, 687 A, 700 C.

#### Type Locality

Soil from a garden, Moskovská ulica street, Staré mesto, Bratislava, Slovakia (48°09′05.0″N, 17°07′18.2″E).

#### Type Host

*O. lacteovicinum* Zicsi, 1968.

#### Type Material

A DNA sample of holotype specimen has been deposited in the Natural History Museum, Vajanského nábrežie 2, 810 06 Bratislava, Slovakia (ID Collection Code 01427578).

#### Gene Sequences

The 18S rRNA gene, ITS1–5.8S–ITS2-28S rRNA gene, 16S rRNA gene, and COI sequences of the holotype specimen have been deposited in GenBank under accession nos. MZ048828, MZ048779, MZ048793, and MZ044304, respectively.

#### Etymology

The specific epithet is a singular genitive case of the Latin noun *octolasion*, ⋅*is* [n], meaning an *Anoplophrya* from *Octolasion*. The species-group name is to be treated as an adjective used as a substantive in the genitive case, because of its derivation from the host’s generic name (Article 11.9.1.4. of the International Commission on Zoological Nomenclature, 1999).

*Incertae sedis* in order Astomatida Schewiakoff, 1896

Genus *Subnoplophrya* Obert and Vd’ačný, 2020a (type species: *S. nodulata* nov. spec.)

### *S. nodulata* Nov. Spec.

#### Zoobank Registration Number of New Species

urn:lsid:zoobank.org:act:F1976768-FE49-4E12-8B26-33FA14D6BDE0

#### Diagnosis

18S rRNA gene: 176 C, 214 T, 237 T, 271 G, 273 A, 276 C, 476 T, 631 C, 642 C, 643 G, 648 C, 649 T, 653 A, 662 A, 667 C, 700 T, 706 G, 709 A, 710 C, 715 G, 723 C, 730 C, 745 C, 766 A, 768 C, 799 C, 818 A, 1043 T, 1044 A, 1177 A, 1339 A, 1345 T, 1346 C, 1372 G, 1467 T, 1471 A, 1475 A, 1476 G, 1478 T, 1485 A, 1624 A, 1666 A, 1671 A, 1680 G, 1685 C, 1686 T. ITS1–5.8S–ITS2 region: 7 A, 17 T, 20 T, 21 G, 24 A, 25 T, 26 T, 27 T, 29 T, 30 G, 31 T, 32 T, 33 G, 34 C, 35 T, 36 G, 38 A, 44 C, 45 A, 53 G, 59 A, 62 T, 64 A, 71 T, 74 A, 246 T, 247 A, 248 A, 250 A, 254 A, 255 C, 256 T, 265 A, 267 T, 272 A, 280 T, 286 A, 288 G, 290 G, 300 C, 313 A, 315 T, 317 A, 320 T, 329 G, 330 A, 332 T, 336 T, 354 A, 357 A, 381 A, 384 T, 388 A, 389 A, 392 T, 394 A, 411 C. 28S rRNA gene: 99 A, 102 A, 110 T, 133 A, 135 G, 136 A, 147 T, 149 T, 153 C, 154 T, 156 T, 165 T, 166 C, 174 A, 178 A, 195 T, 206 A, 208 A, 209 G, 228 A, 229 T, 234 A, 364 C, 399 G, 408 A, 417 C, 420 T, 424 A, 426 G, 428 A, 438 T, 439 T, 440 A, 450 G, 451 T, 452 A, 456 A, 461 C, 463 T, 465 A, 466 A, 471 T, 472 T, 474 T, 483 A, 487 A, 489 T, 490 A, 503 C, 504 C, 508 T, 532 C, 534 A, 535 A, 537 A, 555 A, 561 T, 562 T, 563 T, 564 T, 566 C, 590 C, 592 A, 593 T, 595 T, 596 T, 598 A, 630 G, 682 A, 710 A, 724 G, 767 G, 775 C, 776 A, 789 A, 790 A, 848 A, 849 A, 878 T, 894 A.

#### Type Locality

Upper 50-cm turf layer in the riparian zone of the Rašelinisko pond in the vicinity of the Pusté Úl’any village, Galanta district, Slovakia (48°13′21.9″N, 17°34′49.9″E).

#### Type Host

*O. tyrtaeum* (Savigny, 1826).

#### Type Material

A DNA sample of holotype specimen has been deposited in the Natural History Museum, Vajanského nábrežie 2, 810 06 Bratislava, Slovakia (ID Collection Code 01427579).

#### Gene Sequences

The 18S rRNA gene, ITS1–5.8S–ITS2-28S rRNA gene, and 16S rRNA gene sequences of the holotype specimen have been deposited in GenBank under accession nos. MN121063, MN897873, and MZ048822, respectively.

#### Etymology

The Latin adjective *nodulat*⋅*us*, -*a*, -*um* [m, f, n] (nodulated) refers to contractile vacuoles that appear as scattered noduli.

## Data Availability Statement

The data presented in the study are deposited in the GenBank database (https://www.ncbi.nlm.nih.gov/nucleotide/), accession numbers MZ048824-MZ048839, MZ048775-MZ048788, MZ048789-MZ048823, MZ044303-MZ04332, MZ044869-MZ044895, MZ056758-MZ056794. Results of all analyses are included in this published article and Supplementary Material. GenBank accession numbers of sequences used in phylogenetic analyses can be found in the Supplementary Material.

## Author Contributions

PV conceptualized the research. TO performed the laboratory work. IR prepared all custom Python scripts. PV and TO created visualizations and wrote the original draft of the manuscript. All the authors analyzed data, reviewed, edited, and approved the final version of the manuscript.

## Conflict of Interest

The authors declare that the research was conducted in the absence of any commercial or financial relationships that could be construed as a potential conflict of interest.

## Footnotes

1 http://mafft.cbrc.jp/alignment/server/

2 http://www.phylo.org/
