# Supplementary material for: Diversity and Eco-Evolutionary Associations of Endosymbiotic Astome Ciliates With Their Lumbricid Earthworm Hosts
Source: Front Microbiol. 2021 Jun 18;12:689987. doi: 10.3389/fmicb.2021.689987 (PMC8250849; doi:10.3389/fmicb.2021.689987)
Supplement: Supplementary file 1 [file Table_1.pdf]

# SUPPLEMENTARY MATERIAL

# Diversity and eco-evolutionary associations of endosymbiotic astome ciliates with their lumbricid earthworm hosts

***Tomáš Obert, Ivan Rurik, Peter Vďačný****

*Department of Zoology, Faculty of Natural Sciences, Comenius University in Bratislava, Bratislava, Slovakia*

***Correspondence:***
*Peter Vďačný*
*peter.vdacny@uniba.sk*

| | |
|---|---|
| **Pages:** | **70** |
| **Tables:** | **12** |
| **Figures:** | **21** |
| **Alignments:** | **5** |
| **References:** | **12** |

**SUPPLEMENTARY TABLE 1 |** Characterization of collection sites of earthworm species examined for the presence of astome ciliates.

| Locality no. | Locality code | Collection date[a] | Collection site | GPS coordinates | Host species | No. of specimens |
|---|---|---|---|---|---|---|
| **Decomposing plant material** | | | | | | |
| 1 | BZ | 05/19/2018 | Decomposing plant material from a compost heap, south part of the Botanical Garden, Karlova Ves, Bratislava | 48°08′43.5″N 17°04′21.1″E | *Eisenia andrei*[b] | 60 |
| 2 | BZkv | 09/12/2019 | Decomposing plant material from a compost heap, west part of the Botanical Garden, Karlova Ves, Bratislava | 48°08′51.0″N 17°04′26.5″E | *Eisenia andrei* | 50 |
| 3 | JA-1 | 06/28/2018 | Decomposing plant material and humous soil from a garden compost heap, Jakubská ulica street, Rača, Bratislava | 48°12′10.9″N 17°09′05.7″E | *Eisenia andrei* | 60 |
| 4 | NG | 06/30/2018 | Humous soil with high content of decomposing plant material from a garden at the foothill of the Malé Karpaty Mts., Na Grunte street, Nové mesto, Bratislava | 48°11′44.4″N 17°07′47.5″E | *Dendrobaena veneta* | 25 |
| 5 | PUh | 12/04/2019 | Decomposing material under fallen trees, poplar forest in the vicinity of the Pusté Úľany village, Galanta district | 48°13′08.2″N 17°34′43.0″E | *Lumbricus rubellus* | 20 |
| **Floodplain and waterlogged soils** | | | | | | |
| 6 | KR | 10/03/2017 | Floodplain soil from a riparian, willow-poplar forest near the Karlova Ves branch of the Danube river, Bratislava | 48°08′47.5″N 17°04′08.0″E | *Lumbricus terrestris* | 50 |
| 7 | AMc | 10/14/2019 | Floodplain soil from a riparian, willow-poplar forest near the Danube river, Devín, Bratislava | 48°10′46.0″N 16°58′39.6″E | *Aporrectodea tuberculata* | 25 |
| 8 | HkD | 10/14/2019 | Moist soil near the confluence of the Danube and Morava rivers, foot of the cliff of the Devín Castle, Bratislava | 48°10′28.0″N 16°58′37.0″E | *Aporrectodea rosea*<br>*Aporrectodea tuberculata* | 5<br>15 |
| 9 | LS | 10/14/2019 | Floodplain soil from a meadow near the Danube river, Devín, Bratislava | 48°10′15.3″N 16°59′04.3″E | *Aporrectodea tuberculata*<br>*Octolasion lacteum* | 20<br>10 |
| 10 | KDo | 09/15/2019 | Waterlogged soil around the Banský potok stream in an urban oak-hornbeam forest, Knižková dolina valley, Bratislava, Malé Karpaty Mts. | 48°13′37.0″N 17°07′13.1″E | *Dendrobaena octaedra*<br>*Eiseniella tetraedra* | 3<br>10 |
| 11 | KDo | 09/29/2019 | Moist soil in the vicinity of the Banský potok stream in an urban oak-hornbeam forest, Knižková dolina valley, Bratislava, Malé Karpaty Mts. | 48°12′60.0″N 17°08′21.7″E | *Lumbricus terrestris* | 1 |
| 12 | CBk | 10/06/2019 | Waterlogged soil around the Šenkársky potok stream in an urban oak-hornbeam forest, Bratislava, Malé Karpaty Mts. | 48°13′50.2″N 17°08′21.9″E | *Dendrodrilus rubidus*<br>*Eiseniella tetraedra*<br>*Octolasion* sp. | 15<br>20<br>30 |
| 13 | MB | 10/27/2019 | Moist soil from an urban oak-hornbeam forest in the vicinity of Malá Baňa, Bratislava, Malé Karpaty Mts. | 48°13′14.7″N 17°07′45.6″E | *Fitzingeria platyura* | 1 |

| Locality no. | Locality code | Collection date[a] | Collection site | GPS coordinates | Host species | No. of specimens |
|---|---|---|---|---|---|---|
| 14 | PU | 07/02/2018 | Upper 50 cm turf layer in the riparian zone of the Rašelinisko pond in the vicinity of the Pusté Úľany village, Galanta district | 48°13′21.9″N 17°34′49.9″E | *Octolasion tyrtaeum* | 5 |
| **Agricultural and grassland soils** | | | | | | |
| 15 | RZ | 06/06/2017 | Agricultural soil from a garden, Šúrska ulica street, Rendez, Bratislava | 48°11′57.6″N 17°10′25.0″E | *Lumbricus terrestris* | 50 |
| 16 | JA-2 | 11/28/2018 | Agricultural soil from a garden, Jakubská ulica street, Rača, Bratislava | 48°12′12.2″N 17°09′03.1″E | *Allolobophora chlorotica* | 10 |
| 17 | FNS | 12/09/2019 | Soil from a grassland in the vicinity of the Faculty of Natural Sciences, Comenius University, Karlova Ves, Bratislava | 48°08′56.7″N 17°04′21.2″E | *Lumbricus terrestris* | 25 |
| 18 | PUz | 06/20/2018 | Agricultural soil from a garden, Spodná ulica street, Pusté Úľany village, Galanta district | 48°13′41.0″N 17°34′48.6″E | *Aporrectodea tuberculata* | 20 |
| 19 | PUz | 09/14/2019 | Agricultural soil from a garden, Spodná ulica street, Pusté Úľany village, Galanta district | 48°13′41.0″N 17°34′48.6″E | *Allolobophora chlorotica* | 10 |
| | | | | | *Aporrectodea trapezoides* | 10 |
| | | | | | *Aporrectodea tuberculata* | 20 |
| | | | | | *Lumbricus terrestris* | 20 |
| 20 | PUp | 11/28/2019 | Agricultural soil from a field in the vicinity of the Pusté Úľany village, Galanta district | 48°13′19.2″N 17°34′57.1″E | *Aporrectodea tuberculata* | 20 |
| **Garden soils** | | | | | | |
| 21 | JA-2 | 06/28/2018 | Loamy soil with fallen needles in the surroundings of a garden wall, Jakubská ulica street, Rača, Bratislava | 48°12′12.2″N 17°09′03.1″E | *Lumbricus terrestris* | 50 |
| 22 | JA-3 | 09/18/2019 | Soil from a garden, Jakubská ulica street, Rača, Bratislava | 48°12′11.4″N 17°09′05.3″E | *Allolobophora chlorotica* | 10 |
| | | | | | *Aporrectodea trapezoides* | 20 |
| 23 | HO | 11/08/2019 | Soil from a garden, Horská ulica street, Nové mesto, Bratislava | 48°11′52.1″N 17°08′03.3″E | *Lumbricus terrestris* | 15 |
| 24 | MU | 10/13/2019 | Soil from a garden, Moskovská ulica street, Staré mesto, Bratislava | 48°09′05.0″N 17°07′18.2″E | *Octolasion lacteovicinum* | 20 |
| 25 | BZ | 05/19/2018 | Soil from the east part of the Botanical Garden, Karlova Ves, Bratislava | 48°08′41.9″N 17°04′24.6″E | *Lumbricus terrestris* | 10 |

[a] *Dates are given as mo/d/yr.*

[b] *Identified as Eisenia fetida by Obert and Vďačný (2019) on the basis of morphological data. However, the identification within the E. fetida complex is specified to E. andrei given the mitochondrial COI and ND1 sequences*

**SUPPLEMENTARY TABLE 2 |** Primers used for amplification of five molecular markers analyzed in astome ciliates and their earthworm hosts.

| **Molecular marker** | **Organism group** | **Primer name** | **Primer sequence (in 5' to 3' direction)** | **Reference** |
|---|---|---|---|---|
| 18S rRNA gene | Astome ciliates | Euk A | AAC CTG GTT GAT CCT GCC AGT | Medlin et al. (1988) |
| | | Euk B | TGA TCC TTC TGC AGG TTC AC | Medlin et al. (1988) |
| 16S rRNA gene | Astome ciliates | 16S-mtSSU-F | TGT GCC AGC AGC CGC GGT AA | van Hoek et al. (2000) |
| | | 16S-mtSSU-R | CCC MTA CCR GTA CCT TGT GT | van Hoek et al. (2000) |
| ITS region and 28S rRNA gene[a] | Astome ciliates | ITS-F | GTA GGT GAA CCT GCG GAA GGA TCA TTA | Miao et al. (2008) |
| | | LO-R | GCT ATC CTG AGR GAA ACT TCG | Pawlowski (2000) |
| Cytochrome *c* oxidase subunit I | Astome ciliates | F388dT | TGT AAA ACG ACG GCC AGT GGW KCB AAA GAT GTW GC | Strüder-Kypke and Lynn (2010) |
| | | R1184dT | CAG GAA ACA GCT ATG ACT ADA CYT C | Strüder-Kypke and Lynn (2010) |
| | | F388dT-mod20 | GGT TCC AAA GAT GTT GCD TA | Present study[b] |
| | | R1184dT-mod21 | AGG GTG ACC GAA AAA TCA RAA | Present study[b] |
| | Earthworms | LCO 1490 | GGT CAA CAA ATC ATA AAG ATA TTG G | Folmer et al. (1994) |
| | | HCO 2198 | TAA ACT TCA GGG TGA CCA AAA AAT CA | Folmer et al. (1994) |
| NADH-ubiquinone oxidoreductase chain 1 | Earthworms | tRNA-Leu-ND1-LumbF2 | GAA TAG TGC CAC AGG TTT AAA C | Pérez-Losada et al. (2009) |
| | | tRNA-Leu-ND1-LumbR1b | TTA ACG TCA TCA GAG TTA TC | Pérez-Losada et al. (2009) |

[a] *The barcoding D1/D2 domains of the 28S rRNA gene were amplified.*

[b] *Based on conserved regions of astome COI sequences amplified with the F388dT and R1184dT primers.*

**SUPPLEMENTARY TABLE 3 |** Conditions of PCR reactions used for amplification of five molecular markers analyzed in astome ciliates and their earthworm hosts.

| Molecular marker | Organism group | PCR program | | | Reference |
|---|---|---|---|---|---|
| | | Initial denaturation | Cycling (denaturation, annealing, extension) | Final extension | |
| 18S rRNA gene | Astome ciliates | 95 °C/15 min | 30 cycles: 95 °C/45 s, 55 °C/60 s, 72 °C/150 s | 72 °C/10 min | Vďačný et al. (2011) |
| 16S rRNA gene | Astome ciliates | 94 °C/3 min | 5 cycles: 94 °C/30 s, 50 °C/60 s, 68 °C/75 s<br>35 cycles: 94 °C/30 s, 60 °C/60 s, 68 °C/75 s | 68 °C/10 min | Rataj and Vďačný (2020) |
| ITS region and 28S rRNA gene[a] | Astome ciliates | 95 °C/15 min | 35 cycles: 95 °C/45 s, 55 °C/60 s, 72 °C/150 s | 72 °C/10 min | Vďačný et al. (2011)[b] |
| Cytochrome *c* oxidase subunit I (COI) | Astome ciliates | 94 °C/4 min | 5 cycles: 94 °C/45 s, 45 °C/75 s, 72 °C/90 s<br>35 cycles: 94 °C/45 s, 55 °C/75 s, 72 °C/90 s | 72 °C/8 min | Rataj and Vďačný (2020) |
| | Earthworms | 95 °C/5 min | 40 cycles: 95 °C/30 s, 50 °C/90s, 72 °C/180 s | 72 °C/10 min | Kolicka (2019) |
| NADH-ubiquinone oxidoreductase chain 1 (ND1) | Earthworms | 94 °C/4 min | 5 cycles: 94 °C/45 s, 45 °C/75 s, 72 °C/90 s<br>35 cycles: 94 °C/45 s, 55 °C/75 s, 72 °C/90 s | 72 °C/8 min | Rataj and Vďačný (2020) |

[a] *The barcoding D1/D2 domains of the 28S rRNA gene were amplified.*

[b] *Modified from Vďačný et al. (2011) by adding five cycles.*

**SUPPLEMENTARY TABLE 4 |** List of astome ciliates with GenBank accession numbers of corresponding 18S rRNA gene sequences included in Phylogenetic Interaction-adjusted Similarity Analyses.

| No. | Taxon | GenBank entry |
|---|---|---|
| 1 | *Almophrya bivacuolata* | HQ446281 |
| 2 | *Anoplophrya allolobophorae* | MZ048824 |
| 3 | *Anoplophrya aporrectodeae* | MZ048825 |
| 4 | *Anoplophrya lumbrici* | MN121062 |
| 5 | *Anoplophrya marylandensis* | AY547546 |
| 6 | *Anoplophrya octolasionis* | MZ048828 |
| 7 | *Anoplophrya vulgaris* | MN121065 |
| 8 | *Eudrilophrya complanata* | HQ446280 |
| 9 | *Maupasella mucronata* | MW182008 |
| 10 | *Metaracoelophrya intermedia* | HQ446278 |
| 11 | *Metaracoelophrya* sp. | HQ446282 |
| 12 | *Metaracoelophrya* sp. | HQ446277 |
| 13 | *Metaradiophrya* sp. | HQ446279 |
| 14 | *Metaradiophrya chlorotica* | MZ048835 |
| 15 | *Metaradiophrya lumbrici* | MN121068 |
| 16 | *Metaradiophrya speculorum* | MW182012 |
| 17 | *Metaradiophrya varians* | MN121076 |
| 18 | *Njinella prolifera* | HQ446276 |
| 19 | *Paraclausilocola constricta* | HQ446275 |
| 20 | *Paraclausilocola elongata* | HQ446274 |
| 21 | *Subanoplophrya nodulata* | MN121063 |

**SUPPLEMENTARY TABLE 5 |** Pairwise *p*-distances of 18S rRNA gene sequences between astomes isolated from lumbricid earthworms.

| No. | Specimen | 1 | 2 | 3 | 4 | 5 | 6 | 7 | 8 | 9 | 10 | 11 | 12 | 13 | 14 | 15 | 16 | 17 | 18 | 19 | 20 | 21 | 22 | 23 | 24 | 25 | 26 | 27 | 28 | 29 | 30 | 31 | 32 | 33 | 34 | 35 | 36 | 37 | 38 |
|---|---|---|---|---|---|---|---|---|---|---|---|---|---|---|---|---|---|---|---|---|---|---|---|---|---|---|---|---|---|---|---|---|---|---|---|---|---|---|---|
| 1 | ***Anoplophrya allolobophorae* JA-3 37 ACH** | | | | | | | | | | | | | | | | | | | | | | | | | | | | | | | | | | | | | | |
| 2 | ***Anoplophrya aporrectodeae* PUz 17 AT** | 0.00171 | | | | | | | | | | | | | | | | | | | | | | | | | | | | | | | | | | | | | |
| 3 | ***Anoplophrya aporrectodeae* PUz 40 AT** | 0.00171 | 0.00000 | | | | | | | | | | | | | | | | | | | | | | | | | | | | | | | | | | | | |
| 4 | ***Anoplophrya aporrectodeae* PUz 41 AT** | 0.00171 | 0.00000 | 0.00000 | | | | | | | | | | | | | | | | | | | | | | | | | | | | | | | | | | | |
| 5 | ***Anoplophrya lumbrici* RZ 6 LT** | 0.01254 | 0.01425 | 0.01425 | 0.01425 | | | | | | | | | | | | | | | | | | | | | | | | | | | | | | | | | | |
| 6 | ***Anoplophrya lumbrici* KR 9 LT** | 0.01254 | 0.01425 | 0.01425 | 0.01425 | 0.00000 | | | | | | | | | | | | | | | | | | | | | | | | | | | | | | | | | |
| 7 | ***Anoplophrya lumbrici* KR 11 LT** | 0.01254 | 0.01425 | 0.01425 | 0.01425 | 0.00000 | 0.00000 | | | | | | | | | | | | | | | | | | | | | | | | | | | | | | | | |
| 8 | ***Anoplophrya octolasionis* MU 56 OL** | 0.00969 | 0.01026 | 0.01026 | 0.01026 | 0.00969 | 0.00969 | 0.00969 | | | | | | | | | | | | | | | | | | | | | | | | | | | | | | | |
| 9 | ***Anoplophrya octolasionis* MU 57 OL** | 0.00969 | 0.01026 | 0.01026 | 0.01026 | 0.00969 | 0.00969 | 0.00969 | 0.00000 | | | | | | | | | | | | | | | | | | | | | | | | | | | | | | |
| 10 | ***Anoplophrya octolasionis* MU 58 OL** | 0.00969 | 0.01026 | 0.01026 | 0.01026 | 0.00969 | 0.00969 | 0.00969 | 0.00000 | 0.00000 | | | | | | | | | | | | | | | | | | | | | | | | | | | | | |
| 11 | ***Anoplophrya vulgaris* BZ 13 EA** | 0.01600 | 0.01657 | 0.01657 | 0.01657 | 0.01601 | 0.01601 | 0.01601 | 0.01314 | 0.01314 | 0.01314 | | | | | | | | | | | | | | | | | | | | | | | | | | | | |
| 12 | ***Anoplophrya vulgaris* JA-1 18 EA** | 0.01600 | 0.01657 | 0.01657 | 0.01657 | 0.01601 | 0.01601 | 0.01601 | 0.01314 | 0.01314 | 0.01314 | 0.00000 | | | | | | | | | | | | | | | | | | | | | | | | | | | |
| 13 | ***Anoplophrya vulgaris* JA-1 20 EA** | 0.01600 | 0.01657 | 0.01657 | 0.01657 | 0.01601 | 0.01601 | 0.01601 | 0.01314 | 0.01314 | 0.01314 | 0.00000 | 0.00000 | | | | | | | | | | | | | | | | | | | | | | | | | | |
| 14 | ***Anoplophrya vulgaris* JA-1 21 EF** | 0.01600 | 0.01657 | 0.01657 | 0.01657 | 0.01601 | 0.01601 | 0.01601 | 0.01314 | 0.01314 | 0.01314 | 0.00000 | 0.00000 | 0.00000 | | | | | | | | | | | | | | | | | | | | | | | | | |
| 15 | ***Anoplophrya vulgaris* NG 27 DV** | 0.01600 | 0.01657 | 0.01657 | 0.01657 | 0.01601 | 0.01601 | 0.01601 | 0.01314 | 0.01314 | 0.01314 | 0.00000 | 0.00000 | 0.00000 | 0.00000 | | | | | | | | | | | | | | | | | | | | | | | | |
| 16 | ***Anoplophrya vulgaris* NG 28 DV** | 0.01600 | 0.01657 | 0.01657 | 0.01657 | 0.01601 | 0.01601 | 0.01601 | 0.01314 | 0.01314 | 0.01314 | 0.00000 | 0.00000 | 0.00000 | 0.00000 | 0.00000 | | | | | | | | | | | | | | | | | | | | | | | |
| 17 | ***Maupasella mucronata* KDo 33 ET** | 0.07094 | 0.07208 | 0.07208 | 0.07208 | 0.07556 | 0.07556 | 0.07556 | 0.07265 | 0.07265 | 0.07265 | 0.07393 | 0.07393 | 0.07393 | 0.07393 | 0.07393 | 0.07393 | | | | | | | | | | | | | | | | | | | | | | |
| 18 | ***Maupasella mucronata* KDo 34 ET** | 0.07094 | 0.07208 | 0.07208 | 0.07208 | 0.07556 | 0.07556 | 0.07556 | 0.07265 | 0.07265 | 0.07265 | 0.07393 | 0.07393 | 0.07393 | 0.07393 | 0.07393 | 0.07393 | 0.00000 | | | | | | | | | | | | | | | | | | | | | |
| 19 | ***Maupasella mucronata* KDo 35 ET** | 0.07094 | 0.07208 | 0.07208 | 0.07208 | 0.07556 | 0.07556 | 0.07556 | 0.07265 | 0.07265 | 0.07265 | 0.07393 | 0.07393 | 0.07393 | 0.07393 | 0.07393 | 0.07393 | 0.00000 | 0.00000 | | | | | | | | | | | | | | | | | | | | |
| 20 | ***Maupasella mucronata* KDo 36 ET** | 0.07094 | 0.07208 | 0.07208 | 0.07208 | 0.07556 | 0.07556 | 0.07556 | 0.07265 | 0.07265 | 0.07265 | 0.07393 | 0.07393 | 0.07393 | 0.07393 | 0.07393 | 0.07393 | 0.00000 | 0.00000 | 0.00000 | | | | | | | | | | | | | | | | | | | |
| 21 | ***Metaradiophrya chlorotica* JA-2 1M ACH** | 0.02738 | 0.02909 | 0.02909 | 0.02909 | 0.03082 | 0.03082 | 0.03082 | 0.02795 | 0.02795 | 0.02795 | 0.03086 | 0.03086 | 0.03086 | 0.03086 | 0.03086 | 0.03086 | 0.06663 | 0.06663 | 0.06663 | 0.06663 | | | | | | | | | | | | | | | | | | |
| 22 | ***Metaradiophrya chlorotica* JA-2 2M ACH** | 0.02738 | 0.02909 | 0.02909 | 0.02909 | 0.03082 | 0.03082 | 0.03082 | 0.02795 | 0.02795 | 0.02795 | 0.03086 | 0.03086 | 0.03086 | 0.03086 | 0.03086 | 0.03086 | 0.06663 | 0.06663 | 0.06663 | 0.06663 | 0.00000 | | | | | | | | | | | | | | | | | |
| 23 | ***Metaradiophrya chlorotica* JA-2 3M ACH** | 0.02738 | 0.02909 | 0.02909 | 0.02909 | 0.03082 | 0.03082 | 0.03082 | 0.02795 | 0.02795 | 0.02795 | 0.03086 | 0.03086 | 0.03086 | 0.03086 | 0.03086 | 0.03086 | 0.06663 | 0.06663 | 0.06663 | 0.06663 | 0.00000 | 0.00000 | | | | | | | | | | | | | | | | |
| 24 | ***Metaradiophrya lumbrici* RZ 4 LT** | 0.02624 | 0.02795 | 0.02795 | 0.02795 | 0.02968 | 0.02968 | 0.02968 | 0.02738 | 0.02738 | 0.02738 | 0.02857 | 0.02857 | 0.02857 | 0.02857 | 0.02857 | 0.02857 | 0.06549 | 0.06549 | 0.06549 | 0.06549 | 0.00795 | 0.00795 | 0.00795 | | | | | | | | | | | | | | | |
| 25 | ***Metaradiophrya lumbrici* RZ 5 LT** | 0.02624 | 0.02795 | 0.02795 | 0.02795 | 0.02968 | 0.02968 | 0.02968 | 0.02738 | 0.02738 | 0.02738 | 0.02857 | 0.02857 | 0.02857 | 0.02857 | 0.02857 | 0.02857 | 0.06549 | 0.06549 | 0.06549 | 0.06549 | 0.00795 | 0.00795 | 0.00795 | 0.00000 | | | | | | | | | | | | | | |
| 26 | ***Metaradiophrya lumbrici* KR 8 LT** | 0.02624 | 0.02795 | 0.02795 | 0.02795 | 0.02968 | 0.02968 | 0.02968 | 0.02738 | 0.02738 | 0.02738 | 0.02857 | 0.02857 | 0.02857 | 0.02857 | 0.02857 | 0.02857 | 0.06549 | 0.06549 | 0.06549 | 0.06549 | 0.00795 | 0.00795 | 0.00795 | 0.00000 | 0.00000 | | | | | | | | | | | | | |
| 27 | ***Metaradiophrya lumbrici* KR 10 LT** | 0.02624 | 0.02795 | 0.02795 | 0.02795 | 0.02968 | 0.02968 | 0.02968 | 0.02738 | 0.02738 | 0.02738 | 0.02857 | 0.02857 | 0.02857 | 0.02857 | 0.02857 | 0.02857 | 0.06549 | 0.06549 | 0.06549 | 0.06549 | 0.00795 | 0.00795 | 0.00795 | 0.00000 | 0.00000 | 0.00000 | | | | | | | | | | | | |
| 28 | ***Metaradiophrya lumbrici* JA-2 25 LT** | 0.02624 | 0.02795 | 0.02795 | 0.02795 | 0.02968 | 0.02968 | 0.02968 | 0.02738 | 0.02738 | 0.02738 | 0.02857 | 0.02857 | 0.02857 | 0.02857 | 0.02857 | 0.02857 | 0.06549 | 0.06549 | 0.06549 | 0.06549 | 0.00795 | 0.00795 | 0.00795 | 0.00000 | 0.00000 | 0.00000 | 0.00000 | | | | | | | | | | | |
| 29 | ***Metaradiophrya lumbrici* JA-2 26 LT** | 0.02624 | 0.02795 | 0.02795 | 0.02795 | 0.02968 | 0.02968 | 0.02968 | 0.02738 | 0.02738 | 0.02738 | 0.02857 | 0.02857 | 0.02857 | 0.02857 | 0.02857 | 0.02857 | 0.06549 | 0.06549 | 0.06549 | 0.06549 | 0.00795 | 0.00795 | 0.00795 | 0.00000 | 0.00000 | 0.00000 | 0.00000 | 0.00000 | | | | | | | | | | |
| 30 | ***Metaradiophrya speculorum* HkD 59 AT** | 0.02624 | 0.02681 | 0.02681 | 0.02681 | 0.03139 | 0.03139 | 0.03139 | 0.02738 | 0.02738 | 0.02738 | 0.02971 | 0.02971 | 0.02971 | 0.02971 | 0.02971 | 0.02971 | 0.06720 | 0.06720 | 0.06720 | 0.06720 | 0.00965 | 0.00965 | 0.00965 | 0.00624 | 0.00624 | 0.00624 | 0.00624 | 0.00624 | 0.00624 | | | | | | | | | |
| 31 | ***Metaradiophrya speculorum* HkD 60 AT** | 0.02624 | 0.02681 | 0.02681 | 0.02681 | 0.03139 | 0.03139 | 0.03139 | 0.02738 | 0.02738 | 0.02738 | 0.02971 | 0.02971 | 0.02971 | 0.02971 | 0.02971 | 0.02971 | 0.06720 | 0.06720 | 0.06720 | 0.06720 | 0.00965 | 0.00965 | 0.00965 | 0.00624 | 0.00624 | 0.00624 | 0.00624 | 0.00624 | 0.00624 | 0.00000 | | | | | | | | |
| 32 | ***Metaradiophrya varians* BZ 12 EA** | 0.03022 | 0.03079 | 0.03079 | 0.03079 | 0.03480 | 0.03480 | 0.03480 | 0.03136 | 0.03136 | 0.03136 | 0.03200 | 0.03200 | 0.03200 | 0.03200 | 0.03200 | 0.03200 | 0.06948 | 0.06948 | 0.06948 | 0.06948 | 0.01476 | 0.01476 | 0.01476 | 0.01305 | 0.01305 | 0.01305 | 0.01305 | 0.01305 | 0.01305 | 0.01022 | 0.01022 | | | | | | | |
| 33 | ***Metaradiophrya varians* BZ 14 EA** | 0.03022 | 0.03079 | 0.03079 | 0.03079 | 0.03480 | 0.03480 | 0.03480 | 0.03136 | 0.03136 | 0.03136 | 0.03200 | 0.03200 | 0.03200 | 0.03200 | 0.03200 | 0.03200 | 0.06948 | 0.06948 | 0.06948 | 0.06948 | 0.01476 | 0.01476 | 0.01476 | 0.01305 | 0.01305 | 0.01305 | 0.01305 | 0.01305 | 0.01305 | 0.01022 | 0.01022 | 0.00000 | | | | | | |
| 34 | ***Metaradiophrya varians* JA-1 19 EA** | 0.03022 | 0.03079 | 0.03079 | 0.03079 | 0.03480 | 0.03480 | 0.03480 | 0.03136 | 0.03136 | 0.03136 | 0.03200 | 0.03200 | 0.03200 | 0.03200 | 0.03200 | 0.03200 | 0.06948 | 0.06948 | 0.06948 | 0.06948 | 0.01476 | 0.01476 | 0.01476 | 0.01305 | 0.01305 | 0.01305 | 0.01305 | 0.01305 | 0.01305 | 0.01022 | 0.01022 | 0.00000 | 0.00000 | | | | | |
| 35 | ***Metaradiophrya varians* JA-1 22 EA** | 0.03022 | 0.03079 | 0.03079 | 0.03079 | 0.03480 | 0.03480 | 0.03480 | 0.03136 | 0.03136 | 0.03136 | 0.03200 | 0.03200 | 0.03200 | 0.03200 | 0.03200 | 0.03200 | 0.06948 | 0.06948 | 0.06948 | 0.06948 | 0.01476 | 0.01476 | 0.01476 | 0.01305 | 0.01305 | 0.01305 | 0.01305 | 0.01305 | 0.01305 | 0.01022 | 0.01022 | 0.00000 | 0.00000 | 0.00000 | | | | |
| 36 | ***Metaradiophrya varians* BZkv 31 EA** | 0.03022 | 0.03079 | 0.03079 | 0.03079 | 0.03480 | 0.03480 | 0.03480 | 0.03136 | 0.03136 | 0.03136 | 0.03200 | 0.03200 | 0.03200 | 0.03200 | 0.03200 | 0.03200 | 0.06948 | 0.06948 | 0.06948 | 0.06948 | 0.01476 | 0.01476 | 0.01476 | 0.01305 | 0.01305 | 0.01305 | 0.01305 | 0.01305 | 0.01305 | 0.01022 | 0.01022 | 0.00000 | 0.00000 | 0.00000 | 0.00000 | | | |
| 37 | ***Metaradiophrya varians* BZkv 32 EA** | 0.03022 | 0.03079 | 0.03079 | 0.03079 | 0.03480 | 0.03480 | 0.03480 | 0.03136 | 0.03136 | 0.03136 | 0.03200 | 0.03200 | 0.03200 | 0.03200 | 0.03200 | 0.03200 | 0.06948 | 0.06948 | 0.06948 | 0.06948 | 0.01476 | 0.01476 | 0.01476 | 0.01305 | 0.01305 | 0.01305 | 0.01305 | 0.01305 | 0.01305 | 0.01022 | 0.01022 | 0.00000 | 0.00000 | 0.00000 | 0.00000 | 0.00000 | | |
| 38 | ***Subanoplophrya nodulata* PU 29 OT** | 0.05711 | 0.05825 | 0.05825 | 0.05825 | 0.06057 | 0.06057 | 0.06057 | 0.06054 | 0.06054 | 0.06054 | 0.05839 | 0.05839 | 0.05839 | 0.05839 | 0.05839 | 0.05839 | 0.07070 | 0.07070 | 0.07070 | 0.07070 | 0.05410 | 0.05410 | 0.05410 | 0.05353 | 0.05353 | 0.05353 | 0.05353 | 0.05353 | 0.05353 | 0.05182 | 0.05182 | 0.05293 | 0.05293 | 0.05293 | 0.05293 | 0.05293 | 0.05293 | |
| 39 | ***Subanoplophrya nodulata* PU 30 OT** | 0.05711 | 0.05825 | 0.05825 | 0.05825 | 0.06057 | 0.06057 | 0.06057 | 0.06054 | 0.06054 | 0.06054 | 0.05839 | 0.05839 | 0.05839 | 0.05839 | 0.05839 | 0.05839 | 0.07070 | 0.07070 | 0.07070 | 0.07070 | 0.05410 | 0.05410 | 0.05410 | 0.05353 | 0.05353 | 0.05353 | 0.05353 | 0.05353 | 0.05353 | 0.05182 | 0.05182 | 0.05293 | 0.05293 | 0.05293 | 0.05293 | 0.05293 | 0.05293 | 0.00000 |

*Note: Specimen code consists of a locality code, an isolate code, and an abbreviation of host name. Locality codes are summarized in Supplementary Table 1.*

**SUPPLEMENTARY TABLE 6 |** Pairwise *p*-distances of ITS region sequences between astomes isolated from lumbricid earthworms.

| No. | Specimen | 1 | 2 | 3 | 4 | 5 | 6 | 7 | 8 | 9 | 10 | 11 | 12 | 13 | 14 | 15 | 16 | 17 | 18 | 19 | 20 | 21 | 22 | 23 | 24 | 25 | 26 | 27 | 28 | 29 | 30 | 31 | 32 | 33 | 34 | 35 | 36 | 37 | 38 |
|---|---|---|---|---|---|---|---|---|---|---|---|---|---|---|---|---|---|---|---|---|---|---|---|---|---|---|---|---|---|---|---|---|---|---|---|---|---|---|---|
| 1 | **_Anoplophrya allolobophorae_ JA-3 37 ACH** | | | | | | | | | | | | | | | | | | | | | | | | | | | | | | | | | | | | | | |
| 2 | **_Anoplophrya aporrectodeae_ PUz 17 AT** | 0.00964 | | | | | | | | | | | | | | | | | | | | | | | | | | | | | | | | | | | | | |
| 3 | **_Anoplophrya aporrectodeae_ PUz 40 AT** | 0.00964 | 0.00000 | | | | | | | | | | | | | | | | | | | | | | | | | | | | | | | | | | | | |
| 4 | **_Anoplophrya aporrectodeae_ PUz 41 AT** | 0.00964 | 0.00000 | 0.00000 | | | | | | | | | | | | | | | | | | | | | | | | | | | | | | | | | | | |
| 5 | **_Anoplophrya lumbrici_ RZ 6 LT** | 0.05556 | 0.05314 | 0.05314 | 0.05314 | | | | | | | | | | | | | | | | | | | | | | | | | | | | | | | | | | |
| 6 | **_Anoplophrya lumbrici_ KR 9 LT** | 0.05556 | 0.05314 | 0.05314 | 0.05314 | 0.00000 | | | | | | | | | | | | | | | | | | | | | | | | | | | | | | | | | |
| 7 | **_Anoplophrya lumbrici_ KR 11 LT** | 0.05556 | 0.05314 | 0.05314 | 0.05314 | 0.00000 | 0.00000 | | | | | | | | | | | | | | | | | | | | | | | | | | | | | | | | |
| 8 | **_Anoplophrya octolasionis_ MU 56 OL** | 0.06053 | 0.06053 | 0.06053 | 0.06053 | 0.03357 | 0.03357 | 0.03357 | | | | | | | | | | | | | | | | | | | | | | | | | | | | | | | |
| 9 | **_Anoplophrya octolasionis_ MU 57 OL** | 0.06053 | 0.06053 | 0.06053 | 0.06053 | 0.03357 | 0.03357 | 0.03357 | 0.00000 | | | | | | | | | | | | | | | | | | | | | | | | | | | | | | |
| 10 | **_Anoplophrya octolasionis_ MU 58 OL** | 0.06053 | 0.06053 | 0.06053 | 0.06053 | 0.03357 | 0.03357 | 0.03357 | 0.00000 | 0.00000 | | | | | | | | | | | | | | | | | | | | | | | | | | | | | |
| 11 | **_Anoplophrya vulgaris_ BZ 13 EA** | 0.07353 | 0.07353 | 0.07353 | 0.07353 | 0.06863 | 0.06863 | 0.06863 | 0.05897 | 0.05897 | 0.05897 | | | | | | | | | | | | | | | | | | | | | | | | | | | | |
| 12 | **_Anoplophrya vulgaris_ JA-1 18 EA** | 0.07353 | 0.07353 | 0.07353 | 0.07353 | 0.06863 | 0.06863 | 0.06863 | 0.05897 | 0.05897 | 0.05897 | 0.00000 | | | | | | | | | | | | | | | | | | | | | | | | | | | |
| 13 | **_Anoplophrya vulgaris_ JA-1 20 EA** | 0.07353 | 0.07353 | 0.07353 | 0.07353 | 0.06863 | 0.06863 | 0.06863 | 0.05897 | 0.05897 | 0.05897 | 0.00000 | 0.00000 | | | | | | | | | | | | | | | | | | | | | | | | | | |
| 14 | **_Anoplophrya vulgaris_ JA-1 21 EF** | 0.07353 | 0.07353 | 0.07353 | 0.07353 | 0.06863 | 0.06863 | 0.06863 | 0.05897 | 0.05897 | 0.05897 | 0.00000 | 0.00000 | 0.00000 | | | | | | | | | | | | | | | | | | | | | | | | | |
| 15 | **_Anoplophrya vulgaris_ NG 27 DV** | 0.07353 | 0.07353 | 0.07353 | 0.07353 | 0.06863 | 0.06863 | 0.06863 | 0.05897 | 0.05897 | 0.05897 | 0.00000 | 0.00000 | 0.00000 | 0.00000 | | | | | | | | | | | | | | | | | | | | | | | | |
| 16 | **_Anoplophrya vulgaris_ NG 28 DV** | 0.07353 | 0.07353 | 0.07353 | 0.07353 | 0.06863 | 0.06863 | 0.06863 | 0.05897 | 0.05897 | 0.05897 | 0.00000 | 0.00000 | 0.00000 | 0.00000 | 0.00000 | | | | | | | | | | | | | | | | | | | | | | | |
| 17 | **_Maupasella mucronata_ KDo 33 ET** | 0.27407 | 0.26914 | 0.26914 | 0.26914 | 0.25369 | 0.25369 | 0.25369 | 0.25679 | 0.25679 | 0.25679 | 0.26316 | 0.26316 | 0.26316 | 0.26316 | 0.26316 | 0.26316 | | | | | | | | | | | | | | | | | | | | | | |
| 18 | **_Maupasella mucronata_ KDo 34 ET** | 0.27407 | 0.26914 | 0.26914 | 0.26914 | 0.25369 | 0.25369 | 0.25369 | 0.25679 | 0.25679 | 0.25679 | 0.26316 | 0.26316 | 0.26316 | 0.26316 | 0.26316 | 0.26316 | 0.00000 | | | | | | | | | | | | | | | | | | | | | |
| 19 | **_Maupasella mucronata_ KDo 35 ET** | 0.27407 | 0.26914 | 0.26914 | 0.26914 | 0.25369 | 0.25369 | 0.25369 | 0.25679 | 0.25679 | 0.25679 | 0.26316 | 0.26316 | 0.26316 | 0.26316 | 0.26316 | 0.26316 | 0.00000 | 0.00000 | | | | | | | | | | | | | | | | | | | | |
| 20 | **_Maupasella mucronata_ KDo 36 ET** | 0.27407 | 0.26914 | 0.26914 | 0.26914 | 0.25369 | 0.25369 | 0.25369 | 0.25679 | 0.25679 | 0.25679 | 0.26316 | 0.26316 | 0.26316 | 0.26316 | 0.26316 | 0.26316 | 0.00000 | 0.00000 | 0.00000 | | | | | | | | | | | | | | | | | | | |
| 21 | **_Metaradiophrya chlorotica_ JA-2 1M ACH** | 0.15663 | 0.14940 | 0.14940 | 0.14940 | 0.13397 | 0.13397 | 0.13397 | 0.12950 | 0.12950 | 0.12950 | 0.14216 | 0.14216 | 0.14216 | 0.14216 | 0.14216 | 0.14216 | 0.26877 | 0.26877 | 0.26877 | 0.26877 | | | | | | | | | | | | | | | | | | |
| 22 | **_Metaradiophrya chlorotica_ JA-2 2M ACH** | 0.15663 | 0.14940 | 0.14940 | 0.14940 | 0.13397 | 0.13397 | 0.13397 | 0.12950 | 0.12950 | 0.12950 | 0.14216 | 0.14216 | 0.14216 | 0.14216 | 0.14216 | 0.14216 | 0.26877 | 0.26877 | 0.26877 | 0.26877 | 0.00000 | | | | | | | | | | | | | | | | | |
| 23 | **_Metaradiophrya chlorotica_ JA-2 3M ACH** | 0.15663 | 0.14940 | 0.14940 | 0.14940 | 0.13397 | 0.13397 | 0.13397 | 0.12950 | 0.12950 | 0.12950 | 0.14216 | 0.14216 | 0.14216 | 0.14216 | 0.14216 | 0.14216 | 0.26877 | 0.26877 | 0.26877 | 0.26877 | 0.00000 | 0.00000 | | | | | | | | | | | | | | | | |
| 24 | **_Metaradiophrya lumbrici_ RZ 4 LT** | 0.16748 | 0.16262 | 0.16262 | 0.16262 | 0.14183 | 0.14183 | 0.14183 | 0.13735 | 0.13735 | 0.13735 | 0.14461 | 0.14461 | 0.14461 | 0.14461 | 0.14461 | 0.14461 | 0.26098 | 0.26098 | 0.26098 | 0.26098 | 0.08768 | 0.08768 | 0.08768 | | | | | | | | | | | | | | | |
| 25 | **_Metaradiophrya lumbrici_ RZ 5 LT** | 0.16748 | 0.16262 | 0.16262 | 0.16262 | 0.14183 | 0.14183 | 0.14183 | 0.13735 | 0.13735 | 0.13735 | 0.14461 | 0.14461 | 0.14461 | 0.14461 | 0.14461 | 0.14461 | 0.26098 | 0.26098 | 0.26098 | 0.26098 | 0.08768 | 0.08768 | 0.08768 | 0.00000 | | | | | | | | | | | | | | |
| 26 | **_Metaradiophrya lumbrici_ KR 8 LT** | 0.16748 | 0.16262 | 0.16262 | 0.16262 | 0.14183 | 0.14183 | 0.14183 | 0.13735 | 0.13735 | 0.13735 | 0.14461 | 0.14461 | 0.14461 | 0.14461 | 0.14461 | 0.14461 | 0.26098 | 0.26098 | 0.26098 | 0.26098 | 0.08768 | 0.08768 | 0.08768 | 0.00000 | 0.00000 | | | | | | | | | | | | | |
| 27 | **_Metaradiophrya lumbrici_ KR 10 LT** | 0.16748 | 0.16262 | 0.16262 | 0.16262 | 0.14183 | 0.14183 | 0.14183 | 0.13735 | 0.13735 | 0.13735 | 0.14461 | 0.14461 | 0.14461 | 0.14461 | 0.14461 | 0.14461 | 0.26098 | 0.26098 | 0.26098 | 0.26098 | 0.08768 | 0.08768 | 0.08768 | 0.00000 | 0.00000 | 0.00000 | | | | | | | | | | | | |
| 28 | **_Metaradiophrya lumbrici_ JA-2 25 LT** | 0.16748 | 0.16262 | 0.16262 | 0.16262 | 0.14183 | 0.14183 | 0.14183 | 0.13735 | 0.13735 | 0.13735 | 0.14461 | 0.14461 | 0.14461 | 0.14461 | 0.14461 | 0.14461 | 0.26098 | 0.26098 | 0.26098 | 0.26098 | 0.08768 | 0.08768 | 0.08768 | 0.00000 | 0.00000 | 0.00000 | 0.00000 | | | | | | | | | | | |
| 29 | **_Metaradiophrya lumbrici_ JA-2 26 LT** | 0.16748 | 0.16262 | 0.16262 | 0.16262 | 0.14183 | 0.14183 | 0.14183 | 0.13735 | 0.13735 | 0.13735 | 0.14461 | 0.14461 | 0.14461 | 0.14461 | 0.14461 | 0.14461 | 0.26098 | 0.26098 | 0.26098 | 0.26098 | 0.08768 | 0.08768 | 0.08768 | 0.00000 | 0.00000 | 0.00000 | 0.00000 | 0.00000 | | | | | | | | | | |
| 30 | **_Metaradiophrya speculorum_ HkD 59 AT** | 0.14734 | 0.14251 | 0.14251 | 0.14251 | 0.12440 | 0.12440 | 0.12440 | 0.13429 | 0.13429 | 0.13429 | 0.13725 | 0.13725 | 0.13725 | 0.13725 | 0.13725 | 0.13725 | 0.26225 | 0.26225 | 0.26225 | 0.26225 | 0.07381 | 0.07381 | 0.07381 | 0.08612 | 0.08612 | 0.08612 | 0.08612 | 0.08612 | 0.08612 | | | | | | | | | |
| 31 | **_Metaradiophrya speculorum_ HkD 60 AT** | 0.14734 | 0.14251 | 0.14251 | 0.14251 | 0.12440 | 0.12440 | 0.12440 | 0.13429 | 0.13429 | 0.13429 | 0.13725 | 0.13725 | 0.13725 | 0.13725 | 0.13725 | 0.13725 | 0.26225 | 0.26225 | 0.26225 | 0.26225 | 0.07381 | 0.07381 | 0.07381 | 0.08612 | 0.08612 | 0.08612 | 0.08612 | 0.08612 | 0.08612 | 0.00000 | | | | | | | | |
| 32 | **_Metaradiophrya varians_ BZ 12 EA** | 0.15663 | 0.14940 | 0.14940 | 0.14940 | 0.13397 | 0.13397 | 0.13397 | 0.13189 | 0.13189 | 0.13189 | 0.14461 | 0.14461 | 0.14461 | 0.14461 | 0.14461 | 0.14461 | 0.25672 | 0.25672 | 0.25672 | 0.25672 | 0.07583 | 0.07583 | 0.07583 | 0.07416 | 0.07416 | 0.07416 | 0.07416 | 0.07416 | 0.07416 | 0.05238 | 0.05238 | | | | | | | |
| 33 | **_Metaradiophrya varians_ BZ 14 EA** | 0.15663 | 0.14940 | 0.14940 | 0.14940 | 0.13397 | 0.13397 | 0.13397 | 0.13189 | 0.13189 | 0.13189 | 0.14461 | 0.14461 | 0.14461 | 0.14461 | 0.14461 | 0.14461 | 0.25672 | 0.25672 | 0.25672 | 0.25672 | 0.07583 | 0.07583 | 0.07583 | 0.07416 | 0.07416 | 0.07416 | 0.07416 | 0.07416 | 0.07416 | 0.05238 | 0.05238 | 0.00000 | | | | | | |
| 34 | **_Metaradiophrya varians_ JA-1 19 EA** | 0.15663 | 0.14940 | 0.14940 | 0.14940 | 0.13397 | 0.13397 | 0.13397 | 0.13189 | 0.13189 | 0.13189 | 0.14461 | 0.14461 | 0.14461 | 0.14461 | 0.14461 | 0.14461 | 0.25672 | 0.25672 | 0.25672 | 0.25672 | 0.07583 | 0.07583 | 0.07583 | 0.07416 | 0.07416 | 0.07416 | 0.07416 | 0.07416 | 0.07416 | 0.05238 | 0.05238 | 0.00000 | 0.00000 | | | | | |
| 35 | **_Metaradiophrya varians_ JA-1 22 EA** | 0.15663 | 0.14940 | 0.14940 | 0.14940 | 0.13397 | 0.13397 | 0.13397 | 0.13189 | 0.13189 | 0.13189 | 0.14461 | 0.14461 | 0.14461 | 0.14461 | 0.14461 | 0.14461 | 0.25672 | 0.25672 | 0.25672 | 0.25672 | 0.07583 | 0.07583 | 0.07583 | 0.07416 | 0.07416 | 0.07416 | 0.07416 | 0.07416 | 0.07416 | 0.05238 | 0.05238 | 0.00000 | 0.00000 | 0.00000 | | | | |
| 36 | **_Metaradiophrya varians_ BZkv 31 EA** | 0.15663 | 0.14940 | 0.14940 | 0.14940 | 0.13397 | 0.13397 | 0.13397 | 0.13189 | 0.13189 | 0.13189 | 0.14461 | 0.14461 | 0.14461 | 0.14461 | 0.14461 | 0.14461 | 0.25672 | 0.25672 | 0.25672 | 0.25672 | 0.07583 | 0.07583 | 0.07583 | 0.07416 | 0.07416 | 0.07416 | 0.07416 | 0.07416 | 0.07416 | 0.05238 | 0.05238 | 0.00000 | 0.00000 | 0.00000 | 0.00000 | | | |
| 37 | **_Metaradiophrya varians_ BZkv 32 EA** | 0.15663 | 0.14940 | 0.14940 | 0.14940 | 0.13397 | 0.13397 | 0.13397 | 0.13189 | 0.13189 | 0.13189 | 0.14461 | 0.14461 | 0.14461 | 0.14461 | 0.14461 | 0.14461 | 0.25672 | 0.25672 | 0.25672 | 0.25672 | 0.07583 | 0.07583 | 0.07583 | 0.07416 | 0.07416 | 0.07416 | 0.07416 | 0.07416 | 0.07416 | 0.05238 | 0.05238 | 0.00000 | 0.00000 | 0.00000 | 0.00000 | 0.00000 | | |
| 38 | **_Subanoplophrya nodulata_ PU 29 OT** | 0.27603 | 0.27603 | 0.27603 | 0.27603 | 0.26024 | 0.26024 | 0.26024 | 0.26812 | 0.26812 | 0.26812 | 0.25369 | 0.25369 | 0.25369 | 0.25369 | 0.25369 | 0.25369 | 0.28293 | 0.28293 | 0.28293 | 0.28293 | 0.25891 | 0.25891 | 0.25891 | 0.23501 | 0.23501 | 0.23501 | 0.23501 | 0.23501 | 0.23501 | 0.24221 | 0.24221 | 0.26014 | 0.26014 | 0.26014 | 0.26014 | 0.26014 | 0.26014 | |
| 39 | **_Subanoplophrya nodulata_ PU 30 OT** | 0.27603 | 0.27603 | 0.27603 | 0.27603 | 0.26024 | 0.26024 | 0.26024 | 0.26812 | 0.26812 | 0.26812 | 0.25369 | 0.25369 | 0.25369 | 0.25369 | 0.25369 | 0.25369 | 0.28293 | 0.28293 | 0.28293 | 0.28293 | 0.25891 | 0.25891 | 0.25891 | 0.23501 | 0.23501 | 0.23501 | 0.23501 | 0.23501 | 0.23501 | 0.24221 | 0.24221 | 0.26014 | 0.26014 | 0.26014 | 0.26014 | 0.26014 | 0.26014 | 0.00000 |

*Note: Specimen code consists of a locality code, an isolate code, and an abbreviation of host name. Locality codes are summarized in Supplementary Table 1.*

**SUPPLEMENTARY TABLE 7 |** Pairwise *p*-distances of the barcoding domains D1/D2 of the 28S rRNA gene between astomes isolated from lumbricid earthworms.

| No. | Specimen | 1 | 2 | 3 | 4 | 5 | 6 | 7 | 8 | 9 | 10 | 11 | 12 | 13 | 14 | 15 | 16 | 17 | 18 | 19 | 20 | 21 | 22 | 23 | 24 | 25 | 26 | 27 | 28 | 29 | 30 | 31 | 32 | 33 | 34 | 35 | 36 | 37 | 38 |
|---|---|---|---|---|---|---|---|---|---|---|---|---|---|---|---|---|---|---|---|---|---|---|---|---|---|---|---|---|---|---|---|---|---|---|---|---|---|---|---|
| 1 | ***Anoplophrya allolobophorae* JA-3 37 ACH** | | | | | | | | | | | | | | | | | | | | | | | | | | | | | | | | | | | | | | |
| 2 | ***Anoplophrya aporrectodeae* PUz 17 AT** | 0.00326 | | | | | | | | | | | | | | | | | | | | | | | | | | | | | | | | | | | | | |
| 3 | ***Anoplophrya aporrectodeae* PUz 40 AT** | 0.00326 | 0.00000 | | | | | | | | | | | | | | | | | | | | | | | | | | | | | | | | | | | | |
| 4 | ***Anoplophrya aporrectodeae* PUz 41 AT** | 0.00326 | 0.00000 | 0.00000 | | | | | | | | | | | | | | | | | | | | | | | | | | | | | | | | | | | |
| 5 | ***Anoplophrya lumbrici* RZ 6 LT** | 0.02285 | 0.01959 | 0.01959 | 0.01959 | | | | | | | | | | | | | | | | | | | | | | | | | | | | | | | | | | |
| 6 | ***Anoplophrya lumbrici* KR 9 LT** | 0.02285 | 0.01959 | 0.01959 | 0.01959 | 0.00000 | | | | | | | | | | | | | | | | | | | | | | | | | | | | | | | | | |
| 7 | ***Anoplophrya lumbrici* KR 11 LT** | 0.02285 | 0.01959 | 0.01959 | 0.01959 | 0.00000 | 0.00000 | | | | | | | | | | | | | | | | | | | | | | | | | | | | | | | | |
| 8 | ***Anoplophrya octolasionis* MU 56 OL** | 0.01741 | 0.01850 | 0.01850 | 0.01850 | 0.02176 | 0.02176 | 0.02176 | | | | | | | | | | | | | | | | | | | | | | | | | | | | | | | |
| 9 | ***Anoplophrya octolasionis* MU 57 OL** | 0.01741 | 0.01850 | 0.01850 | 0.01850 | 0.02176 | 0.02176 | 0.02176 | 0.00000 | | | | | | | | | | | | | | | | | | | | | | | | | | | | | | |
| 10 | ***Anoplophrya octolasionis* MU 58 OL** | 0.01850 | 0.01959 | 0.01959 | 0.01959 | 0.02285 | 0.02285 | 0.02285 | 0.00109 | 0.00109 | | | | | | | | | | | | | | | | | | | | | | | | | | | | | |
| 11 | ***Anoplophrya vulgaris* BZ 13 EA** | 0.02832 | 0.02941 | 0.02941 | 0.02941 | 0.04139 | 0.04139 | 0.04139 | 0.03595 | 0.03595 | 0.03704 | | | | | | | | | | | | | | | | | | | | | | | | | | | | |
| 12 | ***Anoplophrya vulgaris* JA-1 18 EA** | 0.02832 | 0.02941 | 0.02941 | 0.02941 | 0.04139 | 0.04139 | 0.04139 | 0.03595 | 0.03595 | 0.03704 | 0.00000 | | | | | | | | | | | | | | | | | | | | | | | | | | | |
| 13 | ***Anoplophrya vulgaris* JA-1 20 EA** | 0.02832 | 0.02941 | 0.02941 | 0.02941 | 0.04139 | 0.04139 | 0.04139 | 0.03595 | 0.03595 | 0.03704 | 0.00000 | 0.00000 | | | | | | | | | | | | | | | | | | | | | | | | | | |
| 14 | ***Anoplophrya vulgaris* JA-1 21 EF** | 0.02832 | 0.02941 | 0.02941 | 0.02941 | 0.04139 | 0.04139 | 0.04139 | 0.03595 | 0.03595 | 0.03704 | 0.00000 | 0.00000 | 0.00000 | | | | | | | | | | | | | | | | | | | | | | | | | |
| 15 | ***Anoplophrya vulgaris* NG 27 DV** | 0.02832 | 0.02941 | 0.02941 | 0.02941 | 0.04139 | 0.04139 | 0.04139 | 0.03595 | 0.03595 | 0.03704 | 0.00000 | 0.00000 | 0.00000 | 0.00000 | | | | | | | | | | | | | | | | | | | | | | | | |
| 16 | ***Anoplophrya vulgaris* NG 28 DV** | 0.02832 | 0.02941 | 0.02941 | 0.02941 | 0.04139 | 0.04139 | 0.04139 | 0.03595 | 0.03595 | 0.03704 | 0.00000 | 0.00000 | 0.00000 | 0.00000 | 0.00000 | | | | | | | | | | | | | | | | | | | | | | | |
| 17 | ***Maupasella mucronata* KDo 33 ET** | 0.16031 | 0.16249 | 0.16249 | 0.16249 | 0.16903 | 0.16903 | 0.16903 | 0.16249 | 0.16249 | 0.16358 | 0.15939 | 0.15939 | 0.15939 | 0.15939 | 0.15939 | 0.15939 | | | | | | | | | | | | | | | | | | | | | | |
| 18 | ***Maupasella mucronata* KDo 34 ET** | 0.16031 | 0.16249 | 0.16249 | 0.16249 | 0.16903 | 0.16903 | 0.16903 | 0.16249 | 0.16249 | 0.16358 | 0.15939 | 0.15939 | 0.15939 | 0.15939 | 0.15939 | 0.15939 | 0.00000 | | | | | | | | | | | | | | | | | | | | | |
| 19 | ***Maupasella mucronata* KDo 35 ET** | 0.16031 | 0.16249 | 0.16249 | 0.16249 | 0.16903 | 0.16903 | 0.16903 | 0.16249 | 0.16249 | 0.16358 | 0.15939 | 0.15939 | 0.15939 | 0.15939 | 0.15939 | 0.15939 | 0.00000 | 0.00000 | | | | | | | | | | | | | | | | | | | | |
| 20 | ***Maupasella mucronata* KDo 36 ET** | 0.16031 | 0.16249 | 0.16249 | 0.16249 | 0.16903 | 0.16903 | 0.16903 | 0.16249 | 0.16249 | 0.16358 | 0.15939 | 0.15939 | 0.15939 | 0.15939 | 0.15939 | 0.15939 | 0.00000 | 0.00000 | 0.00000 | | | | | | | | | | | | | | | | | | | |
| 21 | ***Metaradiophrya chlorotica* JA-2 1M ACH** | 0.04788 | 0.04679 | 0.04679 | 0.04679 | 0.05332 | 0.05332 | 0.05332 | 0.05332 | 0.05332 | 0.05441 | 0.05773 | 0.05773 | 0.05773 | 0.05773 | 0.05773 | 0.05773 | 0.15142 | 0.15142 | 0.15142 | 0.15142 | | | | | | | | | | | | | | | | | | |
| 22 | ***Metaradiophrya chlorotica* JA-2 2M ACH** | 0.04788 | 0.04679 | 0.04679 | 0.04679 | 0.05332 | 0.05332 | 0.05332 | 0.05332 | 0.05332 | 0.05441 | 0.05773 | 0.05773 | 0.05773 | 0.05773 | 0.05773 | 0.05773 | 0.15142 | 0.15142 | 0.15142 | 0.15142 | 0.00000 | | | | | | | | | | | | | | | | | |
| 23 | ***Metaradiophrya chlorotica* JA-2 3M ACH** | 0.04788 | 0.04679 | 0.04679 | 0.04679 | 0.05332 | 0.05332 | 0.05332 | 0.05332 | 0.05332 | 0.05441 | 0.05773 | 0.05773 | 0.05773 | 0.05773 | 0.05773 | 0.05773 | 0.15142 | 0.15142 | 0.15142 | 0.15142 | 0.00000 | 0.00000 | | | | | | | | | | | | | | | | |
| 24 | ***Metaradiophrya lumbrici* RZ 4 LT** | 0.05229 | 0.05338 | 0.05338 | 0.05338 | 0.05229 | 0.05229 | 0.05229 | 0.05120 | 0.05120 | 0.05011 | 0.06652 | 0.06652 | 0.06652 | 0.06652 | 0.06652 | 0.06652 | 0.15577 | 0.15577 | 0.15577 | 0.15577 | 0.02285 | 0.02285 | 0.02285 | | | | | | | | | | | | | | | |
| 25 | ***Metaradiophrya lumbrici* RZ 5 LT** | 0.05229 | 0.05338 | 0.05338 | 0.05338 | 0.05229 | 0.05229 | 0.05229 | 0.05120 | 0.05120 | 0.05011 | 0.06652 | 0.06652 | 0.06652 | 0.06652 | 0.06652 | 0.06652 | 0.15577 | 0.15577 | 0.15577 | 0.15577 | 0.02285 | 0.02285 | 0.02285 | 0.00000 | | | | | | | | | | | | | | |
| 26 | ***Metaradiophrya lumbrici* KR 8 LT** | 0.05229 | 0.05338 | 0.05338 | 0.05338 | 0.05229 | 0.05229 | 0.05229 | 0.05120 | 0.05120 | 0.05011 | 0.06652 | 0.06652 | 0.06652 | 0.06652 | 0.06652 | 0.06652 | 0.15577 | 0.15577 | 0.15577 | 0.15577 | 0.02285 | 0.02285 | 0.02285 | 0.00000 | 0.00000 | | | | | | | | | | | | | |
| 27 | ***Metaradiophrya lumbrici* KR 10 LT** | 0.05229 | 0.05338 | 0.05338 | 0.05338 | 0.05229 | 0.05229 | 0.05229 | 0.05120 | 0.05120 | 0.05011 | 0.06652 | 0.06652 | 0.06652 | 0.06652 | 0.06652 | 0.06652 | 0.15577 | 0.15577 | 0.15577 | 0.15577 | 0.02285 | 0.02285 | 0.02285 | 0.00000 | 0.00000 | 0.00000 | | | | | | | | | | | | |
| 28 | ***Metaradiophrya lumbrici* JA-2 25 LT** | 0.05229 | 0.05338 | 0.05338 | 0.05338 | 0.05229 | 0.05229 | 0.05229 | 0.05120 | 0.05120 | 0.05011 | 0.06652 | 0.06652 | 0.06652 | 0.06652 | 0.06652 | 0.06652 | 0.15577 | 0.15577 | 0.15577 | 0.15577 | 0.02285 | 0.02285 | 0.02285 | 0.00000 | 0.00000 | 0.00000 | 0.00000 | | | | | | | | | | | |
| 29 | ***Metaradiophrya lumbrici* JA-2 26 LT** | 0.05229 | 0.05338 | 0.05338 | 0.05338 | 0.05229 | 0.05229 | 0.05229 | 0.05120 | 0.05120 | 0.05011 | 0.06652 | 0.06652 | 0.06652 | 0.06652 | 0.06652 | 0.06652 | 0.15577 | 0.15577 | 0.15577 | 0.15577 | 0.02285 | 0.02285 | 0.02285 | 0.00000 | 0.00000 | 0.00000 | 0.00000 | 0.00000 | | | | | | | | | | |
| 30 | ***Metaradiophrya speculorum* HkD 59 AT** | 0.04913 | 0.04803 | 0.04803 | 0.04803 | 0.05131 | 0.05131 | 0.05131 | 0.05022 | 0.05022 | 0.04913 | 0.06230 | 0.06230 | 0.06230 | 0.06230 | 0.06230 | 0.06230 | 0.15082 | 0.15082 | 0.15082 | 0.15082 | 0.02290 | 0.02290 | 0.02290 | 0.01528 | 0.01528 | 0.01528 | 0.01528 | 0.01528 | 0.01528 | | | | | | | | | |
| 31 | ***Metaradiophrya speculorum* HkD 60 AT** | 0.05022 | 0.04913 | 0.04913 | 0.04913 | 0.05240 | 0.05240 | 0.05240 | 0.05131 | 0.05131 | 0.05022 | 0.06339 | 0.06339 | 0.06339 | 0.06339 | 0.06339 | 0.06339 | 0.15191 | 0.15191 | 0.15191 | 0.15191 | 0.02399 | 0.02399 | 0.02399 | 0.01638 | 0.01638 | 0.01638 | 0.01638 | 0.01638 | 0.01638 | 0.00109 | | | | | | | | |
| 32 | ***Metaradiophrya varians* BZ 12 EA** | 0.05882 | 0.05773 | 0.05773 | 0.05773 | 0.05556 | 0.05556 | 0.05556 | 0.05447 | 0.05447 | 0.05338 | 0.06870 | 0.06870 | 0.06870 | 0.06870 | 0.06870 | 0.06870 | 0.15359 | 0.15359 | 0.15359 | 0.15359 | 0.03917 | 0.03917 | 0.03917 | 0.02938 | 0.02938 | 0.02938 | 0.02938 | 0.02938 | 0.02938 | 0.02511 | 0.02620 | | | | | | | |
| 33 | ***Metaradiophrya varians* BZ 14 EA** | 0.05882 | 0.05773 | 0.05773 | 0.05773 | 0.05556 | 0.05556 | 0.05556 | 0.05447 | 0.05447 | 0.05338 | 0.06870 | 0.06870 | 0.06870 | 0.06870 | 0.06870 | 0.06870 | 0.15359 | 0.15359 | 0.15359 | 0.15359 | 0.03917 | 0.03917 | 0.03917 | 0.02938 | 0.02938 | 0.02938 | 0.02938 | 0.02938 | 0.02938 | 0.02511 | 0.02620 | 0.00000 | | | | | | |
| 34 | ***Metaradiophrya varians* JA-1 19 EA** | 0.05882 | 0.05773 | 0.05773 | 0.05773 | 0.05556 | 0.05556 | 0.05556 | 0.05447 | 0.05447 | 0.05338 | 0.06870 | 0.06870 | 0.06870 | 0.06870 | 0.06870 | 0.06870 | 0.15359 | 0.15359 | 0.15359 | 0.15359 | 0.03917 | 0.03917 | 0.03917 | 0.02938 | 0.02938 | 0.02938 | 0.02938 | 0.02938 | 0.02938 | 0.02511 | 0.02620 | 0.00000 | 0.00000 | | | | | |
| 35 | ***Metaradiophrya varians* JA-1 22 EA** | 0.05882 | 0.05773 | 0.05773 | 0.05773 | 0.05556 | 0.05556 | 0.05556 | 0.05447 | 0.05447 | 0.05338 | 0.06870 | 0.06870 | 0.06870 | 0.06870 | 0.06870 | 0.06870 | 0.15359 | 0.15359 | 0.15359 | 0.15359 | 0.03917 | 0.03917 | 0.03917 | 0.02938 | 0.02938 | 0.02938 | 0.02938 | 0.02938 | 0.02938 | 0.02511 | 0.02620 | 0.00000 | 0.00000 | 0.00000 | | | | |
| 36 | ***Metaradiophrya varians* BZkv 31 EA** | 0.05882 | 0.05773 | 0.05773 | 0.05773 | 0.05556 | 0.05556 | 0.05556 | 0.05447 | 0.05447 | 0.05338 | 0.06870 | 0.06870 | 0.06870 | 0.06870 | 0.06870 | 0.06870 | 0.15359 | 0.15359 | 0.15359 | 0.15359 | 0.03917 | 0.03917 | 0.03917 | 0.02938 | 0.02938 | 0.02938 | 0.02938 | 0.02938 | 0.02938 | 0.02511 | 0.02620 | 0.00000 | 0.00000 | 0.00000 | 0.00000 | | | |
| 37 | ***Metaradiophrya varians* BZkv 32 EA** | 0.05882 | 0.05773 | 0.05773 | 0.05773 | 0.05556 | 0.05556 | 0.05556 | 0.05447 | 0.05447 | 0.05338 | 0.06870 | 0.06870 | 0.06870 | 0.06870 | 0.06870 | 0.06870 | 0.15359 | 0.15359 | 0.15359 | 0.15359 | 0.03917 | 0.03917 | 0.03917 | 0.02938 | 0.02938 | 0.02938 | 0.02938 | 0.02938 | 0.02938 | 0.02511 | 0.02620 | 0.00000 | 0.00000 | 0.00000 | 0.00000 | 0.00000 | | |
| 38 | ***Subanoplophrya nodulata* PU 29 OT** | 0.15580 | 0.15470 | 0.15470 | 0.15470 | 0.15470 | 0.15470 | 0.15470 | 0.15028 | 0.15028 | 0.15028 | 0.16022 | 0.16022 | 0.16022 | 0.16022 | 0.16022 | 0.16022 | 0.19027 | 0.19027 | 0.19027 | 0.19027 | 0.14459 | 0.14459 | 0.14459 | 0.14254 | 0.14254 | 0.14254 | 0.14254 | 0.14254 | 0.14254 | 0.13621 | 0.13621 | 0.14033 | 0.14033 | 0.14033 | 0.14033 | 0.14033 | 0.14033 | |
| 39 | ***Subanoplophrya nodulata* PU 30 OT** | 0.15691 | 0.15580 | 0.15580 | 0.15580 | 0.15580 | 0.15580 | 0.15580 | 0.15138 | 0.15138 | 0.15138 | 0.16133 | 0.16133 | 0.16133 | 0.16133 | 0.16133 | 0.16133 | 0.19137 | 0.19137 | 0.19137 | 0.19137 | 0.14570 | 0.14570 | 0.14570 | 0.14365 | 0.14365 | 0.14365 | 0.14365 | 0.14365 | 0.14365 | 0.13732 | 0.13732 | 0.14144 | 0.14144 | 0.14144 | 0.14144 | 0.14144 | 0.14144 | 0.00110 |

*Note: Specimen code consists of a locality code, an isolate code, and an abbreviation of host name. Locality codes are summarized in Supplementary Table 1.*

**SUPPLEMENTARY TABLE 8 |** Pairwise *p*-distances of 16S rRNA gene sequences between astomes isolated from lumbricid earthworms.

| No. | Specimen | 1 | 2 | 3 | 4 | 5 | 6 | 7 | 8 | 9 | 10 | 11 | 12 | 13 | 14 | 15 | 16 | 17 | 18 | 19 | 20 | 21 | 22 | 23 | 24 | 25 | 26 | 27 | 28 | 29 | 30 | 31 | 32 | 33 | 34 |
|---|---|---|---|---|---|---|---|---|---|---|---|---|---|---|---|---|---|---|---|---|---|---|---|---|---|---|---|---|---|---|---|---|---|---|---|
| 1 | ***Anoplophrya allolobophorae* JA-3 37 ACH** | | | | | | | | | | | | | | | | | | | | | | | | | | | | | | | | | | |
| 2 | ***Anoplophrya aporrectodeae* PUz 17 AT** | 0.10624 | | | | | | | | | | | | | | | | | | | | | | | | | | | | | | | | | |
| 3 | ***Anoplophrya aporrectodeae* PUz 40 AT** | 0.10624 | 0.00000 | | | | | | | | | | | | | | | | | | | | | | | | | | | | | | | | |
| 4 | ***Anoplophrya aporrectodeae* PUz 41 AT** | 0.10624 | 0.00000 | 0.00000 | | | | | | | | | | | | | | | | | | | | | | | | | | | | | | | |
| 5 | ***Anoplophrya lumbrici* RZ 6 LT** | 0.20185 | 0.17803 | 0.17803 | 0.17803 | | | | | | | | | | | | | | | | | | | | | | | | | | | | | | |
| 6 | ***Anoplophrya lumbrici* KR 9 LT** | 0.20069 | 0.17803 | 0.17803 | 0.17803 | 0.00113 | | | | | | | | | | | | | | | | | | | | | | | | | | | | | |
| 7 | ***Anoplophrya lumbrici* KR 11 LT** | 0.20069 | 0.17803 | 0.17803 | 0.17803 | 0.00113 | 0.00000 | | | | | | | | | | | | | | | | | | | | | | | | | | | | |
| 8 | ***Anoplophrya octolasionis* MU 56 OL** | 0.16975 | 0.16416 | 0.16416 | 0.16416 | 0.17640 | 0.17526 | 0.17526 | | | | | | | | | | | | | | | | | | | | | | | | | | | |
| 9 | ***Anoplophrya octolasionis* MU 57 OL** | 0.16975 | 0.16416 | 0.16416 | 0.16416 | 0.17640 | 0.17526 | 0.17526 | 0.00000 | | | | | | | | | | | | | | | | | | | | | | | | | | |
| 10 | ***Anoplophrya octolasionis* MU 58 OL** | 0.16975 | 0.16416 | 0.16416 | 0.16416 | 0.17640 | 0.17526 | 0.17526 | 0.00000 | 0.00000 | | | | | | | | | | | | | | | | | | | | | | | | | |
| 11 | ***Anoplophrya vulgaris* BZ 13 EA** | 0.20281 | 0.20447 | 0.20447 | 0.20447 | 0.19396 | 0.19280 | 0.19280 | 0.18553 | 0.18553 | 0.18553 | | | | | | | | | | | | | | | | | | | | | | | | |
| 12 | ***Anoplophrya vulgaris* JA-1 18 EA** | 0.20281 | 0.20447 | 0.20447 | 0.20447 | 0.19396 | 0.19280 | 0.19280 | 0.18553 | 0.18553 | 0.18553 | 0.00000 | | | | | | | | | | | | | | | | | | | | | | | |
| 13 | ***Anoplophrya vulgaris* JA-1 20 EA** | 0.20281 | 0.20447 | 0.20447 | 0.20447 | 0.19396 | 0.19280 | 0.19280 | 0.18553 | 0.18553 | 0.18553 | 0.00000 | 0.00000 | | | | | | | | | | | | | | | | | | | | | | |
| 14 | ***Anoplophrya vulgaris* JA-1 21 EF** | 0.20164 | 0.20329 | 0.20329 | 0.20329 | 0.19280 | 0.19164 | 0.19164 | 0.18436 | 0.18436 | 0.18436 | 0.00116 | 0.00116 | 0.00116 | | | | | | | | | | | | | | | | | | | | | |
| 15 | ***Anoplophrya vulgaris* NG 27 DV** | 0.18852 | 0.19601 | 0.19601 | 0.19601 | 0.19026 | 0.18910 | 0.18910 | 0.17949 | 0.17949 | 0.17949 | 0.06597 | 0.06597 | 0.06597 | 0.06481 | | | | | | | | | | | | | | | | | | | | |
| 16 | ***Anoplophrya vulgaris* NG 28 DV** | 0.18852 | 0.19601 | 0.19601 | 0.19601 | 0.19026 | 0.18910 | 0.18910 | 0.17949 | 0.17949 | 0.17949 | 0.06597 | 0.06597 | 0.06597 | 0.06481 | 0.00000 | | | | | | | | | | | | | | | | | | | |
| 17 | ***Metaradiophrya chlorotica* JA-2 1M ACH** | 0.25234 | 0.25527 | 0.25527 | 0.25527 | 0.26212 | 0.26097 | 0.26097 | 0.23660 | 0.23660 | 0.23660 | 0.27660 | 0.27660 | 0.27660 | 0.27541 | 0.26564 | 0.26564 | | | | | | | | | | | | | | | | | | |
| 18 | ***Metaradiophrya chlorotica* JA-2 2M ACH** | 0.25234 | 0.25527 | 0.25527 | 0.25527 | 0.26212 | 0.26097 | 0.26097 | 0.23660 | 0.23660 | 0.23660 | 0.27660 | 0.27660 | 0.27660 | 0.27541 | 0.26564 | 0.26564 | 0.00000 | | | | | | | | | | | | | | | | | |
| 19 | ***Metaradiophrya chlorotica* JA-2 3M ACH** | 0.25234 | 0.25527 | 0.25527 | 0.25527 | 0.26212 | 0.26097 | 0.26097 | 0.23660 | 0.23660 | 0.23660 | 0.27660 | 0.27660 | 0.27660 | 0.27541 | 0.26564 | 0.26564 | 0.00000 | 0.00000 | | | | | | | | | | | | | | | | |
| 20 | ***Metaradiophrya lumbrici* RZ 4 LT** | 0.25029 | 0.23910 | 0.23910 | 0.23910 | 0.26163 | 0.26163 | 0.26163 | 0.25176 | 0.25176 | 0.25176 | 0.26154 | 0.26154 | 0.26154 | 0.26036 | 0.26005 | 0.26005 | 0.16163 | 0.16163 | 0.16163 | | | | | | | | | | | | | | | |
| 21 | ***Metaradiophrya lumbrici* RZ 5 LT** | 0.25029 | 0.23910 | 0.23910 | 0.23910 | 0.26163 | 0.26163 | 0.26163 | 0.25176 | 0.25176 | 0.25176 | 0.26154 | 0.26154 | 0.26154 | 0.26036 | 0.26005 | 0.26005 | 0.16163 | 0.16163 | 0.16163 | 0.00000 | | | | | | | | | | | | | | |
| 22 | ***Metaradiophrya lumbrici* KR 8 LT** | 0.25029 | 0.24146 | 0.24146 | 0.24146 | 0.26279 | 0.26279 | 0.26279 | 0.25410 | 0.25410 | 0.25410 | 0.26154 | 0.26154 | 0.26154 | 0.26036 | 0.26005 | 0.26005 | 0.16512 | 0.16512 | 0.16512 | 0.00569 | 0.00569 | | | | | | | | | | | | | |
| 23 | ***Metaradiophrya lumbrici* KR 10 LT** | 0.25029 | 0.24146 | 0.24146 | 0.24146 | 0.26279 | 0.26279 | 0.26279 | 0.25410 | 0.25410 | 0.25410 | 0.26154 | 0.26154 | 0.26154 | 0.26036 | 0.26005 | 0.26005 | 0.16512 | 0.16512 | 0.16512 | 0.00569 | 0.00569 | 0.00000 | | | | | | | | | | | | |
| 24 | ***Metaradiophrya lumbrici* JA-2 25 LT** | 0.25029 | 0.23910 | 0.23910 | 0.23910 | 0.26163 | 0.26163 | 0.26163 | 0.25176 | 0.25176 | 0.25176 | 0.26154 | 0.26154 | 0.26154 | 0.26036 | 0.26005 | 0.26005 | 0.16163 | 0.16163 | 0.16163 | 0.00000 | 0.00000 | 0.00569 | 0.00569 | | | | | | | | | | | |
| 25 | ***Metaradiophrya lumbrici* JA-2 26 LT** | 0.25029 | 0.23910 | 0.23910 | 0.23910 | 0.26163 | 0.26163 | 0.26163 | 0.25176 | 0.25176 | 0.25176 | 0.26154 | 0.26154 | 0.26154 | 0.26036 | 0.26005 | 0.26005 | 0.16163 | 0.16163 | 0.16163 | 0.00000 | 0.00000 | 0.00569 | 0.00569 | 0.00000 | | | | | | | | | | |
| 26 | ***Metaradiophrya speculorum* HkD 59 AT** | 0.24186 | 0.24592 | 0.24592 | 0.24592 | 0.25751 | 0.25635 | 0.25635 | 0.24102 | 0.24102 | 0.24102 | 0.24442 | 0.24442 | 0.24442 | 0.24324 | 0.23709 | 0.23709 | 0.18476 | 0.18476 | 0.18476 | 0.18677 | 0.18677 | 0.18677 | 0.18677 | 0.18677 | 0.18677 | | | | | | | | | |
| 27 | ***Metaradiophrya speculorum* HkD 60 AT** | 0.24186 | 0.24592 | 0.24592 | 0.24592 | 0.25751 | 0.25635 | 0.25635 | 0.24102 | 0.24102 | 0.24102 | 0.24442 | 0.24442 | 0.24442 | 0.24324 | 0.23709 | 0.23709 | 0.18476 | 0.18476 | 0.18476 | 0.18677 | 0.18677 | 0.18677 | 0.18677 | 0.18677 | 0.18677 | 0.00000 | | | | | | | | |
| 28 | ***Metaradiophrya varians* BZ 12 EA** | 0.25584 | 0.24590 | 0.24590 | 0.24590 | 0.25349 | 0.25349 | 0.25349 | 0.23166 | 0.23166 | 0.23166 | 0.24853 | 0.24853 | 0.24853 | 0.24735 | 0.24588 | 0.24588 | 0.17961 | 0.17961 | 0.17961 | 0.18765 | 0.18765 | 0.18648 | 0.18648 | 0.18765 | 0.18765 | 0.17321 | 0.17321 | | | | | | | |
| 29 | ***Metaradiophrya varians* BZ 14 EA** | 0.25584 | 0.24590 | 0.24590 | 0.24590 | 0.25349 | 0.25349 | 0.25349 | 0.23166 | 0.23166 | 0.23166 | 0.24853 | 0.24853 | 0.24853 | 0.24735 | 0.24588 | 0.24588 | 0.17961 | 0.17961 | 0.17961 | 0.18765 | 0.18765 | 0.18648 | 0.18648 | 0.18765 | 0.18765 | 0.17321 | 0.17321 | 0.00000 | | | | | | |
| 30 | ***Metaradiophrya varians* JA-1 19 EA** | 0.25584 | 0.24590 | 0.24590 | 0.24590 | 0.25349 | 0.25349 | 0.25349 | 0.23166 | 0.23166 | 0.23166 | 0.24853 | 0.24853 | 0.24853 | 0.24735 | 0.24588 | 0.24588 | 0.17961 | 0.17961 | 0.17961 | 0.18765 | 0.18765 | 0.18648 | 0.18648 | 0.18765 | 0.18765 | 0.17321 | 0.17321 | 0.00000 | 0.00000 | | | | | |
| 31 | ***Metaradiophrya varians* JA-1 22 EA** | 0.25584 | 0.24590 | 0.24590 | 0.24590 | 0.25349 | 0.25349 | 0.25349 | 0.23166 | 0.23166 | 0.23166 | 0.24853 | 0.24853 | 0.24853 | 0.24735 | 0.24588 | 0.24588 | 0.17961 | 0.17961 | 0.17961 | 0.18765 | 0.18765 | 0.18648 | 0.18648 | 0.18765 | 0.18765 | 0.17321 | 0.17321 | 0.00000 | 0.00000 | 0.00000 | | | | |
| 32 | ***Metaradiophrya varians* BZkv 31 EA** | 0.25584 | 0.24590 | 0.24590 | 0.24590 | 0.25349 | 0.25349 | 0.25349 | 0.23166 | 0.23166 | 0.23166 | 0.24853 | 0.24853 | 0.24853 | 0.24735 | 0.24588 | 0.24588 | 0.17961 | 0.17961 | 0.17961 | 0.18765 | 0.18765 | 0.18648 | 0.18648 | 0.18765 | 0.18765 | 0.17321 | 0.17321 | 0.00000 | 0.00000 | 0.00000 | 0.00000 | | | |
| 33 | ***Metaradiophrya varians* BZkv 32 EA** | 0.25584 | 0.24590 | 0.24590 | 0.24590 | 0.25349 | 0.25349 | 0.25349 | 0.23166 | 0.23166 | 0.23166 | 0.24853 | 0.24853 | 0.24853 | 0.24735 | 0.24588 | 0.24588 | 0.17961 | 0.17961 | 0.17961 | 0.18765 | 0.18765 | 0.18648 | 0.18648 | 0.18765 | 0.18765 | 0.17321 | 0.17321 | 0.00000 | 0.00000 | 0.00000 | 0.00000 | 0.00000 | | |
| 34 | ***Subanoplophrya nodulata* PU 29 OT** | 0.34559 | 0.33784 | 0.33784 | 0.33784 | 0.34751 | 0.34629 | 0.34629 | 0.34348 | 0.34348 | 0.34348 | 0.33787 | 0.33787 | 0.33787 | 0.33663 | 0.32880 | 0.32880 | 0.32274 | 0.32274 | 0.32274 | 0.32641 | 0.32641 | 0.32885 | 0.32885 | 0.32641 | 0.32641 | 0.34024 | 0.34024 | 0.33088 | 0.33088 | 0.33088 | 0.33088 | 0.33088 | 0.33088 | |
| 35 | ***Subanoplophrya nodulata* PU 30 OT** | 0.34559 | 0.33784 | 0.33784 | 0.33784 | 0.34751 | 0.34629 | 0.34629 | 0.34348 | 0.34348 | 0.34348 | 0.33787 | 0.33787 | 0.33787 | 0.33663 | 0.32880 | 0.32880 | 0.32274 | 0.32274 | 0.32274 | 0.32641 | 0.32641 | 0.32885 | 0.32885 | 0.32641 | 0.32641 | 0.34024 | 0.34024 | 0.33088 | 0.33088 | 0.33088 | 0.33088 | 0.33088 | 0.33088 | 0.00000 |

*Note: Specimen code consists of a locality code, an isolate code, and an abbreviation of host name. Locality codes are summarized in Supplementary Table 1.*

**SUPPLEMENTARY TABLE 9 |** Pairwise *p*-distances of COI gene sequences between astomes isolated from lumbricid earthworms.

| No. | Specimen | 1 | 2 | 3 | 4 | 5 | 6 | 7 | 8 | 9 | 10 | 11 | 12 | 13 | 14 | 15 | 16 | 17 | 18 | 19 | 20 | 21 | 22 | 23 | 24 | 25 | 26 | 27 | 28 | 29 |
|---|---|---|---|---|---|---|---|---|---|---|---|---|---|---|---|---|---|---|---|---|---|---|---|---|---|---|---|---|---|---|
| 1 | ***Anoplophrya allolobophorae* JA-3 37 ACH** | | | | | | | | | | | | | | | | | | | | | | | | | | | | | |
| 2 | ***Anoplophrya vulgaris* NG 27 DV** | 0.22778 | | | | | | | | | | | | | | | | | | | | | | | | | | | | |
| 3 | ***Anoplophrya vulgaris* NG 28 DV** | 0.22778 | 0.00000 | | | | | | | | | | | | | | | | | | | | | | | | | | | |
| 4 | ***Anoplophrya lumbrici* RZ 6 LT** | 0.24722 | 0.27917 | 0.27917 | | | | | | | | | | | | | | | | | | | | | | | | | | |
| 5 | ***Anoplophrya lumbrici* KR 9 LT** | 0.24722 | 0.27917 | 0.27917 | 0.00000 | | | | | | | | | | | | | | | | | | | | | | | | | |
| 6 | ***Anoplophrya lumbrici* KR 11 LT** | 0.24722 | 0.27917 | 0.27917 | 0.00000 | 0.00000 | | | | | | | | | | | | | | | | | | | | | | | | |
| 7 | ***Anoplophrya octolasionis* MU 56 OL** | 0.24583 | 0.24861 | 0.24861 | 0.29028 | 0.29028 | 0.29028 | | | | | | | | | | | | | | | | | | | | | | | |
| 8 | ***Anoplophrya octolasionis* MU 57 OL** | 0.24583 | 0.24861 | 0.24861 | 0.29028 | 0.29028 | 0.29028 | 0.00000 | | | | | | | | | | | | | | | | | | | | | | |
| 9 | ***Anoplophrya octolasionis* MU 58 OL** | 0.24583 | 0.24861 | 0.24861 | 0.29028 | 0.29028 | 0.29028 | 0.00000 | 0.00000 | | | | | | | | | | | | | | | | | | | | | |
| 10 | ***Maupasella mucronata* KDo 33 ET** | 0.36652 | 0.37229 | 0.37229 | 0.37229 | 0.37229 | 0.37229 | 0.35498 | 0.35498 | 0.35498 | | | | | | | | | | | | | | | | | | | | |
| 11 | ***Maupasella mucronata* KDo 34 ET** | 0.36652 | 0.37229 | 0.37229 | 0.37229 | 0.37229 | 0.37229 | 0.35498 | 0.35498 | 0.35498 | 0.00000 | | | | | | | | | | | | | | | | | | | |
| 12 | ***Maupasella mucronata* KDo 35 ET** | 0.36652 | 0.37229 | 0.37229 | 0.37229 | 0.37229 | 0.37229 | 0.35498 | 0.35498 | 0.35498 | 0.00000 | 0.00000 | | | | | | | | | | | | | | | | | | |
| 13 | ***Maupasella mucronata* KDo 36 ET** | 0.36652 | 0.37229 | 0.37229 | 0.37229 | 0.37229 | 0.37229 | 0.35498 | 0.35498 | 0.35498 | 0.00000 | 0.00000 | 0.00000 | | | | | | | | | | | | | | | | | |
| 14 | ***Metaradiophrya chlorotica* JA-2 1M ACH** | 0.24861 | 0.26806 | 0.26806 | 0.30000 | 0.30000 | 0.30000 | 0.26111 | 0.26111 | 0.26111 | 0.37085 | 0.37085 | 0.37085 | 0.37085 | | | | | | | | | | | | | | | | |
| 15 | ***Metaradiophrya chlorotica* JA-2 2M ACH** | 0.24861 | 0.26806 | 0.26806 | 0.30000 | 0.30000 | 0.30000 | 0.26111 | 0.26111 | 0.26111 | 0.37085 | 0.37085 | 0.37085 | 0.37085 | 0.00000 | | | | | | | | | | | | | | | |
| 16 | ***Metaradiophrya chlorotica* JA-2 3M ACH** | 0.24861 | 0.26806 | 0.26806 | 0.30000 | 0.30000 | 0.30000 | 0.26111 | 0.26111 | 0.26111 | 0.37085 | 0.37085 | 0.37085 | 0.37085 | 0.00000 | 0.00000 | | | | | | | | | | | | | | |
| 17 | ***Metaradiophrya lumbrici* RZ 4 LT** | 0.25972 | 0.26111 | 0.26111 | 0.28611 | 0.28611 | 0.28611 | 0.26111 | 0.26111 | 0.26111 | 0.36219 | 0.36219 | 0.36219 | 0.36219 | 0.18750 | 0.18750 | 0.18750 | | | | | | | | | | | | | |
| 18 | ***Metaradiophrya lumbrici* RZ 5 LT** | 0.25833 | 0.26111 | 0.26111 | 0.28472 | 0.28472 | 0.28472 | 0.26111 | 0.26111 | 0.26111 | 0.36219 | 0.36219 | 0.36219 | 0.36219 | 0.18611 | 0.18611 | 0.18611 | 0.00417 | | | | | | | | | | | | |
| 19 | ***Metaradiophrya lumbrici* KR 8 LT** | 0.25833 | 0.25972 | 0.25972 | 0.28333 | 0.28333 | 0.28333 | 0.25972 | 0.25972 | 0.25972 | 0.36219 | 0.36219 | 0.36219 | 0.36219 | 0.18472 | 0.18472 | 0.18472 | 0.00833 | 0.00972 | | | | | | | | | | | |
| 20 | ***Metaradiophrya lumbrici* KR 10 LT** | 0.26111 | 0.26111 | 0.26111 | 0.28333 | 0.28333 | 0.28333 | 0.26111 | 0.26111 | 0.26111 | 0.36219 | 0.36219 | 0.36219 | 0.36219 | 0.18611 | 0.18611 | 0.18611 | 0.00833 | 0.00972 | 0.00417 | | | | | | | | | | |
| 21 | ***Metaradiophrya lumbrici* JA-2 25 LT** | 0.25833 | 0.25972 | 0.25972 | 0.28611 | 0.28611 | 0.28611 | 0.25972 | 0.25972 | 0.25972 | 0.36508 | 0.36508 | 0.36508 | 0.36508 | 0.18889 | 0.18889 | 0.18889 | 0.00417 | 0.00278 | 0.00833 | 0.01111 | | | | | | | | | |
| 22 | ***Metaradiophrya lumbrici* JA-2 26 LT** | 0.25833 | 0.26111 | 0.26111 | 0.28611 | 0.28611 | 0.28611 | 0.26111 | 0.26111 | 0.26111 | 0.36364 | 0.36364 | 0.36364 | 0.36364 | 0.18750 | 0.18750 | 0.18750 | 0.00278 | 0.00139 | 0.00833 | 0.01111 | 0.00139 | | | | | | | | |
| 23 | ***Metaradiophrya speculorum* HkD 59 AT** | 0.26944 | 0.27500 | 0.27500 | 0.29722 | 0.29722 | 0.29722 | 0.25972 | 0.25972 | 0.25972 | 0.35786 | 0.35786 | 0.35786 | 0.35786 | 0.23333 | 0.23333 | 0.23333 | 0.23194 | 0.23194 | 0.23056 | 0.23194 | 0.23194 | 0.23194 | | | | | | | |
| 24 | ***Metaradiophrya speculorum* HkD 60 AT** | 0.26944 | 0.27500 | 0.27500 | 0.29722 | 0.29722 | 0.29722 | 0.25972 | 0.25972 | 0.25972 | 0.35786 | 0.35786 | 0.35786 | 0.35786 | 0.23333 | 0.23333 | 0.23333 | 0.23194 | 0.23194 | 0.23056 | 0.23194 | 0.23194 | 0.23194 | 0.00000 | | | | | | |
| 25 | ***Metaradiophrya varians* BZ 12 EA** | 0.26331 | 0.25770 | 0.25770 | 0.30252 | 0.30252 | 0.30252 | 0.26331 | 0.26331 | 0.26331 | 0.37229 | 0.37229 | 0.37229 | 0.37229 | 0.20448 | 0.20448 | 0.20448 | 0.22129 | 0.22269 | 0.22129 | 0.22129 | 0.22409 | 0.22269 | 0.26751 | 0.26751 | | | | | |
| 26 | ***Metaradiophrya varians* BZ 14 EA** | 0.26471 | 0.26050 | 0.26050 | 0.30392 | 0.30392 | 0.30392 | 0.26190 | 0.26190 | 0.26190 | 0.37229 | 0.37229 | 0.37229 | 0.37229 | 0.20588 | 0.20588 | 0.20588 | 0.22129 | 0.22269 | 0.21989 | 0.21989 | 0.22409 | 0.22269 | 0.26891 | 0.26891 | 0.00280 | | | | |
| 27 | ***Metaradiophrya varians* JA-1 19 EA** | 0.26331 | 0.25770 | 0.25770 | 0.30252 | 0.30252 | 0.30252 | 0.26331 | 0.26331 | 0.26331 | 0.37229 | 0.37229 | 0.37229 | 0.37229 | 0.20448 | 0.20448 | 0.20448 | 0.22129 | 0.22269 | 0.22129 | 0.22129 | 0.22409 | 0.22269 | 0.26751 | 0.26751 | 0.00000 | 0.00280 | | | |
| 28 | ***Metaradiophrya varians* JA-1 22 EA** | 0.26331 | 0.25770 | 0.25770 | 0.30252 | 0.30252 | 0.30252 | 0.26331 | 0.26331 | 0.26331 | 0.37229 | 0.37229 | 0.37229 | 0.37229 | 0.20448 | 0.20448 | 0.20448 | 0.22129 | 0.22269 | 0.22129 | 0.22129 | 0.22409 | 0.22269 | 0.26751 | 0.26751 | 0.00000 | 0.00280 | 0.00000 | | |
| 29 | ***Metaradiophrya varians* BZkv 31 EA** | 0.26331 | 0.25770 | 0.25770 | 0.30252 | 0.30252 | 0.30252 | 0.26331 | 0.26331 | 0.26331 | 0.37229 | 0.37229 | 0.37229 | 0.37229 | 0.20448 | 0.20448 | 0.20448 | 0.22129 | 0.22269 | 0.22129 | 0.22129 | 0.22409 | 0.22269 | 0.26751 | 0.26751 | 0.00000 | 0.00280 | 0.00000 | 0.00000 | |
| 30 | ***Metaradiophrya varians* BZkv 32 EA** | 0.26331 | 0.25770 | 0.25770 | 0.30252 | 0.30252 | 0.30252 | 0.26331 | 0.26331 | 0.26331 | 0.37229 | 0.37229 | 0.37229 | 0.37229 | 0.20448 | 0.20448 | 0.20448 | 0.22129 | 0.22269 | 0.22129 | 0.22129 | 0.22409 | 0.22269 | 0.26751 | 0.26751 | 0.00000 | 0.00280 | 0.00000 | 0.00000 | 0.00000 |

*Note: Specimen code consists of a locality code, an isolate code, and an abbreviation of host name. Locality codes are summarized in Supplementary Table 1.*

**SUPPLEMENTARY TABLE 10 |** Occurrence of eleven astome ciliates in sixteen earthworm species.

| Locality no. | Locality code | Host species | Endosymbiont species: *Subanoplophrya nodulata* | *Maupasella mucronata* | *Metaradiophrya chlorotica* | *Metaradiophrya lumbrici* | *Metaradiophrya speculorum* | *Metaradiophrya varians* | *Anoplophrya allolobophorae* | *Anoplophrya aporrectodeae* | *Anoplophrya lumbrici* | *Anoplophrya octolasioni* | *Anoplophrya vulgaris* |
|---|---|---|---|---|---|---|---|---|---|---|---|---|---|
| **Earthworms from decomposing plant material** | | | | | | | | | | | | | |
| 1 | BZ | *Eisenia andrei* | | | | | | + | | | | | |
| 2 | BZ | *Eisenia andrei* | | | | | | + | | | | | + |
| 3 | JA-1 | *Eisenia andrei* | | | | | | + | | | | | + |
| 4 | NG | *Dendrobaena veneta* | | | | | | | | | | | + |
| 5 | PUh | *Lumbricus rubellus* | | | | | | | | | | | |
| **Earthworms from floodplain and waterlogged soils** | | | | | | | | | | | | | |
| 6 | KR | *Lumbricus terrestris* | | | | + | | | | | + | | |
| 7 | AMc | *Aporrectodea tuberculata* | | | | | | | | | | | |
| 8 | HkD | *Aporrectodea rosea* | | | | | | | | | | | |
| 8 | HkD | *Aporrectodea tuberculata* | | | | | + | | | | | | |
| 9 | LS | *Aporrectodea tuberculata* | | | | | | | | | | | |
| | | *Octolasion lacteum* | | | | | | | | | | | |
| 10 | KDo | *Dendrobaena octaedra* | | | | | | | | | | | |
| | | *Eiseniella tetraedra* | | + | | | | | | | | | |
| 11 | KDo | *Lumbricus terrestris* | | | | | | | | | | | |
| 12 | CBk | *Bimastos rubidus* | | | | | | | | | | | |
| | | *Eiseniella tetraedra* | | | | | | | | | | | |
| | | *Octolasion* sp. | | | | | | | | | | | |
| 13 | MB | *Fitzingeria platyura* | | | | | | | | | | | |
| 14 | PU | *Octolasion tyrtaeum* | + | | | | | | | | | | |
| **Earthworms from agricultural and grassland soils** | | | | | | | | | | | | | |
| 15 | RZ | *Lumbricus terrestris* | | | | + | | | | | + | | |
| 16 | JA-2 | *Allolobophora chlorotica* | | | + | | | | | | | | |
| 17 | FNS | *Lumbricus terrestris* | | | | | | | | | | | |
| 18 | PUz | *Aporrectodea tuberculata* | | | | | | | | + | | | |
| 19 | PUz | *Allolobophora chlorotica* | | | | | | | | | | | |
| | | *Aporrectodea trapezoides* | | | | | | | | | | | |
| | | *Aporrectodea tuberculata* | | | | | | | | + | | | |
| | | *Lumbricus terrestris* | | | | | | | | | | | |
| 20 | PUp | *Aporrectodea tuberculata* | | | | | | | | | | | |
| **Earthworms from garden soils** | | | | | | | | | | | | | |
| 21 | JA-2 | *Lumbricus terrestris* | | | | + | | | | | | | |
| 22 | JA-3 | *Allolobophora chlorotica* | | | | | | | + | | | | |
| | | *Aporrectodea trapezoides* | | | | | | | | | | | |
| 23 | HO | *Lumbricus terrestris* | | | | | | | | | | | |
| 24 | MU | *Octolasion lacteovicinum* | | | | | | | | | | + | |
| 25 | BZ | *Lumbricus terrestris* | | | | | | | | | | | |

*Presence is designated by plus (+). Colors denote the individual ecological group of earthworms (yellow = epigeic, green = anecic, and blue = endogeic).*
*For locality codes, see Supplementary Table 1.*

**SUPPLEMENTARY TABLE 11 |** Characterization and origin of the mitochondrial ND1 gene sequences of earthworms analyzed in the present study.

| Species | Speci-men | Locality no. | Locality | GenBank no. |
|---|---|---|---|---|
| *Allolobophora chlorotica* | JA-2 6 | 16 | Garden, Jakubská ulica, Rača, Bratislava | MZ056758 |
| *Allolobophora chlorotica* | JA-3 8 | 22 | Garden, Jakubská ulica, Rača, Bratislava | MZ056759 |
| *Allolobophora chlorotica* | PUz 16 | 19 | Garden, Spodná ulica, Pusté Úľany village | MZ056760 |
| *Aporrectodea rosea* | HkD 22 | 8 | Foot of the cliff of the Devín Castle, Bratislava | MZ056761 |
| *Aporrectodea trapezoides* | JA-3 7 | 22 | Garden, Jakubská ulica, Rača, Bratislava | MZ056762 |
| *Aporrectodea trapezoides* | PUz 9 | 19 | Garden, Spodná ulica, Pusté Úľany village | MZ056763 |
| *Aporrectodea tuberculata* | AMc 19 | 7 | Willow-poplar forest near the Danube river, Devín, Bratislava | MZ056764 |
| *Aporrectodea tuberculata* | AMc 20 | | | MZ056765 |
| *Aporrectodea tuberculata* | HkD 21 | 8 | Foot of the cliff of the Devín Castle, Bratislava | MZ056766 |
| *Aporrectodea tuberculata* | HkD 26 | | | MZ056767 |
| *Aporrectodea tuberculata* | LS 17 | 9 | Meadow near the Danube river, Devín, Bratislava | MZ056768 |
| *Aporrectodea tuberculata* | PUz 2 | 19 | Garden, Spodná ulica, Pusté Úľany village | MZ056769 |
| *Aporrectodea tuberculata* | PUz 11 | | | MZ056770 |
| *Aporrectodea tuberculata* | PUp 29 | 20 | Field in the vicinity of the Pusté Úľany village | MZ056771 |
| *Dendrodrilus rubidus* | CBk 13 | 12 | Oak-hornbeam forest, Bratislava, Malé Karpaty Mts. | MZ056772 |
| *Dendrobaena octaedra* | KDo 5 | 10 | Oak-hornbeam forest, Knižková dolina valley, Bratislava, Malé Karpaty Mts | MZ056773 |
| *Eisenia andrei* | BZ 1 | 1 | Botanical Garden, Karlova Ves, Bratislava | MZ056774 |
| *Eisenia andrei* | JA-1 | 3 | Jakubská ulica, Rača, Bratislava | MZ056775 |
| *Eiseniella tetraedra* | CBk 14 | 12 | Oak-hornbeam forest, Bratislava, Malé Karpaty Mts. | MZ056776 |
| *Eiseniella tetraedra* | KDo 3 | 10 | Oak-hornbeam forest, Knižková dolina valley, Bratislava, Malé Karpaty Mts. | MZ056777 |
| *Eiseniella tetraedra* | KDo 4 | | | MZ056778 |
| *Fitzingeria platyura* | MB 27 | 13 | Oak-hornbeam forest, Malá Baňa, Bratislava, Malé Karpaty Mts. | MZ056779 |
| *Lumbricus rubellus* | PUh 30 | 5 | Poplar forest in the vicinity of the Pusté Úľany village | MZ056780 |
| *Lumbricus rubellus* | PUh 31 | | | MZ056781 |
| *Lumbricus terrestris* | BZ | 25 | Botanical Garden, Karlova Ves, Bratislava | MZ056782 |
| *Lumbricus terrestris* | FNS 32 | 17 | Grassland, Comenius University, Bratislava | MZ056783 |
| *Lumbricus terrestris* | HO 28 | 23 | Garden, Horská ulica, Nové mesto, Bratislava | MZ056784 |
| *Lumbricus terrestris* | JA-2 | 21 | Garden wall, Jakubská ulica, Rača, Bratislava | MZ056785 |
| *Lumbricus terrestris* | KDo 15 | | Oak-hornbeam forest, Knižková dolina valley, Bratislava, Malé Karpaty Mts. | MZ056786 |
| *Lumbricus terrestris* | KR | 6 | Willow-poplar forest, Karlova Ves, Bratislava | MZ056787 |
| *Lumbricus terrestris* | RZ | 15 | Garden, Šúrska ulica, Rendez, Bratislava | MZ056788 |
| *Lumbricus terrestris* | PUz 10 | 19 | Garden, Spodná ulica, Pusté Úľany village | MZ056789 |
| *Octolasion lacteovicinum* | MU 23 | 24 | Garden, Moskovská ulica, Staré mesto, Bratislava | MZ056790 |
| *Octolasion lacteovicinum* | MU 24 | | | MZ056791 |
| *Octolasion lacteovicinum* | MU 25 | | | MZ056792 |
| *Octolasion lacteum* | LS 18 | 9 | Meadow near the Danube river, Devín, Bratislava | MZ056793 |
| *Octolasion* sp. | CBk 12 | 12 | Oak-hornbeam forest, Bratislava, Malé Karpaty Mts. | MZ056794 |

*For locality codes and further details, see Supplementary Table 1.*

**SUPPLEMENTARY TABLE 12 |** Characterization and origin of the mitochondrial COI gene sequences of earthworms analyzed in the present study.

| Species | Speci-men | Locality no. | Locality | GenBank no. |
|---|---|---|---|---|
| *Allolobophora chlorotica* | JA-2 6 | 16 | Garden, Jakubská ulica, Rača, Bratislava | MZ044869 |
| *Allolobophora chlorotica* | JA-3 8 | 22 | Garden, Jakubská ulica, Rača, Bratislava | MZ044870 |
| *Allolobophora chlorotica* | PUz 16 | 19 | Garden, Spodná ulica, Pusté Úľany village | MZ044871 |
| *Aporrectodea rosea* | HkD 22 | 8 | Foot of the cliff of the Devín Castle, Bratislava | MZ044872 |
| *Aporrectodea trapezoides* | JA-3 7 | 22 | Garden, Jakubská ulica, Rača, Bratislava | MZ044873 |
| *Aporrectodea trapezoides* | PUz 9 | 19 | Garden, Spodná ulica, Pusté Úľany village | MZ044874 |
| *Aporrectodea tuberculata* | LS 17 | 9 | Meadow near the Danube river, Devín, Bratislava | MZ044875 |
| *Dendrodrilus rubidus* | CBk 13 | 12 | Oak-hornbeam forest, Bratislava, Malé Karpaty Mts. | MZ044876 |
| *Eisenia andrei* | BZ 1 | 1 | Botanical Garden, Karlova Ves, Bratislava | MZ044877 |
| *Eisenia andrei* | JA-1 | 3 | Jakubská ulica, Rača, Bratislava | MZ044878 |
| *Eiseniella tetraedra* | CBk 14 | 12 | Oak-hornbeam forest, Bratislava, Malé Karpaty Mts. | MZ044879 |
| *Eiseniella tetraedra* | KDo 3 | 10 | Oak-hornbeam forest, Knižková dolina valley, | MZ044880 |
| *Eiseniella tetraedra* | KDo 4 | | Bratislava, Malé Karpaty Mts. | MZ044881 |
| *Fitzingeria platyura* | MB 27 | 13 | Oak-hornbeam forest, Malá Baňa, Bratislava, Malé Karpaty Mts. | MZ044882 |
| *Lumbricus rubellus* | PUh 30 | 5 | Poplar forest in the vicinity of the Pusté Úľany village | MZ044883 |
| *Lumbricus rubellus* | PUh 31 | | | MZ044884 |
| *Lumbricus terrestris* | BZ | 25 | Botanical Garden, Karlova Ves, Bratislava | MZ044885 |
| *Lumbricus terrestris* | FNS 32 | 17 | Grassland, Comenius University, Bratislava | MZ044886 |
| *Lumbricus terrestris* | HO 28 | 23 | Garden, Horská ulica, Nové mesto, Bratislava | MZ044887 |
| *Lumbricus terrestris* | JA-2 | 21 | Garden wall, Jakubská ulica, Rača, Bratislava | MZ044888 |
| *Lumbricus terrestris* | KDo 15 | | Oak-hornbeam forest, Knižková dolina valley, Bratislava, Malé Karpaty Mts. | MZ044889 |
| *Lumbricus terrestris* | KR | 6 | Willow-poplar forest, Karlova Ves, Bratislava | MZ044890 |
| *Lumbricus terrestris* | RZ | 15 | Garden, Šúrska ulica, Rendez, Bratislava | MZ044891 |
| *Lumbricus terrestris* | PUz 10 | 19 | Garden, Spodná ulica, Pusté Úľany village | MZ044892 |
| *Octolasion lacteovicinum* | MU 23 | 24 | Garden, Moskovská ulica, Staré mesto, Bratislava | MZ044893 |
| *Octolasion lacteovicinum* | MU 24 | | | MZ044894 |
| *Octolasion lacteovicinum* | MU 25 | | | MZ044895 |

*For locality codes and further details, see Supplementary Table 1.*

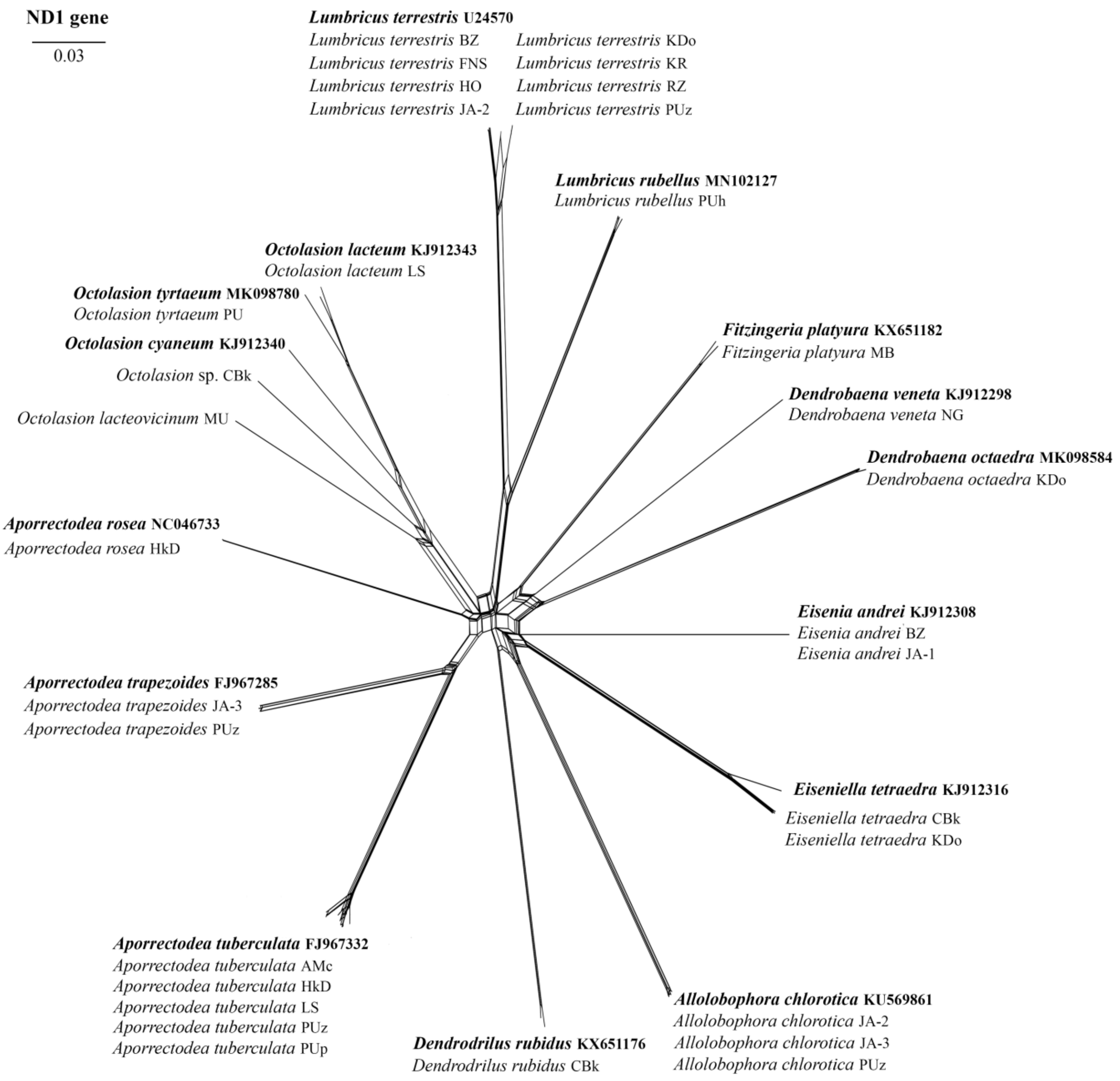


**SUPPLEMENTARY FIGURE 1 |** Phylogenetic network computed from NADH-ubiquinone oxidoreductase chain 1 (ND1) sequences of all studied lumbricid earthworms, using the neighbornet algorithm and the uncorrected distances in SplitsTree ver. 4. Sequences in bold face were downloaded from GenBank and served for the classification of the earthworm species studied. For GenBank accession numbers of newly obtained sequences, see Supplementary Table 11. The scale bar indicates three substitutions per one hundred nucleotide positions.

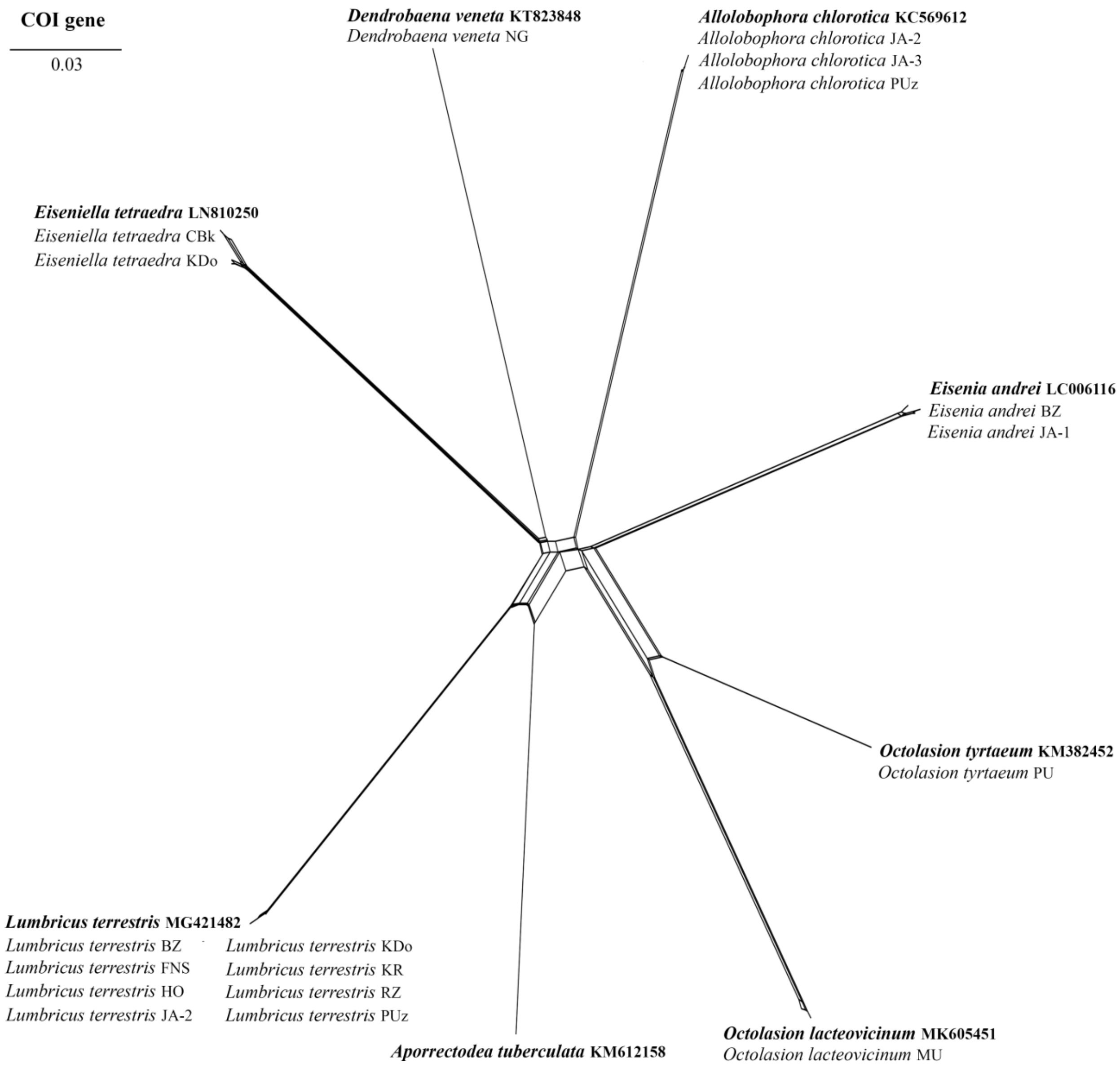


**SUPPLEMENTARY FIGURE 2 |** Phylogenetic network computed from cytochrome oxidase *c* subunit I (COI) sequences of lumbricid earthworms carrying astome ciliates with the neighbornet algorithm and the uncorrected distances in SplitsTree ver. 4. Sequences in bold face were downloaded from GenBank and served for the classification of the earthworm species carrying astome ciliates. For GenBank accession numbers of newly obtained sequences, see Supplementary Table 12. The scale bar indicates three substitutions per one hundred nucleotide positions.

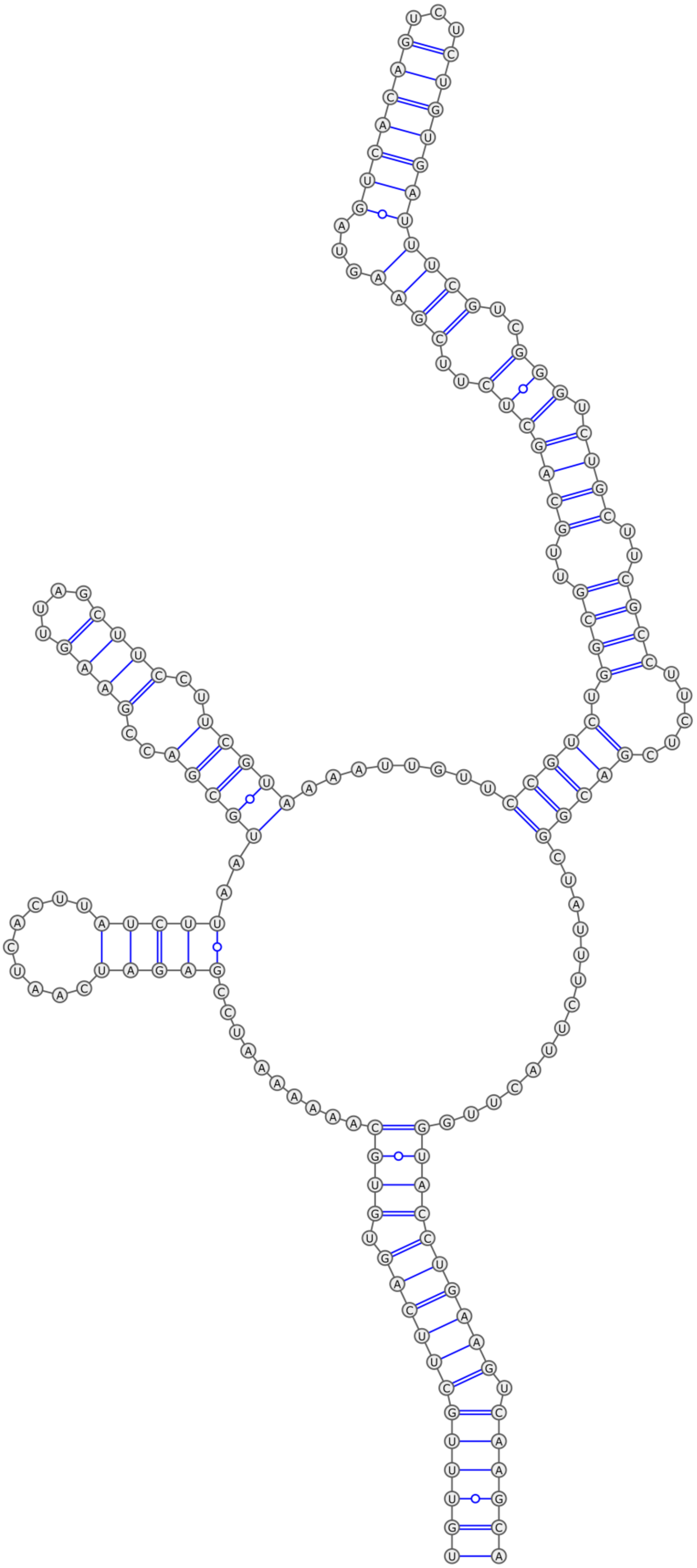

**SUPPLEMENTARY FIGURE 3 |** Putative secondary structure of the ITS2 molecule of *Almophrya bivacuolata* (HQ446281, host earthworms: *Alma emini / Alma nilotica*).

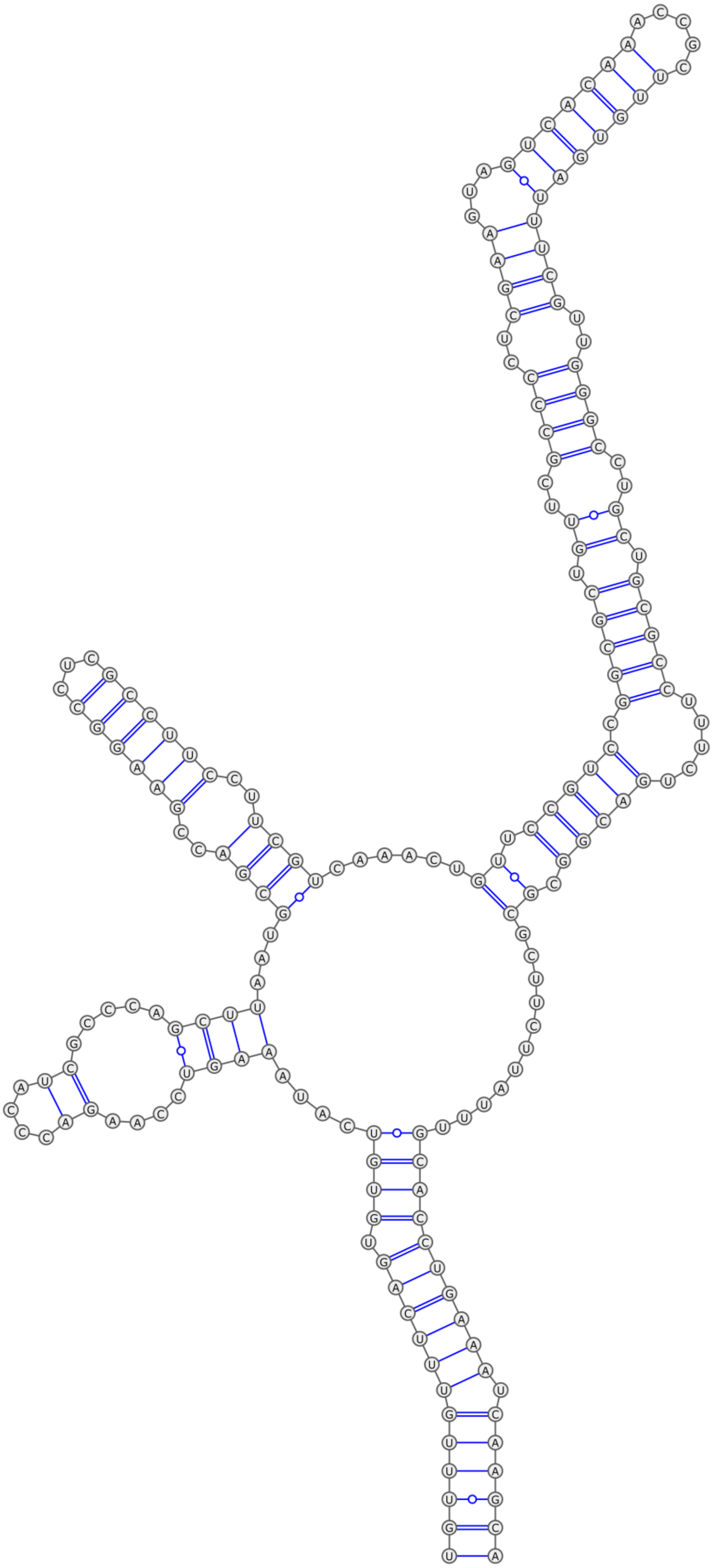

**SUPPLEMENTARY FIGURE 4 |** Putative secondary structure of the ITS2 molecule of *Anoplophrya aporrectodeae* (samples PUz 17 AT, PUz 40 AT, and PUz 41 AT, host earthworm: *Aporrectodea tuberculata*).

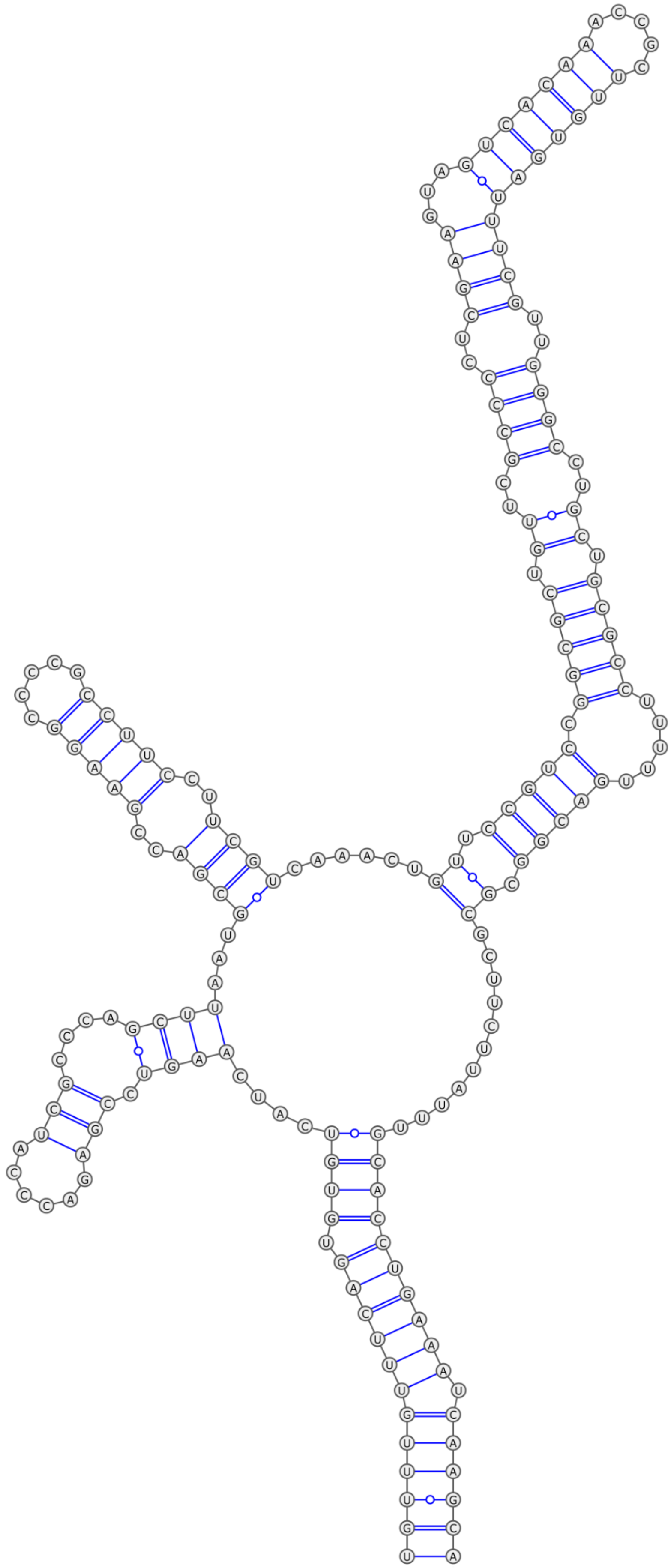

**SUPPLEMENTARY FIGURE 5 |** Putative secondary structure of the ITS2 molecule of *Anoplophrya allolobophorae* (sample JA-3 37 ACH, host earthworm: *Allolobophora chlorotica*).

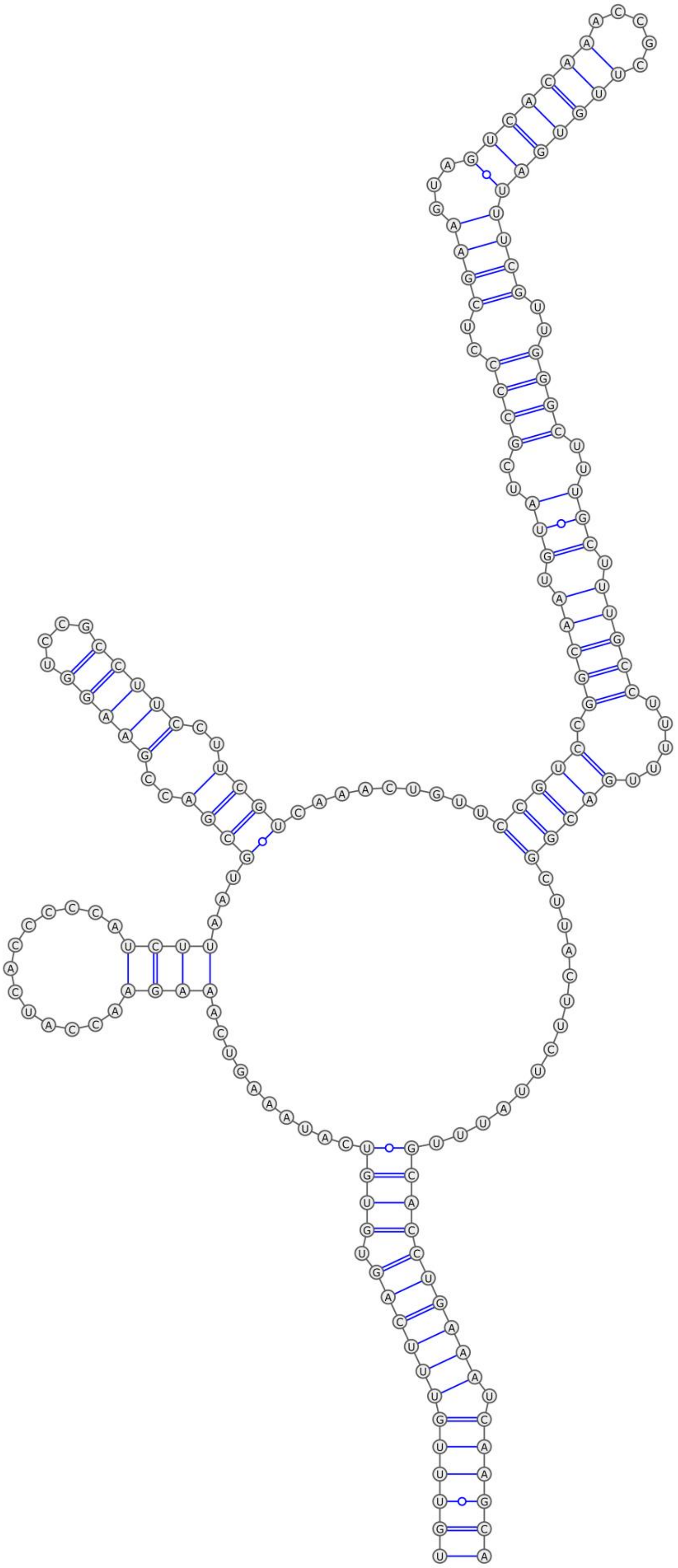

**SUPPLEMENTARY FIGURE 6 |** Putative secondary structure of the ITS2 molecule of *Anoplophrya lumbrici* (samples RZ 6 LT, KR 9 LT, and KR 11 LT, host earthworm: *Lumbricus terrestris*).

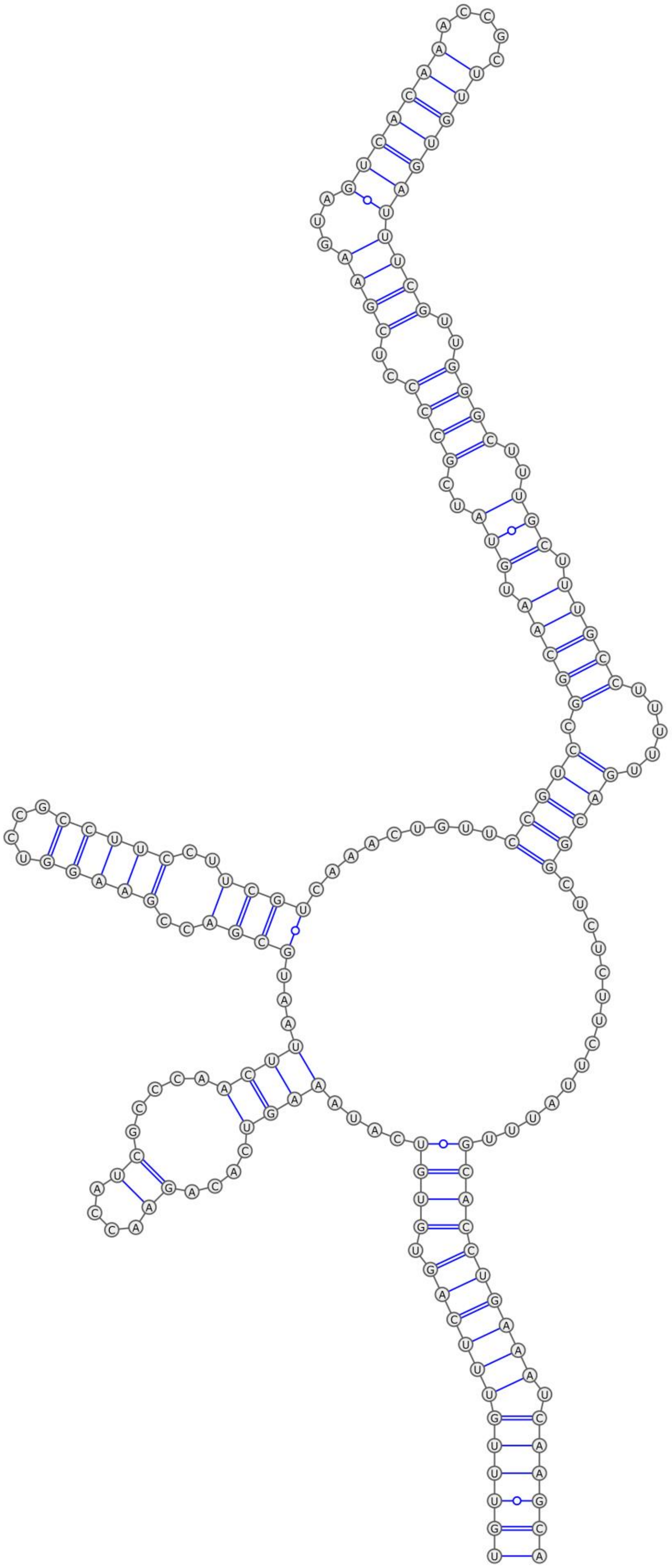

**SUPPLEMENTARY FIGURE 7 |** Putative secondary structure of the ITS2 molecule of *Anoplophrya octolasionis* (samples MU 56 OL, MU 57 OL, and MU 58 OL, host earthworm: *Octolasion lacteovicinum*).

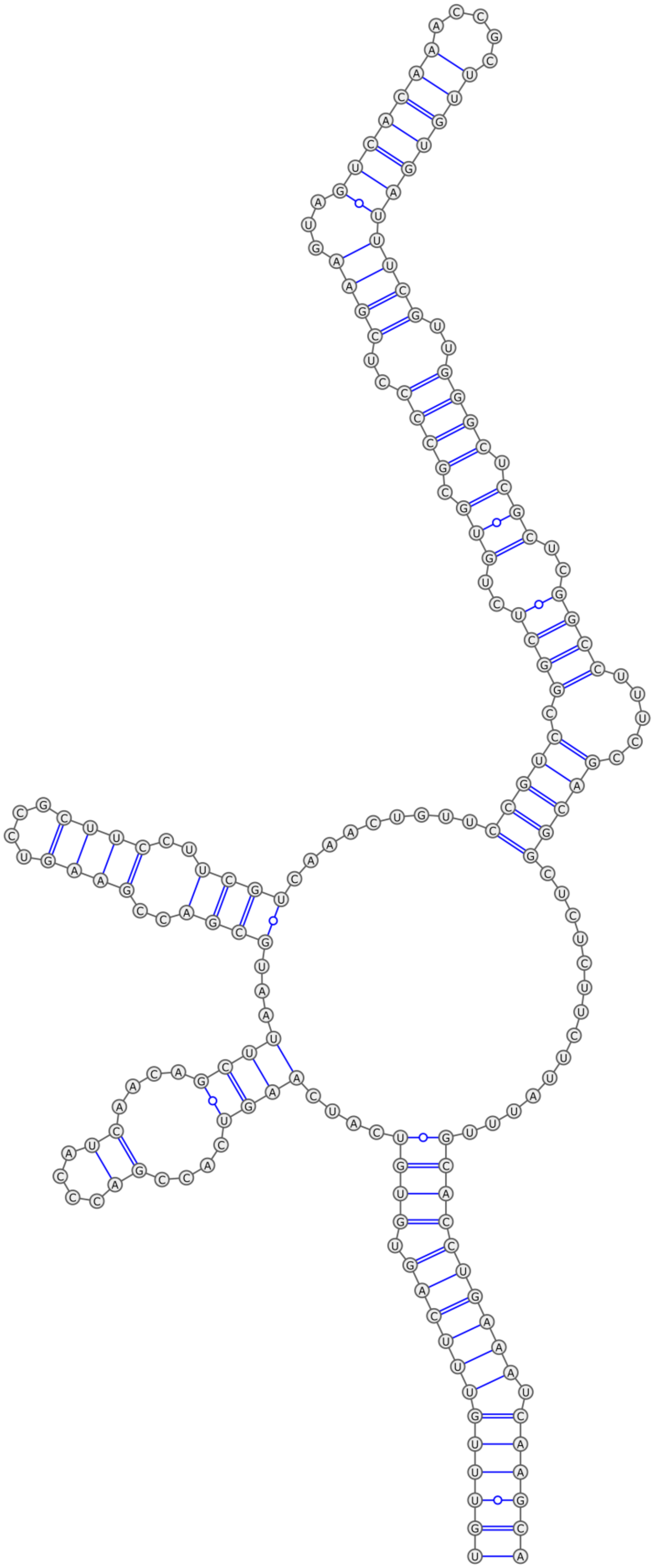

**SUPPLEMENTARY FIGURE 8 |** Putative secondary structure of the ITS2 molecule of *Anoplophrya vulgaris* (samples BZ 13 EF, JA-1 18 EF, JA-1 20 EF, JA-1 21 EF, NG 27 DV, and NG 28 DV, host earthworms: *Eisenia fetida* complex and *Dendrobaena veneta*).

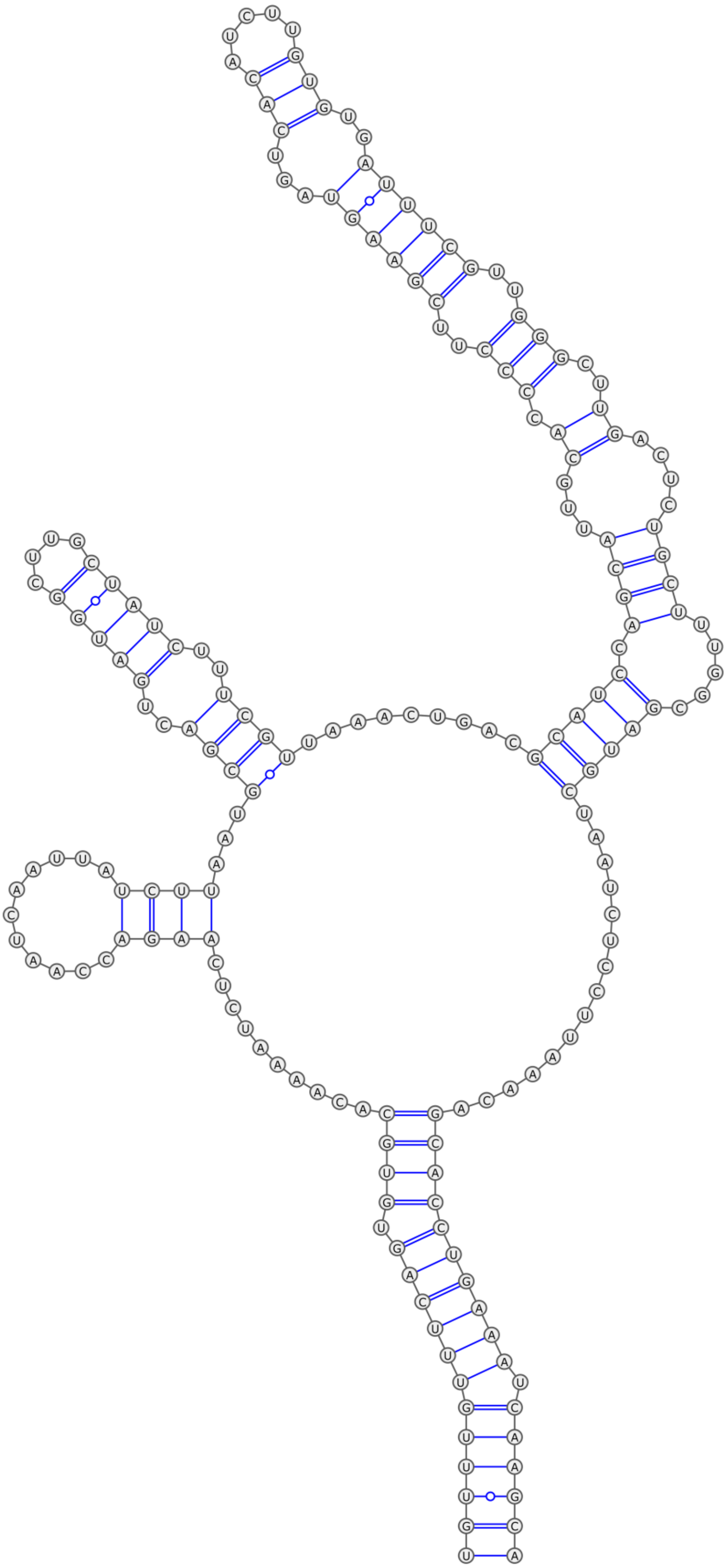

**SUPPLEMENTARY FIGURE 9 |** Putative secondary structure of the ITS2 molecule of *Eudrilophrya complanata* (HQ446280, host earthworm: *Eupolytoreutus* sp.).

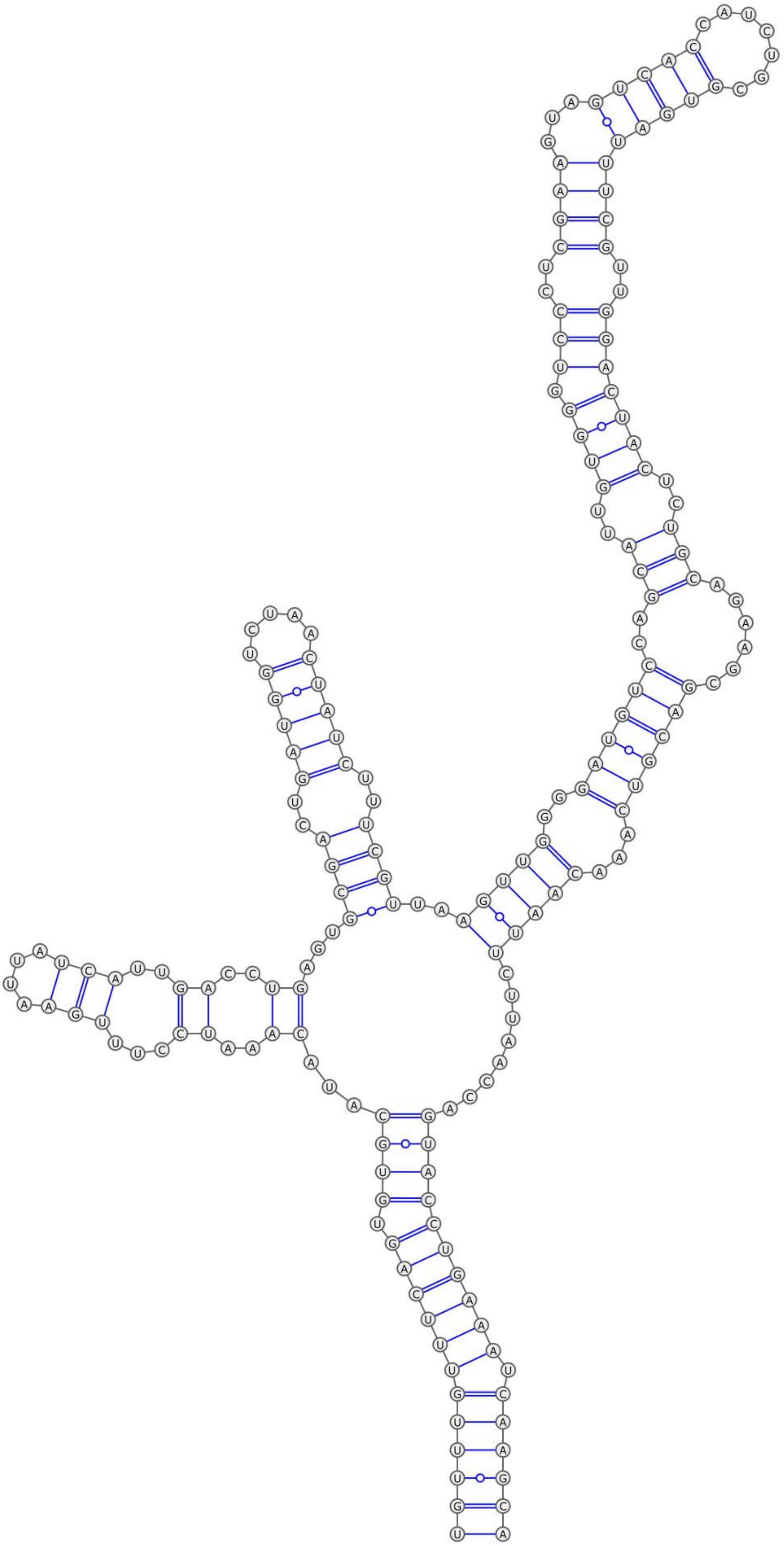

**SUPPLEMENTARY FIGURE 10 |** Putative secondary structure of the ITS2 molecule of *Maupasella mucronata* (samples KDo 33 ET, KDo 34 ET, KDo 35 ET, and KDo 36 ET, host earthworm: *Eiseniella tetraedra*).

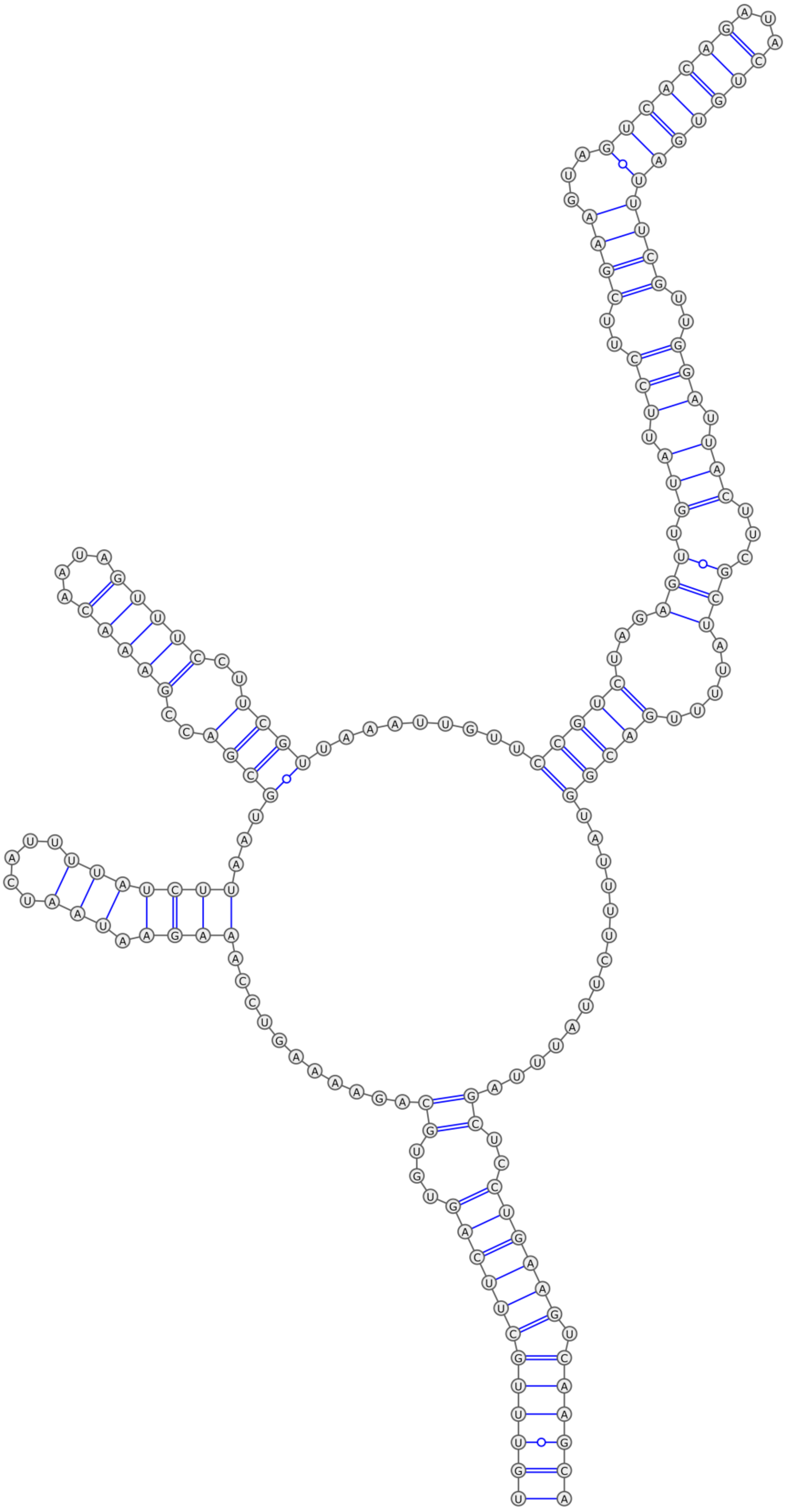

**SUPPLEMENTARY FIGURE 11 |** Putative secondary structure of the ITS2 molecule of *Metaracoelophrya* sp. 1 (HQ446277, host earthworms: *Alma emini / Alma nilotica*).

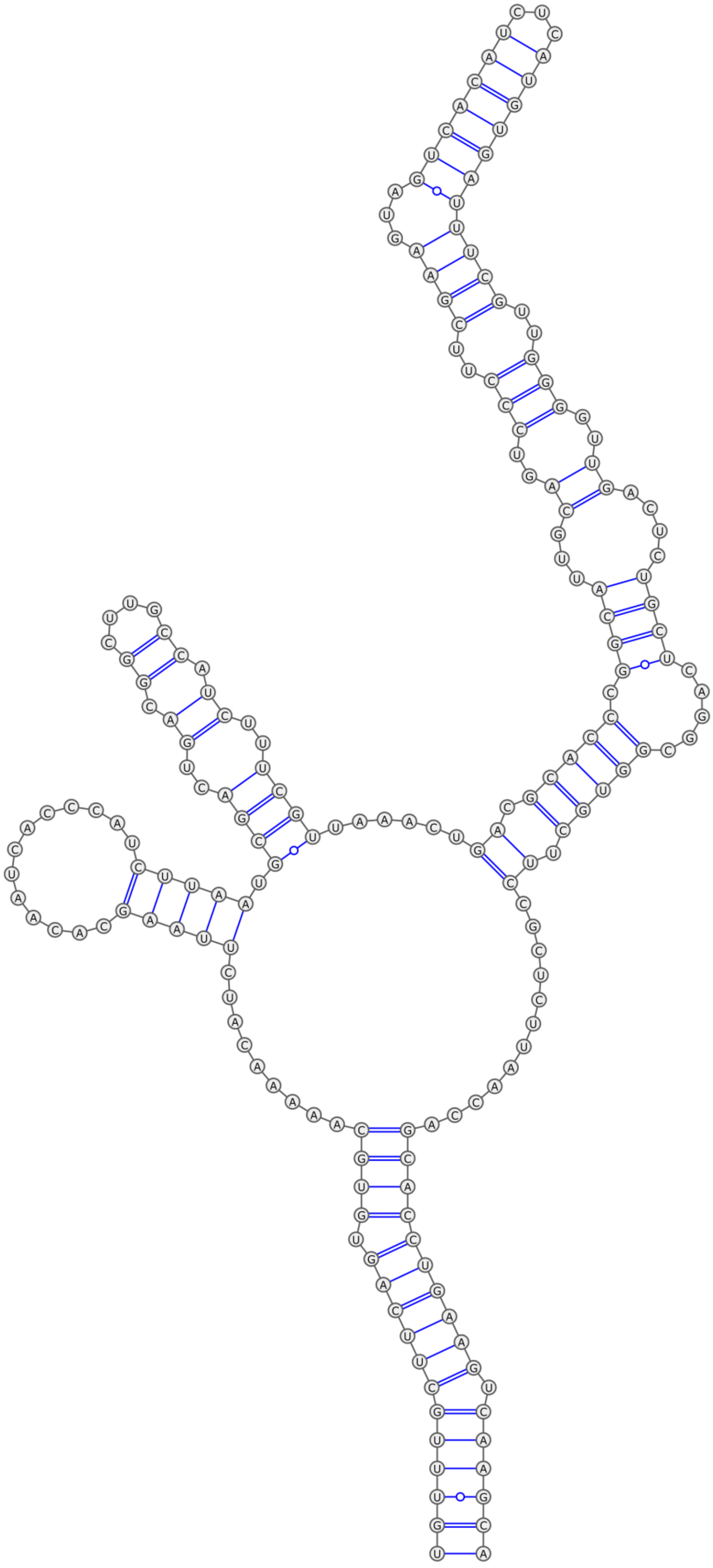

**SUPPLEMENTARY FIGURE 12 |** Putative secondary structure of the ITS2 molecule of *Njinella prolifera* (HQ446276, host earthworm: *Eupolytoreutus* sp.).

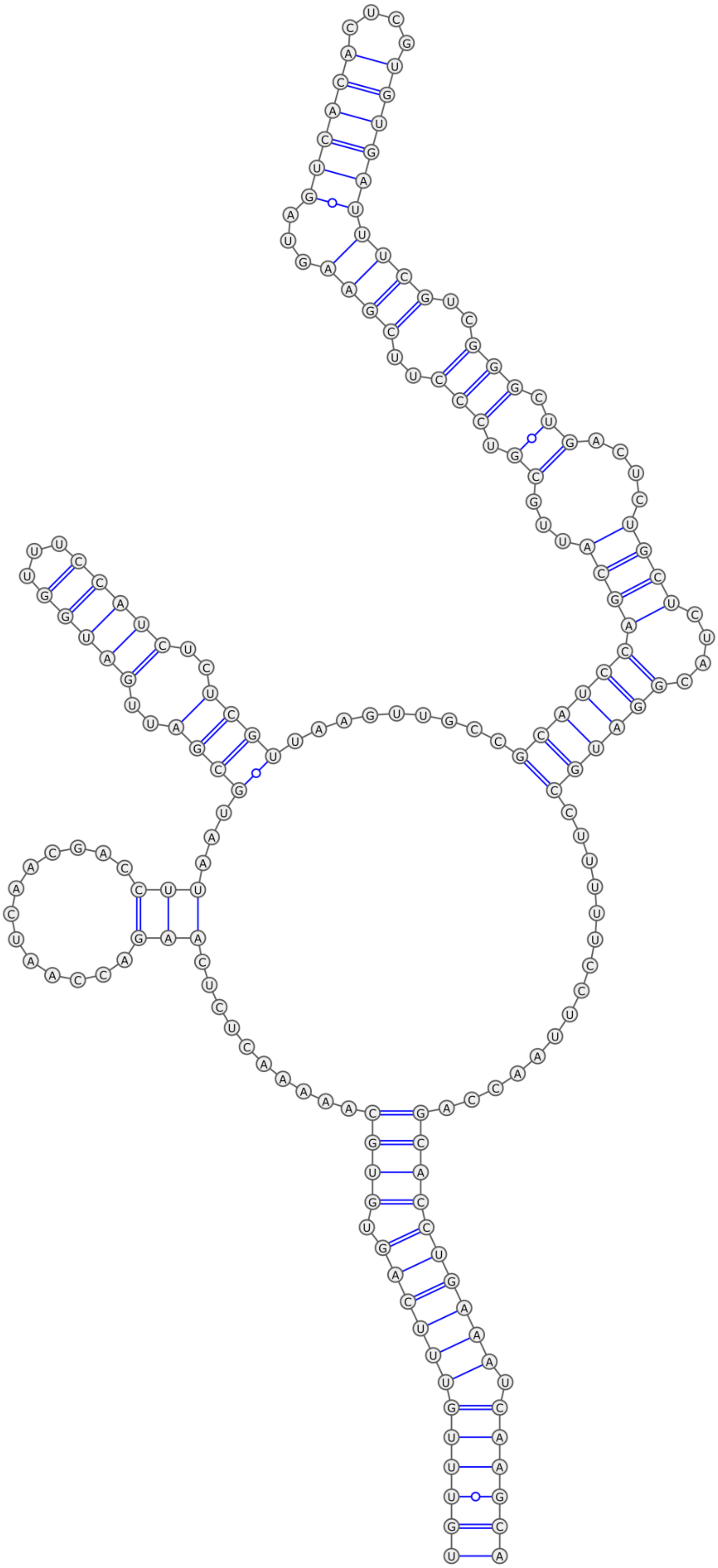

**SUPPLEMENTARY FIGURE 13 |** Putative secondary structure of the ITS2 molecule of *Paraclausilocola constricta* (HQ446275, host earthworm: *Eupolytoreutus* sp.).

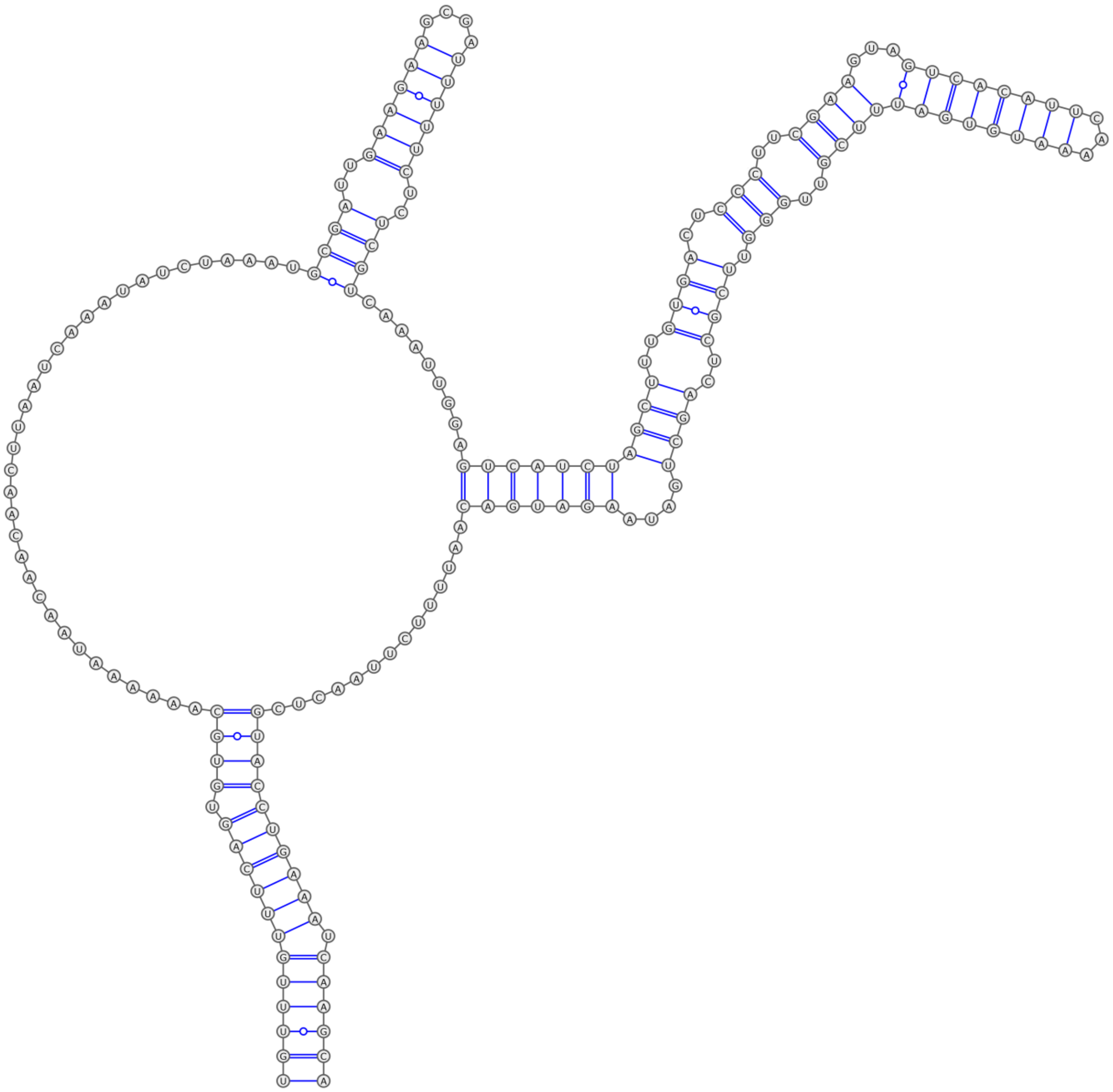

**SUPPLEMENTARY FIGURE 14 |** Putative secondary structure of the ITS2 molecule of *Subanoplophrya nodulata* (samples PU 29 OT and PU 30 OT, host earthworm: *Octolasion tyrtaeum*).

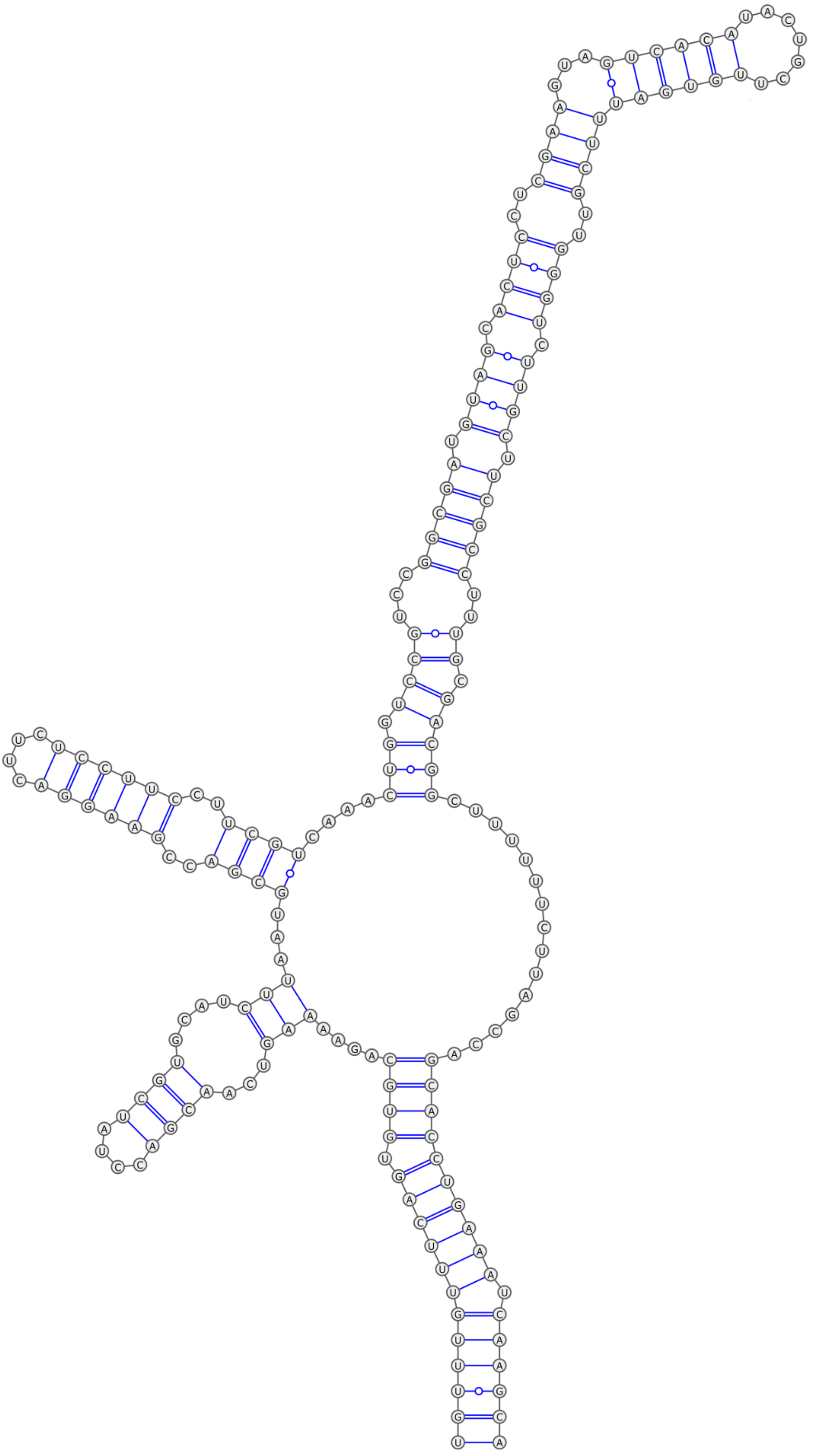

**SUPPLEMENTARY FIGURE 15 |** Putative secondary structure of the ITS2 molecule of *Metaradiophrya chlorotica* (samples JA-2 1M ACH, JA-2 2M ACH, and JA-2 3M ACH, host earthworm: *Allolobophora chlorotica*).

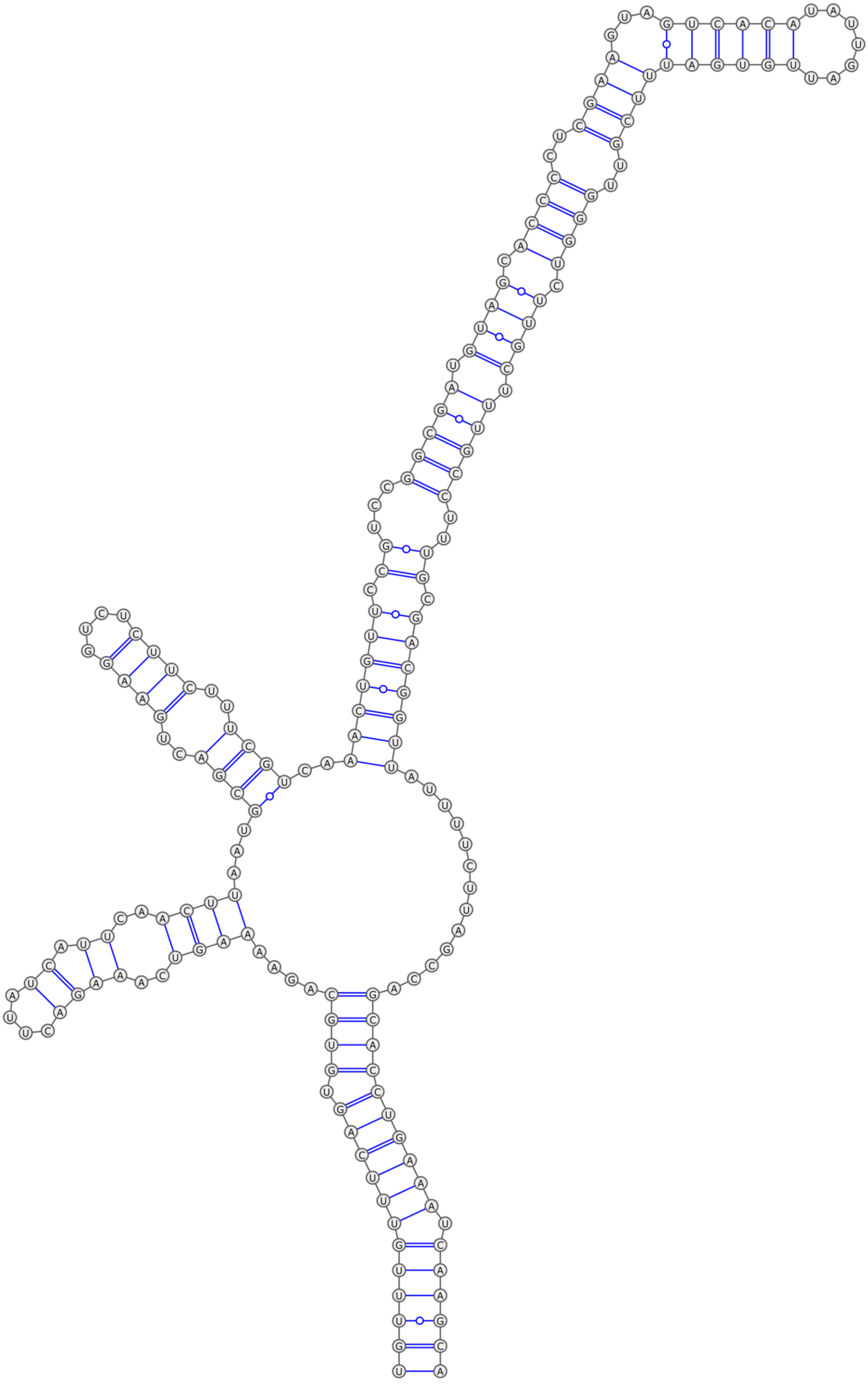

**SUPPLEMENTARY FIGURE 16 |** Putative secondary structure of the ITS2 molecule of *Metaradiophrya lumbrici* (samples RZ 4 LT, RZ 5 LT, KR 8 LT, KR 10 LT, JA-2 25 LT, and JA-2 26 LT, host earthworm: *Lumbricus terrestris*).

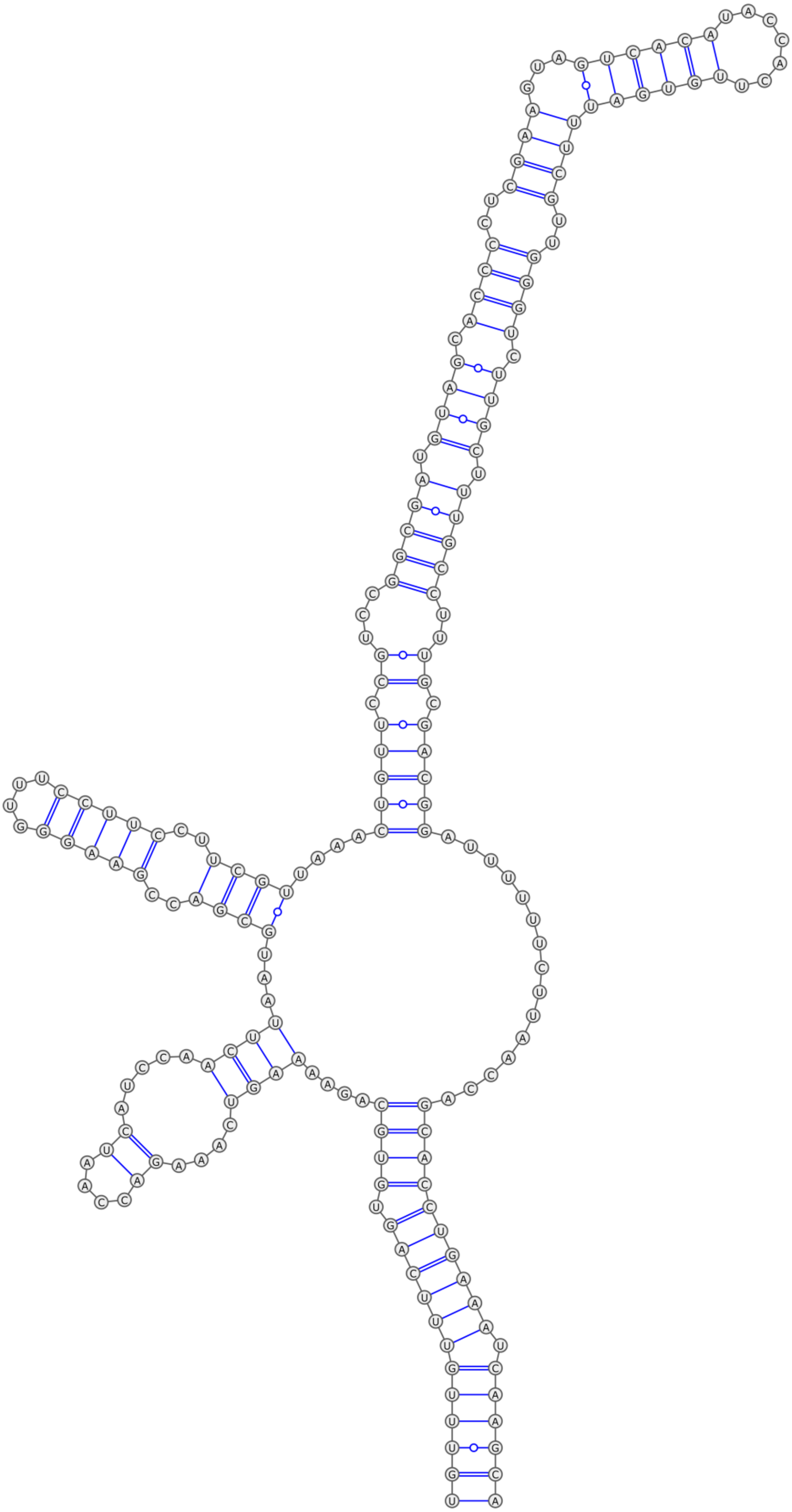

**SUPPLEMENTARY FIGURE 17 |** Putative secondary structure of the ITS2 molecule of *Metaradiophrya speculorum* (samples HkD 59 AT and HkD 60 AT, host earthworm: *Aporrectodea tuberculata*).

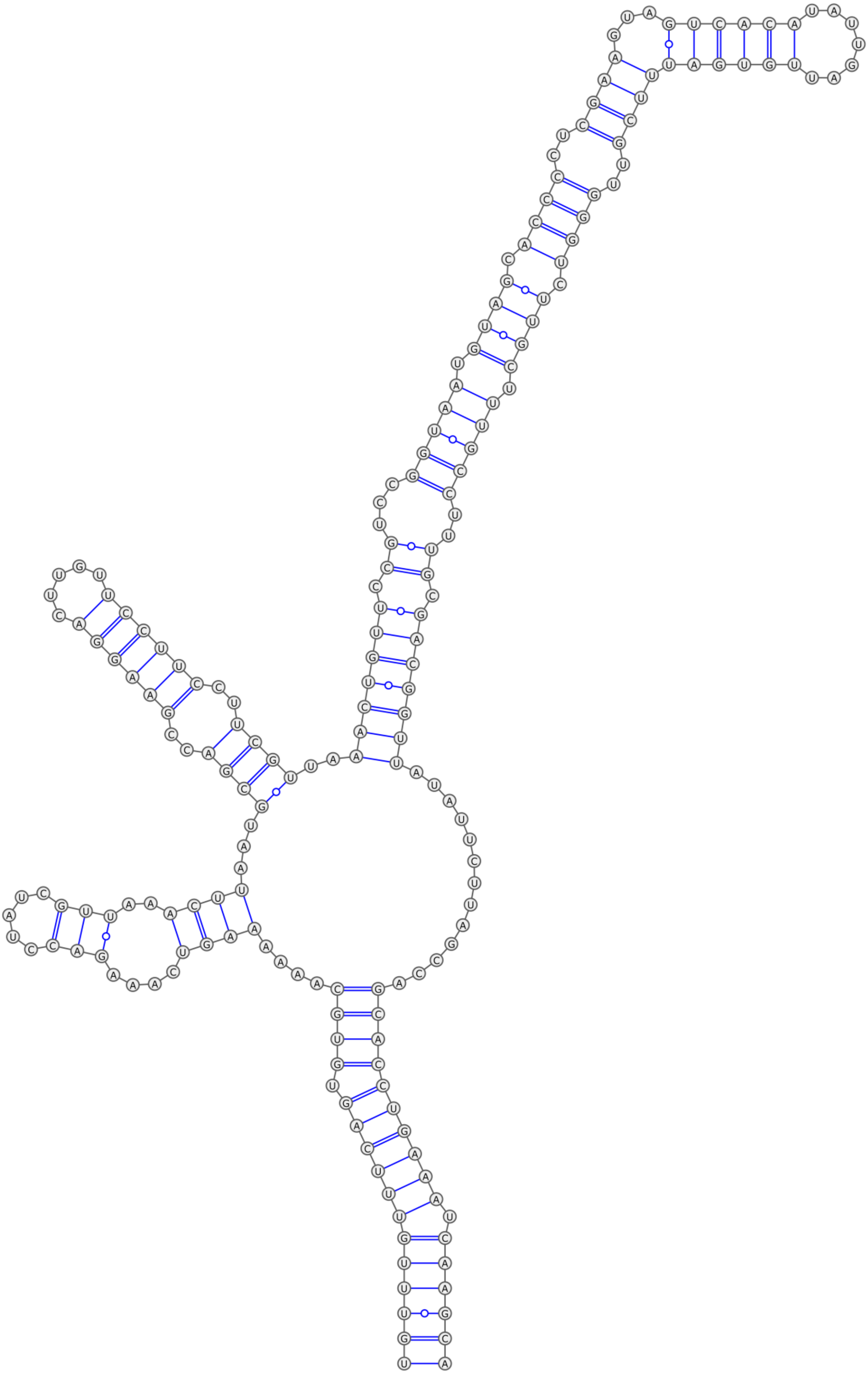

**SUPPLEMENTARY FIGURE 18 |** Putative secondary structure of the ITS2 molecule of *Metaradiophrya varians* (samples BZ 12 EF, BZ 14 EF, JA-1 19 EF, JA-1 22 ED, BZkv 31 EA, and BZkv 32 EA, host earthworms: *Eisenia fetida* complex).

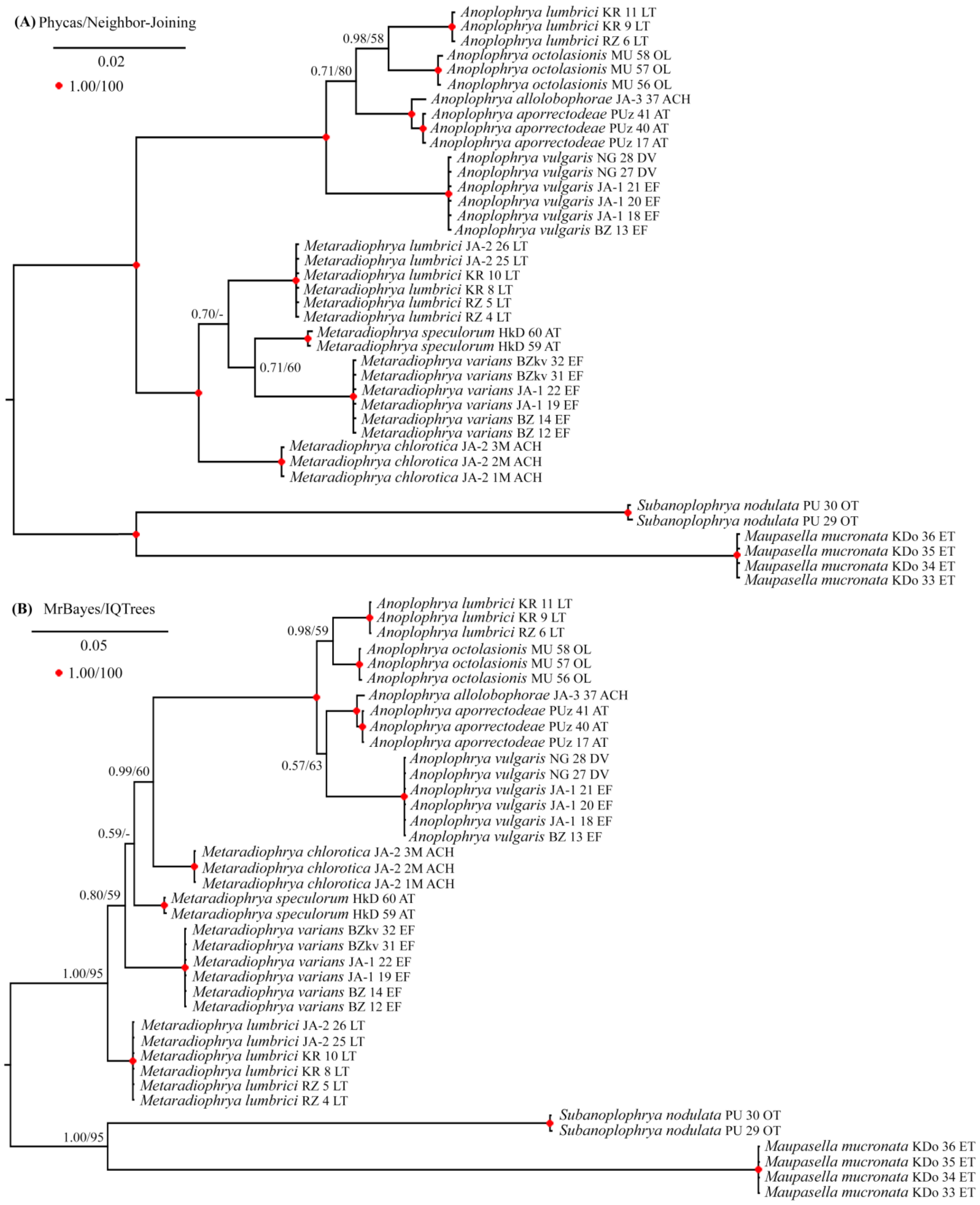


**SUPPLEMENTARY FIGURE 19 |** Phylogenetic trees based on 18S rRNA, ITS region, and D1/D2-28S rRNA gene sequences of astome ciliates isolated from lumbricid earthworms. The positions of *Metaradiophrya* species conflict between the Phycas/neighbor-joining trees **(A)** and MrBayes/IQTree **(B)**. The discrepancy is very likely caused by the plesiomorphic trap, as thoroughly discussed by Obert and Vďačný (2019, 2020). Bootstrap values for neighbor-joining and maximum likelihood IQTree analyses as well as posterior probabilities for Bayesian interference performed in MrBayes and Phycas were mapped onto the respective trees. Fully statistically supported nodes are marked with red solid circles. Dashes indicate statistical support below 50%. For specimen codes and further details, see Supplementary Table 1. Scale bars denote the fraction of substitutions.

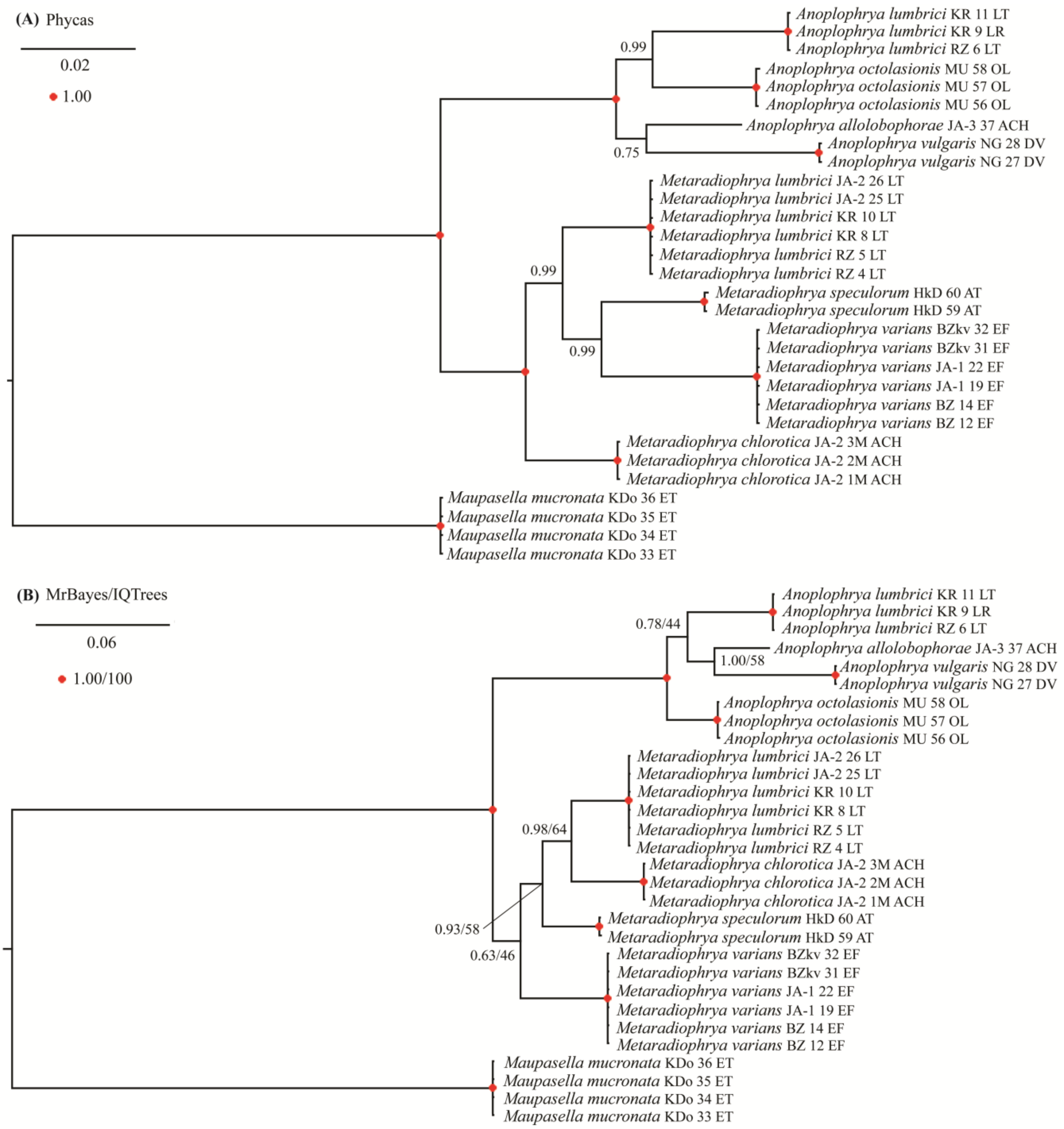


**SUPPLEMENTARY FIGURE 20 |** Phylogenetic trees based on 18S rRNA, ITS region, D1/D2-28S rRNA, and COI gene sequences of astome ciliates isolated from lumbricid earthworms. Note that there is a conflict between the Phycas tree **(A)** and MrBayes/IQTree **(B)** in the position of some *Anoplophrya* and M*etaradiophrya* species. Bootstrap values for maximum likelihood IQTree analyses as well as posterior probabilities for Bayesian interference performed in MrBayes and Phycas were mapped onto the respective trees. Fully statistically supported nodes are marked with red solid circles. Dashes indicate statistical support below 50%. For specimen codes and further details, see Supplementary Table 1. Scale bars denote the fraction of substitutions.

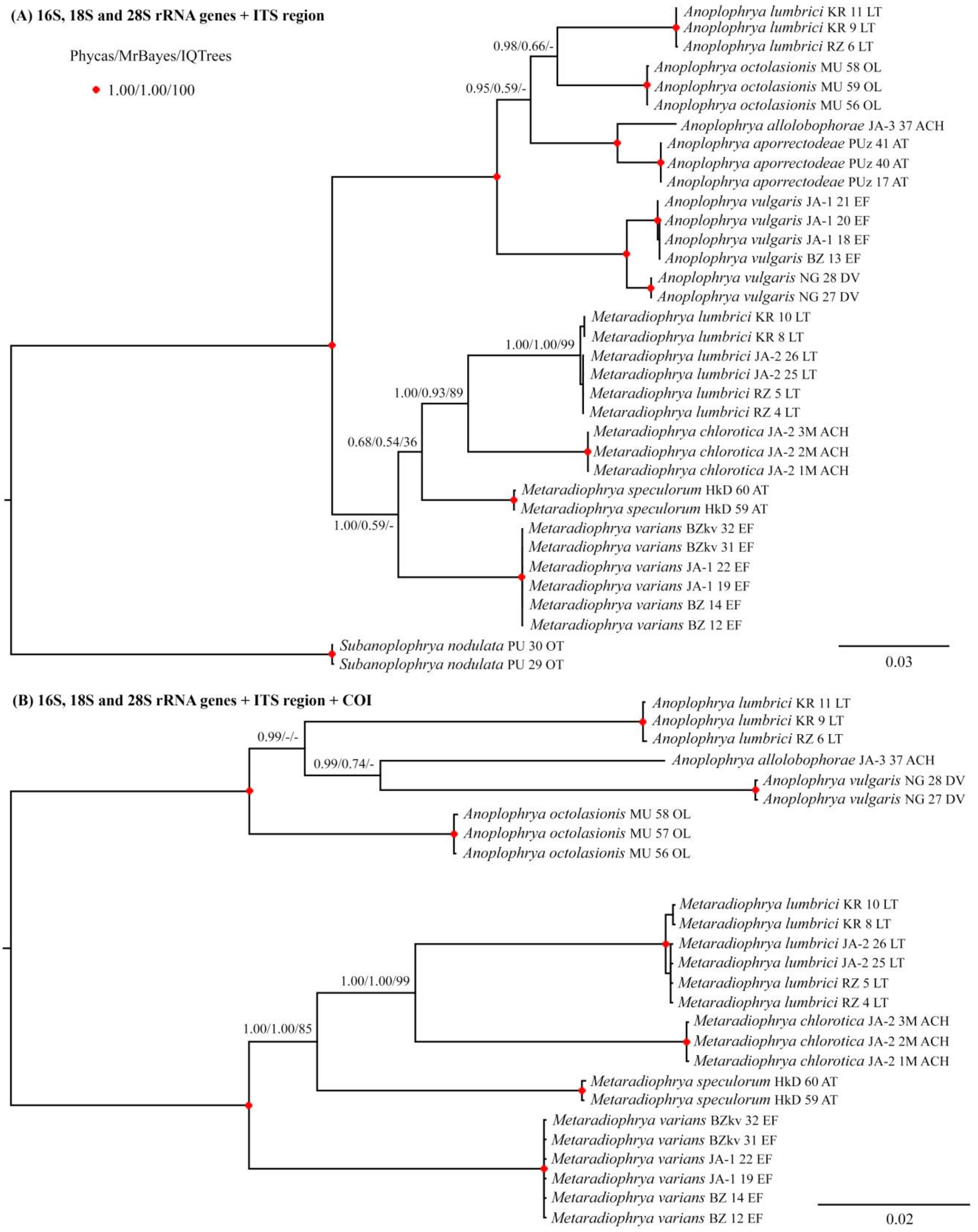


**SUPPLEMENTARY FIGURE 21 |** Phylogenetic trees based on 16S, 18S, 28S rRNA, and ITS region sequences **(A)** and on 16S, 18S, 28S rRNA, ITS region, and COI sequences **(B)** of astome ciliates isolated from lumbricid earthworms. Posterior probabilities for Bayesian interference performed in Phycas and MrBayes as well as bootstrap values for maximum likelihood analyses conducted in IQTrees were mapped onto the 50%-majority rule consensus Phycas trees. Fully statistically supported nodes are marked with red solid circles. Dashes indicate statistical support below 0.50 or 50%. For specimen codes and further details, see Supplementary Table 1. Scale bars denote the fraction of substitutions.

# 18S rRNA gene alignment

**>Anoplophrya_allolobophorae_JA3_37_ACH**
AACCTGGTTGATCCTGCCAGTAGTCATATGCTTGTCTCAAAGATTAAGCCATGCATGTCTAAGTATAAATAGTATACAGTGAAACTGCG
AATGGCTCATTATAACAGTTATAGTTTATTTGATAATTG-AAACCACATGGATAACCGTGGTAATTCTAGAGCTAATACATGCCTAAAA
ACCGGGTTCA-CGCTCGGTTGTATTTATTAGATATCAAACCAATATTCCTTCGGGTCTATTGTGGTGAATCATAGTAACTTAACGAATC
TCGTACTTACGAGATAAATCATTCAAGTTTCTGCCCTATCAGCTTTCGATGGTAGTGTATTGGACTACCATGGCAGTCACGGGTAACGG
AGAATTAGGGTTCGATTCCGGAGAGGGAGCCTGAGAAACGGCTACCACATCTAAGGAAGGCAGCAGGCGCGTAAATTACCCAATCCTGA
CTCAGGGAGGTAGTGACAAGAAATAACAACCTGGGGGC-TATTAGCCTTACGGGATTGCAATGAGAACAATCCAAAACTCTTAGCGAGG
AACAATTGGAGGGCAAGTCTGGTGCCAGCAGCCGCGGTAATTCCAGCTCCAATAGCGTATATTAAAGTTGTTGCAGTTAAAAAGCTCGT
AGTTGAATTTCTGGCTGTTCTTAGTCTTGGCTTCGGTCGAGCTAGGGGCAGTCATCCGTCTGCGAACCATGCTAGGCCTTCACTGGTCG
GTATGGGGAGTAGGCACTTTACTTTGAAAAAATTAGAGTGTTTCAGGCAGGCTTTTGCCCGAATACATTAGCATGGAATAATGGAATAG
GACTTAGTCCATTTTTGTTGGTTATTGGATTGAGTAATGATTAATAGGGACAGTTGGGGGCATTAGTATTTAATTGTCAGAGGTGAAAT
TCTTGGATTTATTAAAGACTAACTTATGCGAAAGCATTTGCCAAGGATGTTTTCATTAATCAAGAACGAAAGTTAGGGGATCAAAGACG
ATCAGATACCGTCCTAGTCTTAACTATAAACTATACCGACTCGGGATCGGCCGGGTA--TTTAAGCTCGGTCGGCACCGTATGAGAAAT
CAAAGTCTTTGGGTTCTGGGGGGAGTATGGTCGCAAGGCTGAAACTTAAAGGAATTGACGGAAGGGCACCACCAGGAGTGGAGCCTGCG
GCTTAATTTGACTCAACACGGGAAAACTTACCAGGTCAAAACATGGGTGAGATTGACAGATTGAGAGCTCTTTCTTGATTCTATGGGTG
GTGGTGCATGGCCGTTCTTAGTTGGTGGAGTGATTTGTCTGGTTAATTCCGTTAACGAACGAGACCTTAACCTGCTAACTAGTCACTGG
AACTTCATTCTAGTGTACTTCTTAGAGGGACTATGCTGCGGAAGCGCATGGAAGTTTGAGGCAATAACAGGTCTGTGATGCCCCTAGAC
GTCCTGGGCCGCACGCGCGCTACAATGACACGCTCAGCAAGTATTATCCTGACCCGGAAGGGCTTCGGTAATCTTCACAATACGTGTCG
TGTTAGGGATAGATCTTTGCAATTATGGATCTTGAACGAGGAATTCCTAGTAAGTGCAAGTCATTAGCTTGTACTGATTACGTCCCTGC
CCTTTGTACACACCGCCCGTCGCTCCTACCGATTTCGAGTGGTCAGGTGAACATTCCGGACTGCAATTTCC-------CAATTGTAGAA
AGTTAAGTAAACCTTATCACTTAGAGGAAGGAAAAGTCGTAACAAGGTTTCCGTAGGTGAACCTGCAGAAGGATCA
**>Anoplophrya_aporrectodeae_PUz_17_AT**
AACCTGGTTGATCCTGCCAGTAGTCATATGCTTGTCTCAAAGATTAAGCCATGCATGTCTAAGTATAAATAGTATACAGTGAAACTGCG
AATGGCTCATTATAACAGTTATAGTTTATTTGATAATTG-AAACCACATGGATAACCGTGGTAATTCTAGAGCTAATACATGCCTAAAA
ACCGGGTTCA-CGCTCGGTTGTATTTATTAGATATCAAACCAATATTCCTTCGGGTCTATTGTGGTGAATCATAGTAACTTAACGAATC
TCGTACTTACGAGATAAATCATTCAAGTTTCTGCCCTATCAGCTTTCGATGGTAGTGTATTGGACTACCATGGCAGTCACGGGTAACGG
AGAATTAGGGTTCGATTCCGGAGAGGGAGCCTGAGAAACGGCTACCACATCTGAGGAAGGCAGCAGGCGCGTAAATTACCCAATCCTGA
CTCAGGGAGGTAGTGACAAGAAATAACAACCTGGGGGC-TATTAGCCTTACGGGATTGCAATGAGAACAATCCAAAACTCTTAGCGAGG
AACAATTGGAGGGCAAGTCTGGTGCCAGCAGCCGCGGTAATTCCAGCTCCAATAGCGTATATTAAAGTTGTTGCAGTTAAAAAGCTCGT
AGTTGAATTTCTGGCTGTTCTTAGTCTTGGCTTCGGTCGAGCTAGGGGCAGTCATCCGTCTGCGAACCATGCTAGGCCTTTACTGGTCG
GTATGAGGAGTAGGCACTTTACTTTGAAAAAATTAGAGTGTTTCAGGCAGGCTTTTGCCCGAATACATTAGCATGGAATAATGGAATAG
GACTTAGTCCATTTTTGTTGGTTATTGGATTGAGTAATGATTAATAGGGACAGTTGGGGGCATTAGTATTTAATTGTCAGAGGTGAAAT
TCTTGGATTTATTAAAGACTAACTTATGCGAAAGCATTTGCCAAGGATGTTTTCATTAATCAAGAACGAAAGTTAGGGGATCAAAGACG
ATCAGATACCGTCCTAGTCTTAACTATAAACTATACCGACTCGGGATCGGCCGGGTA--TTTAAGCTCGGTCGGCACCGTATGAGAAAT
CAAAGTCTTTGGGTTCTGGGGGGAGTATGGTCGCAAGGCTGAAACTTAAAGGAATTGACGGAAGGGCACCACCAGGAGTGGAGCCTGCG
GCTTAATTTGACTCAACACGGGAAAACTTACCAGGTCAAAACATGGGTGAGATTGACAGATTGAGAGCTCTTTCTTGATTCTATGGGTG
GTGGTGCATGGCCGTTCTTAGTTGGTGGAGTGATTTGTCTGGTTAATTCCGTTAACGAACGAGACCTTAACCTGCTAACTAGTCACTGG
AACTTCATTCTAGTGTACTTCTTAGAGGGACTATGCTGCGGAAGCGCATGGAAGTTTGAGGCAATAACAGGTCTGTGATGCCCCTAGAC
GTCCTGGGCCGCACGCGCGCTACAATGACACGCTCAGCAAGTATTATCCTGACCCGGAAGGGCTTCGGTAATCTTCACAATACGTGTCG
TGTTAGGGATAGATCTTTGCAATTATGGATCTTGAACGAGGAATTCCTAGTAAGTGCAAGTCATTAGCTTGTACTGATTACGTCCCTGC
CCTTTGTACACACCGCCCGTCGCTCCTACCGATTTCGAGTGGTCAGGTGAACATTCCGGACTGCAATTTCC-------CAATTGTAGAA
AGTTAAGTAAACCTTATCACTTAGAGGAAGGAAAAGTCGTAACAAGGTTTCCGTAGGTGAACCTGCAGAAGGATCA
**>Anoplophrya_aporrectodeae_PUz_40_AT**
AACCTGGTTGATCCTGCCAGTAGTCATATGCTTGTCTCAAAGATTAAGCCATGCATGTCTAAGTATAAATAGTATACAGTGAAACTGCG
AATGGCTCATTATAACAGTTATAGTTTATTTGATAATTG-AAACCACATGGATAACCGTGGTAATTCTAGAGCTAATACATGCCTAAAA
ACCGGGTTCA-CGCTCGGTTGTATTTATTAGATATCAAACCAATATTCCTTCGGGTCTATTGTGGTGAATCATAGTAACTTAACGAATC
TCGTACTTACGAGATAAATCATTCAAGTTTCTGCCCTATCAGCTTTCGATGGTAGTGTATTGGACTACCATGGCAGTCACGGGTAACGG
AGAATTAGGGTTCGATTCCGGAGAGGGAGCCTGAGAAACGGCTACCACATCTGAGGAAGGCAGCAGGCGCGTAAATTACCCAATCCTGA
CTCAGGGAGGTAGTGACAAGAAATAACAACCTGGGGGC-TATTAGCCTTACGGGATTGCAATGAGAACAATCCAAAACTCTTAGCGAGG
AACAATTGGAGGGCAAGTCTGGTGCCAGCAGCCGCGGTAATTCCAGCTCCAATAGCGTATATTAAAGTTGTTGCAGTTAAAAAGCTCGT
AGTTGAATTTCTGGCTGTTCTTAGTCTTGGCTTCGGTCGAGCTAGGGGCAGTCATCCGTCTGCGAACCATGCTAGGCCTTTACTGGTCG
GTATGAGGAGTAGGCACTTTACTTTGAAAAAATTAGAGTGTTTCAGGCAGGCTTTTGCCCGAATACATTAGCATGGAATAATGGAATAG
GACTTAGTCCATTTTTGTTGGTTATTGGATTGAGTAATGATTAATAGGGACAGTTGGGGGCATTAGTATTTAATTGTCAGAGGTGAAAT
TCTTGGATTTATTAAAGACTAACTTATGCGAAAGCATTTGCCAAGGATGTTTTCATTAATCAAGAACGAAAGTTAGGGGATCAAAGACG
ATCAGATACCGTCCTAGTCTTAACTATAAACTATACCGACTCGGGATCGGCCGGGTA--TTTAAGCTCGGTCGGCACCGTATGAGAAAT
CAAAGTCTTTGGGTTCTGGGGGGAGTATGGTCGCAAGGCTGAAACTTAAAGGAATTGACGGAAGGGCACCACCAGGAGTGGAGCCTGCG
GCTTAATTTGACTCAACACGGGAAAACTTACCAGGTCAAAACATGGGTGAGATTGACAGATTGAGAGCTCTTTCTTGATTCTATGGGTG
GTGGTGCATGGCCGTTCTTAGTTGGTGGAGTGATTTGTCTGGTTAATTCCGTTAACGAACGAGACCTTAACCTGCTAACTAGTCACTGG
AACTTCATTCTAGTGTACTTCTTAGAGGGACTATGCTGCGGAAGCGCATGGAAGTTTGAGGCAATAACAGGTCTGTGATGCCCCTAGAC
GTCCTGGGCCGCACGCGCGCTACAATGACACGCTCAGCAAGTATTATCCTGACCCGGAAGGGCTTCGGTAATCTTCACAATACGTGTCG
TGTTAGGGATAGATCTTTGCAATTATGGATCTTGAACGAGGAATTCCTAGTAAGTGCAAGTCATTAGCTTGTACTGATTACGTCCCTGC
CCTTTGTACACACCGCCCGTCGCTCCTACCGATTTCGAGTGGTCAGGTGAACATTCCGGACTGCAATTTCC-------CAATTGTAGAA
AGTTAAGTAAACCTTATCACTTAGAGGAAGGAAAAGTCGTAACAAGGTTTCCGTAGGTGAACCTGCAGAAGGATCA
**>Anoplophrya_aporrectodeae_PUz_41_AT**
AACCTGGTTGATCCTGCCAGTAGTCATATGCTTGTCTCAAAGATTAAGCCATGCATGTCTAAGTATAAATAGTATACAGTGAAACTGCG
AATGGCTCATTATAACAGTTATAGTTTATTTGATAATTG-AAACCACATGGATAACCGTGGTAATTCTAGAGCTAATACATGCCTAAAA
ACCGGGTTCA-CGCTCGGTTGTATTTATTAGATATCAAACCAATATTCCTTCGGGTCTATTGTGGTGAATCATAGTAACTTAACGAATC
TCGTACTTACGAGATAAATCATTCAAGTTTCTGCCCTATCAGCTTTCGATGGTAGTGTATTGGACTACCATGGCAGTCACGGGTAACGG

AGAATTAGGGTTCGATTCCGGAGAGGGAGCCTGAGAAACGGCTACCACATCTGAGGAAGGCAGCAGGCGCGTAAATTACCCAATCCTGA
CTCAGGGAGGTAGTGACAAGAAATAACAACCTGGGGGC-TATTAGCCTTACGGGATTGCAATGAGAACAATCCAAAACTCTTAGCGAGG
AACAATTGGAGGGCAAGTCTGGTGCCAGCAGCCGCGGTAATTCCAGCTCCAATAGCGTATATTAAAGTTGTTGCAGTTAAAAAGCTCGT
AGTTGAATTTCTGGCTGTTCTTAGTCTTGGCTTCGGTCGAGCTAGGGGCAGTCATCCGTCTGCGAACCATGCTAGGCCTTTACTGGTCG
GTATGAGGAGTAGGCACTTTACTTTGAAAAAATTAGAGTGTTTCAGGCAGGCTTTTGCCCGAATACATTAGCATGGAATAATGGAATAG
GACTTAGTCCATTTTTGTTGGTTATTGGATTGAGTAATGATTAATAGGGACAGTTGGGGGCATTAGTATTTAATTGTCAGAGGTGAAAT
TCTTGGATTTATTAAAGACTAACTTATGCGAAAGCATTTGCCAAGGATGTTTTCATTAATCAAGAACGAAAGTTAGGGGATCAAAGACG
ATCAGATACCGTCCTAGTCTTAACTATAAACTATACCGACTCGGGATCGGCCGGGTA--TTTAAGCTCGGTCGGCACCGTATGAGAAAT
CAAAGTCTTTGGGTTCTGGGGGGAGTATGGTCGCAAGGCTGAAACTTAAAGGAATTGACGGAAGGGCACCACCAGGAGTGGAGCCTGCG
GCTTAATTTGACTCAACACGGGAAAACTTACCAGGTCAAAACATGGGTGAGATTGACAGATTGAGAGCTCTTTCTTGATTCTATGGGTG
GTGGTGCATGGCCGTTCTTAGTTGGTGGAGTGATTTGTCTGGTTAATTCCGTTAACGAACGAGACCTTAACCTGCTAACTAGTCACTGG
AACTTCATTCTAGTGTACTTCTTAGAGGGACTATGCTGCGGAAGCGCATGGAAGTTTGAGGCAATAACAGGTCTGTGATGCCCCTAGAC
GTCCTGGGCCGCACGCGCGCTACAATGACACGCTCAGCAAGTATTATCCTGACCCGGAAGGGCTTCGGTAATCTTCACAATACGTGTCG
TGTTAGGGATAGATCTTTGCAATTATGGATCTTGAACGAGGAATTCCTAGTAAGTGCAAGTCATTAGCTTGTACTGATTACGTCCCTGC
CCTTTGTACACACCGCCCGTCGCTCCTACCGATTTCGAGTGGTCAGGTGAACATTCCGGACTGCAATTTCC-------CAATTGTAGAA
AGTTAAGTAAACCTTATCACTTAGAGGAAGGAAAAGTCGTAACAAGGTTTCCGTAGGTGAACCTGCAGAAGGATCA

**>Anoplophrya_lumbrici_RZ_6_LT**

AACCTGGTTGATCCTGCCAGTAGTCATATGCTTGTCTCAAAGATTAAGCCATGCATGTCTAAGTATAAATAGTATACAGTGAAACTGCG
AATGGCTCATTATAACAGTTATAGTTTATTTGATAATTG-AAACCACATGGATAACCGTGGTAATTCTAGAGCTAATACATGCCTAAAA
ACTGGGCTCA-CGCTCAGTTGTATTTATTAGATATCAAACCAATATTCCTTCGGGTCTATTGTGGTGAATCATAGTAACTTAACGAATC
TCGTATTTACGAGATAAATCATTCAAGTTTCTGCCCTATCAGCTTTCGATGGTAGTGTATTGGACTACCATGGCAATCACGGGTAACGG
AGAATTAGGGTTCGATTCCGGAGAGGGAGCCTGAGAAACGGCTACCACATCTAAGGAAGGCAGCAGGCGCGTAAATTACCCAATCCTGA
TTCAGGGAGGTAGTGACAAGAAATAACAACCTGGGGGC-TATTAGCCTTACGGGATTGCAATGAGAACAATCCAAAACTCTTAGCGAGG
AACAATTGGAGGGCAAGTCTGGTGCCAGCAGCCGCGGTAATTCCAGCTCCAATAGCGTATATTAAAGTTGTTGCAGTTAAAAAGCTCGT
AGTTGAATTTCTGTCTGTTTCTAGTCTTGGCCTCGGTCGAGCTAGGGGCAGTCATCCGTCTGCAAACCATGCTAGTCCTTCATTGGTCG
GTATGGGGAGTAGGCACTTTACTTTGAAAAAATTAGAGTGTTTCAGGCAGGCTTTTGCCCGAATACATTAGCATGGAATAATGGAATAG
GACTTAGTCCATTTTTGTTGGTTATTGGATTGAGTAATGATTAATAGGGACAGTTGGGGGCATTAGTATTTAATTGTCAGAGGTGAAAT
TCTTGGATTTATTAAAGACTAACTTATGCGAAAGCATTTGCCAAGGATGTTTTCATTAATCAAGAACGAAAGTTAGGGGATCAAAGACG
ATCAGATACCGTCCTAGTCTTAACTATAAACTATACCGACTCGGGATCGGCCGGGTA--TTTAAGCTCGGTCGGCACCGTATGAGAAAT
CAAAGTCTTTGGGTTCTGGGGGGAGTATGGTCGCAAGGCTGAAACTTAAAGGAATTGACGGAAGGGCACCACCAGGAGTGGAGCCTGCG
GCTTAATTTGACTCAACACGGGAAAACTTACCAGGTCAAAACATGGGTGAGATTGACAGATTGAGAGCTCTTTCTTGATTCTATGGGTG
GTGGTGCATGGCCGTTCTTAGTTGGTGGAGTGATTTGTCTGGTTAATTCCGTTAACGAACGAGACCTTAACCTGCTAACTAGTCATTTG
AACTTTATTCGAATGTACTTCTTAGAGGGACTATGCTGCGGAAGCGCATGGAAGTTTGAGGCAATAACAGGTCTGTGATGCCCCTAGAC
GTCCTGGGCCGCACGCGCGCTACAATGACACGCTCAGCAAGCATTTTCCTGACCCGGAAGGG-TTCGGTAATCTTCACAATACGTGTCG
TGTTAGGGATAGATCTTTGCAATTATGGATCTTGAACGAGGAATTCCTAGTAAGTGCAAGTCATTAGCTTGTACTGATTACGTCCCTGC
CCTTTGTACACACCGCCCGTCGCTCCTACCGATTTCGAGTGATCAGGTGAACATTCCGGACTGCAGTTTCC-------CAATTGTAGAA
AGTTAAGTAAACCTTATCACTTAGAGGAAGGAAAAGTCGTAACAAGGTTTCCGTAGGTGAACCTGCAGAAGGATCA

**>Anoplophrya_lumbrici_KR_9_LT**

AACCTGGTTGATCCTGCCAGTAGTCATATGCTTGTCTCAAAGATTAAGCCATGCATGTCTAAGTATAAATAGTATACAGTGAAACTGCG
AATGGCTCATTATAACAGTTATAGTTTATTTGATAATTG-AAACCACATGGATAACCGTGGTAATTCTAGAGCTAATACATGCCTAAAA
ACTGGGCTCA-CGCTCAGTTGTATTTATTAGATATCAAACCAATATTCCTTCGGGTCTATTGTGGTGAATCATAGTAACTTAACGAATC
TCGTATTTACGAGATAAATCATTCAAGTTTCTGCCCTATCAGCTTTCGATGGTAGTGTATTGGACTACCATGGCAATCACGGGTAACGG
AGAATTAGGGTTCGATTCCGGAGAGGGAGCCTGAGAAACGGCTACCACATCTAAGGAAGGCAGCAGGCGCGTAAATTACCCAATCCTGA
TTCAGGGAGGTAGTGACAAGAAATAACAACCTGGGGGC-TATTAGCCTTACGGGATTGCAATGAGAACAATCCAAAACTCTTAGCGAGG
AACAATTGGAGGGCAAGTCTGGTGCCAGCAGCCGCGGTAATTCCAGCTCCAATAGCGTATATTAAAGTTGTTGCAGTTAAAAAGCTCGT
AGTTGAATTTCTGTCTGTTTCTAGTCTTGGCCTCGGTCGAGCTAGGGGCAGTCATCCGTCTGCAAACCATGCTAGTCCTTCATTGGTCG
GTATGGGGAGTAGGCACTTTACTTTGAAAAAATTAGAGTGTTTCAGGCAGGCTTTTGCCCGAATACATTAGCATGGAATAATGGAATAG
GACTTAGTCCATTTTTGTTGGTTATTGGATTGAGTAATGATTAATAGGGACAGTTGGGGGCATTAGTATTTAATTGTCAGAGGTGAAAT
TCTTGGATTTATTAAAGACTAACTTATGCGAAAGCATTTGCCAAGGATGTTTTCATTAATCAAGAACGAAAGTTAGGGGATCAAAGACG
ATCAGATACCGTCCTAGTCTTAACTATAAACTATACCGACTCGGGATCGGCCGGGTA--TTTAAGCTCGGTCGGCACCGTATGAGAAAT
CAAAGTCTTTGGGTTCTGGGGGGAGTATGGTCGCAAGGCTGAAACTTAAAGGAATTGACGGAAGGGCACCACCAGGAGTGGAGCCTGCG
GCTTAATTTGACTCAACACGGGAAAACTTACCAGGTCAAAACATGGGTGAGATTGACAGATTGAGAGCTCTTTCTTGATTCTATGGGTG
GTGGTGCATGGCCGTTCTTAGTTGGTGGAGTGATTTGTCTGGTTAATTCCGTTAACGAACGAGACCTTAACCTGCTAACTAGTCATTTG
AACTTTATTCGAATGTACTTCTTAGAGGGACTATGCTGCGGAAGCGCATGGAAGTTTGAGGCAATAACAGGTCTGTGATGCCCCTAGAC
GTCCTGGGCCGCACGCGCGCTACAATGACACGCTCAGCAAGCATTTTCCTGACCCGGAAGGG-TTCGGTAATCTTCACAATACGTGTCG
TGTTAGGGATAGATCTTTGCAATTATGGATCTTGAACGAGGAATTCCTAGTAAGTGCAAGTCATTAGCTTGTACTGATTACGTCCCTGC
CCTTTGTACACACCGCCCGTCGCTCCTACCGATTTCGAGTGATCAGGTGAACATTCCGGACTGCAGTTTCC-------CAATTGTAGAA
AGTTAAGTAAACCTTATCACTTAGAGGAAGGAAAAGTCGTAACAAGGTTTCCGTAGGTGAACCTGCAGAAGGATCA

**>Anoplophrya_lumbrici_KR_11_LT**

AACCTGGTTGATCCTGCCAGTAGTCATATGCTTGTCTCAAAGATTAAGCCATGCATGTCTAAGTATAAATAGTATACAGTGAAACTGCG
AATGGCTCATTATAACAGTTATAGTTTATTTGATAATTG-AAACCACATGGATAACCGTGGTAATTCTAGAGCTAATACATGCCTAAAA
ACTGGGCTCA-CGCTCAGTTGTATTTATTAGATATCAAACCAATATTCCTTCGGGTCTATTGTGGTGAATCATAGTAACTTAACGAATC
TCGTATTTACGAGATAAATCATTCAAGTTTCTGCCCTATCAGCTTTCGATGGTAGTGTATTGGACTACCATGGCAATCACGGGTAACGG
AGAATTAGGGTTCGATTCCGGAGAGGGAGCCTGAGAAACGGCTACCACATCTAAGGAAGGCAGCAGGCGCGTAAATTACCCAATCCTGA
TTCAGGGAGGTAGTGACAAGAAATAACAACCTGGGGGC-TATTAGCCTTACGGGATTGCAATGAGAACAATCCAAAACTCTTAGCGAGG
AACAATTGGAGGGCAAGTCTGGTGCCAGCAGCCGCGGTAATTCCAGCTCCAATAGCGTATATTAAAGTTGTTGCAGTTAAAAAGCTCGT
AGTTGAATTTCTGTCTGTTTCTAGTCTTGGCCTCGGTCGAGCTAGGGGCAGTCATCCGTCTGCAAACCATGCTAGTCCTTCATTGGTCG
GTATGGGGAGTAGGCACTTTACTTTGAAAAAATTAGAGTGTTTCAGGCAGGCTTTTGCCCGAATACATTAGCATGGAATAATGGAATAG
GACTTAGTCCATTTTTGTTGGTTATTGGATTGAGTAATGATTAATAGGGACAGTTGGGGGCATTAGTATTTAATTGTCAGAGGTGAAAT
TCTTGGATTTATTAAAGACTAACTTATGCGAAAGCATTTGCCAAGGATGTTTTCATTAATCAAGAACGAAAGTTAGGGGATCAAAGACG
ATCAGATACCGTCCTAGTCTTAACTATAAACTATACCGACTCGGGATCGGCCGGGTA--TTTAAGCTCGGTCGGCACCGTATGAGAAAT

CAAAGTCTTTGGGTTCTGGGGGGAGTATGGTCGCAAGGCTGAAACTTAAAGGAATTGACGGAAGGGCACCACCAGGAGTGGAGCCTGCG
GCTTAATTTGACTCAACACGGGAAAACTTACCAGGTCAAAACATGGGTGAGATTGACAGATTGAGAGCTCTTTCTTGATTCTATGGGTG
GTGGTGCATGGCCGTTCTTAGTTGGTGGAGTGATTTGTCTGGTTAATTCCGTTAACGAACGAGACCTTAACCTGCTAACTAGTCATTTG
AACTTTATTCGAATGTACTTCTTAGAGGGACTATGCTGCGGAAGCGCATGGAAGTTTGAGGCAATAACAGGTCTGTGATGCCCCTAGAC
GTCCTGGGCCGCACGCGCGCTACAATGACACGCTCAGCAAGCATTTTCCTGACCCGGAAGGG-TTCGGTAATCTTCACAATACGTGTCG
TGTTAGGGATAGATCTTTGCAATTATGGATCTTGAACGAGGAATTCCTAGTAAGTGCAAGTCATTAGCTTGTACTGATTACGTCCCTGC
CCTTTGTACACACCGCCCGTCGCTCCTACCGATTTCGAGTGATCAGGTGAACATTCCGGACTGCAGTTTCC-------CAATTGTAGAA
AGTTAAGTAAACCTTATCACTTAGAGGAAGGAAAAGTCGTAACAAGGTTTCCGTAGGTGAACCTGCAGAAGGATCA
**>Anoplophrya_octolasionis_MU_56_OL**
AACCTGGTTGATCCTGCCAGTAGTCATATGCTTGTCTCAAAGATTAAGCCATGCATGTCTAAGTATAAATAGTATACAGTGAAACTGCG
AATGGCTCATTATAACAGTTATAGTTTATTTGATAATTG-AAACCACATGGATAACCGTGGTAATTCTAGAGCTAATACATGCCTAAAA
GCCGGGTTCA-CGCTCGGCTGTATTTATTAGATATCAAACCAATATTCCTTCGGGTCTATTGTGGTGAATCATAGTAACTTAACGAATC
TCGAACTTTCGAGATAAATCATTCAAGTTTCTGCCCTATCAGCTTTCGATGGTAGTGTATTGGACTACCATGGCAGTCACGGGTAACGG
AGAATTAGGGTTCGATTCCGGAGAGGGAGCCTGAGAAACGGCTACCACATCTAAGGAAGGCAGCAGGCGCGTAAATTACCCAATCCTGA
TTCAGGGAGGTAGTGACAAGAAATAACAACCTGGGGGC-TATTAGCCTTACGGGATTGCAATGAGAACAATCCAAAACTCTTAGCGAGG
AACAATTGGAGGGCAAGTCTGGTGCCAGCAGCCGCGGTAATTCCAGCTCCAATAGCGTATATTAAAGTTGTTGCAGTTAAAAAGCTCGT
AGTTGAATTTCTGGCTGTTTCTAGTCTTGGCCTCGGTCGAGCTAGGGGCAGTCATCCGTCTGCAAACCATGCTAGTCCTTTACTGGTCG
GTATGGGGAGTAGGCATTTTACTTTGAAAAAATTAGAGTGTTTCAGGCAGGCTTTTGCCCGAATACATTAGCATGGAATAATGGAATAG
GACTTAGTCCATTTTTGTTGGTTATTGGATTGAGTAATGATTAATAGGGACAGTTGGGGGCATTAGTATTTAATTGTCAGAGGTGAAAT
TCTTGGATTTATTAAAGACTAACTTATGCGAAAGCATTTGCCAAGGATGTTTTCATTAATCAAGAACGAAAGTTAGGGGATCAAAGACG
ATCAGATACCGTCCTAGTCTTAACTATAAACTATACCGACTCGGGATCGGCCGGGTA--TTTAAGCTCGGTCGGCACCGTATGAGAAAT
CAAAGTCTTTGGGTTCTGGGGGGAGTATGGTCGCAAGGCTGAAACTTAAAGGAATTGACGGAAGGGCACCACCAGGAGTGGAGCCTGCG
GCTTAATTTGACTCAACACGGGAAAACTTACCAGGTCAAAACATGGGTGAGATTGACAGATTGAGAGCTCTTTCTTGATTCTATGGGTG
GTGGTGCATGGCCGTTCTTAGTTGGTGGAGTGATTTGTCTGGTTAATTCCGTTAACGAACGAGACCTTAACCTGCTAACTAGTCATTTG
AACTTTATTCGAGTGTACTTCTTAGAGGGACTATGCTGCGGAAGCGCATGGAAGTTTGAGGCAATAACAGGTCTGTGATGCCCCTAGAC
GTCCTGGGCCGCACGCGCGCTACAATGACACGCTCAGCAAGTATTATCCTGACCCGGAAGGGCTTCGGTAATCTTCACAATACGTGTCG
TGTTAGGGATAGATCTTTGCAATTATGGATCTTGAACGAGGAATTCCTAGTAAGTGCAAGTCATTAGCTTGTACTGATTACGTCCCTGC
CCTTTGTACACACCGCCCGTCGCTCCTACCGATTTCGAGTGATCAGGTGAACATTCCGGACTGCAATTTCC-------CAATTGTAGAA
AGTTAAGTAAACCTTATCACTTAGAGGAAGGAAAAGTCGTAACAAGGTTTCCGTAGGTGAACCTGCAGAAGGATCA
**>Anoplophrya_octolasionis_MU_57_OL**
AACCTGGTTGATCCTGCCAGTAGTCATATGCTTGTCTCAAAGATTAAGCCATGCATGTCTAAGTATAAATAGTATACAGTGAAACTGCG
AATGGCTCATTATAACAGTTATAGTTTATTTGATAATTG-AAACCACATGGATAACCGTGGTAATTCTAGAGCTAATACATGCCTAAAA
GCCGGGTTCA-CGCTCGGCTGTATTTATTAGATATCAAACCAATATTCCTTCGGGTCTATTGTGGTGAATCATAGTAACTTAACGAATC
TCGAACTTTCGAGATAAATCATTCAAGTTTCTGCCCTATCAGCTTTCGATGGTAGTGTATTGGACTACCATGGCAGTCACGGGTAACGG
AGAATTAGGGTTCGATTCCGGAGAGGGAGCCTGAGAAACGGCTACCACATCTAAGGAAGGCAGCAGGCGCGTAAATTACCCAATCCTGA
TTCAGGGAGGTAGTGACAAGAAATAACAACCTGGGGGC-TATTAGCCTTACGGGATTGCAATGAGAACAATCCAAAACTCTTAGCGAGG
AACAATTGGAGGGCAAGTCTGGTGCCAGCAGCCGCGGTAATTCCAGCTCCAATAGCGTATATTAAAGTTGTTGCAGTTAAAAAGCTCGT
AGTTGAATTTCTGGCTGTTTCTAGTCTTGGCCTCGGTCGAGCTAGGGGCAGTCATCCGTCTGCAAACCATGCTAGTCCTTTACTGGTCG
GTATGGGGAGTAGGCATTTTACTTTGAAAAAATTAGAGTGTTTCAGGCAGGCTTTTGCCCGAATACATTAGCATGGAATAATGGAATAG
GACTTAGTCCATTTTTGTTGGTTATTGGATTGAGTAATGATTAATAGGGACAGTTGGGGGCATTAGTATTTAATTGTCAGAGGTGAAAT
TCTTGGATTTATTAAAGACTAACTTATGCGAAAGCATTTGCCAAGGATGTTTTCATTAATCAAGAACGAAAGTTAGGGGATCAAAGACG
ATCAGATACCGTCCTAGTCTTAACTATAAACTATACCGACTCGGGATCGGCCGGGTA--TTTAAGCTCGGTCGGCACCGTATGAGAAAT
CAAAGTCTTTGGGTTCTGGGGGGAGTATGGTCGCAAGGCTGAAACTTAAAGGAATTGACGGAAGGGCACCACCAGGAGTGGAGCCTGCG
GCTTAATTTGACTCAACACGGGAAAACTTACCAGGTCAAAACATGGGTGAGATTGACAGATTGAGAGCTCTTTCTTGATTCTATGGGTG
GTGGTGCATGGCCGTTCTTAGTTGGTGGAGTGATTTGTCTGGTTAATTCCGTTAACGAACGAGACCTTAACCTGCTAACTAGTCATTTG
AACTTTATTCGAGTGTACTTCTTAGAGGGACTATGCTGCGGAAGCGCATGGAAGTTTGAGGCAATAACAGGTCTGTGATGCCCCTAGAC
GTCCTGGGCCGCACGCGCGCTACAATGACACGCTCAGCAAGTATTATCCTGACCCGGAAGGGCTTCGGTAATCTTCACAATACGTGTCG
TGTTAGGGATAGATCTTTGCAATTATGGATCTTGAACGAGGAATTCCTAGTAAGTGCAAGTCATTAGCTTGTACTGATTACGTCCCTGC
CCTTTGTACACACCGCCCGTCGCTCCTACCGATTTCGAGTGATCAGGTGAACATTCCGGACTGCAATTTCC-------CAATTGTAGAA
AGTTAAGTAAACCTTATCACTTAGAGGAAGGAAAAGTCGTAACAAGGTTTCCGTAGGTGAACCTGCAGAAGGATCA
**>Anoplophrya_octolasionis_MU_58_OL**
AACCTGGTTGATCCTGCCAGTAGTCATATGCTTGTCTCAAAGATTAAGCCATGCATGTCTAAGTATAAATAGTATACAGTGAAACTGCG
AATGGCTCATTATAACAGTTATAGTTTATTTGATAATTG-AAACCACATGGATAACCGTGGTAATTCTAGAGCTAATACATGCCTAAAA
GCCGGGTTCA-CGCTCGGCTGTATTTATTAGATATCAAACCAATATTCCTTCGGGTCTATTGTGGTGAATCATAGTAACTTAACGAATC
TCGAACTTTCGAGATAAATCATTCAAGTTTCTGCCCTATCAGCTTTCGATGGTAGTGTATTGGACTACCATGGCAGTCACGGGTAACGG
AGAATTAGGGTTCGATTCCGGAGAGGGAGCCTGAGAAACGGCTACCACATCTAAGGAAGGCAGCAGGCGCGTAAATTACCCAATCCTGA
TTCAGGGAGGTAGTGACAAGAAATAACAACCTGGGGGC-TATTAGCCTTACGGGATTGCAATGAGAACAATCCAAAACTCTTAGCGAGG
AACAATTGGAGGGCAAGTCTGGTGCCAGCAGCCGCGGTAATTCCAGCTCCAATAGCGTATATTAAAGTTGTTGCAGTTAAAAAGCTCGT
AGTTGAATTTCTGGCTGTTTCTAGTCTTGGCCTCGGTCGAGCTAGGGGCAGTCATCCGTCTGCAAACCATGCTAGTCCTTTACTGGTCG
GTATGGGGAGTAGGCATTTTACTTTGAAAAAATTAGAGTGTTTCAGGCAGGCTTTTGCCCGAATACATTAGCATGGAATAATGGAATAG
GACTTAGTCCATTTTTGTTGGTTATTGGATTGAGTAATGATTAATAGGGACAGTTGGGGGCATTAGTATTTAATTGTCAGAGGTGAAAT
TCTTGGATTTATTAAAGACTAACTTATGCGAAAGCATTTGCCAAGGATGTTTTCATTAATCAAGAACGAAAGTTAGGGGATCAAAGACG
ATCAGATACCGTCCTAGTCTTAACTATAAACTATACCGACTCGGGATCGGCCGGGTA--TTTAAGCTCGGTCGGCACCGTATGAGAAAT
CAAAGTCTTTGGGTTCTGGGGGGAGTATGGTCGCAAGGCTGAAACTTAAAGGAATTGACGGAAGGGCACCACCAGGAGTGGAGCCTGCG
GCTTAATTTGACTCAACACGGGAAAACTTACCAGGTCAAAACATGGGTGAGATTGACAGATTGAGAGCTCTTTCTTGATTCTATGGGTG
GTGGTGCATGGCCGTTCTTAGTTGGTGGAGTGATTTGTCTGGTTAATTCCGTTAACGAACGAGACCTTAACCTGCTAACTAGTCATTTG
AACTTTATTCGAGTGTACTTCTTAGAGGGACTATGCTGCGGAAGCGCATGGAAGTTTGAGGCAATAACAGGTCTGTGATGCCCCTAGAC
GTCCTGGGCCGCACGCGCGCTACAATGACACGCTCAGCAAGTATTATCCTGACCCGGAAGGGCTTCGGTAATCTTCACAATACGTGTCG
TGTTAGGGATAGATCTTTGCAATTATGGATCTTGAACGAGGAATTCCTAGTAAGTGCAAGTCATTAGCTTGTACTGATTACGTCCCTGC
CCTTTGTACACACCGCCCGTCGCTCCTACCGATTTCGAGTGATCAGGTGAACATTCCGGACTGCAATTTCC-------CAATTGTAGAA
AGTTAAGTAAACCTTATCACTTAGAGGAAGGAAAAGTCGTAACAAGGTTTCCGTAGGTGAACCTGCAGAAGGATCA

**>Anoplophrya_vulgaris_BZ_13_EF**
AACCTGGTTGATCCTGCCAGTAGTCATATGCTTGTCTCAAAGATTAAGCCATGCATGTCTAAGTATAAATAGTATACAGTGAAACTGCG
AATGGCTCATTATAACAGTTATAGTTTATTTGATAATTG-ACTCCACATGGATAACCGTGGTAATTCTAGAGCTAATACATGCCTAAAA
GCCGGGCT---CGCTCGGCTGTATTTATTAGATATCAAACCAATATTCCTTCGGGTCTATTGTGGTGAATCATAGTAACTTAACGAATC
TCGCATTTGCGAGATAAATCATTCAAGTTTCTGCCCTATCAGCTTTCGATGGTAGTGTATTGGACTACCATGGCAGTCACGGGTAACGG
AGAATTAGGGTTCGATTCCGGAGAGGGAGCCTGAGAAACGGCTACCACATCTAAGGAAGGCAGCAGGCGCGTAAATTACCCAATCCTGA
CTCAGGGAGGTAGTGACAAGAAATAACAACCTGGGGGC-TATTAGCCTTACGGGATTGCAATGAGAACAATCCAAAACTCTTAGCGAGG
AACAATTGGAGGGCAAGTCTGGTGCCAGCAGCCGCGGTAATTCCAGCTCCAATAGCGTATATTAAAGTTGTTGCAGTTAAAAAGCTCGT
AGTTGAATTTCTGGCTGTTCTTAGTCTTGGCCTCGGTCGAGCTAGGGGCAGTCATCCGTCTGCAAACCATGCTAGTCCTTTACTGGTCG
GTATGGGGAGTAGGCACTTTACTTTGAAAAAATTAGAGTGTTTCAGGCAGGCTTTTGCCCGAATACATTAGCATGGAATAATGGAATAG
GACTTAGTCCG-TTTTGTTGGTTATTGGATTGAGTAATGATTAATAGGGACAGTTGGGGGCATTAGTATTTAATTGTCAGAGGTGAAAT
TCTTGGATTTATTAAAGACTAACTTATGCGAAAGCATTTGCCAAGGATGTTTTCATTAATCAAGAACGAAAGTTAGGGGATCAAAGACG
ATCAGATACCGTCCTAGTCTTAACTATAAACTATACCGACTCGGGATCGGCCGGGTA--TCTAAGCTCGGTCGGCACCGTATGAGAAAT
CAAAGTCTTTGGGTTCTGGGGGGAGTATGGTCGCAAGGCTGAAACTTAAAGGAATTGACGGAAGGGCACCACCAGGAGTGGAGCCTGCG
GCTTAATTTGACTCAACACGGGAAAACTTACCAGGTCAAAACATGGGTGAGATTGACAGATTGAGAGCTCTTTCTTGATTCTATGGGTG
GTGGTGCATGGCCGTTCTTAGTTGGTGGAGTGATTTGTCTGGTTAATTCCGTTAACGAACGAGACCTTAACCTGCTAACTAGTCGTTTG
AATCTTATTCGAACGTACTTCTTAGAGGGACTATGCTGCGGAAGCGCATGGAAGTTTGAGGCAATAACAGGTCTGTGATGCCCCTAGAC
GTCCTGGGCCGCACGCGCGCTACAATGACACGCTCAGCAAGTATT-TCCTGCCCCGGAAGGGTTTCGGTAATCTTCACAATACGTGTCG
TGTTAGGGATAGATCTTTGCAATTATGGATCTTGAACGAGGAATTCCTAGTAAGTGCAAGTCATTAGCTTGTACTGATTACGTCCCTGC
CCTTTGTACACACCGCCCGTCGCTCCTACCGATTTCGAGTGGTCAGGTGAACATTCCGGACTGCGATTT-T-------TAATTGTAGAA
AGTTAAGTAAACCTTATCACTTAGAGGAAGGAAAAGTCGTAACAAGGTTTCCGTAGGTGAACCTGCAGAAGGATCA
**>Anoplophrya_vulgaris_JA1_18_EF**
AACCTGGTTGATCCTGCCAGTAGTCATATGCTTGTCTCAAAGATTAAGCCATGCATGTCTAAGTATAAATAGTATACAGTGAAACTGCG
AATGGCTCATTATAACAGTTATAGTTTATTTGATAATTG-ACTCCACATGGATAACCGTGGTAATTCTAGAGCTAATACATGCCTAAAA
GCCGGGCT---CGCTCGGCTGTATTTATTAGATATCAAACCAATATTCCTTCGGGTCTATTGTGGTGAATCATAGTAACTTAACGAATC
TCGCATTTGCGAGATAAATCATTCAAGTTTCTGCCCTATCAGCTTTCGATGGTAGTGTATTGGACTACCATGGCAGTCACGGGTAACGG
AGAATTAGGGTTCGATTCCGGAGAGGGAGCCTGAGAAACGGCTACCACATCTAAGGAAGGCAGCAGGCGCGTAAATTACCCAATCCTGA
CTCAGGGAGGTAGTGACAAGAAATAACAACCTGGGGGC-TATTAGCCTTACGGGATTGCAATGAGAACAATCCAAAACTCTTAGCGAGG
AACAATTGGAGGGCAAGTCTGGTGCCAGCAGCCGCGGTAATTCCAGCTCCAATAGCGTATATTAAAGTTGTTGCAGTTAAAAAGCTCGT
AGTTGAATTTCTGGCTGTTCTTAGTCTTGGCCTCGGTCGAGCTAGGGGCAGTCATCCGTCTGCAAACCATGCTAGTCCTTTACTGGTCG
GTATGGGGAGTAGGCACTTTACTTTGAAAAAATTAGAGTGTTTCAGGCAGGCTTTTGCCCGAATACATTAGCATGGAATAATGGAATAG
GACTTAGTCCG-TTTTGTTGGTTATTGGATTGAGTAATGATTAATAGGGACAGTTGGGGGCATTAGTATTTAATTGTCAGAGGTGAAAT
TCTTGGATTTATTAAAGACTAACTTATGCGAAAGCATTTGCCAAGGATGTTTTCATTAATCAAGAACGAAAGTTAGGGGATCAAAGACG
ATCAGATACCGTCCTAGTCTTAACTATAAACTATACCGACTCGGGATCGGCCGGGTA--TCTAAGCTCGGTCGGCACCGTATGAGAAAT
CAAAGTCTTTGGGTTCTGGGGGGAGTATGGTCGCAAGGCTGAAACTTAAAGGAATTGACGGAAGGGCACCACCAGGAGTGGAGCCTGCG
GCTTAATTTGACTCAACACGGGAAAACTTACCAGGTCAAAACATGGGTGAGATTGACAGATTGAGAGCTCTTTCTTGATTCTATGGGTG
GTGGTGCATGGCCGTTCTTAGTTGGTGGAGTGATTTGTCTGGTTAATTCCGTTAACGAACGAGACCTTAACCTGCTAACTAGTCGTTTG
AATCTTATTCGAACGTACTTCTTAGAGGGACTATGCTGCGGAAGCGCATGGAAGTTTGAGGCAATAACAGGTCTGTGATGCCCCTAGAC
GTCCTGGGCCGCACGCGCGCTACAATGACACGCTCAGCAAGTATT-TCCTGCCCCGGAAGGGTTTCGGTAATCTTCACAATACGTGTCG
TGTTAGGGATAGATCTTTGCAATTATGGATCTTGAACGAGGAATTCCTAGTAAGTGCAAGTCATTAGCTTGTACTGATTACGTCCCTGC
CCTTTGTACACACCGCCCGTCGCTCCTACCGATTTCGAGTGGTCAGGTGAACATTCCGGACTGCGATTT-T-------TAATTGTAGAA
AGTTAAGTAAACCTTATCACTTAGAGGAAGGAAAAGTCGTAACAAGGTTTCCGTAGGTGAACCTGCAGAAGGATCA
**>Anoplophrya_vulgaris_JA1_20_EF**
AACCTGGTTGATCCTGCCAGTAGTCATATGCTTGTCTCAAAGATTAAGCCATGCATGTCTAAGTATAAATAGTATACAGTGAAACTGCG
AATGGCTCATTATAACAGTTATAGTTTATTTGATAATTG-ACTCCACATGGATAACCGTGGTAATTCTAGAGCTAATACATGCCTAAAA
GCCGGGCT---CGCTCGGCTGTATTTATTAGATATCAAACCAATATTCCTTCGGGTCTATTGTGGTGAATCATAGTAACTTAACGAATC
TCGCATTTGCGAGATAAATCATTCAAGTTTCTGCCCTATCAGCTTTCGATGGTAGTGTATTGGACTACCATGGCAGTCACGGGTAACGG
AGAATTAGGGTTCGATTCCGGAGAGGGAGCCTGAGAAACGGCTACCACATCTAAGGAAGGCAGCAGGCGCGTAAATTACCCAATCCTGA
CTCAGGGAGGTAGTGACAAGAAATAACAACCTGGGGGC-TATTAGCCTTACGGGATTGCAATGAGAACAATCCAAAACTCTTAGCGAGG
AACAATTGGAGGGCAAGTCTGGTGCCAGCAGCCGCGGTAATTCCAGCTCCAATAGCGTATATTAAAGTTGTTGCAGTTAAAAAGCTCGT
AGTTGAATTTCTGGCTGTTCTTAGTCTTGGCCTCGGTCGAGCTAGGGGCAGTCATCCGTCTGCAAACCATGCTAGTCCTTTACTGGTCG
GTATGGGGAGTAGGCACTTTACTTTGAAAAAATTAGAGTGTTTCAGGCAGGCTTTTGCCCGAATACATTAGCATGGAATAATGGAATAG
GACTTAGTCCG-TTTTGTTGGTTATTGGATTGAGTAATGATTAATAGGGACAGTTGGGGGCATTAGTATTTAATTGTCAGAGGTGAAAT
TCTTGGATTTATTAAAGACTAACTTATGCGAAAGCATTTGCCAAGGATGTTTTCATTAATCAAGAACGAAAGTTAGGGGATCAAAGACG
ATCAGATACCGTCCTAGTCTTAACTATAAACTATACCGACTCGGGATCGGCCGGGTA--TCTAAGCTCGGTCGGCACCGTATGAGAAAT
CAAAGTCTTTGGGTTCTGGGGGGAGTATGGTCGCAAGGCTGAAACTTAAAGGAATTGACGGAAGGGCACCACCAGGAGTGGAGCCTGCG
GCTTAATTTGACTCAACACGGGAAAACTTACCAGGTCAAAACATGGGTGAGATTGACAGATTGAGAGCTCTTTCTTGATTCTATGGGTG
GTGGTGCATGGCCGTTCTTAGTTGGTGGAGTGATTTGTCTGGTTAATTCCGTTAACGAACGAGACCTTAACCTGCTAACTAGTCGTTTG
AATCTTATTCGAACGTACTTCTTAGAGGGACTATGCTGCGGAAGCGCATGGAAGTTTGAGGCAATAACAGGTCTGTGATGCCCCTAGAC
GTCCTGGGCCGCACGCGCGCTACAATGACACGCTCAGCAAGTATT-TCCTGCCCCGGAAGGGTTTCGGTAATCTTCACAATACGTGTCG
TGTTAGGGATAGATCTTTGCAATTATGGATCTTGAACGAGGAATTCCTAGTAAGTGCAAGTCATTAGCTTGTACTGATTACGTCCCTGC
CCTTTGTACACACCGCCCGTCGCTCCTACCGATTTCGAGTGGTCAGGTGAACATTCCGGACTGCGATTT-T-------TAATTGTAGAA
AGTTAAGTAAACCTTATCACTTAGAGGAAGGAAAAGTCGTAACAAGGTTTCCGTAGGTGAACCTGCAGAAGGATCA
**>Anoplophrya_vulgaris_JA1_21_EF**
AACCTGGTTGATCCTGCCAGTAGTCATATGCTTGTCTCAAAGATTAAGCCATGCATGTCTAAGTATAAATAGTATACAGTGAAACTGCG
AATGGCTCATTATAACAGTTATAGTTTATTTGATAATTG-ACTCCACATGGATAACCGTGGTAATTCTAGAGCTAATACATGCCTAAAA
GCCGGGCT---CGCTCGGCTGTATTTATTAGATATCAAACCAATATTCCTTCGGGTCTATTGTGGTGAATCATAGTAACTTAACGAATC
TCGCATTTGCGAGATAAATCATTCAAGTTTCTGCCCTATCAGCTTTCGATGGTAGTGTATTGGACTACCATGGCAGTCACGGGTAACGG
AGAATTAGGGTTCGATTCCGGAGAGGGAGCCTGAGAAACGGCTACCACATCTAAGGAAGGCAGCAGGCGCGTAAATTACCCAATCCTGA
CTCAGGGAGGTAGTGACAAGAAATAACAACCTGGGGGC-TATTAGCCTTACGGGATTGCAATGAGAACAATCCAAAACTCTTAGCGAGG
AACAATTGGAGGGCAAGTCTGGTGCCAGCAGCCGCGGTAATTCCAGCTCCAATAGCGTATATTAAAGTTGTTGCAGTTAAAAAGCTCGT

AGTTGAATTTCTGGCTGTTCTTAGTCTTGGCCTCGGTCGAGCTAGGGGCAGTCATCCGTCTGCAAACCATGCTAGTCCTTTACTGGTCG
GTATGGGGAGTAGGCACTTTACTTTGAAAAAATTAGAGTGTTTCAGGCAGGCTTTTGCCCGAATACATTAGCATGGAATAATGGAATAG
GACTTAGTCCG-TTTTGTTGGTTATTGGATTGAGTAATGATTAATAGGGACAGTTGGGGGCATTAGTATTTAATTGTCAGAGGTGAAAT
TCTTGGATTTATTAAAGACTAACTTATGCGAAAGCATTTGCCAAGGATGTTTTCATTAATCAAGAACGAAAGTTAGGGGATCAAAGACG
ATCAGATACCGTCCTAGTCTTAACTATAAACTATACCGACTCGGGATCGGCCGGGTA--TCTAAGCTCGGTCGGCACCGTATGAGAAAT
CAAAGTCTTTGGGTTCTGGGGGGAGTATGGTCGCAAGGCTGAAACTTAAAGGAATTGACGGAAGGGCACCACCAGGAGTGGAGCCTGCG
GCTTAATTTGACTCAACACGGGAAAACTTACCAGGTCAAAACATGGGTGAGATTGACAGATTGAGAGCTCTTTCTTGATTCTATGGGTG
GTGGTGCATGGCCGTTCTTAGTTGGTGGAGTGATTTGTCTGGTTAATTCCGTTAACGAACGAGACCTTAACCTGCTAACTAGTCGTTTG
AATCTTATTCGAACGTACTTCTTAGAGGGACTATGCTGCGGAAGCGCATGGAAGTTTGAGGCAATAACAGGTCTGTGATGCCCCTAGAC
GTCCTGGGCCGCACGCGCGCTACAATGACACGCTCAGCAAGTATT-TCCTGCCCCGGAAGGGTTTCGGTAATCTTCACAATACGTGTCG
TGTTAGGGATAGATCTTTGCAATTATGGATCTTGAACGAGGAATTCCTAGTAAGTGCAAGTCATTAGCTTGTACTGATTACGTCCCTGC
CCTTTGTACACACCGCCCGTCGCTCCTACCGATTTCGAGTGGTCAGGTGAACATTCCGGACTGCGATTT-T-------TAATTGTAGAA
AGTTAAGTAAACCTTATCACTTAGAGGAAGGAAAAGTCGTAACAAGGTTTCCGTAGGTGAACCTGCAGAAGGATCA
**>Anoplophrya_vulgaris_NG_27_DV**
AACCTGGTTGATCCTGCCAGTAGTCATATGCTTGTCTCAAAGATTAAGCCATGCATGTCTAAGTATAAATAGTATACAGTGAAACTGCG
AATGGCTCATTATAACAGTTATAGTTTATTTGATAATTG-ACTCCACATGGATAACCGTGGTAATTCTAGAGCTAATACATGCCTAAAA
GCCGGGCT---CGCTCGGCTGTATTTATTAGATATCAAACCAATATTCCTTCGGGTCTATTGTGGTGAATCATAGTAACTTAACGAATC
TCGCATTTGCGAGATAAATCATTCAAGTTTCTGCCCTATCAGCTTTCGATGGTAGTGTATTGGACTACCATGGCAGTCACGGGTAACGG
AGAATTAGGGTTCGATTCCGGAGAGGGAGCCTGAGAAACGGCTACCACATCTAAGGAAGGCAGCAGGCGCGTAAATTACCCAATCCTGA
CTCAGGGAGGTAGTGACAAGAAATAACAACCTGGGGGC-TATTAGCCTTACGGGATTGCAATGAGAACAATCCAAAACTCTTAGCGAGG
AACAATTGGAGGGCAAGTCTGGTGCCAGCAGCCGCGGTAATTCCAGCTCCAATAGCGTATATTAAAGTTGTTGCAGTTAAAAAGCTCGT
AGTTGAATTTCTGGCTGTTCTTAGTCTTGGCCTCGGTCGAGCTAGGGGCAGTCATCCGTCTGCAAACCATGCTAGTCCTTTACTGGTCG
GTATGGGGAGTAGGCACTTTACTTTGAAAAAATTAGAGTGTTTCAGGCAGGCTTTTGCCCGAATACATTAGCATGGAATAATGGAATAG
GACTTAGTCCG-TTTTGTTGGTTATTGGATTGAGTAATGATTAATAGGGACAGTTGGGGGCATTAGTATTTAATTGTCAGAGGTGAAAT
TCTTGGATTTATTAAAGACTAACTTATGCGAAAGCATTTGCCAAGGATGTTTTCATTAATCAAGAACGAAAGTTAGGGGATCAAAGACG
ATCAGATACCGTCCTAGTCTTAACTATAAACTATACCGACTCGGGATCGGCCGGGTA--TCTAAGCTCGGTCGGCACCGTATGAGAAAT
CAAAGTCTTTGGGTTCTGGGGGGAGTATGGTCGCAAGGCTGAAACTTAAAGGAATTGACGGAAGGGCACCACCAGGAGTGGAGCCTGCG
GCTTAATTTGACTCAACACGGGAAAACTTACCAGGTCAAAACATGGGTGAGATTGACAGATTGAGAGCTCTTTCTTGATTCTATGGGTG
GTGGTGCATGGCCGTTCTTAGTTGGTGGAGTGATTTGTCTGGTTAATTCCGTTAACGAACGAGACCTTAACCTGCTAACTAGTCGTTTG
AATCTTATTCGAACGTACTTCTTAGAGGGACTATGCTGCGGAAGCGCATGGAAGTTTGAGGCAATAACAGGTCTGTGATGCCCCTAGAC
GTCCTGGGCCGCACGCGCGCTACAATGACACGCTCAGCAAGTATT-TCCTGCCCCGGAAGGGTTTCGGTAATCTTCACAATACGTGTCG
TGTTAGGGATAGATCTTTGCAATTATGGATCTTGAACGAGGAATTCCTAGTAAGTGCAAGTCATTAGCTTGTACTGATTACGTCCCTGC
CCTTTGTACACACCGCCCGTCGCTCCTACCGATTTCGAGTGGTCAGGTGAACATTCCGGACTGCGATTT-T-------TAATTGTAGAA
AGTTAAGTAAACCTTATCACTTAGAGGAAGGAAAAGTCGTAACAAGGTTTCCGTAGGTGAACCTGCAGAAGGATCA
**>Anoplophrya_vulgaris_NG_28_DV**
AACCTGGTTGATCCTGCCAGTAGTCATATGCTTGTCTCAAAGATTAAGCCATGCATGTCTAAGTATAAATAGTATACAGTGAAACTGCG
AATGGCTCATTATAACAGTTATAGTTTATTTGATAATTG-ACTCCACATGGATAACCGTGGTAATTCTAGAGCTAATACATGCCTAAAA
GCCGGGCT---CGCTCGGCTGTATTTATTAGATATCAAACCAATATTCCTTCGGGTCTATTGTGGTGAATCATAGTAACTTAACGAATC
TCGCATTTGCGAGATAAATCATTCAAGTTTCTGCCCTATCAGCTTTCGATGGTAGTGTATTGGACTACCATGGCAGTCACGGGTAACGG
AGAATTAGGGTTCGATTCCGGAGAGGGAGCCTGAGAAACGGCTACCACATCTAAGGAAGGCAGCAGGCGCGTAAATTACCCAATCCTGA
CTCAGGGAGGTAGTGACAAGAAATAACAACCTGGGGGC-TATTAGCCTTACGGGATTGCAATGAGAACAATCCAAAACTCTTAGCGAGG
AACAATTGGAGGGCAAGTCTGGTGCCAGCAGCCGCGGTAATTCCAGCTCCAATAGCGTATATTAAAGTTGTTGCAGTTAAAAAGCTCGT
AGTTGAATTTCTGGCTGTTCTTAGTCTTGGCCTCGGTCGAGCTAGGGGCAGTCATCCGTCTGCAAACCATGCTAGTCCTTTACTGGTCG
GTATGGGGAGTAGGCACTTTACTTTGAAAAAATTAGAGTGTTTCAGGCAGGCTTTTGCCCGAATACATTAGCATGGAATAATGGAATAG
GACTTAGTCCG-TTTTGTTGGTTATTGGATTGAGTAATGATTAATAGGGACAGTTGGGGGCATTAGTATTTAATTGTCAGAGGTGAAAT
TCTTGGATTTATTAAAGACTAACTTATGCGAAAGCATTTGCCAAGGATGTTTTCATTAATCAAGAACGAAAGTTAGGGGATCAAAGACG
ATCAGATACCGTCCTAGTCTTAACTATAAACTATACCGACTCGGGATCGGCCGGGTA--TCTAAGCTCGGTCGGCACCGTATGAGAAAT
CAAAGTCTTTGGGTTCTGGGGGGAGTATGGTCGCAAGGCTGAAACTTAAAGGAATTGACGGAAGGGCACCACCAGGAGTGGAGCCTGCG
GCTTAATTTGACTCAACACGGGAAAACTTACCAGGTCAAAACATGGGTGAGATTGACAGATTGAGAGCTCTTTCTTGATTCTATGGGTG
GTGGTGCATGGCCGTTCTTAGTTGGTGGAGTGATTTGTCTGGTTAATTCCGTTAACGAACGAGACCTTAACCTGCTAACTAGTCGTTTG
AATCTTATTCGAACGTACTTCTTAGAGGGACTATGCTGCGGAAGCGCATGGAAGTTTGAGGCAATAACAGGTCTGTGATGCCCCTAGAC
GTCCTGGGCCGCACGCGCGCTACAATGACACGCTCAGCAAGTATT-TCCTGCCCCGGAAGGGTTTCGGTAATCTTCACAATACGTGTCG
TGTTAGGGATAGATCTTTGCAATTATGGATCTTGAACGAGGAATTCCTAGTAAGTGCAAGTCATTAGCTTGTACTGATTACGTCCCTGC
CCTTTGTACACACCGCCCGTCGCTCCTACCGATTTCGAGTGGTCAGGTGAACATTCCGGACTGCGATTT-T-------TAATTGTAGAA
AGTTAAGTAAACCTTATCACTTAGAGGAAGGAAAAGTCGTAACAAGGTTTCCGTAGGTGAACCTGCAGAAGGATCA
**>Maupasella_mucronata_KDo_33_ET**
AACCTGGTTGATCCTGCCAGTAGTCATATGCTTGTCTCAAAGATTAAGCCATGCATGTCTAAGTATAAATAGTATACAGTGAAACTGCG
AATGGCTCATTACAACAGGTATAGTTTATTTGATAATCGCACGCTACATGGATAACCGTGGTAATTCTAGAGCTAATACATGCTGTTAA
ACCCGACTTCTGGAAGGGTTGTATTTATTAGATATCAAACCAATGGGCTTC---GGCCGTTGCGGTGAGTCATAGTAACTGATCGAATC
TCGAGCTTTCGAGATAAATCATTCAAGTTTCTGCCCTATCAGCTTCCGATGGTAGTGTATTGGACTACCATGGCAGTCACGGGTAACGG
AGAATTAGGGTTCGATTCCGGAGAGGGAGCCTGAGAAACGGCTACCACATCTAAGGAAGGCAGCAGGCGCGTAAATTACCCAATCCTGA
CTCAGGGAGGTAGTGACAAGAAATAACAAACCGGGGGC-TTCGCGCCTTACGGTATTGCAATGAGAACAATTTAAAACTCTTAGCGAGG
AACAATTGGAGGGCAAGTCTGGTGCCAGCAGCCGCGGTAATTCCAGCTCCAATAGCGTATATTAAAGTTGTTGCAGTTAAAAAGCTCGT
AGTTGAATTTCTGGCTGTTCGC-TTGCTGGCTTCGGTCGGGAAGTGGGCAGTCATCCGTTTACAAACCATATTGGGCCTTAACTGGTCG
GTATGGCGAGTAAACATTTTACTTTGAAAAAATCAGAGTGTTTCAGGCAGGCTTTTGCCAGAATACATTAGCATGGAATAATGGAATAG
GACTTAGTCCA-TTTTGTTGGTTATTGGATTAAGTAATGATTAATAGGGACAGTTGGGGGCATTAGTATTTAATTGTCAGAGGTGAAAT
TCTTGGATTTATTAAAGACTAACTTAGGCGAAAGCATTTGCCAAGGATGTTTTCATTAATCAAGAACGAAAGTTAGGGGATCAAAGACG
ATCAGATACCGTCCTAGTCTTAACTATAAACTATACCGACTCGGGATCGGCCGGGAT--TTCTAGCCCGGTCGGCACCGTATGAGAAAT
CAAAGTCTTTGGGTTCTGGGGGGAGTATGGTCGCAAGGCTGAAACTTAAAGGAATTGACGGAAGGGCACCACCAGGAGTGGAGCCTGCG
GCTTAATTTGACTCAACACGGGAAAACTCACCAGGGCAAAACACAGGTGGGATTGACAGATTGAGAGCTCTTTCTTGATTCTGTGGGTG
GTGGTGCATGGCCGTTCTTAGTTGGTGGAGTGATTTGTCTGGTTAATTCCGTTAACGAACGAGACCTTAACCTGCTAACTAGTCATCGG

AACCACATTCTGATGTACTTCTTAGAGGGACTATGC-GCACAAACGCATGGAAGTTTGAGGCAATAACAGGTCTGTGATGCCCCTAGAC
GTCCTGGGCCGCACGCGCGCTACAATGACACGCTCAACAAGTCTAACCTGGCCCGGAAGGGAGTCGGGTAATCTTTTTAATACGTGTCG
TGTTAGGGATAGATCTTTGTAATTATAGATCTTGAACGAGGAATTCCTAGTAAGTGCAAGTCATTAGCTTGTACTGATTACGTCCCTGC
CCTTTGTACACACCGCCCGTCGCTCCTACCGATTTCGAGTGATAAGGTGAACCCTCCAGACCGTGGTGT-CCTCGAGACTACTGCGGAA
AGTTGAGTAAACCTTGTCACTTAGAGGAAGGAAAAGTCGTAACAAGGTTTCCGTAGGTGAACCTGCAGAAGGATCA
**>Maupasella_mucronata_KDo_34_ET**
AACCTGGTTGATCCTGCCAGTAGTCATATGCTTGTCTCAAAGATTAAGCCATGCATGTCTAAGTATAAATAGTATACAGTGAAACTGCG
AATGGCTCATTACAACAGGTATAGTTTATTTGATAATCGCACGCTACATGGATAACCGTGGTAATTCTAGAGCTAATACATGCTGTTAA
ACCCGACTTCTGGAAGGGTTGTATTTATTAGATATCAAACCAATGGGCTTC---GGCCGTTGCGGTGAGTCATAGTAACTGATCGAATC
TCGAGCTTTCGAGATAAATCATTCAAGTTTCTGCCCTATCAGCTTCCGATGGTAGTGTATTGGACTACCATGGCAGTCACGGGTAACGG
AGAATTAGGGTTCGATTCCGGAGAGGGAGCCTGAGAAACGGCTACCACATCTAAGGAAGGCAGCAGGCGCGTAAATTACCCAATCCTGA
CTCAGGGAGGTAGTGACAAGAAATAACAAACCGGGGGC-TTCGCGCCTTACGGTATTGCAATGAGAACAATTTAAAACTCTTAGCGAGG
AACAATTGGAGGGCAAGTCTGGTGCCAGCAGCCGCGGTAATTCCAGCTCCAATAGCGTATATTAAAGTTGTTGCAGTTAAAAAGCTCGT
AGTTGAATTTCTGGCTGTTCGC-TTGCTGGCTTCGGTCGGGAAGTGGGCAGTCATCCGTTTACAAACCATATTGGGCCTTAACTGGTCG
GTATGGCGAGTAAACATTTTACTTTGAAAAAATCAGAGTGTTTCAGGCAGGCTTTTGCCAGAATACATTAGCATGGAATAATGGAATAG
GACTTAGTCCA-TTTTGTTGGTTATTGGATTAAGTAATGATTAATAGGGACAGTTGGGGGCATTAGTATTTAATTGTCAGAGGTGAAAT
TCTTGGATTTATTAAAGACTAACTTAGGCGAAAGCATTTGCCAAGGATGTTTTCATTAATCAAGAACGAAAGTTAGGGGATCAAAGACG
ATCAGATACCGTCCTAGTCTTAACTATAAACTATACCGACTCGGGATCGGCCGGGAT--TTCTAGCCCGGTCGGCACCGTATGAGAAAT
CAAAGTCTTTGGGTTCTGGGGGGAGTATGGTCGCAAGGCTGAAACTTAAAGGAATTGACGGAAGGGCACCACCAGGAGTGGAGCCTGCG
GCTTAATTTGACTCAACACGGGAAAACTCACCAGGGCAAAACACAGGTGGGATTGACAGATTGAGAGCTCTTTCTTGATTCTGTGGGTG
GTGGTGCATGGCCGTTCTTAGTTGGTGGAGTGATTTGTCTGGTTAATTCCGTTAACGAACGAGACCTTAACCTGCTAACTAGTCATCGG
AACCACATTCTGATGTACTTCTTAGAGGGACTATGC-GCACAAACGCATGGAAGTTTGAGGCAATAACAGGTCTGTGATGCCCCTAGAC
GTCCTGGGCCGCACGCGCGCTACAATGACACGCTCAACAAGTCTAACCTGGCCCGGAAGGGAGTCGGGTAATCTTTTTAATACGTGTCG
TGTTAGGGATAGATCTTTGTAATTATAGATCTTGAACGAGGAATTCCTAGTAAGTGCAAGTCATTAGCTTGTACTGATTACGTCCCTGC
CCTTTGTACACACCGCCCGTCGCTCCTACCGATTTCGAGTGATAAGGTGAACCCTCCAGACCGTGGTGT-CCTCGAGACTACTGCGGAA
AGTTGAGTAAACCTTGTCACTTAGAGGAAGGAAAAGTCGTAACAAGGTTTCCGTAGGTGAACCTGCAGAAGGATCA
**>Maupasella_mucronata_KDo_35_ET**
AACCTGGTTGATCCTGCCAGTAGTCATATGCTTGTCTCAAAGATTAAGCCATGCATGTCTAAGTATAAATAGTATACAGTGAAACTGCG
AATGGCTCATTACAACAGGTATAGTTTATTTGATAATCGCACGCTACATGGATAACCGTGGTAATTCTAGAGCTAATACATGCTGTTAA
ACCCGACTTCTGGAAGGGTTGTATTTATTAGATATCAAACCAATGGGCTTC---GGCCGTTGCGGTGAGTCATAGTAACTGATCGAATC
TCGAGCTTTCGAGATAAATCATTCAAGTTTCTGCCCTATCAGCTTCCGATGGTAGTGTATTGGACTACCATGGCAGTCACGGGTAACGG
AGAATTAGGGTTCGATTCCGGAGAGGGAGCCTGAGAAACGGCTACCACATCTAAGGAAGGCAGCAGGCGCGTAAATTACCCAATCCTGA
CTCAGGGAGGTAGTGACAAGAAATAACAAACCGGGGGC-TTCGCGCCTTACGGTATTGCAATGAGAACAATTTAAAACTCTTAGCGAGG
AACAATTGGAGGGCAAGTCTGGTGCCAGCAGCCGCGGTAATTCCAGCTCCAATAGCGTATATTAAAGTTGTTGCAGTTAAAAAGCTCGT
AGTTGAATTTCTGGCTGTTCGC-TTGCTGGCTTCGGTCGGGAAGTGGGCAGTCATCCGTTTACAAACCATATTGGGCCTTAACTGGTCG
GTATGGCGAGTAAACATTTTACTTTGAAAAAATCAGAGTGTTTCAGGCAGGCTTTTGCCAGAATACATTAGCATGGAATAATGGAATAG
GACTTAGTCCA-TTTTGTTGGTTATTGGATTAAGTAATGATTAATAGGGACAGTTGGGGGCATTAGTATTTAATTGTCAGAGGTGAAAT
TCTTGGATTTATTAAAGACTAACTTAGGCGAAAGCATTTGCCAAGGATGTTTTCATTAATCAAGAACGAAAGTTAGGGGATCAAAGACG
ATCAGATACCGTCCTAGTCTTAACTATAAACTATACCGACTCGGGATCGGCCGGGAT--TTCTAGCCCGGTCGGCACCGTATGAGAAAT
CAAAGTCTTTGGGTTCTGGGGGGAGTATGGTCGCAAGGCTGAAACTTAAAGGAATTGACGGAAGGGCACCACCAGGAGTGGAGCCTGCG
GCTTAATTTGACTCAACACGGGAAAACTCACCAGGGCAAAACACAGGTGGGATTGACAGATTGAGAGCTCTTTCTTGATTCTGTGGGTG
GTGGTGCATGGCCGTTCTTAGTTGGTGGAGTGATTTGTCTGGTTAATTCCGTTAACGAACGAGACCTTAACCTGCTAACTAGTCATCGG
AACCACATTCTGATGTACTTCTTAGAGGGACTATGC-GCACAAACGCATGGAAGTTTGAGGCAATAACAGGTCTGTGATGCCCCTAGAC
GTCCTGGGCCGCACGCGCGCTACAATGACACGCTCAACAAGTCTAACCTGGCCCGGAAGGGAGTCGGGTAATCTTTTTAATACGTGTCG
TGTTAGGGATAGATCTTTGTAATTATAGATCTTGAACGAGGAATTCCTAGTAAGTGCAAGTCATTAGCTTGTACTGATTACGTCCCTGC
CCTTTGTACACACCGCCCGTCGCTCCTACCGATTTCGAGTGATAAGGTGAACCCTCCAGACCGTGGTGT-CCTCGAGACTACTGCGGAA
AGTTGAGTAAACCTTGTCACTTAGAGGAAGGAAAAGTCGTAACAAGGTTTCCGTAGGTGAACCTGCAGAAGGATCA
**>Maupasella_mucronata_KDo_36_ET**
AACCTGGTTGATCCTGCCAGTAGTCATATGCTTGTCTCAAAGATTAAGCCATGCATGTCTAAGTATAAATAGTATACAGTGAAACTGCG
AATGGCTCATTACAACAGGTATAGTTTATTTGATAATCGCACGCTACATGGATAACCGTGGTAATTCTAGAGCTAATACATGCTGTTAA
ACCCGACTTCTGGAAGGGTTGTATTTATTAGATATCAAACCAATGGGCTTC---GGCCGTTGCGGTGAGTCATAGTAACTGATCGAATC
TCGAGCTTTCGAGATAAATCATTCAAGTTTCTGCCCTATCAGCTTCCGATGGTAGTGTATTGGACTACCATGGCAGTCACGGGTAACGG
AGAATTAGGGTTCGATTCCGGAGAGGGAGCCTGAGAAACGGCTACCACATCTAAGGAAGGCAGCAGGCGCGTAAATTACCCAATCCTGA
CTCAGGGAGGTAGTGACAAGAAATAACAAACCGGGGGC-TTCGCGCCTTACGGTATTGCAATGAGAACAATTTAAAACTCTTAGCGAGG
AACAATTGGAGGGCAAGTCTGGTGCCAGCAGCCGCGGTAATTCCAGCTCCAATAGCGTATATTAAAGTTGTTGCAGTTAAAAAGCTCGT
AGTTGAATTTCTGGCTGTTCGC-TTGCTGGCTTCGGTCGGGAAGTGGGCAGTCATCCGTTTACAAACCATATTGGGCCTTAACTGGTCG
GTATGGCGAGTAAACATTTTACTTTGAAAAAATCAGAGTGTTTCAGGCAGGCTTTTGCCAGAATACATTAGCATGGAATAATGGAATAG
GACTTAGTCCA-TTTTGTTGGTTATTGGATTAAGTAATGATTAATAGGGACAGTTGGGGGCATTAGTATTTAATTGTCAGAGGTGAAAT
TCTTGGATTTATTAAAGACTAACTTAGGCGAAAGCATTTGCCAAGGATGTTTTCATTAATCAAGAACGAAAGTTAGGGGATCAAAGACG
ATCAGATACCGTCCTAGTCTTAACTATAAACTATACCGACTCGGGATCGGCCGGGAT--TTCTAGCCCGGTCGGCACCGTATGAGAAAT
CAAAGTCTTTGGGTTCTGGGGGGAGTATGGTCGCAAGGCTGAAACTTAAAGGAATTGACGGAAGGGCACCACCAGGAGTGGAGCCTGCG
GCTTAATTTGACTCAACACGGGAAAACTCACCAGGGCAAAACACAGGTGGGATTGACAGATTGAGAGCTCTTTCTTGATTCTGTGGGTG
GTGGTGCATGGCCGTTCTTAGTTGGTGGAGTGATTTGTCTGGTTAATTCCGTTAACGAACGAGACCTTAACCTGCTAACTAGTCATCGG
AACCACATTCTGATGTACTTCTTAGAGGGACTATGC-GCACAAACGCATGGAAGTTTGAGGCAATAACAGGTCTGTGATGCCCCTAGAC
GTCCTGGGCCGCACGCGCGCTACAATGACACGCTCAACAAGTCTAACCTGGCCCGGAAGGGAGTCGGGTAATCTTTTTAATACGTGTCG
TGTTAGGGATAGATCTTTGTAATTATAGATCTTGAACGAGGAATTCCTAGTAAGTGCAAGTCATTAGCTTGTACTGATTACGTCCCTGC
CCTTTGTACACACCGCCCGTCGCTCCTACCGATTTCGAGTGATAAGGTGAACCCTCCAGACCGTGGTGT-CCTCGAGACTACTGCGGAA
AGTTGAGTAAACCTTGTCACTTAGAGGAAGGAAAAGTCGTAACAAGGTTTCCGTAGGTGAACCTGCAGAAGGATCA
**>Metaradiophrya_chlorotica_JA2_1M_ACH**
AACCTGGTTGATCCTGCCAGTAGTCATATGCTTGTCTCAAAGATTAAGCCATGCATGTCTAAGTATAAATAGTATACAGTGAAACTGCG
AATGGCTCATTATAACAGTTATAGTTTATTTGATAATTGCAAGCTACATGGATAACCGTGGTAATTCTAGAGCTAATACATGCTGCAAA

ACCCGACTTC-GGAAGGGTTGTATTTATTAGATATCAAACCAATATTCCTTCGGGGATATTGTGGTGAATCATAGTAACTGATCGAATC
TCGAACTATCGAGATACATCATTCAAGTTTCTGCCCTATCAGCTTTCGATGGTAGTGTATTGGACTACCATGGCAGTCACGGGTAACGG
AGAATTAGGGTTCGATTCCGGAGAGGGAGCCTGAGAAACGGCTACCACATCTAAGGAAGGCAGCAGGCGCGTAAATTACCCAATCCTGA
CTCAGGGAGGTAGTGACAAGAAATAACAACCTGGGGGC-TATTAGCCTTACGGGATTGCAATGAGAACAATCCAAAACTCTTAGCGAGG
AACAATTGGAGGGCAAGTCTGGTGCCAGCAGCCGCGGTAATTCCAGCTCCAATAGCGTATATTAAAGTTGTTGCAGTTAAAAAGCTCGT
AGTTGAATTTCTGGCCGTTCTTAGTCTTGGCCTCGGTCGAGCTAAGGGCAGTCATCCGTCTGCAAACCATGCTAGTCCTTCACTGGTCG
GTATGGGGAGTAGGCACTTTACTTTGAAAAAATTAGAGTGTTTCAGGCAGGCCTTTGCCCGAATACATTAGCATGGAATAATGGAATAG
GACTTAGTCCA-TTTTGTTGGTTATTGGATTGAGTAATGATTAATAGGGACAGTTGGGGGCATTAGTATTTAATTGTCAGAGGTGAAAT
TCTTGGATTTATTAAAGACTAACTTATGCGAAAGCATTTGCCAAGGATGTTTTCATTAATCAAGAACGAAAGTTAGGGGATCAAAGACG
ATCAGATACCGTCCTAGTCTTAACTATAAACTATACCGACTCGGGATCGGCCGGGTGA-TTTAAGCTCGGTCGGCACCGTATGAGAAAT
CAAAGTCTTTGGGTTCTGGGGGGAGTATGGTCGCAAGGCTGAAACTTAAAGGAATTGACGGAAGGGCACCACCAGGAGTGGAGCCTGCG
GCTTAATTTGACTCAACACGGGAAAACTTACCAGGTCAAAACATGGGTGAGATTGACAGATTGAGAGCTCTTTCTTGATTCTATGGGTG
GTGGTGCATGGCCGTTCTTAGTTGGTGGAGTGATTTGTCTGGTTAATTCCGTTAACGAACGAGACCTTAACCTGCTAATTAGTCAATTG
AATCACATTCAGTTGTACTTCTTAGAGGGACTATGCAGCGGAAGCGCATGGAAGTTTGAGGCAATAACAGGTCTGTGATGCCCCTAGAC
GTCCTGGGCCGCACGCGCGCTACAATGACACGCTCAGCAAGTATTATCCTGACCCGGAAGGGCCCCGGTAATCTTCACAATACGTGTCG
TGTTAGGGATAGATCTTTGCAATTATGGATCTTGAACGAGGAATTCCTAGTAAGTGCAAGTCATTAGCTTGTACTGATTACGTCCCTGC
CCTTTGTACACACCGCCCGTCGCTCCTACCGATTTCGAGTGATCAGGTGAACATTCCGGACTGCATTAG-CCCTATGACTAGTGTAGGA
AGTTAAGTAAACCTTATCACTTAGAGGAAGGAAAAGTCGTAACAAGGTTTCCGTAGGTGAACCTGCAGAAGGATCA
**>Metaradiophrya_chlorotica_JA2_2M_ACH**
AACCTGGTTGATCCTGCCAGTAGTCATATGCTTGTCTCAAAGATTAAGCCATGCATGTCTAAGTATAAATAGTATACAGTGAAACTGCG
AATGGCTCATTATAACAGTTATAGTTTATTTGATAATTGCAAGCTACATGGATAACCGTGGTAATTCTAGAGCTAATACATGCTGCAAA
ACCCGACTTC-GGAAGGGTTGTATTTATTAGATATCAAACCAATATTCCTTCGGGGATATTGTGGTGAATCATAGTAACTGATCGAATC
TCGAACTATCGAGATACATCATTCAAGTTTCTGCCCTATCAGCTTTCGATGGTAGTGTATTGGACTACCATGGCAGTCACGGGTAACGG
AGAATTAGGGTTCGATTCCGGAGAGGGAGCCTGAGAAACGGCTACCACATCTAAGGAAGGCAGCAGGCGCGTAAATTACCCAATCCTGA
CTCAGGGAGGTAGTGACAAGAAATAACAACCTGGGGGC-TATTAGCCTTACGGGATTGCAATGAGAACAATCCAAAACTCTTAGCGAGG
AACAATTGGAGGGCAAGTCTGGTGCCAGCAGCCGCGGTAATTCCAGCTCCAATAGCGTATATTAAAGTTGTTGCAGTTAAAAAGCTCGT
AGTTGAATTTCTGGCCGTTCTTAGTCTTGGCCTCGGTCGAGCTAAGGGCAGTCATCCGTCTGCAAACCATGCTAGTCCTTCACTGGTCG
GTATGGGGAGTAGGCACTTTACTTTGAAAAAATTAGAGTGTTTCAGGCAGGCCTTTGCCCGAATACATTAGCATGGAATAATGGAATAG
GACTTAGTCCA-TTTTGTTGGTTATTGGATTGAGTAATGATTAATAGGGACAGTTGGGGGCATTAGTATTTAATTGTCAGAGGTGAAAT
TCTTGGATTTATTAAAGACTAACTTATGCGAAAGCATTTGCCAAGGATGTTTTCATTAATCAAGAACGAAAGTTAGGGGATCAAAGACG
ATCAGATACCGTCCTAGTCTTAACTATAAACTATACCGACTCGGGATCGGCCGGGTGA-TTTAAGCTCGGTCGGCACCGTATGAGAAAT
CAAAGTCTTTGGGTTCTGGGGGGAGTATGGTCGCAAGGCTGAAACTTAAAGGAATTGACGGAAGGGCACCACCAGGAGTGGAGCCTGCG
GCTTAATTTGACTCAACACGGGAAAACTTACCAGGTCAAAACATGGGTGAGATTGACAGATTGAGAGCTCTTTCTTGATTCTATGGGTG
GTGGTGCATGGCCGTTCTTAGTTGGTGGAGTGATTTGTCTGGTTAATTCCGTTAACGAACGAGACCTTAACCTGCTAATTAGTCAATTG
AATCACATTCAGTTGTACTTCTTAGAGGGACTATGCAGCGGAAGCGCATGGAAGTTTGAGGCAATAACAGGTCTGTGATGCCCCTAGAC
GTCCTGGGCCGCACGCGCGCTACAATGACACGCTCAGCAAGTATTATCCTGACCCGGAAGGGCCCCGGTAATCTTCACAATACGTGTCG
TGTTAGGGATAGATCTTTGCAATTATGGATCTTGAACGAGGAATTCCTAGTAAGTGCAAGTCATTAGCTTGTACTGATTACGTCCCTGC
CCTTTGTACACACCGCCCGTCGCTCCTACCGATTTCGAGTGATCAGGTGAACATTCCGGACTGCATTAG-CCCTATGACTAGTGTAGGA
AGTTAAGTAAACCTTATCACTTAGAGGAAGGAAAAGTCGTAACAAGGTTTCCGTAGGTGAACCTGCAGAAGGATCA
**>Metaradiophrya_chlorotica_JA2_3M_ACH**
AACCTGGTTGATCCTGCCAGTAGTCATATGCTTGTCTCAAAGATTAAGCCATGCATGTCTAAGTATAAATAGTATACAGTGAAACTGCG
AATGGCTCATTATAACAGTTATAGTTTATTTGATAATTGCAAGCTACATGGATAACCGTGGTAATTCTAGAGCTAATACATGCTGCAAA
ACCCGACTTC-GGAAGGGTTGTATTTATTAGATATCAAACCAATATTCCTTCGGGGATATTGTGGTGAATCATAGTAACTGATCGAATC
TCGAACTATCGAGATACATCATTCAAGTTTCTGCCCTATCAGCTTTCGATGGTAGTGTATTGGACTACCATGGCAGTCACGGGTAACGG
AGAATTAGGGTTCGATTCCGGAGAGGGAGCCTGAGAAACGGCTACCACATCTAAGGAAGGCAGCAGGCGCGTAAATTACCCAATCCTGA
CTCAGGGAGGTAGTGACAAGAAATAACAACCTGGGGGC-TATTAGCCTTACGGGATTGCAATGAGAACAATCCAAAACTCTTAGCGAGG
AACAATTGGAGGGCAAGTCTGGTGCCAGCAGCCGCGGTAATTCCAGCTCCAATAGCGTATATTAAAGTTGTTGCAGTTAAAAAGCTCGT
AGTTGAATTTCTGGCCGTTCTTAGTCTTGGCCTCGGTCGAGCTAAGGGCAGTCATCCGTCTGCAAACCATGCTAGTCCTTCACTGGTCG
GTATGGGGAGTAGGCACTTTACTTTGAAAAAATTAGAGTGTTTCAGGCAGGCCTTTGCCCGAATACATTAGCATGGAATAATGGAATAG
GACTTAGTCCA-TTTTGTTGGTTATTGGATTGAGTAATGATTAATAGGGACAGTTGGGGGCATTAGTATTTAATTGTCAGAGGTGAAAT
TCTTGGATTTATTAAAGACTAACTTATGCGAAAGCATTTGCCAAGGATGTTTTCATTAATCAAGAACGAAAGTTAGGGGATCAAAGACG
ATCAGATACCGTCCTAGTCTTAACTATAAACTATACCGACTCGGGATCGGCCGGGTGA-TTTAAGCTCGGTCGGCACCGTATGAGAAAT
CAAAGTCTTTGGGTTCTGGGGGGAGTATGGTCGCAAGGCTGAAACTTAAAGGAATTGACGGAAGGGCACCACCAGGAGTGGAGCCTGCG
GCTTAATTTGACTCAACACGGGAAAACTTACCAGGTCAAAACATGGGTGAGATTGACAGATTGAGAGCTCTTTCTTGATTCTATGGGTG
GTGGTGCATGGCCGTTCTTAGTTGGTGGAGTGATTTGTCTGGTTAATTCCGTTAACGAACGAGACCTTAACCTGCTAATTAGTCAATTG
AATCACATTCAGTTGTACTTCTTAGAGGGACTATGCAGCGGAAGCGCATGGAAGTTTGAGGCAATAACAGGTCTGTGATGCCCCTAGAC
GTCCTGGGCCGCACGCGCGCTACAATGACACGCTCAGCAAGTATTATCCTGACCCGGAAGGGCCCCGGTAATCTTCACAATACGTGTCG
TGTTAGGGATAGATCTTTGCAATTATGGATCTTGAACGAGGAATTCCTAGTAAGTGCAAGTCATTAGCTTGTACTGATTACGTCCCTGC
CCTTTGTACACACCGCCCGTCGCTCCTACCGATTTCGAGTGATCAGGTGAACATTCCGGACTGCATTAG-CCCTATGACTAGTGTAGGA
AGTTAAGTAAACCTTATCACTTAGAGGAAGGAAAAGTCGTAACAAGGTTTCCGTAGGTGAACCTGCAGAAGGATCA
**>Metaradiophrya_lumbrici_RZ_4_LT**
AACCTGGTTGATCCTGCCAGTAGTCATATGCTTGTCTCAAAGATTAAGCCATGCATGTCTAAGTATAAATAGTATACAGTGAAACTGCG
AATGGCTCATTATAACAGTTATAGTTTATTTGATAATTGCAAGCTACATGGATAACCGTGGTAATTCTAGAGCTAATACATGCTGCAAA
ACCCGACTTC-GGAAGGGTTGTATTTATTAGATATCAAACCAATATTCCTTCGGGACTATTGTGGTGAATCATAGTAACTGATCGAATC
TCGAACTTTCGAGATAAATCATTCAAGTTTCTGCCCTATCAGCTTTCGATGGTAGTGTATTGGACTACCATGGCGGTCACGGGTAACGG
AGAATTAGGGTTCGATTCCGGAGAGGGAGCCTGAGAAACGGCTACCACATCTAAGGAAGGCAGCAGGCGCGTAAATTACCCAATCCTGA
CTCAGGGAGGTAGTGACAAGAAATAACAACCTGGGGGC-TATTAGCCTTACGGGATTGCAATGAGAACAATCCAAAACTCTTAGCGAGG
AACAATTGGAGGGCAAGTCTGGTGCCAGCAGCCGCGGTAATTCCAGCTCCAATAGCGTATATTAAAGTTGTTGCAGTTAAAAAGCTCGT
AGTTGAATTTCTGGCCGTTCTTAGTCTTGGCTTCGGTCGAGCTAAGGGCAGTCATCCGTCTGCAAACCATGCTAGTCCTTCACTGGTCG
GTATGGGGAGTAGGCACTTTACTTTGAAAAAATTAGAGTGTTTCAGGCAGGCCTTTGCCCGAATACATTAGCATGGAATAATGGAATAG
GACTTAGTCCA-TTTTGTTGGTTATTGGATTGAGTAATGATTAATAGGGACAGTTGGGGGCATTAGTATTTAATTGTCAGAGGTGAAAT

TCTTGGATTTATTAAAGACTAACTTATGCGAAAGCATTTGCCAAGGATGTTTTCATTAATCAAGAACGAAAGTTAGGGGATCAAAGACG
ATCAGATACCGTCCTAGTCTTAACTATAAACTATACCGACTCGGGATCGGCCGGGTGA-TTTAAGCTCGGTCGGCACCGTATGAGAAAT
CAAAGTCTTTGGGTTCTGGGGGGAGTATGGTCGCAAGGCTGAAACTTAAAGGAATTGACGGAAGGGCACCACCAGGAGTGGAGCCTGCG
GCTTAATTTGACTCAACACGGGAAAACTTACCAGGTCAAAACATGGGTGAGATTGACAGATTGAGAGCTCTTTCTTGATTCTATGGGTG
GTGGTGCATGGCCGTTCTTAGTTGGTGGAGTGATTTGTCTGGTTAATTCCGTTAACGAACGAGACCTTAACCTGCTAATTAGTCATTTG
AATCGCATTCAGATGTACTTCTTAGAGGGACTATGCAGCGGAAGCGCATGGAAGTTTGAGGCAATAACAGGTCTGTGATGCCCCTAGAC
GTCCTGGGCCGCACGCGCGCTACAATGACACGCTCAGCAAGTATTATCCTGACCCGGAAGGGCCCCGGTAATCTTCACAATACGTGTCG
TGTTAGGGATAGATCTTTGCAATTATGGATCTTGAACGAGGAATTCCTAGTAAGTGCAAGTCATTAGCTTGTACTGATTACGTCCCTGC
CCTTTGTACACACCGCCCGTCGCTCCTACCGATTTCGAGTGATCAGGTGAACATTCCGGACTGCATTAG-TCTTCGGTCTAGTGTAGGA
AGTTAAGTAAACCTTATCACTTAGAGGAAGGAAAAGTCGTAACAAGGTTTCCGTAGGTGAACCTGCAGAAGGATCA
**>Metaradiophrya_lumbrici_RZ_5_LT**
AACCTGGTTGATCCTGCCAGTAGTCATATGCTTGTCTCAAAGATTAAGCCATGCATGTCTAAGTATAAATAGTATACAGTGAAACTGCG
AATGGCTCATTATAACAGTTATAGTTTATTTGATAATTGCAAGCTACATGGATAACCGTGGTAATTCTAGAGCTAATACATGCTGCAAA
ACCCGACTTC-GGAAGGGTTGTATTTATTAGATATCAAACCAATATTCCTTCGGGACTATTGTGGTGAATCATAGTAACTGATCGAATC
TCGAACTTTCGAGATAAATCATTCAAGTTTCTGCCCTATCAGCTTTCGATGGTAGTGTATTGGACTACCATGGCGGTCACGGGTAACGG
AGAATTAGGGTTCGATTCCGGAGAGGGAGCCTGAGAAACGGCTACCACATCTAAGGAAGGCAGCAGGCGCGTAAATTACCCAATCCTGA
CTCAGGGAGGTAGTGACAAGAAATAACAACCTGGGGGC-TATTAGCCTTACGGGATTGCAATGAGAACAATCCAAAACTCTTAGCGAGG
AACAATTGGAGGGCAAGTCTGGTGCCAGCAGCCGCGGTAATTCCAGCTCCAATAGCGTATATTAAAGTTGTTGCAGTTAAAAAGCTCGT
AGTTGAATTTCTGGCCGTTCTTAGTCTTGGCTTCGGTCGAGCTAAGGGCAGTCATCCGTCTGCAAACCATGCTAGTCCTTCACTGGTCG
GTATGGGGAGTAGGCACTTTACTTTGAAAAAATTAGAGTGTTTCAGGCAGGCCTTTGCCCGAATACATTAGCATGGAATAATGGAATAG
GACTTAGTCCA-TTTTGTTGGTTATTGGATTGAGTAATGATTAATAGGGACAGTTGGGGGCATTAGTATTTAATTGTCAGAGGTGAAAT
TCTTGGATTTATTAAAGACTAACTTATGCGAAAGCATTTGCCAAGGATGTTTTCATTAATCAAGAACGAAAGTTAGGGGATCAAAGACG
ATCAGATACCGTCCTAGTCTTAACTATAAACTATACCGACTCGGGATCGGCCGGGTGA-TTTAAGCTCGGTCGGCACCGTATGAGAAAT
CAAAGTCTTTGGGTTCTGGGGGGAGTATGGTCGCAAGGCTGAAACTTAAAGGAATTGACGGAAGGGCACCACCAGGAGTGGAGCCTGCG
GCTTAATTTGACTCAACACGGGAAAACTTACCAGGTCAAAACATGGGTGAGATTGACAGATTGAGAGCTCTTTCTTGATTCTATGGGTG
GTGGTGCATGGCCGTTCTTAGTTGGTGGAGTGATTTGTCTGGTTAATTCCGTTAACGAACGAGACCTTAACCTGCTAATTAGTCATTTG
AATCGCATTCAGATGTACTTCTTAGAGGGACTATGCAGCGGAAGCGCATGGAAGTTTGAGGCAATAACAGGTCTGTGATGCCCCTAGAC
GTCCTGGGCCGCACGCGCGCTACAATGACACGCTCAGCAAGTATTATCCTGACCCGGAAGGGCCCCGGTAATCTTCACAATACGTGTCG
TGTTAGGGATAGATCTTTGCAATTATGGATCTTGAACGAGGAATTCCTAGTAAGTGCAAGTCATTAGCTTGTACTGATTACGTCCCTGC
CCTTTGTACACACCGCCCGTCGCTCCTACCGATTTCGAGTGATCAGGTGAACATTCCGGACTGCATTAG-TCTTCGGTCTAGTGTAGGA
AGTTAAGTAAACCTTATCACTTAGAGGAAGGAAAAGTCGTAACAAGGTTTCCGTAGGTGAACCTGCAGAAGGATCA
**>Metaradiophrya_lumbrici_KR_8_LT**
AACCTGGTTGATCCTGCCAGTAGTCATATGCTTGTCTCAAAGATTAAGCCATGCATGTCTAAGTATAAATAGTATACAGTGAAACTGCG
AATGGCTCATTATAACAGTTATAGTTTATTTGATAATTGCAAGCTACATGGATAACCGTGGTAATTCTAGAGCTAATACATGCTGCAAA
ACCCGACTTC-GGAAGGGTTGTATTTATTAGATATCAAACCAATATTCCTTCGGGACTATTGTGGTGAATCATAGTAACTGATCGAATC
TCGAACTTTCGAGATAAATCATTCAAGTTTCTGCCCTATCAGCTTTCGATGGTAGTGTATTGGACTACCATGGCGGTCACGGGTAACGG
AGAATTAGGGTTCGATTCCGGAGAGGGAGCCTGAGAAACGGCTACCACATCTAAGGAAGGCAGCAGGCGCGTAAATTACCCAATCCTGA
CTCAGGGAGGTAGTGACAAGAAATAACAACCTGGGGGC-TATTAGCCTTACGGGATTGCAATGAGAACAATCCAAAACTCTTAGCGAGG
AACAATTGGAGGGCAAGTCTGGTGCCAGCAGCCGCGGTAATTCCAGCTCCAATAGCGTATATTAAAGTTGTTGCAGTTAAAAAGCTCGT
AGTTGAATTTCTGGCCGTTCTTAGTCTTGGCTTCGGTCGAGCTAAGGGCAGTCATCCGTCTGCAAACCATGCTAGTCCTTCACTGGTCG
GTATGGGGAGTAGGCACTTTACTTTGAAAAAATTAGAGTGTTTCAGGCAGGCCTTTGCCCGAATACATTAGCATGGAATAATGGAATAG
GACTTAGTCCA-TTTTGTTGGTTATTGGATTGAGTAATGATTAATAGGGACAGTTGGGGGCATTAGTATTTAATTGTCAGAGGTGAAAT
TCTTGGATTTATTAAAGACTAACTTATGCGAAAGCATTTGCCAAGGATGTTTTCATTAATCAAGAACGAAAGTTAGGGGATCAAAGACG
ATCAGATACCGTCCTAGTCTTAACTATAAACTATACCGACTCGGGATCGGCCGGGTGA-TTTAAGCTCGGTCGGCACCGTATGAGAAAT
CAAAGTCTTTGGGTTCTGGGGGGAGTATGGTCGCAAGGCTGAAACTTAAAGGAATTGACGGAAGGGCACCACCAGGAGTGGAGCCTGCG
GCTTAATTTGACTCAACACGGGAAAACTTACCAGGTCAAAACATGGGTGAGATTGACAGATTGAGAGCTCTTTCTTGATTCTATGGGTG
GTGGTGCATGGCCGTTCTTAGTTGGTGGAGTGATTTGTCTGGTTAATTCCGTTAACGAACGAGACCTTAACCTGCTAATTAGTCATTTG
AATCGCATTCAGATGTACTTCTTAGAGGGACTATGCAGCGGAAGCGCATGGAAGTTTGAGGCAATAACAGGTCTGTGATGCCCCTAGAC
GTCCTGGGCCGCACGCGCGCTACAATGACACGCTCAGCAAGTATTATCCTGACCCGGAAGGGCCCCGGTAATCTTCACAATACGTGTCG
TGTTAGGGATAGATCTTTGCAATTATGGATCTTGAACGAGGAATTCCTAGTAAGTGCAAGTCATTAGCTTGTACTGATTACGTCCCTGC
CCTTTGTACACACCGCCCGTCGCTCCTACCGATTTCGAGTGATCAGGTGAACATTCCGGACTGCATTAG-TCTTCGGTCTAGTGTAGGA
AGTTAAGTAAACCTTATCACTTAGAGGAAGGAAAAGTCGTAACAAGGTTTCCGTAGGTGAACCTGCAGAAGGATCA
**>Metaradiophrya_lumbrici_KR_10_LT**
AACCTGGTTGATCCTGCCAGTAGTCATATGCTTGTCTCAAAGATTAAGCCATGCATGTCTAAGTATAAATAGTATACAGTGAAACTGCG
AATGGCTCATTATAACAGTTATAGTTTATTTGATAATTGCAAGCTACATGGATAACCGTGGTAATTCTAGAGCTAATACATGCTGCAAA
ACCCGACTTC-GGAAGGGTTGTATTTATTAGATATCAAACCAATATTCCTTCGGGACTATTGTGGTGAATCATAGTAACTGATCGAATC
TCGAACTTTCGAGATAAATCATTCAAGTTTCTGCCCTATCAGCTTTCGATGGTAGTGTATTGGACTACCATGGCGGTCACGGGTAACGG
AGAATTAGGGTTCGATTCCGGAGAGGGAGCCTGAGAAACGGCTACCACATCTAAGGAAGGCAGCAGGCGCGTAAATTACCCAATCCTGA
CTCAGGGAGGTAGTGACAAGAAATAACAACCTGGGGGC-TATTAGCCTTACGGGATTGCAATGAGAACAATCCAAAACTCTTAGCGAGG
AACAATTGGAGGGCAAGTCTGGTGCCAGCAGCCGCGGTAATTCCAGCTCCAATAGCGTATATTAAAGTTGTTGCAGTTAAAAAGCTCGT
AGTTGAATTTCTGGCCGTTCTTAGTCTTGGCTTCGGTCGAGCTAAGGGCAGTCATCCGTCTGCAAACCATGCTAGTCCTTCACTGGTCG
GTATGGGGAGTAGGCACTTTACTTTGAAAAAATTAGAGTGTTTCAGGCAGGCCTTTGCCCGAATACATTAGCATGGAATAATGGAATAG
GACTTAGTCCA-TTTTGTTGGTTATTGGATTGAGTAATGATTAATAGGGACAGTTGGGGGCATTAGTATTTAATTGTCAGAGGTGAAAT
TCTTGGATTTATTAAAGACTAACTTATGCGAAAGCATTTGCCAAGGATGTTTTCATTAATCAAGAACGAAAGTTAGGGGATCAAAGACG
ATCAGATACCGTCCTAGTCTTAACTATAAACTATACCGACTCGGGATCGGCCGGGTGA-TTTAAGCTCGGTCGGCACCGTATGAGAAAT
CAAAGTCTTTGGGTTCTGGGGGGAGTATGGTCGCAAGGCTGAAACTTAAAGGAATTGACGGAAGGGCACCACCAGGAGTGGAGCCTGCG
GCTTAATTTGACTCAACACGGGAAAACTTACCAGGTCAAAACATGGGTGAGATTGACAGATTGAGAGCTCTTTCTTGATTCTATGGGTG
GTGGTGCATGGCCGTTCTTAGTTGGTGGAGTGATTTGTCTGGTTAATTCCGTTAACGAACGAGACCTTAACCTGCTAATTAGTCATTTG
AATCGCATTCAGATGTACTTCTTAGAGGGACTATGCAGCGGAAGCGCATGGAAGTTTGAGGCAATAACAGGTCTGTGATGCCCCTAGAC
GTCCTGGGCCGCACGCGCGCTACAATGACACGCTCAGCAAGTATTATCCTGACCCGGAAGGGCCCCGGTAATCTTCACAATACGTGTCG
TGTTAGGGATAGATCTTTGCAATTATGGATCTTGAACGAGGAATTCCTAGTAAGTGCAAGTCATTAGCTTGTACTGATTACGTCCCTGC

CCTTTGTACACACCGCCCGTCGCTCCTACCGATTTCGAGTGATCAGGTGAACATTCCGGACTGCATTAG-TCTTCGGTCTAGTGTAGGA
AGTTAAGTAAACCTTATCACTTAGAGGAAGGAAAAGTCGTAACAAGGTTTCCGTAGGTGAACCTGCAGAAGGATCA
**>Metaradiophrya_lumbrici_JA2_25_LT**
AACCTGGTTGATCCTGCCAGTAGTCATATGCTTGTCTCAAAGATTAAGCCATGCATGTCTAAGTATAAATAGTATACAGTGAAACTGCG
AATGGCTCATTATAACAGTTATAGTTTATTTGATAATTGCAAGCTACATGGATAACCGTGGTAATTCTAGAGCTAATACATGCTGCAAA
ACCCGACTTC-GGAAGGGTTGTATTTATTAGATATCAAACCAATATTCCTTCGGGACTATTGTGGTGAATCATAGTAACTGATCGAATC
TCGAACTTTCGAGATAAATCATTCAAGTTTCTGCCCTATCAGCTTTCGATGGTAGTGTATTGGACTACCATGGCGGTCACGGGTAACGG
AGAATTAGGGTTCGATTCCGGAGAGGGAGCCTGAGAAACGGCTACCACATCTAAGGAAGGCAGCAGGCGCGTAAATTACCCAATCCTGA
CTCAGGGAGGTAGTGACAAGAAATAACAACCTGGGGGC-TATTAGCCTTACGGGATTGCAATGAGAACAATCCAAAACTCTTAGCGAGG
AACAATTGGAGGGCAAGTCTGGTGCCAGCAGCCGCGGTAATTCCAGCTCCAATAGCGTATATTAAAGTTGTTGCAGTTAAAAAGCTCGT
AGTTGAATTTCTGGCCGTTCTTAGTCTTGGCTTCGGTCGAGCTAAGGGCAGTCATCCGTCTGCAAACCATGCTAGTCCTTCACTGGTCG
GTATGGGGAGTAGGCACTTTACTTTGAAAAAATTAGAGTGTTTCAGGCAGGCCTTTGCCCGAATACATTAGCATGGAATAATGGAATAG
GACTTAGTCCA-TTTTGTTGGTTATTGGATTGAGTAATGATTAATAGGGACAGTTGGGGGCATTAGTATTTAATTGTCAGAGGTGAAAT
TCTTGGATTTATTAAAGACTAACTTATGCGAAAGCATTTGCCAAGGATGTTTTCATTAATCAAGAACGAAAGTTAGGGGATCAAAGACG
ATCAGATACCGTCCTAGTCTTAACTATAAACTATACCGACTCGGGATCGGCCGGGTGA-TTTAAGCTCGGTCGGCACCGTATGAGAAAT
CAAAGTCTTTGGGTTCTGGGGGGAGTATGGTCGCAAGGCTGAAACTTAAAGGAATTGACGGAAGGGCACCACCAGGAGTGGAGCCTGCG
GCTTAATTTGACTCAACACGGGAAAACTTACCAGGTCAAAACATGGGTGAGATTGACAGATTGAGAGCTCTTTCTTGATTCTATGGGTG
GTGGTGCATGGCCGTTCTTAGTTGGTGGAGTGATTTGTCTGGTTAATTCCGTTAACGAACGAGACCTTAACCTGCTAATTAGTCATTTG
AATCGCATTCAGATGTACTTCTTAGAGGGACTATGCAGCGGAAGCGCATGGAAGTTTGAGGCAATAACAGGTCTGTGATGCCCCTAGAC
GTCCTGGGCCGCACGCGCGCTACAATGACACGCTCAGCAAGTATTATCCTGACCCGGAAGGGCCCCGGTAATCTTCACAATACGTGTCG
TGTTAGGGATAGATCTTTGCAATTATGGATCTTGAACGAGGAATTCCTAGTAAGTGCAAGTCATTAGCTTGTACTGATTACGTCCCTGC
CCTTTGTACACACCGCCCGTCGCTCCTACCGATTTCGAGTGATCAGGTGAACATTCCGGACTGCATTAG-TCTTCGGTCTAGTGTAGGA
AGTTAAGTAAACCTTATCACTTAGAGGAAGGAAAAGTCGTAACAAGGTTTCCGTAGGTGAACCTGCAGAAGGATCA
**>Metaradiophrya_lumbrici_JA2_26_LT**
AACCTGGTTGATCCTGCCAGTAGTCATATGCTTGTCTCAAAGATTAAGCCATGCATGTCTAAGTATAAATAGTATACAGTGAAACTGCG
AATGGCTCATTATAACAGTTATAGTTTATTTGATAATTGCAAGCTACATGGATAACCGTGGTAATTCTAGAGCTAATACATGCTGCAAA
ACCCGACTTC-GGAAGGGTTGTATTTATTAGATATCAAACCAATATTCCTTCGGGACTATTGTGGTGAATCATAGTAACTGATCGAATC
TCGAACTTTCGAGATAAATCATTCAAGTTTCTGCCCTATCAGCTTTCGATGGTAGTGTATTGGACTACCATGGCGGTCACGGGTAACGG
AGAATTAGGGTTCGATTCCGGAGAGGGAGCCTGAGAAACGGCTACCACATCTAAGGAAGGCAGCAGGCGCGTAAATTACCCAATCCTGA
CTCAGGGAGGTAGTGACAAGAAATAACAACCTGGGGGC-TATTAGCCTTACGGGATTGCAATGAGAACAATCCAAAACTCTTAGCGAGG
AACAATTGGAGGGCAAGTCTGGTGCCAGCAGCCGCGGTAATTCCAGCTCCAATAGCGTATATTAAAGTTGTTGCAGTTAAAAAGCTCGT
AGTTGAATTTCTGGCCGTTCTTAGTCTTGGCTTCGGTCGAGCTAAGGGCAGTCATCCGTCTGCAAACCATGCTAGTCCTTCACTGGTCG
GTATGGGGAGTAGGCACTTTACTTTGAAAAAATTAGAGTGTTTCAGGCAGGCCTTTGCCCGAATACATTAGCATGGAATAATGGAATAG
GACTTAGTCCA-TTTTGTTGGTTATTGGATTGAGTAATGATTAATAGGGACAGTTGGGGGCATTAGTATTTAATTGTCAGAGGTGAAAT
TCTTGGATTTATTAAAGACTAACTTATGCGAAAGCATTTGCCAAGGATGTTTTCATTAATCAAGAACGAAAGTTAGGGGATCAAAGACG
ATCAGATACCGTCCTAGTCTTAACTATAAACTATACCGACTCGGGATCGGCCGGGTGA-TTTAAGCTCGGTCGGCACCGTATGAGAAAT
CAAAGTCTTTGGGTTCTGGGGGGAGTATGGTCGCAAGGCTGAAACTTAAAGGAATTGACGGAAGGGCACCACCAGGAGTGGAGCCTGCG
GCTTAATTTGACTCAACACGGGAAAACTTACCAGGTCAAAACATGGGTGAGATTGACAGATTGAGAGCTCTTTCTTGATTCTATGGGTG
GTGGTGCATGGCCGTTCTTAGTTGGTGGAGTGATTTGTCTGGTTAATTCCGTTAACGAACGAGACCTTAACCTGCTAATTAGTCATTTG
AATCGCATTCAGATGTACTTCTTAGAGGGACTATGCAGCGGAAGCGCATGGAAGTTTGAGGCAATAACAGGTCTGTGATGCCCCTAGAC
GTCCTGGGCCGCACGCGCGCTACAATGACACGCTCAGCAAGTATTATCCTGACCCGGAAGGGCCCCGGTAATCTTCACAATACGTGTCG
TGTTAGGGATAGATCTTTGCAATTATGGATCTTGAACGAGGAATTCCTAGTAAGTGCAAGTCATTAGCTTGTACTGATTACGTCCCTGC
CCTTTGTACACACCGCCCGTCGCTCCTACCGATTTCGAGTGATCAGGTGAACATTCCGGACTGCATTAG-TCTTCGGTCTAGTGTAGGA
AGTTAAGTAAACCTTATCACTTAGAGGAAGGAAAAGTCGTAACAAGGTTTCCGTAGGTGAACCTGCAGAAGGATCA
**>Metaradiophrya_speculorum_HkD_59_AT**
AACCTGGTTGATCCTGCCAGTAGTCATATGCTTGTCTCAAAGATTAAGCCATGCATGTCTAAGTATAAATAGTATACAGTGAAACTGCG
AATGGCTCATTATAACAGTTATAGTTTATTTGATAATTGCAAGCTACATGGATAACCGTGGTAATTCTAGAGCTAATACATGCTGCAAA
ACCCAACTTC-GGAAGGGTTGTATTTATTAGATATCAAACCAATATTCCTTCGGGTCTATTGTGGTGAATCATAGTAACTGATCGAATC
TCGAACTTTCGAGATAAATCATTCAAGTTTCTGCCCTATCAGCTTTCGATGGTAGTGTATTGGACTACCATGGCAGTCACGGGTAACGG
AGAATTAGGGTTCGATTCCGGAGAGGGAGCCTGAGAAACGGCTACCACATCTAAGGAAGGCAGCAGGCGCGTAAATTACCCAATCCTGA
CTCAGGGAGGTAGTGACAAGAAATAACAACCTGGGGGC-TATTAGCCTTACGGGATTGCAATGAGAACAATCCAAAACTCTTAGCGAGG
AACAATTGGAGGGCAAGTCTGGTGCCAGCAGCCGCGGTAATTCCAGCTCCAATAGCGTATATTAAAGTTGTTGCAGTTAAAAAGCTCGT
AGTTGAATTTCTGACCGTTCTTAGTCTTGGCTTCGGTCGAGTTAAGGGCAGTCATCCGTCTGCAAACCATGCTAGTCCTTTACTGGTCG
GTATGGGGAGTAGGCACTTTACTTTGAAAAAATTAGAGTGTTTCAGGCAGGCCTTTGCCCGAATACATTAGCATGGAATAATGGAATAG
GACTTAGTCCA-TTTTGTTGGTTATTGGATTGAGTAATGATTAATAGGGACAGTTGGGGGCATTAGTATTTAATTGTCAGAGGTGAAAT
TCTTGGATTTATTAAAGACTAACTTATGCGAAAGCATTTGCCAAGGATGTTTTCATTAATCAAGAACGAAAGTTAGGGGATCAAAGACG
ATCAGATACCGTCCTAGTCTTAACTATAAACTATACCGACTCGGGATCGGCCGGGTGACTTTAAGCTCGGTCGGCACCGTATGAGAAAT
CAAAGTCTTTGGGTTCTGGGGGGAGTATGGTCGCAAGGCTGAAACTTAAAGGAATTGACGGAAGGGCACCACCAGGAGTGGAGCCTGCG
GCTTAATTTGACTCAACACGGGAAAACTTACCAGGTCAAAACATGGGTGAGATTGACAGATTGAGAGCTCTTTCTTGATTCTATGGGTG
GTGGTGCATGGCCGTTCTTAGTTGGTGGAGTGATTTGTCTGGTTAATTCCGTTAACGAACGAGACCTTAACCTGCTAATTAGTCACTTG
AATCCCATTCAGGTGTACTTCTTAGAGGGACTATGCAGCGGAAGCGCATGGAAGTTTGAGGCAATAACAGGTCTGTGATGCCCCTAGAC
GTCCTGGGCCGCACGCGCGCTACAATGACACGCTCAGCAAGTATTATCCTGACCCGGAAGGGCCCCGGTAATCTTCACAATACGTGTCG
TGTTAGGGATAGATCTTTGCAATTATGGATCTTGAACGAGGAATTCCTAGTAAGTGCAAGTCATTAGCTTGTACTGATTACGTCCCTGC
CCTTTGTACACACCGCCCGTCGCTCCTACCGATTTCGAGTGATCAGGTGAACATTCCGGACTGCATTAG-TTTTCGGACTAGTGTAGGA
AGTTAAGTAAACCTTATCACTTAGAGGAAGGAAAAGTCGTAACAAGGTTTCCGTAGGTGAACCTGCAGAAGGATCA
**>Metaradiophrya_speculorum_HkD_60_AT**
AACCTGGTTGATCCTGCCAGTAGTCATATGCTTGTCTCAAAGATTAAGCCATGCATGTCTAAGTATAAATAGTATACAGTGAAACTGCG
AATGGCTCATTATAACAGTTATAGTTTATTTGATAATTGCAAGCTACATGGATAACCGTGGTAATTCTAGAGCTAATACATGCTGCAAA
ACCCAACTTC-GGAAGGGTTGTATTTATTAGATATCAAACCAATATTCCTTCGGGTCTATTGTGGTGAATCATAGTAACTGATCGAATC
TCGAACTTTCGAGATAAATCATTCAAGTTTCTGCCCTATCAGCTTTCGATGGTAGTGTATTGGACTACCATGGCAGTCACGGGTAACGG
AGAATTAGGGTTCGATTCCGGAGAGGGAGCCTGAGAAACGGCTACCACATCTAAGGAAGGCAGCAGGCGCGTAAATTACCCAATCCTGA

CTCAGGGAGGTAGTGACAAGAAATAACAACCTGGGGGC-TATTAGCCTTACGGGATTGCAATGAGAACAATCCAAAACTCTTAGCGAGG
AACAATTGGAGGGCAAGTCTGGTGCCAGCAGCCGCGGTAATTCCAGCTCCAATAGCGTATATTAAAGTTGTTGCAGTTAAAAAGCTCGT
AGTTGAATTTCTGACCGTTCTTAGTCTTGGCTTCGGTCGAGTTAAGGGCAGTCATCCGTCTGCAAACCATGCTAGTCCTTTACTGGTCG
GTATGGGGAGTAGGCACTTTACTTTGAAAAAATTAGAGTGTTTCAGGCAGGCCTTTGCCCGAATACATTAGCATGGAATAATGGAATAG
GACTTAGTCCA-TTTTGTTGGTTATTGGATTGAGTAATGATTAATAGGGACAGTTGGGGGCATTAGTATTTAATTGTCAGAGGTGAAAT
TCTTGGATTTATTAAAGACTAACTTATGCGAAAGCATTTGCCAAGGATGTTTTCATTAATCAAGAACGAAAGTTAGGGGATCAAAGACG
ATCAGATACCGTCCTAGTCTTAACTATAAACTATACCGACTCGGGATCGGCCGGGTGACTTTAAGCTCGGTCGGCACCGTATGAGAAAT
CAAAGTCTTTGGGTTCTGGGGGGAGTATGGTCGCAAGGCTGAAACTTAAAGGAATTGACGGAAGGGCACCACCAGGAGTGGAGCCTGCG
GCTTAATTTGACTCAACACGGGAAAACTTACCAGGTCAAAACATGGGTGAGATTGACAGATTGAGAGCTCTTTCTTGATTCTATGGGTG
GTGGTGCATGGCCGTTCTTAGTTGGTGGAGTGATTTGTCTGGTTAATTCCGTTAACGAACGAGACCTTAACCTGCTAATTAGTCACTTG
AATCCCATTCAGGTGTACTTCTTAGAGGGACTATGCAGCGGAAGCGCATGGAAGTTTGAGGCAATAACAGGTCTGTGATGCCCCTAGAC
GTCCTGGGCCGCACGCGCGCTACAATGACACGCTCAGCAAGTATTATCCTGACCCGGAAGGGCCCCGGTAATCTTCACAATACGTGTCG
TGTTAGGGATAGATCTTTGCAATTATGGATCTTGAACGAGGAATTCCTAGTAAGTGCAAGTCATTAGCTTGTACTGATTACGTCCCTGC
CCTTTGTACACACCGCCCGTCGCTCCTACCGATTTCGAGTGATCAGGTGAACATTCCGGACTGCATTAG-TTTTCGGACTAGTGTAGGA
AGTTAAGTAAACCTTATCACTTAGAGGAAGGAAAAGTCGTAACAAGGTTTCCGTAGGTGAACCTGCAGAAGGATCA

**>Metaradiophrya_varians_BZ_12_EF**

AACCTGGTTGATCCTGCCAGTAGTCATATGCTTGTCTCAAAGATTAAGCCATGCATGTCTAAGTATAAATAGTATACAGTGAAACTGCG
AATGGCTCATTATAACAGTTATAGTTTATTTGATAATTGCAAGCTACATGGATAACCGTGGTAATTCTAGAGCTAATACATGCTGCAAA
ACCCAACTTC-GGAAGGGTTGTATTTATTAGATATCAAACCAATATTCCTTCGGGTCTATTGTGGTGAATCATAGTAACTGATCAAATC
TCGAACCTTCGAGATAAATCATTCAAGTTTCTGCCCTATCAGCTTTCGATGGTAGTGTATTGGACTACCATGGCAGTCACGGGTAACGG
AGAATTAGGGTTCGATTCCGGAGAGGGAGCCTGAGAAACGGCTACCACATCTAAGGAAGGCAGCAGGCGCGTAAATTACCCAATCCTGA
CTCAGGGAGGTAGTGACAAGAAATAACAACCTGGGGGCATAACAGCCTTACGGGATTGCAATGAGAACAATCCAAAACTCTTAGCGAGG
AACAATTGGAGGGCAAGTCTGGTGCCAGCAGCCGCGGTAATTCCAGCTCCAATAGCGTATATTAAAGTTGTTGCAGTTAAAAAGCTCGT
AGTTGAATTTCTGGCCGTTCTTAGTCTTGGCTTTAGTCGAGCTAAGGGCAGTCATCCGTCTGCAAACCATGCTAGTCCTTTATTGGTCG
GTATGGGGAGTAGGCACTTTACTTTGAAAAAATTAGAGTGTTTCAGGCAGGCATTTGCCCGAATACATTAGCATGGAATAATGGAATAG
GACTTAGTCCA-TTTTGTTGGTTATTGGATTGAGTAATGATTAATAGGGACAGTTGGGGGCATTAGTATTTAATTGTCAGAGGTGAAAT
TCTTGGATTTATTAAAGACTAACTTATGCGAAAGCATTTGCCAAGGATGTTTTCATTAATCAAGAACGAAAGTTAGGGGATCAAAGACG
ATCAGATACCGTCCTAGTCTTAACTATAAACTATACCGACTCGGGATCGGCCGGGTGA-CTAAAGCTCGGTCGGCACCGTATGAGAAAT
CAAAGTCTTTGGGTTCTGGGGGGAGTATGGTCGCAAGGCTGAAACTTAAAGGAATTGACGGAAGGGCACCACCAGGAGTGGAGCCTGCG
GCTTAATTTGACTCAACACGGGAAAACTTACCAGGTCAAAACATGGGTGAGATTGACAGATTGAGAGCTCTTTCTTGATTCTATGGGTG
GTGGTGCATGGCCGTTCTTAGTTGGTGGAGTGATTTGTCTGGTTAATTCCGTTAACGAACGAGACCTTAACCTGCTAATTAGTCACTTG
AATCCCATTCAGGTGTACTTCTTAGAGGGACTATGCAGCGGAAGCGCATGGAAGTTTGAGGCAATAACAGGTCTGTGATGCCCCTAGAC
GTCCTGGGCCGCACGCGCGCTACAATGACACGCTCAGCAAGTATTATCCTGACCCGGAAGGGCTTCGGTAATCTTCACAATACGTGTCG
TGTTAGGGATAGATCTTTGCAATTATGGATCTTGAACGAGGAATTCCTAGTAAGTGCAAGTCATTAGCTTGTACTGATTACGTCCCTGC
CCTTTGTACACACCGCCCGTCGCTCCTACCGATTTCGAGTGATCAGGTGAACATTCCGGACTGCATTAGTTCTTAAGATTAGTGTAGGA
AGTTAAGTAAACCTTATCACTTAGAGGAAGGAAAAGTCGTAACAAGGTTTCCGTAGGTGAACCTGCAGAAGGATCA

**>Metaradiophrya_varians_BZ_14_EF**

AACCTGGTTGATCCTGCCAGTAGTCATATGCTTGTCTCAAAGATTAAGCCATGCATGTCTAAGTATAAATAGTATACAGTGAAACTGCG
AATGGCTCATTATAACAGTTATAGTTTATTTGATAATTGCAAGCTACATGGATAACCGTGGTAATTCTAGAGCTAATACATGCTGCAAA
ACCCAACTTC-GGAAGGGTTGTATTTATTAGATATCAAACCAATATTCCTTCGGGTCTATTGTGGTGAATCATAGTAACTGATCAAATC
TCGAACCTTCGAGATAAATCATTCAAGTTTCTGCCCTATCAGCTTTCGATGGTAGTGTATTGGACTACCATGGCAGTCACGGGTAACGG
AGAATTAGGGTTCGATTCCGGAGAGGGAGCCTGAGAAACGGCTACCACATCTAAGGAAGGCAGCAGGCGCGTAAATTACCCAATCCTGA
CTCAGGGAGGTAGTGACAAGAAATAACAACCTGGGGGCATAACAGCCTTACGGGATTGCAATGAGAACAATCCAAAACTCTTAGCGAGG
AACAATTGGAGGGCAAGTCTGGTGCCAGCAGCCGCGGTAATTCCAGCTCCAATAGCGTATATTAAAGTTGTTGCAGTTAAAAAGCTCGT
AGTTGAATTTCTGGCCGTTCTTAGTCTTGGCTTTAGTCGAGCTAAGGGCAGTCATCCGTCTGCAAACCATGCTAGTCCTTTATTGGTCG
GTATGGGGAGTAGGCACTTTACTTTGAAAAAATTAGAGTGTTTCAGGCAGGCATTTGCCCGAATACATTAGCATGGAATAATGGAATAG
GACTTAGTCCA-TTTTGTTGGTTATTGGATTGAGTAATGATTAATAGGGACAGTTGGGGGCATTAGTATTTAATTGTCAGAGGTGAAAT
TCTTGGATTTATTAAAGACTAACTTATGCGAAAGCATTTGCCAAGGATGTTTTCATTAATCAAGAACGAAAGTTAGGGGATCAAAGACG
ATCAGATACCGTCCTAGTCTTAACTATAAACTATACCGACTCGGGATCGGCCGGGTGA-CTAAAGCTCGGTCGGCACCGTATGAGAAAT
CAAAGTCTTTGGGTTCTGGGGGGAGTATGGTCGCAAGGCTGAAACTTAAAGGAATTGACGGAAGGGCACCACCAGGAGTGGAGCCTGCG
GCTTAATTTGACTCAACACGGGAAAACTTACCAGGTCAAAACATGGGTGAGATTGACAGATTGAGAGCTCTTTCTTGATTCTATGGGTG
GTGGTGCATGGCCGTTCTTAGTTGGTGGAGTGATTTGTCTGGTTAATTCCGTTAACGAACGAGACCTTAACCTGCTAATTAGTCACTTG
AATCCCATTCAGGTGTACTTCTTAGAGGGACTATGCAGCGGAAGCGCATGGAAGTTTGAGGCAATAACAGGTCTGTGATGCCCCTAGAC
GTCCTGGGCCGCACGCGCGCTACAATGACACGCTCAGCAAGTATTATCCTGACCCGGAAGGGCTTCGGTAATCTTCACAATACGTGTCG
TGTTAGGGATAGATCTTTGCAATTATGGATCTTGAACGAGGAATTCCTAGTAAGTGCAAGTCATTAGCTTGTACTGATTACGTCCCTGC
CCTTTGTACACACCGCCCGTCGCTCCTACCGATTTCGAGTGATCAGGTGAACATTCCGGACTGCATTAGTTCTTAAGATTAGTGTAGGA
AGTTAAGTAAACCTTATCACTTAGAGGAAGGAAAAGTCGTAACAAGGTTTCCGTAGGTGAACCTGCAGAAGGATCA

**>Metaradiophrya_varians_JA1_19_EF**

AACCTGGTTGATCCTGCCAGTAGTCATATGCTTGTCTCAAAGATTAAGCCATGCATGTCTAAGTATAAATAGTATACAGTGAAACTGCG
AATGGCTCATTATAACAGTTATAGTTTATTTGATAATTGCAAGCTACATGGATAACCGTGGTAATTCTAGAGCTAATACATGCTGCAAA
ACCCAACTTC-GGAAGGGTTGTATTTATTAGATATCAAACCAATATTCCTTCGGGTCTATTGTGGTGAATCATAGTAACTGATCAAATC
TCGAACCTTCGAGATAAATCATTCAAGTTTCTGCCCTATCAGCTTTCGATGGTAGTGTATTGGACTACCATGGCAGTCACGGGTAACGG
AGAATTAGGGTTCGATTCCGGAGAGGGAGCCTGAGAAACGGCTACCACATCTAAGGAAGGCAGCAGGCGCGTAAATTACCCAATCCTGA
CTCAGGGAGGTAGTGACAAGAAATAACAACCTGGGGGCATAACAGCCTTACGGGATTGCAATGAGAACAATCCAAAACTCTTAGCGAGG
AACAATTGGAGGGCAAGTCTGGTGCCAGCAGCCGCGGTAATTCCAGCTCCAATAGCGTATATTAAAGTTGTTGCAGTTAAAAAGCTCGT
AGTTGAATTTCTGGCCGTTCTTAGTCTTGGCTTTAGTCGAGCTAAGGGCAGTCATCCGTCTGCAAACCATGCTAGTCCTTTATTGGTCG
GTATGGGGAGTAGGCACTTTACTTTGAAAAAATTAGAGTGTTTCAGGCAGGCATTTGCCCGAATACATTAGCATGGAATAATGGAATAG
GACTTAGTCCA-TTTTGTTGGTTATTGGATTGAGTAATGATTAATAGGGACAGTTGGGGGCATTAGTATTTAATTGTCAGAGGTGAAAT
TCTTGGATTTATTAAAGACTAACTTATGCGAAAGCATTTGCCAAGGATGTTTTCATTAATCAAGAACGAAAGTTAGGGGATCAAAGACG
ATCAGATACCGTCCTAGTCTTAACTATAAACTATACCGACTCGGGATCGGCCGGGTGA-CTAAAGCTCGGTCGGCACCGTATGAGAAAT
CAAAGTCTTTGGGTTCTGGGGGGAGTATGGTCGCAAGGCTGAAACTTAAAGGAATTGACGGAAGGGCACCACCAGGAGTGGAGCCTGCG

GCTTAATTTGACTCAACACGGGAAAACTTACCAGGTCAAAACATGGGTGAGATTGACAGATTGAGAGCTCTTTCTTGATTCTATGGGTG
GTGGTGCATGGCCGTTCTTAGTTGGTGGAGTGATTTGTCTGGTTAATTCCGTTAACGAACGAGACCTTAACCTGCTAATTAGTCACTTG
AATCCCATTCAGGTGTACTTCTTAGAGGGACTATGCAGCGGAAGCGCATGGAAGTTTGAGGCAATAACAGGTCTGTGATGCCCCTAGAC
GTCCTGGGCCGCACGCGCGCTACAATGACACGCTCAGCAAGTATTATCCTGACCCGGAAGGGCTTCGGTAATCTTCACAATACGTGTCG
TGTTAGGGATAGATCTTTGCAATTATGGATCTTGAACGAGGAATTCCTAGTAAGTGCAAGTCATTAGCTTGTACTGATTACGTCCCTGC
CCTTTGTACACACCGCCCGTCGCTCCTACCGATTTCGAGTGATCAGGTGAACATTCCGGACTGCATTAGTTCTTAAGATTAGTGTAGGA
AGTTAAGTAAACCTTATCACTTAGAGGAAGGAAAAGTCGTAACAAGGTTTCCGTAGGTGAACCTGCAGAAGGATCA
**>Metaradiophrya_varians_JA1_22_EF**
AACCTGGTTGATCCTGCCAGTAGTCATATGCTTGTCTCAAAGATTAAGCCATGCATGTCTAAGTATAAATAGTATACAGTGAAACTGCG
AATGGCTCATTATAACAGTTATAGTTTATTTGATAATTGCAAGCTACATGGATAACCGTGGTAATTCTAGAGCTAATACATGCTGCAAA
ACCCAACTTC-GGAAGGGTTGTATTTATTAGATATCAAACCAATATTCCTTCGGGTCTATTGTGGTGAATCATAGTAACTGATCAAATC
TCGAACCTTCGAGATAAATCATTCAAGTTTCTGCCCTATCAGCTTTCGATGGTAGTGTATTGGACTACCATGGCAGTCACGGGTAACGG
AGAATTAGGGTTCGATTCCGGAGAGGGAGCCTGAGAAACGGCTACCACATCTAAGGAAGGCAGCAGGCGCGTAAATTACCCAATCCTGA
CTCAGGGAGGTAGTGACAAGAAATAACAACCTGGGGGCATAACAGCCTTACGGGATTGCAATGAGAACAATCCAAAACTCTTAGCGAGG
AACAATTGGAGGGCAAGTCTGGTGCCAGCAGCCGCGGTAATTCCAGCTCCAATAGCGTATATTAAAGTTGTTGCAGTTAAAAAGCTCGT
AGTTGAATTTCTGGCCGTTCTTAGTCTTGGCTTTAGTCGAGCTAAGGGCAGTCATCCGTCTGCAAACCATGCTAGTCCTTTATTGGTCG
GTATGGGGAGTAGGCACTTTACTTTGAAAAAATTAGAGTGTTTCAGGCAGGCATTTGCCCGAATACATTAGCATGGAATAATGGAATAG
GACTTAGTCCA-TTTTGTTGGTTATTGGATTGAGTAATGATTAATAGGGACAGTTGGGGGCATTAGTATTTAATTGTCAGAGGTGAAAT
TCTTGGATTTATTAAAGACTAACTTATGCGAAAGCATTTGCCAAGGATGTTTTCATTAATCAAGAACGAAAGTTAGGGGATCAAAGACG
ATCAGATACCGTCCTAGTCTTAACTATAAACTATACCGACTCGGGATCGGCCGGGTGA-CTAAAGCTCGGTCGGCACCGTATGAGAAAT
CAAAGTCTTTGGGTTCTGGGGGGAGTATGGTCGCAAGGCTGAAACTTAAAGGAATTGACGGAAGGGCACCACCAGGAGTGGAGCCTGCG
GCTTAATTTGACTCAACACGGGAAAACTTACCAGGTCAAAACATGGGTGAGATTGACAGATTGAGAGCTCTTTCTTGATTCTATGGGTG
GTGGTGCATGGCCGTTCTTAGTTGGTGGAGTGATTTGTCTGGTTAATTCCGTTAACGAACGAGACCTTAACCTGCTAATTAGTCACTTG
AATCCCATTCAGGTGTACTTCTTAGAGGGACTATGCAGCGGAAGCGCATGGAAGTTTGAGGCAATAACAGGTCTGTGATGCCCCTAGAC
GTCCTGGGCCGCACGCGCGCTACAATGACACGCTCAGCAAGTATTATCCTGACCCGGAAGGGCTTCGGTAATCTTCACAATACGTGTCG
TGTTAGGGATAGATCTTTGCAATTATGGATCTTGAACGAGGAATTCCTAGTAAGTGCAAGTCATTAGCTTGTACTGATTACGTCCCTGC
CCTTTGTACACACCGCCCGTCGCTCCTACCGATTTCGAGTGATCAGGTGAACATTCCGGACTGCATTAGTTCTTAAGATTAGTGTAGGA
AGTTAAGTAAACCTTATCACTTAGAGGAAGGAAAAGTCGTAACAAGGTTTCCGTAGGTGAACCTGCAGAAGGATCA
**>Metaradiophrya_varians_BZkv_31_EF**
AACCTGGTTGATCCTGCCAGTAGTCATATGCTTGTCTCAAAGATTAAGCCATGCATGTCTAAGTATAAATAGTATACAGTGAAACTGCG
AATGGCTCATTATAACAGTTATAGTTTATTTGATAATTGCAAGCTACATGGATAACCGTGGTAATTCTAGAGCTAATACATGCTGCAAA
ACCCAACTTC-GGAAGGGTTGTATTTATTAGATATCAAACCAATATTCCTTCGGGTCTATTGTGGTGAATCATAGTAACTGATCAAATC
TCGAACCTTCGAGATAAATCATTCAAGTTTCTGCCCTATCAGCTTTCGATGGTAGTGTATTGGACTACCATGGCAGTCACGGGTAACGG
AGAATTAGGGTTCGATTCCGGAGAGGGAGCCTGAGAAACGGCTACCACATCTAAGGAAGGCAGCAGGCGCGTAAATTACCCAATCCTGA
CTCAGGGAGGTAGTGACAAGAAATAACAACCTGGGGGCATAACAGCCTTACGGGATTGCAATGAGAACAATCCAAAACTCTTAGCGAGG
AACAATTGGAGGGCAAGTCTGGTGCCAGCAGCCGCGGTAATTCCAGCTCCAATAGCGTATATTAAAGTTGTTGCAGTTAAAAAGCTCGT
AGTTGAATTTCTGGCCGTTCTTAGTCTTGGCTTTAGTCGAGCTAAGGGCAGTCATCCGTCTGCAAACCATGCTAGTCCTTTATTGGTCG
GTATGGGGAGTAGGCACTTTACTTTGAAAAAATTAGAGTGTTTCAGGCAGGCATTTGCCCGAATACATTAGCATGGAATAATGGAATAG
GACTTAGTCCA-TTTTGTTGGTTATTGGATTGAGTAATGATTAATAGGGACAGTTGGGGGCATTAGTATTTAATTGTCAGAGGTGAAAT
TCTTGGATTTATTAAAGACTAACTTATGCGAAAGCATTTGCCAAGGATGTTTTCATTAATCAAGAACGAAAGTTAGGGGATCAAAGACG
ATCAGATACCGTCCTAGTCTTAACTATAAACTATACCGACTCGGGATCGGCCGGGTGA-CTAAAGCTCGGTCGGCACCGTATGAGAAAT
CAAAGTCTTTGGGTTCTGGGGGGAGTATGGTCGCAAGGCTGAAACTTAAAGGAATTGACGGAAGGGCACCACCAGGAGTGGAGCCTGCG
GCTTAATTTGACTCAACACGGGAAAACTTACCAGGTCAAAACATGGGTGAGATTGACAGATTGAGAGCTCTTTCTTGATTCTATGGGTG
GTGGTGCATGGCCGTTCTTAGTTGGTGGAGTGATTTGTCTGGTTAATTCCGTTAACGAACGAGACCTTAACCTGCTAATTAGTCACTTG
AATCCCATTCAGGTGTACTTCTTAGAGGGACTATGCAGCGGAAGCGCATGGAAGTTTGAGGCAATAACAGGTCTGTGATGCCCCTAGAC
GTCCTGGGCCGCACGCGCGCTACAATGACACGCTCAGCAAGTATTATCCTGACCCGGAAGGGCTTCGGTAATCTTCACAATACGTGTCG
TGTTAGGGATAGATCTTTGCAATTATGGATCTTGAACGAGGAATTCCTAGTAAGTGCAAGTCATTAGCTTGTACTGATTACGTCCCTGC
CCTTTGTACACACCGCCCGTCGCTCCTACCGATTTCGAGTGATCAGGTGAACATTCCGGACTGCATTAGTTCTTAAGATTAGTGTAGGA
AGTTAAGTAAACCTTATCACTTAGAGGAAGGAAAAGTCGTAACAAGGTTTCCGTAGGTGAACCTGCAGAAGGATCA
**>Metaradiophrya_varians_BZkv_32_EF**
AACCTGGTTGATCCTGCCAGTAGTCATATGCTTGTCTCAAAGATTAAGCCATGCATGTCTAAGTATAAATAGTATACAGTGAAACTGCG
AATGGCTCATTATAACAGTTATAGTTTATTTGATAATTGCAAGCTACATGGATAACCGTGGTAATTCTAGAGCTAATACATGCTGCAAA
ACCCAACTTC-GGAAGGGTTGTATTTATTAGATATCAAACCAATATTCCTTCGGGTCTATTGTGGTGAATCATAGTAACTGATCAAATC
TCGAACCTTCGAGATAAATCATTCAAGTTTCTGCCCTATCAGCTTTCGATGGTAGTGTATTGGACTACCATGGCAGTCACGGGTAACGG
AGAATTAGGGTTCGATTCCGGAGAGGGAGCCTGAGAAACGGCTACCACATCTAAGGAAGGCAGCAGGCGCGTAAATTACCCAATCCTGA
CTCAGGGAGGTAGTGACAAGAAATAACAACCTGGGGGCATAACAGCCTTACGGGATTGCAATGAGAACAATCCAAAACTCTTAGCGAGG
AACAATTGGAGGGCAAGTCTGGTGCCAGCAGCCGCGGTAATTCCAGCTCCAATAGCGTATATTAAAGTTGTTGCAGTTAAAAAGCTCGT
AGTTGAATTTCTGGCCGTTCTTAGTCTTGGCTTTAGTCGAGCTAAGGGCAGTCATCCGTCTGCAAACCATGCTAGTCCTTTATTGGTCG
GTATGGGGAGTAGGCACTTTACTTTGAAAAAATTAGAGTGTTTCAGGCAGGCATTTGCCCGAATACATTAGCATGGAATAATGGAATAG
GACTTAGTCCA-TTTTGTTGGTTATTGGATTGAGTAATGATTAATAGGGACAGTTGGGGGCATTAGTATTTAATTGTCAGAGGTGAAAT
TCTTGGATTTATTAAAGACTAACTTATGCGAAAGCATTTGCCAAGGATGTTTTCATTAATCAAGAACGAAAGTTAGGGGATCAAAGACG
ATCAGATACCGTCCTAGTCTTAACTATAAACTATACCGACTCGGGATCGGCCGGGTGA-CTAAAGCTCGGTCGGCACCGTATGAGAAAT
CAAAGTCTTTGGGTTCTGGGGGGAGTATGGTCGCAAGGCTGAAACTTAAAGGAATTGACGGAAGGGCACCACCAGGAGTGGAGCCTGCG
GCTTAATTTGACTCAACACGGGAAAACTTACCAGGTCAAAACATGGGTGAGATTGACAGATTGAGAGCTCTTTCTTGATTCTATGGGTG
GTGGTGCATGGCCGTTCTTAGTTGGTGGAGTGATTTGTCTGGTTAATTCCGTTAACGAACGAGACCTTAACCTGCTAATTAGTCACTTG
AATCCCATTCAGGTGTACTTCTTAGAGGGACTATGCAGCGGAAGCGCATGGAAGTTTGAGGCAATAACAGGTCTGTGATGCCCCTAGAC
GTCCTGGGCCGCACGCGCGCTACAATGACACGCTCAGCAAGTATTATCCTGACCCGGAAGGGCTTCGGTAATCTTCACAATACGTGTCG
TGTTAGGGATAGATCTTTGCAATTATGGATCTTGAACGAGGAATTCCTAGTAAGTGCAAGTCATTAGCTTGTACTGATTACGTCCCTGC
CCTTTGTACACACCGCCCGTCGCTCCTACCGATTTCGAGTGATCAGGTGAACATTCCGGACTGCATTAGTTCTTAAGATTAGTGTAGGA
AGTTAAGTAAACCTTATCACTTAGAGGAAGGAAAAGTCGTAACAAGGTTTCCGTAGGTGAACCTGCAGAAGGATCA

**>Subanoplophrya_nodulata_PU_29_OT**
AACCTGGTTGATCCTGCCAGTAGTCATATGCTTGTCTCAAAGATTAAGCCATGCATGTCTAAGTATAAATAGTATACAGTGAAACTGCG
AATGGCTCATTATAACAGTTATAGTTTATTTGATAATTGCAAGCTACATGGATAACCGTGGTAATTCTAGAGCTAATACATGCTGTCAA
ACCCGACTTCTGGAAGGGTTGTATTTATTAGATATTAAACCAATAGTCTTC--GGACTTTTGTGGTGAATCATAGTAACTGATCGAATC
TCGGAACTCCGAGATAAATCATTCAAGTTTCTGCCCTATCAGCTTTCGATGGTAGTGTATTGGACTACCATGGCAGTCACGGGTAACGG
AGAATTAGGGTTCGATTCCGGAGAGGGAGCCTGAGAAACGGCTACCACATCTAAGGAAGGCAGCAGGCGCGTAAATTACCCAATCCTGA
CTCAGGGAGGTAGTGACAAGAAATAACAACTTGGGGGC-TTTTAGCCTTACGGGATTGCAATGAGAACAATCCAAAACTCTTAGCGAGG
AACAATTGGAGGGCAAGTCTGGTGCCAGCAGCCGCGGTAATTCCAGCTCCAATAGCGTATATTAAAGTTGTTGCAGTTAAAAAGCTCGT
AGTTGAACTTCTGACTGTCGTTATCTCTGACTTCGGTCAAGATCGGGGCAGTCATCCGTTTGCAAACCATATTAGGTCTTAAGTGACCG
GTGTGGGGAGCAGACACCTTACTTTGAAAAAACTAGAGTGTTTCAGGCAGGCAATCGCCCGAATACATTAGCATGGAATAATGGAACAG
GACTTAGTCCA-TTTTATTGGTTATTGGATTGAGTAATGATTAATAGGGACAGTTGGGGGCATTAGTATTTAATTGTCAGAGGTGAAAT
TCTTGGATTTATTAAAGACTAACTTATGCGAAAGCATTTGCCAAGGATGTTTTCATTAATCAAGAACGAAAGTTAGGGGATCAAAGACG
ATCAGATACCGTCCTAGTCTTAACTATAAACTATACCGACTCGGGATCGGCCGGGTT--CCTATACTCGGTCGGCACCGTATGAGAAAT
CAAAGTCTTTGGGTTCTGGGGGGAGTATGGTCGCAAGGCTGAAACTTAAAGGAATTGACGGAAGGGCACCACCAGGAGTGGAGCCTGCG
GCTTAATTTGACTCAACACAGGAAAACTTACCAGGTCAAAACATGGGTGGGATTGACAGATTGAGAGCTCTTTCTTGATTCTATGGGTG
GTGGTGCATGGCCGTTCTTAGTTGGTGGAGTGATTTGTCTGGTTAATTCCGTTAACGAACGAGACCTTAACCTGCTAACTAGTCACCGG
AA-AATATTTCGGTGTACTTCTTAGAGGGACTATGCGGCGGAAACGCATGGAAGTTTGAGGCAATAACAGGTCTGTGATGCCCCTAGAC
GTCCTGGGCCGCACGCGCGCTACAATGACACGCTCAGCAAGTTTTAACCTAGCTCGAAAGAGTTTGGGTAATCTTTATAATACGTGTCG
TGTTAGGGATAGATCTTTGTAATTATGGATCTTGAACGAGGAATTCCTAGTAAGTGCAAGTCATTAGCTTGTACTGATTACGTCCCTGC
CCTTTGTACACACCGCCCGTCACTCCTACCGATTTCGAGTGATCAGGTGAACATTCCGGACTGAGGTAATCTCT--GGTTATCTTAGAA
AGTTAAGTAAACCTTATCACTTAGAGGAAGGAAAAGTCGTAACAAGGTTTCCGTAGGTGAACCTGCAGAAGGATCA
**>Subanoplophrya_nodulata_PU_30_OT**
AACCTGGTTGATCCTGCCAGTAGTCATATGCTTGTCTCAAAGATTAAGCCATGCATGTCTAAGTATAAATAGTATACAGTGAAACTGCG
AATGGCTCATTATAACAGTTATAGTTTATTTGATAATTGCAAGCTACATGGATAACCGTGGTAATTCTAGAGCTAATACATGCTGTCAA
ACCCGACTTCTGGAAGGGTTGTATTTATTAGATATTAAACCAATAGTCTTC--GGACTTTTGTGGTGAATCATAGTAACTGATCGAATC
TCGGAACTCCGAGATAAATCATTCAAGTTTCTGCCCTATCAGCTTTCGATGGTAGTGTATTGGACTACCATGGCAGTCACGGGTAACGG
AGAATTAGGGTTCGATTCCGGAGAGGGAGCCTGAGAAACGGCTACCACATCTAAGGAAGGCAGCAGGCGCGTAAATTACCCAATCCTGA
CTCAGGGAGGTAGTGACAAGAAATAACAACTTGGGGGC-TTTTAGCCTTACGGGATTGCAATGAGAACAATCCAAAACTCTTAGCGAGG
AACAATTGGAGGGCAAGTCTGGTGCCAGCAGCCGCGGTAATTCCAGCTCCAATAGCGTATATTAAAGTTGTTGCAGTTAAAAAGCTCGT
AGTTGAACTTCTGACTGTCGTTATCTCTGACTTCGGTCAAGATCGGGGCAGTCATCCGTTTGCAAACCATATTAGGTCTTAAGTGACCG
GTGTGGGGAGCAGACACCTTACTTTGAAAAAACTAGAGTGTTTCAGGCAGGCAATCGCCCGAATACATTAGCATGGAATAATGGAACAG
GACTTAGTCCA-TTTTATTGGTTATTGGATTGAGTAATGATTAATAGGGACAGTTGGGGGCATTAGTATTTAATTGTCAGAGGTGAAAT
TCTTGGATTTATTAAAGACTAACTTATGCGAAAGCATTTGCCAAGGATGTTTTCATTAATCAAGAACGAAAGTTAGGGGATCAAAGACG
ATCAGATACCGTCCTAGTCTTAACTATAAACTATACCGACTCGGGATCGGCCGGGTT--CCTATACTCGGTCGGCACCGTATGAGAAAT
CAAAGTCTTTGGGTTCTGGGGGGAGTATGGTCGCAAGGCTGAAACTTAAAGGAATTGACGGAAGGGCACCACCAGGAGTGGAGCCTGCG
GCTTAATTTGACTCAACACAGGAAAACTTACCAGGTCAAAACATGGGTGGGATTGACAGATTGAGAGCTCTTTCTTGATTCTATGGGTG
GTGGTGCATGGCCGTTCTTAGTTGGTGGAGTGATTTGTCTGGTTAATTCCGTTAACGAACGAGACCTTAACCTGCTAACTAGTCACCGG
AA-AATATTTCGGTGTACTTCTTAGAGGGACTATGCGGCGGAAACGCATGGAAGTTTGAGGCAATAACAGGTCTGTGATGCCCCTAGAC
GTCCTGGGCCGCACGCGCGCTACAATGACACGCTCAGCAAGTTTTAACCTAGCTCGAAAGAGTTTGGGTAATCTTTATAATACGTGTCG
TGTTAGGGATAGATCTTTGTAATTATGGATCTTGAACGAGGAATTCCTAGTAAGTGCAAGTCATTAGCTTGTACTGATTACGTCCCTGC
CCTTTGTACACACCGCCCGTCACTCCTACCGATTTCGAGTGATCAGGTGAACATTCCGGACTGAGGTAATCTCT--GGTTATCTTAGAA
AGTTAAGTAAACCTTATCACTTAGAGGAAGGAAAAGTCGTAACAAGGTTTCCGTAGGTGAACCTGCAGAAGGATCA

# ITS1-5.8S-ITS2 region alignment

**>Anoplophrya_allolobophorae_JA3_37_ACH**
CCTTAACTTCGCGCTTCGGCAGC-GGCAACGCTTGCAG-TCATTTGCCTTTTTGGAACG----CTTCTCGCGC-AC-TAGAACTCAAAA
ATTTTCAACGGTGGATATCTAGGTTCCCATTTCGATGAAGAACGCAGCAAAATGCGATAAGCAATGCGAATTGCAGAATTCCGCGAGTC
ATCAGATCTTTGAACGCAACTTGCGCTGGCGTAAAACCCAGCATGTTTGTTTCAGTGTGTC-ATCAA--GTCCGAGACCCATCG--CCC
AGCTTAATGCGACCGAAGGCCC--CGCCTTCCTTCGTCAAACT-GTTCCGTCCGGCGCTGT-TCGCCCCTCGAAGTAGTCACAAACCGC
TTGTGATTTCGTTGGGC-CTGCTGCGCCTTTTTGACGGCGCGC--TTCTTA-TTTGCACCTGAAATCAAGCAAGGTCACC
**>Anoplophrya_aporrectodeae_PUz_17_AT**
CCTTAACTTCGCGCTTCGGCAGC-GGCAACGCTTGCAG-TCATTTGCCTTTTTGGAACG----CTTCTCGCGC-AC-TAGAACTCAAAA
ATTTTCAACGGTGGATATCTAGGTTCCCATTTCGATGAAGAACGCAGCAAAATGCGATAAGCAATGCGAATTGCAGAATTCCGCGAGTC
ATCAGATCTTTGAACGCAACTTGCGCTGGCGTAAAACCCAGCATGTTTGTTTCAGTGTGTC-ATAAA--GTCCAAGACCCATCG--CCC
AGCTTAATGCGACCGAAGGCCT--CGCCTTCCTTCGTCAAACT-GTTCCGTCCGGCGCTGT-TCGCCCCTCGAAGTAGTCACAAACCGC
TTGTGATTTCGTTGGGC-CTGCTGCGCCTTTCTGACGGCGCGC--TTCTTA-TTTGCACCTGAAATCAAGCAAGGTCACC
**>Anoplophrya_aporrectodeae_PUz_40_AT**
CCTTAACTTCGCGCTTCGGCAGC-GGCAACGCTTGCAG-TCATTTGCCTTTTTGGAACG----CTTCTCGCGC-AC-TAGAACTCAAAA
ATTTTCAACGGTGGATATCTAGGTTCCCATTTCGATGAAGAACGCAGCAAAATGCGATAAGCAATGCGAATTGCAGAATTCCGCGAGTC
ATCAGATCTTTGAACGCAACTTGCGCTGGCGTAAAACCCAGCATGTTTGTTTCAGTGTGTC-ATAAA--GTCCAAGACCCATCG--CCC
AGCTTAATGCGACCGAAGGCCT--CGCCTTCCTTCGTCAAACT-GTTCCGTCCGGCGCTGT-TCGCCCCTCGAAGTAGTCACAAACCGC
TTGTGATTTCGTTGGGC-CTGCTGCGCCTTTCTGACGGCGCGC--TTCTTA-TTTGCACCTGAAATCAAGCAAGGTCACC
**>Anoplophrya_aporrectodeae_PUz_41_AT**
CCTTAACTTCGCGCTTCGGCAGC-GGCAACGCTTGCAG-TCATTTGCCTTTTTGGAACG----CTTCTCGCGC-AC-TAGAACTCAAAA
ATTTTCAACGGTGGATATCTAGGTTCCCATTTCGATGAAGAACGCAGCAAAATGCGATAAGCAATGCGAATTGCAGAATTCCGCGAGTC
ATCAGATCTTTGAACGCAACTTGCGCTGGCGTAAAACCCAGCATGTTTGTTTCAGTGTGTC-ATAAA--GTCCAAGACCCATCG--CCC
AGCTTAATGCGACCGAAGGCCT--CGCCTTCCTTCGTCAAACT-GTTCCGTCCGGCGCTGT-TCGCCCCTCGAAGTAGTCACAAACCGC
TTGTGATTTCGTTGGGC-CTGCTGCGCCTTTCTGACGGCGCGC--TTCTTA-TTTGCACCTGAAATCAAGCAAGGTCACC
**>Anoplophrya_lumbrici_RZ_6_LT**
CCTTAACTTCGCGCTTCAGCAGCGGGCAACCGCTGCAG-TCATTTACCTTTTTGGAACG----CTTCTCGCAA-ACTTAGAAATCAAAA
ATTTTCAACGGTGGATATCTAGGTTCCCATTTCGATGAAGAACGCAGCAAAATGCGATAAGCAATGCGAATTGCAGAATTCCGCGAGTC
ATCAGATCTTTGAACGCAACTTGCGCTGGCGTAAAACCCAGCATGTTTGTTTCAGTGTGTC-ATAAA--GTCAAAGAACCATCACCCCC
ATCTTAATGCGACCGAAGGTC---CGCCTTCCTTCGTCAAACT-GTTCCGTCCGGCAATGTATCGCCCCTCGAAGTAGTCACAAACCGC
TTGTGATTTCGTTGGGCTTTGCTTTGCCTTTTTGACGGCTTAC--TTCTTA-TTTGCACCTGAAATCAAGCAAGGTCACC
**>Anoplophrya_lumbrici_KR_9_LT**
CCTTAACTTCGCGCTTCAGCAGCGGGCAACCGCTGCAG-TCATTTACCTTTTTGGAACG----CTTCTCGCAA-ACTTAGAAATCAAAA
ATTTTCAACGGTGGATATCTAGGTTCCCATTTCGATGAAGAACGCAGCAAAATGCGATAAGCAATGCGAATTGCAGAATTCCGCGAGTC
ATCAGATCTTTGAACGCAACTTGCGCTGGCGTAAAACCCAGCATGTTTGTTTCAGTGTGTC-ATAAA--GTCAAAGAACCATCACCCCC
ATCTTAATGCGACCGAAGGTC---CGCCTTCCTTCGTCAAACT-GTTCCGTCCGGCAATGTATCGCCCCTCGAAGTAGTCACAAACCGC
TTGTGATTTCGTTGGGCTTTGCTTTGCCTTTTTGACGGCTTAC--TTCTTA-TTTGCACCTGAAATCAAGCAAGGTCACC
**>Anoplophrya_lumbrici_KR_11_LT**
CCTTAACTTCGCGCTTCAGCAGCGGGCAACCGCTGCAG-TCATTTACCTTTTTGGAACG----CTTCTCGCAA-ACTTAGAAATCAAAA
ATTTTCAACGGTGGATATCTAGGTTCCCATTTCGATGAAGAACGCAGCAAAATGCGATAAGCAATGCGAATTGCAGAATTCCGCGAGTC
ATCAGATCTTTGAACGCAACTTGCGCTGGCGTAAAACCCAGCATGTTTGTTTCAGTGTGTC-ATAAA--GTCAAAGAACCATCACCCCC
ATCTTAATGCGACCGAAGGTC---CGCCTTCCTTCGTCAAACT-GTTCCGTCCGGCAATGTATCGCCCCTCGAAGTAGTCACAAACCGC
TTGTGATTTCGTTGGGCTTTGCTTTGCCTTTTTGACGGCTTAC--TTCTTA-TTTGCACCTGAAATCAAGCAAGGTCACC
**>Anoplophrya_octolasionis_MU_56_OL**
CCTTAACTTCGCGCTTCAGCAGCGGGCGACCGCTGCAG-TCATTTACCTTTCTGGAACG----CTTCTC-CGA-ACCAAAAACCTAAAA
ATTTTCAACGGTGGATATCTAGGTTCCCATTTCGATGAAGAACGCAGCAAAATGCGATAAGCAATGCGAATTGCAGAATTCCGCGAGTC
ATCAGATCTTTGAACGCAACTTGCGCTGGCGTAAAACCCAGCATGTTTGTTTCAGTGTGTC-ATAAA--GTCACAGAACCATCG--CCC
AACTTAATGCGACCGAAGGTC---CGCCTTCCTTCGTCAAACT-GTTCCGTCCGGCAATGTATCGCCCCTCGAAGTAGTCACAAACCGC
TTGTGATTTCGTTGGGCTTTGCTTTGCCTTTTTGACGGCTCTC--TTCTTA-TTTGCACCTGAAATCAAGCAAGGTCACC
**>Anoplophrya_octolasionis_MU_57_OL**
CCTTAACTTCGCGCTTCAGCAGCGGGCGACCGCTGCAG-TCATTTACCTTTCTGGAACG----CTTCTC-CGA-ACCAAAAACCTAAAA
ATTTTCAACGGTGGATATCTAGGTTCCCATTTCGATGAAGAACGCAGCAAAATGCGATAAGCAATGCGAATTGCAGAATTCCGCGAGTC
ATCAGATCTTTGAACGCAACTTGCGCTGGCGTAAAACCCAGCATGTTTGTTTCAGTGTGTC-ATAAA--GTCACAGAACCATCG--CCC
AACTTAATGCGACCGAAGGTC---CGCCTTCCTTCGTCAAACT-GTTCCGTCCGGCAATGTATCGCCCCTCGAAGTAGTCACAAACCGC
TTGTGATTTCGTTGGGCTTTGCTTTGCCTTTTTGACGGCTCTC--TTCTTA-TTTGCACCTGAAATCAAGCAAGGTCACC
**>Anoplophrya_octolasionis_MU_58_OL**
CCTTAACTTCGCGCTTCAGCAGCGGGCGACCGCTGCAG-TCATTTACCTTTCTGGAACG----CTTCTC-CGA-ACCAAAAACCTAAAA
ATTTTCAACGGTGGATATCTAGGTTCCCATTTCGATGAAGAACGCAGCAAAATGCGATAAGCAATGCGAATTGCAGAATTCCGCGAGTC
ATCAGATCTTTGAACGCAACTTGCGCTGGCGTAAAACCCAGCATGTTTGTTTCAGTGTGTC-ATAAA--GTCACAGAACCATCG--CCC
AACTTAATGCGACCGAAGGTC---CGCCTTCCTTCGTCAAACT-GTTCCGTCCGGCAATGTATCGCCCCTCGAAGTAGTCACAAACCGC
TTGTGATTTCGTTGGGCTTTGCTTTGCCTTTTTGACGGCTCTC--TTCTTA-TTTGCACCTGAAATCAAGCAAGGTCACC
**>Anoplophrya_vulgaris_BZ_13_EF**
CCTTACCTTCGCGCTTCAGCAGC-GGCGAC-GCTGCAG-T-ATTTGCCTTTCTGGAACG----CTTCTCTCA--AC-TCGAATCTAAAA
ATTTTCAACGGTGGATATCTAGGTTCCCATTTCGATGAAGAACGCAGCAAAATGCGATAAGCAATGCGAATTGCAGAATTCCGCGAGTC
ATCAGATCTTTGAACGCAACTTGCGCTGGCGTAAAACCCAGCATGTTTGTTTCAGTGTGTC-ATCAA--GTCACCGACCCATCA---AC
AGCTTAATGCGACCGAAGT-----CCGCTTCCTTCGTCAAACT-GTTCCGTCCGGCTCTGT-GCGCCCCTCGAAGTAGTCACAAACCGC
TTGTGATTTCGTTGGGC-TCGCTCGGCCTTTCCGACGGCTCTC--TTCTTA-TTTGCACCTGAAATCAAGCAAGGTCACC
**>Anoplophrya_vulgaris_JA1_18_EF**
CCTTACCTTCGCGCTTCAGCAGC-GGCGAC-GCTGCAG-T-ATTTGCCTTTCTGGAACG----CTTCTCTCA--AC-TCGAATCTAAAA
ATTTTCAACGGTGGATATCTAGGTTCCCATTTCGATGAAGAACGCAGCAAAATGCGATAAGCAATGCGAATTGCAGAATTCCGCGAGTC

ATCAGATCTTTGAACGCAACTTGCGCTGGCGTAAAACCCAGCATGTTTGTTTCAGTGTGTC-ATCAA--GTCACCGACCCATCA---AC
AGCTTAATGCGACCGAAGT-----CCGCTTCCTTCGTCAAACT-GTTCCGTCCGGCTCTGT-GCGCCCCTCGAAGTAGTCACAAACCGC
TTGTGATTTCGTTGGGC-TCGCTCGGCCTTTCCGACGGCTCTC--TTCTTA-TTTGCACCTGAAATCAAGCAAGGTCACC
**>Anoplophrya_vulgaris_JA1_20_EF**
CCTTACCTTCGCGCTTCAGCAGC-GGCGAC-GCTGCAG-T-ATTTGCCTTTCTGGAACG----CTTCTCTCA--AC-TCGAATCTAAAA
ATTTTCAACGGTGGATATCTAGGTTCCCATTTCGATGAAGAACGCAGCAAAATGCGATAAGCAATGCGAATTGCAGAATTCCGCGAGTC
ATCAGATCTTTGAACGCAACTTGCGCTGGCGTAAAACCCAGCATGTTTGTTTCAGTGTGTC-ATCAA--GTCACCGACCCATCA---AC
AGCTTAATGCGACCGAAGT-----CCGCTTCCTTCGTCAAACT-GTTCCGTCCGGCTCTGT-GCGCCCCTCGAAGTAGTCACAAACCGC
TTGTGATTTCGTTGGGC-TCGCTCGGCCTTTCCGACGGCTCTC--TTCTTA-TTTGCACCTGAAATCAAGCAAGGTCACC
**>Anoplophrya_vulgaris_JA1_21_EF**
CCTTACCTTCGCGCTTCAGCAGC-GGCGAC-GCTGCAG-T-ATTTGCCTTTCTGGAACG----CTTCTCTCA--AC-TCGAATCTAAAA
ATTTTCAACGGTGGATATCTAGGTTCCCATTTCGATGAAGAACGCAGCAAAATGCGATAAGCAATGCGAATTGCAGAATTCCGCGAGTC
ATCAGATCTTTGAACGCAACTTGCGCTGGCGTAAAACCCAGCATGTTTGTTTCAGTGTGTC-ATCAA--GTCACCGACCCATCA---AC
AGCTTAATGCGACCGAAGT-----CCGCTTCCTTCGTCAAACT-GTTCCGTCCGGCTCTGT-GCGCCCCTCGAAGTAGTCACAAACCGC
TTGTGATTTCGTTGGGC-TCGCTCGGCCTTTCCGACGGCTCTC--TTCTTA-TTTGCACCTGAAATCAAGCAAGGTCACC
**>Anoplophrya_vulgaris_NG_27_DV**
CCTTACCTTCGCGCTTCAGCAGC-GGCGAC-GCTGCAG-T-ATTTGCCTTTCTGGAACG----CTTCTCTCA--AC-TCGAATCTAAAA
ATTTTCAACGGTGGATATCTAGGTTCCCATTTCGATGAAGAACGCAGCAAAATGCGATAAGCAATGCGAATTGCAGAATTCCGCGAGTC
ATCAGATCTTTGAACGCAACTTGCGCTGGCGTAAAACCCAGCATGTTTGTTTCAGTGTGTC-ATCAA--GTCACCGACCCATCA---AC
AGCTTAATGCGACCGAAGT-----CCGCTTCCTTCGTCAAACT-GTTCCGTCCGGCTCTGT-GCGCCCCTCGAAGTAGTCACAAACCGC
TTGTGATTTCGTTGGGC-TCGCTCGGCCTTTCCGACGGCTCTC--TTCTTA-TTTGCACCTGAAATCAAGCAAGGTCACC
**>Anoplophrya_vulgaris_NG_28_DV**
CCTTACCTTCGCGCTTCAGCAGC-GGCGAC-GCTGCAG-T-ATTTGCCTTTCTGGAACG----CTTCTCTCA--AC-TCGAATCTAAAA
ATTTTCAACGGTGGATATCTAGGTTCCCATTTCGATGAAGAACGCAGCAAAATGCGATAAGCAATGCGAATTGCAGAATTCCGCGAGTC
ATCAGATCTTTGAACGCAACTTGCGCTGGCGTAAAACCCAGCATGTTTGTTTCAGTGTGTC-ATCAA--GTCACCGACCCATCA---AC
AGCTTAATGCGACCGAAGT-----CCGCTTCCTTCGTCAAACT-GTTCCGTCCGGCTCTGT-GCGCCCCTCGAAGTAGTCACAAACCGC
TTGTGATTTCGTTGGGC-TCGCTCGGCCTTTCCGACGGCTCTC--TTCTTA-TTTGCACCTGAAATCAAGCAAGGTCACC
**>Maupasella_mucronata_KDo_33_ET**
CCTTAACTTCGCTTGCGACCACTG-------CTTGCAG-TGTCACAATCTTCCTTCAAGGAATCTTCACTAGACCCTTTCAATGAAAAA
ATTTTCAACGGTGGATATCTTGGTTCCCATTTCGATGAAGAACGCAGCTAACTGCGATACGCAATGCGAAAAGCAGGACCCCGCGAGTC
ATCAGATCTTCGAACGCAACTTGCGCCGGTGTAAAAGCCGGCATGTTTGTTTCAGTGTGCATACAAA--TCCTTTGAATTATCA--TTG
ACCTGAGTGCGACTGATGGTCT--AACTATCTTTCGTTAAGTTGGGGATGTCCAGCATTGT-GGGTCCCTCGAAGTAGTCACCATCTGC
--GTGATTTCGTTGGAC--TACTCTGCAGAAGCGACGTCAAACAATTCTTAACCAGTACCTGAAATCAAGCAAGGCCACC
**>Maupasella_mucronata_KDo_34_ET**
CCTTAACTTCGCTTGCGACCACTG-------CTTGCAG-TGTCACAATCTTCCTTCAAGGAATCTTCACTAGACCCTTTCAATGAAAAA
ATTTTCAACGGTGGATATCTTGGTTCCCATTTCGATGAAGAACGCAGCTAACTGCGATACGCAATGCGAAAAGCAGGACCCCGCGAGTC
ATCAGATCTTCGAACGCAACTTGCGCCGGTGTAAAAGCCGGCATGTTTGTTTCAGTGTGCATACAAA--TCCTTTGAATTATCA--TTG
ACCTGAGTGCGACTGATGGTCT--AACTATCTTTCGTTAAGTTGGGGATGTCCAGCATTGT-GGGTCCCTCGAAGTAGTCACCATCTGC
--GTGATTTCGTTGGAC--TACTCTGCAGAAGCGACGTCAAACAATTCTTAACCAGTACCTGAAATCAAGCAAGGCCACC
**>Maupasella_mucronata_KDo_35_ET**
CCTTAACTTCGCTTGCGACCACTG-------CTTGCAG-TGTCACAATCTTCCTTCAAGGAATCTTCACTAGACCCTTTCAATGAAAAA
ATTTTCAACGGTGGATATCTTGGTTCCCATTTCGATGAAGAACGCAGCTAACTGCGATACGCAATGCGAAAAGCAGGACCCCGCGAGTC
ATCAGATCTTCGAACGCAACTTGCGCCGGTGTAAAAGCCGGCATGTTTGTTTCAGTGTGCATACAAA--TCCTTTGAATTATCA--TTG
ACCTGAGTGCGACTGATGGTCT--AACTATCTTTCGTTAAGTTGGGGATGTCCAGCATTGT-GGGTCCCTCGAAGTAGTCACCATCTGC
--GTGATTTCGTTGGAC--TACTCTGCAGAAGCGACGTCAAACAATTCTTAACCAGTACCTGAAATCAAGCAAGGCCACC
**>Maupasella_mucronata_KDo_36_ET**
CCTTAACTTCGCTTGCGACCACTG-------CTTGCAG-TGTCACAATCTTCCTTCAAGGAATCTTCACTAGACCCTTTCAATGAAAAA
ATTTTCAACGGTGGATATCTTGGTTCCCATTTCGATGAAGAACGCAGCTAACTGCGATACGCAATGCGAAAAGCAGGACCCCGCGAGTC
ATCAGATCTTCGAACGCAACTTGCGCCGGTGTAAAAGCCGGCATGTTTGTTTCAGTGTGCATACAAA--TCCTTTGAATTATCA--TTG
ACCTGAGTGCGACTGATGGTCT--AACTATCTTTCGTTAAGTTGGGGATGTCCAGCATTGT-GGGTCCCTCGAAGTAGTCACCATCTGC
--GTGATTTCGTTGGAC--TACTCTGCAGAAGCGACGTCAAACAATTCTTAACCAGTACCTGAAATCAAGCAAGGCCACC
**>Metaradiophrya_chlorotica_JA2_1M_ACH**
CCTTAAGTTAGCGCTTCAGCAGCGGGAAACCGCTGCCG-TTATTTACCTTTCCAGCAACAACTTCTCTACACATTTACAAAACCCAAAA
ATTTTCAACGGTGGATATCTTGGTTCCCATTTCGATGAAGAACGCAGCAAAATGCGATAAGCAATGCGAATTGCAGAATTCCGCGAGTC
ATCAGATCTTTGAACGCAACTTGCGCTGGCGTAAAACCCAGCATGTTTGTTTCAGTGTGCA-GAAAA--GTCAACGACCTATCG--TGC
ATCTTAATGCGACCGAAGGACTT-CTCCTTCCTTCGTCAAACT-GGTCCGTCCGGCGATGTAGCACTCCTCGAAGTAGTCACATACTGC
TTGTGATTTCGTTGGGTCTTGCTTCGCCTTTGCGACGGCTTTT--TTCTTAGCCAGCACCTGAAATCAAGCAAGGCTACC
**>Metaradiophrya_chlorotica_JA2_2M_ACH**
CCTTAAGTTAGCGCTTCAGCAGCGGGAAACCGCTGCCG-TTATTTACCTTTCCAGCAACAACTTCTCTACACATTTACAAAACCCAAAA
ATTTTCAACGGTGGATATCTTGGTTCCCATTTCGATGAAGAACGCAGCAAAATGCGATAAGCAATGCGAATTGCAGAATTCCGCGAGTC
ATCAGATCTTTGAACGCAACTTGCGCTGGCGTAAAACCCAGCATGTTTGTTTCAGTGTGCA-GAAAA--GTCAACGACCTATCG--TGC
ATCTTAATGCGACCGAAGGACTT-CTCCTTCCTTCGTCAAACT-GGTCCGTCCGGCGATGTAGCACTCCTCGAAGTAGTCACATACTGC
TTGTGATTTCGTTGGGTCTTGCTTCGCCTTTGCGACGGCTTTT--TTCTTAGCCAGCACCTGAAATCAAGCAAGGCTACC
**>Metaradiophrya_chlorotica_JA2_3M_ACH**
CCTTAAGTTAGCGCTTCAGCAGCGGGAAACCGCTGCCG-TTATTTACCTTTCCAGCAACAACTTCTCTACACATTTACAAAACCCAAAA
ATTTTCAACGGTGGATATCTTGGTTCCCATTTCGATGAAGAACGCAGCAAAATGCGATAAGCAATGCGAATTGCAGAATTCCGCGAGTC
ATCAGATCTTTGAACGCAACTTGCGCTGGCGTAAAACCCAGCATGTTTGTTTCAGTGTGCA-GAAAA--GTCAACGACCTATCG--TGC
ATCTTAATGCGACCGAAGGACTT-CTCCTTCCTTCGTCAAACT-GGTCCGTCCGGCGATGTAGCACTCCTCGAAGTAGTCACATACTGC
TTGTGATTTCGTTGGGTCTTGCTTCGCCTTTGCGACGGCTTTT--TTCTTAGCCAGCACCTGAAATCAAGCAAGGCTACC
**>Metaradiophrya_lumbrici_RZ_4_LT**
CCTTAACTTAGCGTTTCAGCAGCGGGCAACCGCTGCCG-TTATTTACCTTTCTAGTTACACATCTCTATACCATATATCAAACCCAAAA

ATTTTCAACGGTGGATATCTTGGTTCCCATTTCGATGAAGAACGCAGCAAAATGCGATAAGCAATGCGAATTGCAGAATTCCGCGAGTC
ATCAGATCTTTGAACGCAACTTGCGCTGGCGTAAAAACCAGCATGTTTGTTTCAGTGTGCA-GAAAA--GTCAAAGACTTATCA--TTC
AACTTAATGCGACTGAAGG-----TCTCTTCTTTCGTCAAACT-GTTCCGTCCGGCGATGTAGCACCCCTCGAAGTAGTCACATATTGA
TTGTGATTTCGTTGGGTCTTGCTTTGCCTTTGCGACGGTTATT--TTCTTAGCCAGCACCTGAAATCAAGCAAGGCTACC
**>Metaradiophrya_lumbrici_RZ_5_LT**
CCTTAACTTAGCGTTTCAGCAGCGGGCAACCGCTGCCG-TTATTTACCTTTCTAGTTACACATCTCTATACCATATATCAAACCCAAAA
ATTTTCAACGGTGGATATCTTGGTTCCCATTTCGATGAAGAACGCAGCAAAATGCGATAAGCAATGCGAATTGCAGAATTCCGCGAGTC
ATCAGATCTTTGAACGCAACTTGCGCTGGCGTAAAAACCAGCATGTTTGTTTCAGTGTGCA-GAAAA--GTCAAAGACTTATCA--TTC
AACTTAATGCGACTGAAGG-----TCTCTTCTTTCGTCAAACT-GTTCCGTCCGGCGATGTAGCACCCCTCGAAGTAGTCACATATTGA
TTGTGATTTCGTTGGGTCTTGCTTTGCCTTTGCGACGGTTATT--TTCTTAGCCAGCACCTGAAATCAAGCAAGGCTACC
**>Metaradiophrya_lumbrici_KR_8_LT**
CCTTAACTTAGCGTTTCAGCAGCGGGCAACCGCTGCCG-TTATTTACCTTTCTAGTTACACATCTCTATACCATATATCAAACCCAAAA
ATTTTCAACGGTGGATATCTTGGTTCCCATTTCGATGAAGAACGCAGCAAAATGCGATAAGCAATGCGAATTGCAGAATTCCGCGAGTC
ATCAGATCTTTGAACGCAACTTGCGCTGGCGTAAAAACCAGCATGTTTGTTTCAGTGTGCA-GAAAA--GTCAAAGACTTATCA--TTC
AACTTAATGCGACTGAAGG-----TCTCTTCTTTCGTCAAACT-GTTCCGTCCGGCGATGTAGCACCCCTCGAAGTAGTCACATATTGA
TTGTGATTTCGTTGGGTCTTGCTTTGCCTTTGCGACGGTTATT--TTCTTAGCCAGCACCTGAAATCAAGCAAGGCTACC
**>Metaradiophrya_lumbrici_KR_10_LT**
CCTTAACTTAGCGTTTCAGCAGCGGGCAACCGCTGCCG-TTATTTACCTTTCTAGTTACACATCTCTATACCATATATCAAACCCAAAA
ATTTTCAACGGTGGATATCTTGGTTCCCATTTCGATGAAGAACGCAGCAAAATGCGATAAGCAATGCGAATTGCAGAATTCCGCGAGTC
ATCAGATCTTTGAACGCAACTTGCGCTGGCGTAAAAACCAGCATGTTTGTTTCAGTGTGCA-GAAAA--GTCAAAGACTTATCA--TTC
AACTTAATGCGACTGAAGG-----TCTCTTCTTTCGTCAAACT-GTTCCGTCCGGCGATGTAGCACCCCTCGAAGTAGTCACATATTGA
TTGTGATTTCGTTGGGTCTTGCTTTGCCTTTGCGACGGTTATT--TTCTTAGCCAGCACCTGAAATCAAGCAAGGCTACC
**>Metaradiophrya_lumbrici_JA2_25_LT**
CCTTAACTTAGCGTTTCAGCAGCGGGCAACCGCTGCCG-TTATTTACCTTTCTAGTTACACATCTCTATACCATATATCAAACCCAAAA
ATTTTCAACGGTGGATATCTTGGTTCCCATTTCGATGAAGAACGCAGCAAAATGCGATAAGCAATGCGAATTGCAGAATTCCGCGAGTC
ATCAGATCTTTGAACGCAACTTGCGCTGGCGTAAAAACCAGCATGTTTGTTTCAGTGTGCA-GAAAA--GTCAAAGACTTATCA--TTC
AACTTAATGCGACTGAAGG-----TCTCTTCTTTCGTCAAACT-GTTCCGTCCGGCGATGTAGCACCCCTCGAAGTAGTCACATATTGA
TTGTGATTTCGTTGGGTCTTGCTTTGCCTTTGCGACGGTTATT--TTCTTAGCCAGCACCTGAAATCAAGCAAGGCTACC
**>Metaradiophrya_lumbrici_JA2_26_LT**
CCTTAACTTAGCGTTTCAGCAGCGGGCAACCGCTGCCG-TTATTTACCTTTCTAGTTACACATCTCTATACCATATATCAAACCCAAAA
ATTTTCAACGGTGGATATCTTGGTTCCCATTTCGATGAAGAACGCAGCAAAATGCGATAAGCAATGCGAATTGCAGAATTCCGCGAGTC
ATCAGATCTTTGAACGCAACTTGCGCTGGCGTAAAAACCAGCATGTTTGTTTCAGTGTGCA-GAAAA--GTCAAAGACTTATCA--TTC
AACTTAATGCGACTGAAGG-----TCTCTTCTTTCGTCAAACT-GTTCCGTCCGGCGATGTAGCACCCCTCGAAGTAGTCACATATTGA
TTGTGATTTCGTTGGGTCTTGCTTTGCCTTTGCGACGGTTATT--TTCTTAGCCAGCACCTGAAATCAAGCAAGGCTACC
**>Metaradiophrya_speculorum_HkD_59_AT**
CCTTAATTTAGCGCTTCAGCAGCGGGAAACCGTTGCCG-TCATTTACTTTTTCAGAAAC----TTTCTCGAACTTTATCAAATCCAAAA
ATTTTCAACGGTGGATATCTTGGTTCCCATTTCGATGAAGAACGCAGCAAAATGCGATAAGCAATGCGAATTGCAGAATTCCGCGAGTC
ATCAGATCTTTGAACGCAACTTGCGCTGGCGTAAAAACCAGCATGTTTGTTTCAGTGTGCA-GAAAA--GTCAAAGACCAATCA--TCC
AACTTAATGCGACCGAAGGGT---TTCCTTCCTTCGTTAAACT-GTTCCGTCCGGCGATGTAGCACCCCTCGAAGTAGTCACATACCAC
TTGTGATTTCGTTGGGTCTTGCTTTGCCTTTGCGACGGATTTT--TTCTTAACCAGCACCTGAAATCAAGCAAGGCTACC
**>Metaradiophrya_speculorum_HkD_60_AT**
CCTTAATTTAGCGCTTCAGCAGCGGGAAACCGTTGCCG-TCATTTACTTTTTCAGAAAC----TTTCTCGAACTTTATCAAATCCAAAA
ATTTTCAACGGTGGATATCTTGGTTCCCATTTCGATGAAGAACGCAGCAAAATGCGATAAGCAATGCGAATTGCAGAATTCCGCGAGTC
ATCAGATCTTTGAACGCAACTTGCGCTGGCGTAAAAACCAGCATGTTTGTTTCAGTGTGCA-GAAAA--GTCAAAGACCAATCA--TCC
AACTTAATGCGACCGAAGGGT---TTCCTTCCTTCGTTAAACT-GTTCCGTCCGGCGATGTAGCACCCCTCGAAGTAGTCACATACCAC
TTGTGATTTCGTTGGGTCTTGCTTTGCCTTTGCGACGGATTTT--TTCTTAACCAGCACCTGAAATCAAGCAAGGCTACC
**>Metaradiophrya_varians_BZ_12_EF**
CCTTAATTTAGCGCTTCAGCAGCGGGAAACCGTTGCCGTTCATTTACCTTTCTAGAAAC----TTACTCGAACTTTATTAAATCCAAAA
ATTTTCAACGGTGGATATCTTGGTTCCCATTTCGATGAAGAACGCAGCAAAATGCGATAAGCAATGCGAATTGCAGAATTCCGCGAGTC
ATCAGATCTTTGAACGCAACTTGCGCTGGCGTAAAAACCAGCATGTTTGTTTCAGTGTGCA-AAAAA--GTCAAAGACCTATCG--TTA
AACTTAATGCGACCGAAGGACTTGTTCCTTCCTTCGTTAAACT-GTTCCGTCCGGTAATGTAGCACCCCTCGAAGTAGTCACATATTGA
TTGTGATTTCGTTGGGTCTTGCTTTGCCTTTGCGACGGTTATA--TTCTTAGCCAGCACCTGAAATCAAGCAAGGCTACC
**>Metaradiophrya_varians_BZ_14_EF**
CCTTAATTTAGCGCTTCAGCAGCGGGAAACCGTTGCCGTTCATTTACCTTTCTAGAAAC----TTACTCGAACTTTATTAAATCCAAAA
ATTTTCAACGGTGGATATCTTGGTTCCCATTTCGATGAAGAACGCAGCAAAATGCGATAAGCAATGCGAATTGCAGAATTCCGCGAGTC
ATCAGATCTTTGAACGCAACTTGCGCTGGCGTAAAAACCAGCATGTTTGTTTCAGTGTGCA-AAAAA--GTCAAAGACCTATCG--TTA
AACTTAATGCGACCGAAGGACTTGTTCCTTCCTTCGTTAAACT-GTTCCGTCCGGTAATGTAGCACCCCTCGAAGTAGTCACATATTGA
TTGTGATTTCGTTGGGTCTTGCTTTGCCTTTGCGACGGTTATA--TTCTTAGCCAGCACCTGAAATCAAGCAAGGCTACC
**>Metaradiophrya_varians_JA1_19_EF**
CCTTAATTTAGCGCTTCAGCAGCGGGAAACCGTTGCCGTTCATTTACCTTTCTAGAAAC----TTACTCGAACTTTATTAAATCCAAAA
ATTTTCAACGGTGGATATCTTGGTTCCCATTTCGATGAAGAACGCAGCAAAATGCGATAAGCAATGCGAATTGCAGAATTCCGCGAGTC
ATCAGATCTTTGAACGCAACTTGCGCTGGCGTAAAAACCAGCATGTTTGTTTCAGTGTGCA-AAAAA--GTCAAAGACCTATCG--TTA
AACTTAATGCGACCGAAGGACTTGTTCCTTCCTTCGTTAAACT-GTTCCGTCCGGTAATGTAGCACCCCTCGAAGTAGTCACATATTGA
TTGTGATTTCGTTGGGTCTTGCTTTGCCTTTGCGACGGTTATA--TTCTTAGCCAGCACCTGAAATCAAGCAAGGCTACC
**>Metaradiophrya_varians_JA1_22_EF**
CCTTAATTTAGCGCTTCAGCAGCGGGAAACCGTTGCCGTTCATTTACCTTTCTAGAAAC----TTACTCGAACTTTATTAAATCCAAAA
ATTTTCAACGGTGGATATCTTGGTTCCCATTTCGATGAAGAACGCAGCAAAATGCGATAAGCAATGCGAATTGCAGAATTCCGCGAGTC
ATCAGATCTTTGAACGCAACTTGCGCTGGCGTAAAAACCAGCATGTTTGTTTCAGTGTGCA-AAAAA--GTCAAAGACCTATCG--TTA
AACTTAATGCGACCGAAGGACTTGTTCCTTCCTTCGTTAAACT-GTTCCGTCCGGTAATGTAGCACCCCTCGAAGTAGTCACATATTGA
TTGTGATTTCGTTGGGTCTTGCTTTGCCTTTGCGACGGTTATA--TTCTTAGCCAGCACCTGAAATCAAGCAAGGCTACC

**>Metaradiophrya_varians_BZkv_31_EF**
CCTTAATTTAGCGCTTCAGCAGCGGGAAACCGTTGCCGTTCATTTACCTTTCTAGAAAC----TTACTCGAACTTTATTAAATCCAAAA
ATTTTCAACGGTGGATATCTTGGTTCCCATTTCGATGAAGAACGCAGCAAAATGCGATAAGCAATGCGAATTGCAGAATTCCGCGAGTC
ATCAGATCTTTGAACGCAACTTGCGCTGGCGTAAAAACCAGCATGTTTGTTTCAGTGTGCA-AAAAA--GTCAAAGACCTATCG--TTA
AACTTAATGCGACCGAAGGACTTGTTCCTTCCTTCGTTAAACT-GTTCCGTCCGGTAATGTAGCACCCCTCGAAGTAGTCACATATTGA
TTGTGATTTCGTTGGGTCTTGCTTTGCCTTTGCGACGGTTATA--TTCTTAGCCAGCACCTGAAATCAAGCAAGGCTACC
**>Metaradiophrya_varians_BZkv_32_EF**
CCTTAATTTAGCGCTTCAGCAGCGGGAAACCGTTGCCGTTCATTTACCTTTCTAGAAAC----TTACTCGAACTTTATTAAATCCAAAA
ATTTTCAACGGTGGATATCTTGGTTCCCATTTCGATGAAGAACGCAGCAAAATGCGATAAGCAATGCGAATTGCAGAATTCCGCGAGTC
ATCAGATCTTTGAACGCAACTTGCGCTGGCGTAAAAACCAGCATGTTTGTTTCAGTGTGCA-AAAAA--GTCAAAGACCTATCG--TTA
AACTTAATGCGACCGAAGGACTTGTTCCTTCCTTCGTTAAACT-GTTCCGTCCGGTAATGTAGCACCCCTCGAAGTAGTCACATATTGA
TTGTGATTTCGTTGGGTCTTGCTTTGCCTTTGCGACGGTTATA--TTCTTAGCCAGCACCTGAAATCAAGCAAGGCTACC
**>29Subanoplophrya_nodulata_PU_29_OT**
CCTTAAATTCGCTTTTT--TGGCATTTATGTTGCTGCA-TTATCAACTTTTTGAGTTAA--TTATTTTTGTAAACTTCAAAATTTAAAA
ATTTTCAACGGTGGATATCTAGGTTCCCATTTCGATGAAGAACGCAGCTAAATGCGATAAGCAATGCGAATTGCAGAATTCCGCGAGTC
ATCAGATCTTTGAACGCAACTTGCGCTGGTGTAAAAACCAGCATGTTTGTTTCAGTGTGCA-AAAAATAACAACAACTTAATCA--AAT
ATCTAAATGCGATTGAAGAAGCG-ATTTTTCTCTCGTCAAATTGGAGTCATCTAGCTTTGTGACTCCCTTCGAAGTAGTCACATTCAAA
ATGTGATTTCGTTGGGT-TCGCTCAGCTGATAAGATGACAATT--TTCTTAACTCGTACCTGAAATCAAGCAAGGCCACC
**>30Subanoplophrya_nodulata_PU_30_OT**
CCTTAAATTCGCTTTTT--TGGCATTTATGTTGCTGCA-TTATCAACTTTTTGAGTTAA--TTATTTTTGTAAACTTCAAAATTTAAAA
ATTTTCAACGGTGGATATCTAGGTTCCCATTTCGATGAAGAACGCAGCTAAATGCGATAAGCAATGCGAATTGCAGAATTCCGCGAGTC
ATCAGATCTTTGAACGCAACTTGCGCTGGTGTAAAAACCAGCATGTTTGTTTCAGTGTGCA-AAAAATAACAACAACTTAATCA--AAT
ATCTAAATGCGATTGAAGAAGCG-ATTTTTCTCTCGTCAAATTGGAGTCATCTAGCTTTGTGACTCCCTTCGAAGTAGTCACATTCAAA
ATGTGATTTCGTTGGGT-TCGCTCAGCTGATAAGATGACAATT—TTCTTAACTCGTACCTGAAATCAAGCAAGGCCACC

## D1/D2-28S rRNA gene alignment

**>Anoplophrya_allolobophorae_JA3_37_ACH**
CGCTGAACTTAAGCATATCAGTAAGCGGAGGAAAAGAAACTAACAAGGATTGCCCCAGTAGCGGCGAGTGAACAGGCAAAAGCTCAAAT
TGAAAATCCGTGGCACAAGGCTATGGAATTGTAATCTACAGAGGTTTTCGGAAGGCACGAGCTTCGCACAAGTTCCTTGGAACTGGACG
TCATAGAGGGTGAAAACCCCGTATACGGCGAGGCTTCGGCTGTACGAAAGCTTTCGAAGAGTCGGGTTGTTTGGTATTGCAGCCCTAAA
TGGGAGATAAACTTCTTCTAAAGCTAAATATCTATGGGAAACCGATAGCGAACAAGTACTGCGAAGGAAAGATGAAAAGAACTTTGAAA
AGAGGGTTAAAAGACTTGAAACCGTTGAAAAGGAAGCGGTAGAAGAGTAATGATACCAGTTGCGATGTACATGCTATTGGGCGGGCCTT
TGCGCGGCTTCGGACTTCATCGGACGAACTGCGTAACGAGCGACTACCCATTGCAGGTTTCTCGCAAATTGGTAGGCAAAATGAGGTTG
TGCTGGCGGAGTCAGTGGGCTGGAGGACCCTTCATCGGAGTCAGTCCACAAAACGGCGGCGCAGCCTGAGGGGCCTACGGGCGATTTTG
TCAAAATGCTTCTACTGACCCGTCTTGAAACACGGACCAAGGAGTCTACCCAGTATGCGAGTGTTAGGGCGGAAAAACCCGAACGCGCA
ACGAAAGTGATTAGATGCCAAGCCGCAA--GGCCGCAGCACCAGCCAACCCGATCTCTTTGA-GATGGGATTGAGCGGGAGCATATTAG
GTAGGACCCGAAAGATGGTGAACTACGCTTGAGTAGGGTGAAGCCAGGGGAAACTCTGGTGGAAGCTCGAAGCGATACTGACGTGCAAA
TCGTTCGTCAAACTTGAGTGTAGGGGCGAAAG
**>Anoplophrya_aporrectodeae_PUz_17_AT**
CGCTGAACTTAAGCATATCAGTAAGCGGAGGAAAAGAAACTAACAAGGATTGCCCCAGTAGCGGCGAGTGAACAGGCAAAAGCTCAAAT
TGAAAATCCGTGGCACAAGGCTATGGAATTGTAATCTACAGAGGTTTTCGGAAGGCACGAGCTTCGCACAAGTTCCTTGGAACTGGACG
TCATAGAGGGTGAAAACCCCGTATACGGCGAGGCTTCGGCTGTACGAAAGCTTTCGAAGAGTCGGGTTGTTTGGTATTGCAGCCCTAAA
TGGGAGATAAACTTCTTCTAAAGCTAAATATCTATGGGAAACCGATAGCGAACAAGTACTGCGAAGGAAAGATGAAAAGAACTTTGAAA
AGAGGGTTAAAAGACTTGAAACCGTTGAAAAGGAAGCGGTAGAAGAGTAATGATACCAGTTGCGATGTACATGCTATTGGGCGGGCCTT
TGCGTGGCTTCGGACTTCATCGGACGAACTGCGCAACGAGCGACCACCCATTGCAGGTTTCTCGCAAATTGGTAGGCAAAATGAGGTTG
TGCTGGCGGAGTCAGTGGGCTGGAGGACCCTTCATCGGAGTCAGTCCACAAAACGGCGGCGCAGCCTGAGGGGCCTACGGGCGATTTTG
TCAAAATGCTTCTACTGACCCGTCTTGAAACACGGACCAAGGAGTCTACCCAGTATGCGAGTGTTAGGGCGGAAAAACCCGAACGCGCA
ACGAAAGTGATTAGATGCCAAGCCGCAA--GGCCGCAGCACCAGCCAACCCGATCTCTTTGA-GATGGGATTGAGCGGGAGCATATTAG
GTAGGACCCGAAAGATGGTGAACTACGCTTGAGTAGGGTGAAGCCAGGGGAAACTCTGGTGGAAGCTCGAAGCGATACTGACGTGCAAA
TCGTTCGTCAAACTTGAGTGTAGGGGCGAAAG
**>Anoplophrya_aporrectodeae_PUz_40_AT**
CGCTGAACTTAAGCATATCAGTAAGCGGAGGAAAAGAAACTAACAAGGATTGCCCCAGTAGCGGCGAGTGAACAGGCAAAAGCTCAAAT
TGAAAATCCGTGGCACAAGGCTATGGAATTGTAATCTACAGAGGTTTTCGGAAGGCACGAGCTTCGCACAAGTTCCTTGGAACTGGACG
TCATAGAGGGTGAAAACCCCGTATACGGCGAGGCTTCGGCTGTACGAAAGCTTTCGAAGAGTCGGGTTGTTTGGTATTGCAGCCCTAAA
TGGGAGATAAACTTCTTCTAAAGCTAAATATCTATGGGAAACCGATAGCGAACAAGTACTGCGAAGGAAAGATGAAAAGAACTTTGAAA
AGAGGGTTAAAAGACTTGAAACCGTTGAAAAGGAAGCGGTAGAAGAGTAATGATACCAGTTGCGATGTACATGCTATTGGGCGGGCCTT
TGCGTGGCTTCGGACTTCATCGGACGAACTGCGCAACGAGCGACCACCCATTGCAGGTTTCTCGCAAATTGGTAGGCAAAATGAGGTTG
TGCTGGCGGAGTCAGTGGGCTGGAGGACCCTTCATCGGAGTCAGTCCACAAAACGGCGGCGCAGCCTGAGGGGCCTACGGGCGATTTTG
TCAAAATGCTTCTACTGACCCGTCTTGAAACACGGACCAAGGAGTCTACCCAGTATGCGAGTGTTAGGGCGGAAAAACCCGAACGCGCA
ACGAAAGTGATTAGATGCCAAGCCGCAA--GGCCGCAGCACCAGCCAACCCGATCTCTTTGA-GATGGGATTGAGCGGGAGCATATTAG
GTAGGACCCGAAAGATGGTGAACTACGCTTGAGTAGGGTGAAGCCAGGGGAAACTCTGGTGGAAGCTCGAAGCGATACTGACGTGCAAA
TCGTTCGTCAAACTTGAGTGTAGGGGCGAAAG
**>Anoplophrya_aporrectodeae_PUz_41_AT**
CGCTGAACTTAAGCATATCAGTAAGCGGAGGAAAAGAAACTAACAAGGATTGCCCCAGTAGCGGCGAGTGAACAGGCAAAAGCTCAAAT
TGAAAATCCGTGGCACAAGGCTATGGAATTGTAATCTACAGAGGTTTTCGGAAGGCACGAGCTTCGCACAAGTTCCTTGGAACTGGACG
TCATAGAGGGTGAAAACCCCGTATACGGCGAGGCTTCGGCTGTACGAAAGCTTTCGAAGAGTCGGGTTGTTTGGTATTGCAGCCCTAAA
TGGGAGATAAACTTCTTCTAAAGCTAAATATCTATGGGAAACCGATAGCGAACAAGTACTGCGAAGGAAAGATGAAAAGAACTTTGAAA
AGAGGGTTAAAAGACTTGAAACCGTTGAAAAGGAAGCGGTAGAAGAGTAATGATACCAGTTGCGATGTACATGCTATTGGGCGGGCCTT
TGCGTGGCTTCGGACTTCATCGGACGAACTGCGCAACGAGCGACCACCCATTGCAGGTTTCTCGCAAATTGGTAGGCAAAATGAGGTTG
TGCTGGCGGAGTCAGTGGGCTGGAGGACCCTTCATCGGAGTCAGTCCACAAAACGGCGGCGCAGCCTGAGGGGCCTACGGGCGATTTTG
TCAAAATGCTTCTACTGACCCGTCTTGAAACACGGACCAAGGAGTCTACCCAGTATGCGAGTGTTAGGGCGGAAAAACCCGAACGCGCA
ACGAAAGTGATTAGATGCCAAGCCGCAA--GGCCGCAGCACCAGCCAACCCGATCTCTTTGA-GATGGGATTGAGCGGGAGCATATTAG
GTAGGACCCGAAAGATGGTGAACTACGCTTGAGTAGGGTGAAGCCAGGGGAAACTCTGGTGGAAGCTCGAAGCGATACTGACGTGCAAA
TCGTTCGTCAAACTTGAGTGTAGGGGCGAAAG
**>Anoplophrya_lumbrici_RZ_6_LT**
CGCTGAACTTAAGCATATCAGTAAGCGGAGGAAAAGAAACTAACAAGGATTGCCCCAGTAGCGGCGAGTGAACAGGCAAAAGCTCAAAT
TGAAAATCCGTGGTACAAGGCTATGGAATTGTAATCTACAGAGGTTTTCGGAAAGCACGAGCTTCGCACAAGTTCCTTGGAACTGGACG
TCACAGAGGGTGAAAACCCCGTACACGGCGAAGCTTCGACTGTACGAAAGCTTTCGAAGAGTCGGGTTGTTTGGTATTGCAGCCCTAAA
TGGGAGATAAACTTCTTCTAAAGCTAAATATCTATGGGAAACCGATAGCGAACAAGTACTGCGAAGGAAAGATGAAAAGAACTTTGAAA
AGAGGGTTAAAAGACTTGAAACCGTTGAAAAGGAAGCGGTAGAAGAGTAATGATATCAGTTGCGATGTACATGCTATTGAGCGGGCCTT
TGTGTGGCTTCGGACTTCATCGGACGAACTGCGCAACGTGCGACCGATCATTGCAGGTTTCTCGCAAATTGATAGGCAAAATGAGGTTG
TGCTGACGGAGTCAGTGGGCTGGAGGACCCTTCATCGGAGTCAGTTCACAAAACGTTGGCGCAGCCTGAGGGGCCTACGGGCGATTTTG
TCAAAATGCTTCTACTGACCCGTCTTGAAACACGGACCAAGGAGTCTACCCAGTATGCGAGTGTTAGGGCGGAAAAACCCGAACGCGCA
ACGAAAGTGATTAGATGCCAAGCCGCAA--GGCCGCAGCACCAGCCAACCCGATCTCTTTGA-GATGGGATTGAGCGGGAGCATATTAG
GTAGGACCCGAAAGATGGTGAACTACGCTTGAGTAGGGTGAAGCCAGGGGAAACTCTGGTGGAAGCTCGAAGCGATACTGACGTGCAAA
TCGTTCGTCAAACTTGAGTGTAGGGGCGAAAG
**>Anoplophrya_lumbrici_KR_9_LT**
CGCTGAACTTAAGCATATCAGTAAGCGGAGGAAAAGAAACTAACAAGGATTGCCCCAGTAGCGGCGAGTGAACAGGCAAAAGCTCAAAT
TGAAAATCCGTGGTACAAGGCTATGGAATTGTAATCTACAGAGGTTTTCGGAAAGCACGAGCTTCGCACAAGTTCCTTGGAACTGGACG
TCACAGAGGGTGAAAACCCCGTACACGGCGAAGCTTCGACTGTACGAAAGCTTTCGAAGAGTCGGGTTGTTTGGTATTGCAGCCCTAAA
TGGGAGATAAACTTCTTCTAAAGCTAAATATCTATGGGAAACCGATAGCGAACAAGTACTGCGAAGGAAAGATGAAAAGAACTTTGAAA
AGAGGGTTAAAAGACTTGAAACCGTTGAAAAGGAAGCGGTAGAAGAGTAATGATATCAGTTGCGATGTACATGCTATTGAGCGGGCCTT
TGTGTGGCTTCGGACTTCATCGGACGAACTGCGCAACGTGCGACCGATCATTGCAGGTTTCTCGCAAATTGATAGGCAAAATGAGGTTG
TGCTGACGGAGTCAGTGGGCTGGAGGACCCTTCATCGGAGTCAGTTCACAAAACGTTGGCGCAGCCTGAGGGGCCTACGGGCGATTTTG
TCAAAATGCTTCTACTGACCCGTCTTGAAACACGGACCAAGGAGTCTACCCAGTATGCGAGTGTTAGGGCGGAAAAACCCGAACGCGCA

ACGAAAGTGATTAGATGCCAAGCCGCAA--GGCCGCAGCACCAGCCAACCCGATCTCTTTGA-GATGGGATTGAGCGGGAGCATATTAG
GTAGGACCCGAAAGATGGTGAACTACGCTTGAGTAGGGTGAAGCCAGGGGAAACTCTGGTGGAAGCTCGAAGCGATACTGACGTGCAAA
TCGTTCGTCAAACTTGAGTGTAGGGGCGAAAG
**>Anoplophrya_lumbrici_KR_11_LT**
CGCTGAACTTAAGCATATCAGTAAGCGGAGGAAAAGAAACTAACAAGGATTGCCCCAGTAGCGGCGAGTGAACAGGCAAAAGCTCAAAT
TGAAAATCCGTGGTACAAGGCTATGGAATTGTAATCTACAGAGGTTTTCGGAAAGCACGAGCTTCGCACAAGTTCCTTGGAACTGGACG
TCACAGAGGGTGAAAACCCCGTACACGGCGAAGCTTCGACTGTACGAAAGCTTTCGAAGAGTCGGGTTGTTTGGTATTGCAGCCCTAAA
TGGGAGATAAACTTCTTCTAAAGCTAAATATCTATGGGAAACCGATAGCGAACAAGTACTGCGAAGGAAAGATGAAAAGAACTTTGAAA
AGAGGGTTAAAAGACTTGAAACCGTTGAAAAGGAAGCGGTAGAAGAGTAATGATATCAGTTGCGATGTACATGCTATTGAGCGGGCCTT
TGTGTGGCTTCGGACTTCATCGGACGAACTGCGCAACGTGCGACCGATCATTGCAGGTTTCTCGCAAATTGATAGGCAAAATGAGGTTG
TGCTGACGGAGTCAGTGGGCTGGAGGACCCTTCATCGGAGTCAGTTCACAAAACGTTGGCGCAGCCTGAGGGGCCTACGGGCGATTTTG
TCAAAATGCTTCTACTGACCCGTCTTGAAACACGGACCAAGGAGTCTACCCAGTATGCGAGTGTTAGGGCGGAAAAACCCGAACGCGCA
ACGAAAGTGATTAGATGCCAAGCCGCAA--GGCCGCAGCACCAGCCAACCCGATCTCTTTGA-GATGGGATTGAGCGGGAGCATATTAG
GTAGGACCCGAAAGATGGTGAACTACGCTTGAGTAGGGTGAAGCCAGGGGAAACTCTGGTGGAAGCTCGAAGCGATACTGACGTGCAAA
TCGTTCGTCAAACTTGAGTGTAGGGGCGAAAG
**>Anoplophrya_octolasionis_MU_56_OL**
CGCTGAACTTAAGCATATCAGTAAGCGGAGGAAAAGAAACTAACAAGGATTGCCCCAGTAGCGGCGAGTGAACAGGCAAAAGCTCAAAT
TGAAAATCCGTGGCACAAGGCTATGGAATTGTAATCTACAGAGGTTTTCGGAAGGCACGAGCTTCGCACAAGTTCCTTGGAACTGGACG
TCATAGAGGGTGAAAACCCCGTATACGGCGAGGCTGCGGCTGTACGAAAGCTTTCGAAGAGTCGGGTTGTTTGGTATTGCAGCCCTAAA
TGGGAGATAAACTTCTTCTAAAGCTAAATATCTATGGGAAACCGATAGCGAACAAGTACTGCGAAGGAAAGATGAAAAGAACTTTGAAA
AGAGGGTTAAAAGACTTGAAACCGTTGAAAAGGAAGCGGTAGAAGAGTAATGATATCAGTTGCGATGTACATGCTATTGAGCGGGCCTT
TGCGCGGCTTCGGACTTCATCGGACGAACTGTGTAACGAGCGACCGAACATTGCGTGTTTCTCGCAAATTGGTAGGCAAAATGAGGTTG
TGCTGGCGGAGTCAGTGGACTGGAGGACCCTTCATCGGAGTCAGTTCACAAAACGTTGGCGCAGCCTGAGGGGCCTACGGGCGATTTTG
TCAAAATGCTTCTACTGACCCGTCTTGAAACACGGACCAAGGAGTCTACCCAGTATGCGAGTGTTAGGGTGGAAAAACCCGAACGCGCA
ACGAAAGTGATTAGATGCCAAGCCGCAA--GGCCGCAGCACCAGCCAACCCGATCTCTCTGA-GATGGGATTGAGCGGGAGCATATTAG
GTAGGACCCGAAAGATGGTGAACTACGCTTGAGTAGGGTGAAGCCAGGGGAAACTCTGGTGGAAGCTCGAAGCGATACTGACGTGCAAA
TCGTTCGTCAAACTTGAGTGTAGGGGCGAAAG
**>Anoplophrya_octolasionis_MU_57_OL**
CGCTGAACTTAAGCATATCAGTAAGCGGAGGAAAAGAAACTAACAAGGATTGCCCCAGTAGCGGCGAGTGAACAGGCAAAAGCTCAAAT
TGAAAATCCGTGGCACAAGGCTATGGAATTGTAATCTACAGAGGTTTTCGGAAGGCACGAGCTTCGCACAAGTTCCTTGGAACTGGACG
TCATAGAGGGTGAAAACCCCGTATACGGCGAGGCTGCGGCTGTACGAAAGCTTTCGAAGAGTCGGGTTGTTTGGTATTGCAGCCCTAAA
TGGGAGATAAACTTCTTCTAAAGCTAAATATCTATGGGAAACCGATAGCGAACAAGTACTGCGAAGGAAAGATGAAAAGAACTTTGAAA
AGAGGGTTAAAAGACTTGAAACCGTTGAAAAGGAAGCGGTAGAAGAGTAATGATATCAGTTGCGATGTACATGCTATTGAGCGGGCCTT
TGCGCGGCTTCGGACTTCATCGGACGAACTGTGTAACGAGCGACCGAACATTGCGTGTTTCTCGCAAATTGGTAGGCAAAATGAGGTTG
TGCTGGCGGAGTCAGTGGACTGGAGGACCCTTCATCGGAGTCAGTTCACAAAACGTTGGCGCAGCCTGAGGGGCCTACGGGCGATTTTG
TCAAAATGCTTCTACTGACCCGTCTTGAAACACGGACCAAGGAGTCTACCCAGTATGCGAGTGTTAGGGTGGAAAAACCCGAACGCGCA
ACGAAAGTGATTAGATGCCAAGCCGCAA--GGCCGCAGCACCAGCCAACCCGATCTCTCTGA-GATGGGATTGAGCGGGAGCATATTAG
GTAGGACCCGAAAGATGGTGAACTACGCTTGAGTAGGGTGAAGCCAGGGGAAACTCTGGTGGAAGCTCGAAGCGATACTGACGTGCAAA
TCGTTCGTCAAACTTGAGTGTAGGGGCGAAAG
**>Anoplophrya_octolasionis_MU_58_OL**
CGCTGAACTTAAGCATATCAGTAAGCGGAGGAAAAGAAACTAACAAGGATTGCCCCAGTAGCGGCGAGTGAACAGGCAAAAGCTCAAAT
TGAAAATCCGTGGCACAAGGCTATGGAATTGTAATCTACAGAGGTCTTCGGAAGGCACGAGCTTCGCACAAGTTCCTTGGAACTGGACG
TCATAGAGGGTGAAAACCCCGTATACGGCGAGGCTGCGGCTGTACGAAAGCTTTCGAAGAGTCGGGTTGTTTGGTATTGCAGCCCTAAA
TGGGAGATAAACTTCTTCTAAAGCTAAATATCTATGGGAAACCGATAGCGAACAAGTACTGCGAAGGAAAGATGAAAAGAACTTTGAAA
AGAGGGTTAAAAGACTTGAAACCGTTGAAAAGGAAGCGGTAGAAGAGTAATGATATCAGTTGCGATGTACATGCTATTGAGCGGGCCTT
TGCGCGGCTTCGGACTTCATCGGACGAACTGTGTAACGAGCGACCGAACATTGCGTGTTTCTCGCAAATTGGTAGGCAAAATGAGGTTG
TGCTGGCGGAGTCAGTGGACTGGAGGACCCTTCATCGGAGTCAGTTCACAAAACGTTGGCGCAGCCTGAGGGGCCTACGGGCGATTTTG
TCAAAATGCTTCTACTGACCCGTCTTGAAACACGGACCAAGGAGTCTACCCAGTATGCGAGTGTTAGGGTGGAAAAACCCGAACGCGCA
ACGAAAGTGATTAGATGCCAAGCCGCAA--GGCCGCAGCACCAGCCAACCCGATCTCTCTGA-GATGGGATTGAGCGGGAGCATATTAG
GTAGGACCCGAAAGATGGTGAACTACGCTTGAGTAGGGTGAAGCCAGGGGAAACTCTGGTGGAAGCTCGAAGCGATACTGACGTGCAAA
TCGTTCGTCAAACTTGAGTGTAGGGGCGAAAG
**>Anoplophrya_vulgaris_BZ_13_EF**
CGCTGAACTTAAGCATATCAGTAAGCGGAGGAAAAGAAACTAACAAGGATTGCCCCAGTAGCGGCGAGTGAACAGGCAAAAGCTCAAAT
TGAAAATCCGTGGCACAAGGCTATGGAATTGTAATCTACAGAGGTTTTCGGAAGGCACGAGCTGCGCACAAGTTCCTTGGAACTGGACG
TCACAGAGGGTGAAAACCCCGTACACAGCGCGGCT-CGGCTGTACGAAGGCTTTCGAAGAGTCGGGTTGTTTGGTATTGCAGCCCTAAA
TGGGAGATAAACTTCTTCTAAAGCTAAATATCTATGGGAAACCGATAGCGAACAAGTACTGCGAAGGAAAGATGAAAAGAACTTTGAAA
AGAGGGTTAAAAGACTTGAAACCGTTGAAAAGGAAGCGGTAGAAGAGCAATGATACCAGTTGCGCTGTGCGTGCTATTGGGCGGGCCTC
TGCGCGGCTTCGGACTTCATCGGACGAACTGTGCAGCGAGCGACTGTCCATTGCGCGTTTTTCGCAAATTGGTAGGCAAGGTGAGGTTG
TGCTGGCGGAGTCAGTGGGCCGAAAGACCCTTCATCGGAGTCGGTCCACAAAACGGCGGCGCAGCCTGAGGGGCCTACGGGCGACTTTG
TCAAAATGCTTCTACTGACCCGTCTTGAAACACGGACCAAGGAGTCTACCCAGTATGCGAGTGTTAGGGCGGAAAAACCCGAACGCGCA
ACGAAAGTGATTAGATGCCAAGCCGCAA--GGCCGCAGCACCAGCCAACCCGATCTCTTTGA-GATGGGATTGAGCGGGAGCATATTAG
GTAGGACCCGAAAGATGGTGAACTACGCTTGAGTAGGGTGAAGCCAGGGGAAACTCTGGTGGAAGCTCGAAGCGATACTGACGTGCAAA
TCGTTCGTCAAACTTGAGTGTAGGGGCGAAAG
**>Anoplophrya_vulgaris_JA1_18_EF**
CGCTGAACTTAAGCATATCAGTAAGCGGAGGAAAAGAAACTAACAAGGATTGCCCCAGTAGCGGCGAGTGAACAGGCAAAAGCTCAAAT
TGAAAATCCGTGGCACAAGGCTATGGAATTGTAATCTACAGAGGTTTTCGGAAGGCACGAGCTGCGCACAAGTTCCTTGGAACTGGACG
TCACAGAGGGTGAAAACCCCGTACACAGCGCGGCT-CGGCTGTACGAAGGCTTTCGAAGAGTCGGGTTGTTTGGTATTGCAGCCCTAAA
TGGGAGATAAACTTCTTCTAAAGCTAAATATCTATGGGAAACCGATAGCGAACAAGTACTGCGAAGGAAAGATGAAAAGAACTTTGAAA
AGAGGGTTAAAAGACTTGAAACCGTTGAAAAGGAAGCGGTAGAAGAGCAATGATACCAGTTGCGCTGTGCGTGCTATTGGGCGGGCCTC
TGCGCGGCTTCGGACTTCATCGGACGAACTGTGCAGCGAGCGACTGTCCATTGCGCGTTTTTCGCAAATTGGTAGGCAAGGTGAGGTTG
TGCTGGCGGAGTCAGTGGGCCGAAAGACCCTTCATCGGAGTCGGTCCACAAAACGGCGGCGCAGCCTGAGGGGCCTACGGGCGACTTTG

TCAAAATGCTTCTACTGACCCGTCTTGAAACACGGACCAAGGAGTCTACCCAGTATGCGAGTGTTAGGGCGGAAAAACCCGAACGCGCA
ACGAAAGTGATTAGATGCCAAGCCGCAA--GGCCGCAGCACCAGCCAACCCGATCTCTTTGA-GATGGGATTGAGCGGGAGCATATTAG
GTAGGACCCGAAAGATGGTGAACTACGCTTGAGTAGGGTGAAGCCAGGGGAAACTCTGGTGGAAGCTCGAAGCGATACTGACGTGCAAA
TCGTTCGTCAAACTTGAGTGTAGGGGCGAAAG

**>Anoplophrya_vulgaris_JA1_20_EF**

CGCTGAACTTAAGCATATCAGTAAGCGGAGGAAAAGAAACTAACAAGGATTGCCCCAGTAGCGGCGAGTGAACAGGCAAAAGCTCAAAT
TGAAAATCCGTGGCACAAGGCTATGGAATTGTAATCTACAGAGGTTTTCGGAAGGCACGAGCTGCGCACAAGTTCCTTGGAACTGGACG
TCACAGAGGGTGAAAACCCCGTACACAGCGCGGCT-CGGCTGTACGAAGGCTTTCGAAGAGTCGGGTTGTTTGGTATTGCAGCCCTAAA
TGGGAGATAAACTTCTTCTAAAGCTAAATATCTATGGGAAACCGATAGCGAACAAGTACTGCGAAGGAAAGATGAAAAGAACTTTGAAA
AGAGGGTTAAAAGACTTGAAACCGTTGAAAAGGAAGCGGTAGAAGAGCAATGATACCAGTTGCGCTGTGCGTGCTATTGGGCGGGCCTC
TGCGCGGCTTCGGACTTCATCGGACGAACTGTGCAGCGAGCGACTGTCCATTGCGCGTTTTTCGCAAATTGGTAGGCAAGGTGAGGTTG
TGCTGGCGGAGTCAGTGGGCCGAAAGACCCTTCATCGGAGTCGGTCCACAAAACGGCGGCGCAGCCTGAGGGGCCTACGGGCGACTTTG
TCAAAATGCTTCTACTGACCCGTCTTGAAACACGGACCAAGGAGTCTACCCAGTATGCGAGTGTTAGGGCGGAAAAACCCGAACGCGCA
ACGAAAGTGATTAGATGCCAAGCCGCAA--GGCCGCAGCACCAGCCAACCCGATCTCTTTGA-GATGGGATTGAGCGGGAGCATATTAG
GTAGGACCCGAAAGATGGTGAACTACGCTTGAGTAGGGTGAAGCCAGGGGAAACTCTGGTGGAAGCTCGAAGCGATACTGACGTGCAAA
TCGTTCGTCAAACTTGAGTGTAGGGGCGAAAG

**>Anoplophrya_vulgaris_JA1_21_EF**

CGCTGAACTTAAGCATATCAGTAAGCGGAGGAAAAGAAACTAACAAGGATTGCCCCAGTAGCGGCGAGTGAACAGGCAAAAGCTCAAAT
TGAAAATCCGTGGCACAAGGCTATGGAATTGTAATCTACAGAGGTTTTCGGAAGGCACGAGCTGCGCACAAGTTCCTTGGAACTGGACG
TCACAGAGGGTGAAAACCCCGTACACAGCGCGGCT-CGGCTGTACGAAGGCTTTCGAAGAGTCGGGTTGTTTGGTATTGCAGCCCTAAA
TGGGAGATAAACTTCTTCTAAAGCTAAATATCTATGGGAAACCGATAGCGAACAAGTACTGCGAAGGAAAGATGAAAAGAACTTTGAAA
AGAGGGTTAAAAGACTTGAAACCGTTGAAAAGGAAGCGGTAGAAGAGCAATGATACCAGTTGCGCTGTGCGTGCTATTGGGCGGGCCTC
TGCGCGGCTTCGGACTTCATCGGACGAACTGTGCAGCGAGCGACTGTCCATTGCGCGTTTTTCGCAAATTGGTAGGCAAGGTGAGGTTG
TGCTGGCGGAGTCAGTGGGCCGAAAGACCCTTCATCGGAGTCGGTCCACAAAACGGCGGCGCAGCCTGAGGGGCCTACGGGCGACTTTG
TCAAAATGCTTCTACTGACCCGTCTTGAAACACGGACCAAGGAGTCTACCCAGTATGCGAGTGTTAGGGCGGAAAAACCCGAACGCGCA
ACGAAAGTGATTAGATGCCAAGCCGCAA--GGCCGCAGCACCAGCCAACCCGATCTCTTTGA-GATGGGATTGAGCGGGAGCATATTAG
GTAGGACCCGAAAGATGGTGAACTACGCTTGAGTAGGGTGAAGCCAGGGGAAACTCTGGTGGAAGCTCGAAGCGATACTGACGTGCAAA
TCGTTCGTCAAACTTGAGTGTAGGGGCGAAAG

**>Anoplophrya_vulgaris_NG_27_DV**

CGCTGAACTTAAGCATATCAGTAAGCGGAGGAAAAGAAACTAACAAGGATTGCCCCAGTAGCGGCGAGTGAACAGGCAAAAGCTCAAAT
TGAAAATCCGTGGCACAAGGCTATGGAATTGTAATCTACAGAGGTTTTCGGAAGGCACGAGCTGCGCACAAGTTCCTTGGAACTGGACG
TCACAGAGGGTGAAAACCCCGTACACAGCGCGGCT-CGGCTGTACGAAGGCTTTCGAAGAGTCGGGTTGTTTGGTATTGCAGCCCTAAA
TGGGAGATAAACTTCTTCTAAAGCTAAATATCTATGGGAAACCGATAGCGAACAAGTACTGCGAAGGAAAGATGAAAAGAACTTTGAAA
AGAGGGTTAAAAGACTTGAAACCGTTGAAAAGGAAGCGGTAGAAGAGCAATGATACCAGTTGCGCTGTGCGTGCTATTGGGCGGGCCTC
TGCGCGGCTTCGGACTTCATCGGACGAACTGTGCAGCGAGCGACTGTCCATTGCGCGTTTTTCGCAAATTGGTAGGCAAGGTGAGGTTG
TGCTGGCGGAGTCAGTGGGCCGAAAGACCCTTCATCGGAGTCGGTCCACAAAACGGCGGCGCAGCCTGAGGGGCCTACGGGCGACTTTG
TCAAAATGCTTCTACTGACCCGTCTTGAAACACGGACCAAGGAGTCTACCCAGTATGCGAGTGTTAGGGCGGAAAAACCCGAACGCGCA
ACGAAAGTGATTAGATGCCAAGCCGCAA--GGCCGCAGCACCAGCCAACCCGATCTCTTTGA-GATGGGATTGAGCGGGAGCATATTAG
GTAGGACCCGAAAGATGGTGAACTACGCTTGAGTAGGGTGAAGCCAGGGGAAACTCTGGTGGAAGCTCGAAGCGATACTGACGTGCAAA
TCGTTCGTCAAACTTGAGTGTAGGGGCGAAAG

**>Anoplophrya_vulgaris_NG_28_DV**

CGCTGAACTTAAGCATATCAGTAAGCGGAGGAAAAGAAACTAACAAGGATTGCCCCAGTAGCGGCGAGTGAACAGGCAAAAGCTCAAAT
TGAAAATCCGTGGCACAAGGCTATGGAATTGTAATCTACAGAGGTTTTCGGAAGGCACGAGCTGCGCACAAGTTCCTTGGAACTGGACG
TCACAGAGGGTGAAAACCCCGTACACAGCGCGGCT-CGGCTGTACGAAGGCTTTCGAAGAGTCGGGTTGTTTGGTATTGCAGCCCTAAA
TGGGAGATAAACTTCTTCTAAAGCTAAATATCTATGGGAAACCGATAGCGAACAAGTACTGCGAAGGAAAGATGAAAAGAACTTTGAAA
AGAGGGTTAAAAGACTTGAAACCGTTGAAAAGGAAGCGGTAGAAGAGCAATGATACCAGTTGCGCTGTGCGTGCTATTGGGCGGGCCTC
TGCGCGGCTTCGGACTTCATCGGACGAACTGTGCAGCGAGCGACTGTCCATTGCGCGTTTTTCGCAAATTGGTAGGCAAGGTGAGGTTG
TGCTGGCGGAGTCAGTGGGCCGAAAGACCCTTCATCGGAGTCGGTCCACAAAACGGCGGCGCAGCCTGAGGGGCCTACGGGCGACTTTG
TCAAAATGCTTCTACTGACCCGTCTTGAAACACGGACCAAGGAGTCTACCCAGTATGCGAGTGTTAGGGCGGAAAAACCCGAACGCGCA
ACGAAAGTGATTAGATGCCAAGCCGCAA--GGCCGCAGCACCAGCCAACCCGATCTCTTTGA-GATGGGATTGAGCGGGAGCATATTAG
GTAGGACCCGAAAGATGGTGAACTACGCTTGAGTAGGGTGAAGCCAGGGGAAACTCTGGTGGAAGCTCGAAGCGATACTGACGTGCAAA
TCGTTCGTCAAACTTGAGTGTAGGGGCGAAAG

**>Maupasella_mucronata_KDo_33_ET**

CGCTGAACTTAAGCATATCAGTAAGCGGAGGAAAAGAAACTAACAAGGATTGCCTCAGTAGCGGCGAGTGAACAGGCAAAAGCTCACAT
TGAAAATCCTTAGCATA-GGCTTCGGAATTGTAATCTGTAGAAGTTCTCGGAAGGCTAGGACCTCGCACAAGTTCCAAGGAACGGGACG
TCAGAGAGGGTGATAACCCCGTACATGGTGAGGGACCGGCCGTACGAGTGCTTTCCAAGAGTCGGGTTGTTTGGTATTGCAGCCCTAAG
TGGGAGATAAACTTCTTCTAAAGCTAAATATTTATGGGAAACCGATAGCGAACAAGTACTGCGAAGGAAAGATGAAAAGAACTTTGAAA
ATTGTGTTAAAAGACTTGAAACCGTTGGAAAGGAAGCGGTAGAAGAGTAATGGTACCATCGGTGACCTCAGTGCTCTAGCACAGGCTCC
CTTG-GGTTCTAGATTTCATCAACTAGGGCCTTTGGTCAGTCACTGGGCTTTGCATGTTCGTCGCGGAGTGGTAGACAAAATCGGATTG
TACTGCCGGAATCAGTGTTTCGAAAGACCCTTTACCGGAATCGAGACACAAAACGGCGGCGCAGTCCGAGGGGCCTACGGGCGATTTTG
TCAAAATGCTTCTACCGACCCGTCTTGAAACACGGACCAAGGAGTCTACCCAGTATGCGAGTGTTAGAGTGTAGAAACTCGAACGCGTA
GCGAAAGCGACAAGATGCGCCGCCTTTACTGGTCTCAGCACCGACCAGCGCTGACTCTTTGAGGAAGTGACTGAGTTGGAGCATATTAG
GTAGGACCCGAAAGATGGTGAACTACACTTGAGTAGGGTGAAGCCAGGGGAAACTCTGGTGGAGGCTCGCAGCGATACTGACGTGCAAA
TCGTTCGTCAAACTTGAGTGTAGGGGCGAAAG

**>Maupasella_mucronata_KDo_34_ET**

CGCTGAACTTAAGCATATCAGTAAGCGGAGGAAAAGAAACTAACAAGGATTGCCTCAGTAGCGGCGAGTGAACAGGCAAAAGCTCACAT
TGAAAATCCTTAGCATA-GGCTTCGGAATTGTAATCTGTAGAAGTTCTCGGAAGGCTAGGACCTCGCACAAGTTCCAAGGAACGGGACG
TCAGAGAGGGTGATAACCCCGTACATGGTGAGGGACCGGCCGTACGAGTGCTTTCCAAGAGTCGGGTTGTTTGGTATTGCAGCCCTAAG
TGGGAGATAAACTTCTTCTAAAGCTAAATATTTATGGGAAACCGATAGCGAACAAGTACTGCGAAGGAAAGATGAAAAGAACTTTGAAA
ATTGTGTTAAAAGACTTGAAACCGTTGGAAAGGAAGCGGTAGAAGAGTAATGGTACCATCGGTGACCTCAGTGCTCTAGCACAGGCTCC
CTTG-GGTTCTAGATTTCATCAACTAGGGCCTTTGGTCAGTCACTGGGCTTTGCATGTTCGTCGCGGAGTGGTAGACAAAATCGGATTG

TACTGCCGGAATCAGTGTTTCGAAAGACCCTTTACCGGAATCGAGACACAAAACGGCGGCGCAGTCCGAGGGGCCTACGGGCGATTTTG
TCAAAATGCTTCTACCGACCCGTCTTGAAACACGGACCAAGGAGTCTACCCAGTATGCGAGTGTTAGAGTGTAGAAACTCGAACGCGTA
GCGAAAGCGACAAGATGCGCCGCCTTTACTGGTCTCAGCACCGACCAGCGCTGACTCTTTGAGGAAGTGACTGAGTTGGAGCATATTAG
GTAGGACCCGAAAGATGGTGAACTACACTTGAGTAGGGTGAAGCCAGGGGAAACTCTGGTGGAGGCTCGCAGCGATACTGACGTGCAAA
TCGTTCGTCAAACTTGAGTGTAGGGGCGAAAG
**>Maupasella_mucronata_KDo_35_ET**
CGCTGAACTTAAGCATATCAGTAAGCGGAGGAAAAGAAACTAACAAGGATTGCCTCAGTAGCGGCGAGTGAACAGGCAAAAGCTCACAT
TGAAAATCCTTAGCATA-GGCTTCGGAATTGTAATCTGTAGAAGTTCTCGGAAGGCTAGGACCTCGCACAAGTTCCAAGGAACGGGACG
TCAGAGAGGGTGATAACCCCGTACATGGTGAGGGACCGGCCGTACGAGTGCTTTCCAAGAGTCGGGTTGTTTGGTATTGCAGCCCTAAG
TGGGAGATAAACTTCTTCTAAAGCTAAATATTTATGGGAAACCGATAGCGAACAAGTACTGCGAAGGAAAGATGAAAAGAACTTTGAAA
ATTGTGTTAAAAGACTTGAAACCGTTGGAAAGGAAGCGGTAGAAGAGTAATGGTACCATCGGTGACCTCAGTGCTCTAGCACAGGCTCC
CTTG-GGTTCTAGATTTCATCAACTAGGGCCTTTGGTCAGTCACTGGGCTTTGCATGTTCGTCGCGGAGTGGTAGACAAAATCGGATTG
TACTGCCGGAATCAGTGTTTCGAAAGACCCTTTACCGGAATCGAGACACAAAACGGCGGCGCAGTCCGAGGGGCCTACGGGCGATTTTG
TCAAAATGCTTCTACCGACCCGTCTTGAAACACGGACCAAGGAGTCTACCCAGTATGCGAGTGTTAGAGTGTAGAAACTCGAACGCGTA
GCGAAAGCGACAAGATGCGCCGCCTTTACTGGTCTCAGCACCGACCAGCGCTGACTCTTTGAGGAAGTGACTGAGTTGGAGCATATTAG
GTAGGACCCGAAAGATGGTGAACTACACTTGAGTAGGGTGAAGCCAGGGGAAACTCTGGTGGAGGCTCGCAGCGATACTGACGTGCAAA
TCGTTCGTCAAACTTGAGTGTAGGGGCGAAAG
**>Maupasella_mucronata_KDo_36_ET**
CGCTGAACTTAAGCATATCAGTAAGCGGAGGAAAAGAAACTAACAAGGATTGCCTCAGTAGCGGCGAGTGAACAGGCAAAAGCTCACAT
TGAAAATCCTTAGCATA-GGCTTCGGAATTGTAATCTGTAGAAGTTCTCGGAAGGCTAGGACCTCGCACAAGTTCCAAGGAACGGGACG
TCAGAGAGGGTGATAACCCCGTACATGGTGAGGGACCGGCCGTACGAGTGCTTTCCAAGAGTCGGGTTGTTTGGTATTGCAGCCCTAAG
TGGGAGATAAACTTCTTCTAAAGCTAAATATTTATGGGAAACCGATAGCGAACAAGTACTGCGAAGGAAAGATGAAAAGAACTTTGAAA
ATTGTGTTAAAAGACTTGAAACCGTTGGAAAGGAAGCGGTAGAAGAGTAATGGTACCATCGGTGACCTCAGTGCTCTAGCACAGGCTCC
CTTG-GGTTCTAGATTTCATCAACTAGGGCCTTTGGTCAGTCACTGGGCTTTGCATGTTCGTCGCGGAGTGGTAGACAAAATCGGATTG
TACTGCCGGAATCAGTGTTTCGAAAGACCCTTTACCGGAATCGAGACACAAAACGGCGGCGCAGTCCGAGGGGCCTACGGGCGATTTTG
TCAAAATGCTTCTACCGACCCGTCTTGAAACACGGACCAAGGAGTCTACCCAGTATGCGAGTGTTAGAGTGTAGAAACTCGAACGCGTA
GCGAAAGCGACAAGATGCGCCGCCTTTACTGGTCTCAGCACCGACCAGCGCTGACTCTTTGAGGAAGTGACTGAGTTGGAGCATATTAG
GTAGGACCCGAAAGATGGTGAACTACACTTGAGTAGGGTGAAGCCAGGGGAAACTCTGGTGGAGGCTCGCAGCGATACTGACGTGCAAA
TCGTTCGTCAAACTTGAGTGTAGGGGCGAAAG
**>Metaradiophrya_chlorotica_JA2_1M_ACH**
CGCTGAACTTAAGCATATCAGTAAGCGGAGGAAAAGAAACTAACAAGGATTGCTCCAGTAGCGGCGAGTGAACAGGCAAAAGCTCAAAT
TGAAAATCCGTGGCACATGGCTATGGAATTGTAATCTATAGAGGTTTTCGGAAGGTACGAGTTTCGCACAAGTTCCTTGGAACTGGACG
TCATAGAGGGTGAAAACCCCGTACACGGCGAGACTTCGACCGTACGAAGGCTTTCGAAGAGTCGGGTTGTTTGGTATTGCAGCCCTAAA
TGGGAGATAAACTTCTTCTAAAGCTAAATATCTATGGGAAACCGATAGCGAACAAGTACTGCGAAGGAAAGATGAAAAGAACTTTGAAA
AGAGGGTTAAAAGACTTGAAACCGTTGAAAAGGAAGCGGTAGAAGAGTAATGATACCAGTTGCGATATACCTGTTCTAGTGCGGACCCT
TGTGTGGTTTCGGACTTCATCGGACGGGCTATGCAATGCGTGACTGTGCTTTGCAGGTTTTTCGCAAATTGGTAGGCAAAATGAGGTTG
TGCCGGCGGAGTCAGTGGACTGGAAGACCCTTCATCGGAGCCAGTCCACAAAACGGCGGCGCAGCCTGAGGGGCCTACGGGCGATTTTG
TCAAAATGCTTCTACTGACCCGTCTTGAAACACGGACCAAGGAGTCTACCCAGTATGCGAGTGTTAGGGCGGAAAAACCCGAACGCGCA
ACGAAAGTGATTAGATGCCAAGCCGCAA--GGCCGCAGCACCAGCCAACCTTGACTCTCTGAGGAAGGGATTGAGCGGGAGCATATTAG
GTAGGACCCGAAAGATGGTGAACTACGCTTGAGTAGGGTGAAGCCAGGGGAAACTCTGGTGGAAGCTCGAAGCGATACTGACGTGCAAA
TCGTTCGTCAAACTTGAGTGTAGGGGCGAAAG
**>Metaradiophrya_chlorotica_JA2_2M_ACH**
CGCTGAACTTAAGCATATCAGTAAGCGGAGGAAAAGAAACTAACAAGGATTGCTCCAGTAGCGGCGAGTGAACAGGCAAAAGCTCAAAT
TGAAAATCCGTGGCACATGGCTATGGAATTGTAATCTATAGAGGTTTTCGGAAGGTACGAGTTTCGCACAAGTTCCTTGGAACTGGACG
TCATAGAGGGTGAAAACCCCGTACACGGCGAGACTTCGACCGTACGAAGGCTTTCGAAGAGTCGGGTTGTTTGGTATTGCAGCCCTAAA
TGGGAGATAAACTTCTTCTAAAGCTAAATATCTATGGGAAACCGATAGCGAACAAGTACTGCGAAGGAAAGATGAAAAGAACTTTGAAA
AGAGGGTTAAAAGACTTGAAACCGTTGAAAAGGAAGCGGTAGAAGAGTAATGATACCAGTTGCGATATACCTGTTCTAGTGCGGACCCT
TGTGTGGTTTCGGACTTCATCGGACGGGCTATGCAATGCGTGACTGTGCTTTGCAGGTTTTTCGCAAATTGGTAGGCAAAATGAGGTTG
TGCCGGCGGAGTCAGTGGACTGGAAGACCCTTCATCGGAGCCAGTCCACAAAACGGCGGCGCAGCCTGAGGGGCCTACGGGCGATTTTG
TCAAAATGCTTCTACTGACCCGTCTTGAAACACGGACCAAGGAGTCTACCCAGTATGCGAGTGTTAGGGCGGAAAAACCCGAACGCGCA
ACGAAAGTGATTAGATGCCAAGCCGCAA--GGCCGCAGCACCAGCCAACCTTGACTCTCTGAGGAAGGGATTGAGCGGGAGCATATTAG
GTAGGACCCGAAAGATGGTGAACTACGCTTGAGTAGGGTGAAGCCAGGGGAAACTCTGGTGGAAGCTCGAAGCGATACTGACGTGCAAA
TCGTTCGTCAAACTTGAGTGTAGGGGCGAAAG
**>Metaradiophrya_chlorotica_JA2_3M_ACH**
CGCTGAACTTAAGCATATCAGTAAGCGGAGGAAAAGAAACTAACAAGGATTGCTCCAGTAGCGGCGAGTGAACAGGCAAAAGCTCAAAT
TGAAAATCCGTGGCACATGGCTATGGAATTGTAATCTATAGAGGTTTTCGGAAGGTACGAGTTTCGCACAAGTTCCTTGGAACTGGACG
TCATAGAGGGTGAAAACCCCGTACACGGCGAGACTTCGACCGTACGAAGGCTTTCGAAGAGTCGGGTTGTTTGGTATTGCAGCCCTAAA
TGGGAGATAAACTTCTTCTAAAGCTAAATATCTATGGGAAACCGATAGCGAACAAGTACTGCGAAGGAAAGATGAAAAGAACTTTGAAA
AGAGGGTTAAAAGACTTGAAACCGTTGAAAAGGAAGCGGTAGAAGAGTAATGATACCAGTTGCGATATACCTGTTCTAGTGCGGACCCT
TGTGTGGTTTCGGACTTCATCGGACGGGCTATGCAATGCGTGACTGTGCTTTGCAGGTTTTTCGCAAATTGGTAGGCAAAATGAGGTTG
TGCCGGCGGAGTCAGTGGACTGGAAGACCCTTCATCGGAGCCAGTCCACAAAACGGCGGCGCAGCCTGAGGGGCCTACGGGCGATTTTG
TCAAAATGCTTCTACTGACCCGTCTTGAAACACGGACCAAGGAGTCTACCCAGTATGCGAGTGTTAGGGCGGAAAAACCCGAACGCGCA
ACGAAAGTGATTAGATGCCAAGCCGCAA--GGCCGCAGCACCAGCCAACCTTGACTCTCTGAGGAAGGGATTGAGCGGGAGCATATTAG
GTAGGACCCGAAAGATGGTGAACTACGCTTGAGTAGGGTGAAGCCAGGGGAAACTCTGGTGGAAGCTCGAAGCGATACTGACGTGCAAA
TCGTTCGTCAAACTTGAGTGTAGGGGCGAAAG
**>Metaradiophrya_lumbrici_RZ_4_LT**
CGCTGAACTTAAGCATATCAGTAAGCGGAGGAAAAGAAACTAACAAGGATTGCTCCAGTAGCGGCGAGTGAACAGGCAAAAGCTCAAAT
TGAAAATCCGTGGCACA-AGCTATGGAATTGTAATCTATAGAGGTCTTCGGAAAGTACGAGTTTCGCACAAGTTCCTTGGAACTGGACG
TCATAGAGGGTGAAAACCCCGTACATGGCGAGACTTCGACTGTACGAAGGCTTTCGAAGAGTCGGGTTGTTTGGTATTGCAGCCCTAAA
TGGGAGATAAACTTCTTCTAAAGCTAAATATCTATGGGAAACCGATAGCGAACAAGTACTGCGAAGGAAAGATGAAAAGAACTTTGAAA
AGAGGGTTAAAAGACTTGAAACCGTTGAAAAGGAAGCGGTAGAAGAGTAATGATATCAGTTGCGATATACCTGCTATAGTGCGGACCCT

TGGGTCGTTTCGGACTTCATCGGACGGGCGATTTAATGCGTGACTGTGCTTTGTAGGTTTTTCGCAAATTGGTAGGCAAAATGAGGTTG
TGCAGGCGGAGTCAGTGGACTGGAGGACCCTTCATCGGAGTCAGTTCACAAAACGTCGGCGCAGCCTGAGGGGCCTACGGGCGATTTTG
TCAAAATGCTTCTACTGACCCGTCTTGAAACACGGACCAAGGAGTCTACCCAGTATGCGAGTGTTAGGGTGGAAAAACCCGAACGCGCA
ACGAAAGTGATTAGATGCCAAGCCGCAA--GGCCGCAGCACCAGCCAACCTTGACTCTTTGAGGAAGGGATTGAGCGGGAGCATATTAG
GTAGGACCCGAAAGATGGTGAACTACGCTTGAGTAGGGTGAAGCCAGGGGAAACTCTGGTGGAAGCTCGAAGCGATACTGACGTGCAAA
TCGTTCGTCAAACTTGAGTGTAGGGGCGAAAG

**>Metaradiophrya_lumbrici_RZ_5_LT**

CGCTGAACTTAAGCATATCAGTAAGCGGAGGAAAAGAAACTAACAAGGATTGCTCCAGTAGCGGCGAGTGAACAGGCAAAAGCTCAAAT
TGAAAATCCGTGGCACA-AGCTATGGAATTGTAATCTATAGAGGTCTTCGGAAAGTACGAGTTTCGCACAAGTTCCTTGGAACTGGACG
TCATAGAGGGTGAAAACCCCGTACATGGCGAGACTTCGACTGTACGAAGGCTTTCGAAGAGTCGGGTTGTTTGGTATTGCAGCCCTAAA
TGGGAGATAAACTTCTTCTAAAGCTAAATATCTATGGGAAACCGATAGCGAACAAGTACTGCGAAGGAAAGATGAAAAGAACTTTGAAA
AGAGGGTTAAAAGACTTGAAACCGTTGAAAAGGAAGCGGTAGAAGAGTAATGATATCAGTTGCGATATACCTGCTATAGTGCGGACCCT
TGGGTCGTTTCGGACTTCATCGGACGGGCGATTTAATGCGTGACTGTGCTTTGTAGGTTTTTCGCAAATTGGTAGGCAAAATGAGGTTG
TGCAGGCGGAGTCAGTGGACTGGAGGACCCTTCATCGGAGTCAGTTCACAAAACGTCGGCGCAGCCTGAGGGGCCTACGGGCGATTTTG
TCAAAATGCTTCTACTGACCCGTCTTGAAACACGGACCAAGGAGTCTACCCAGTATGCGAGTGTTAGGGTGGAAAAACCCGAACGCGCA
ACGAAAGTGATTAGATGCCAAGCCGCAA--GGCCGCAGCACCAGCCAACCTTGACTCTTTGAGGAAGGGATTGAGCGGGAGCATATTAG
GTAGGACCCGAAAGATGGTGAACTACGCTTGAGTAGGGTGAAGCCAGGGGAAACTCTGGTGGAAGCTCGAAGCGATACTGACGTGCAAA
TCGTTCGTCAAACTTGAGTGTAGGGGCGAAAG

**>Metaradiophrya_lumbrici_KR_8_LT**

CGCTGAACTTAAGCATATCAGTAAGCGGAGGAAAAGAAACTAACAAGGATTGCTCCAGTAGCGGCGAGTGAACAGGCAAAAGCTCAAAT
TGAAAATCCGTGGCACA-AGCTATGGAATTGTAATCTATAGAGGTCTTCGGAAAGTACGAGTTTCGCACAAGTTCCTTGGAACTGGACG
TCATAGAGGGTGAAAACCCCGTACATGGCGAGACTTCGACTGTACGAAGGCTTTCGAAGAGTCGGGTTGTTTGGTATTGCAGCCCTAAA
TGGGAGATAAACTTCTTCTAAAGCTAAATATCTATGGGAAACCGATAGCGAACAAGTACTGCGAAGGAAAGATGAAAAGAACTTTGAAA
AGAGGGTTAAAAGACTTGAAACCGTTGAAAAGGAAGCGGTAGAAGAGTAATGATATCAGTTGCGATATACCTGCTATAGTGCGGACCCT
TGGGTCGTTTCGGACTTCATCGGACGGGCGATTTAATGCGTGACTGTGCTTTGTAGGTTTTTCGCAAATTGGTAGGCAAAATGAGGTTG
TGCAGGCGGAGTCAGTGGACTGGAGGACCCTTCATCGGAGTCAGTTCACAAAACGTCGGCGCAGCCTGAGGGGCCTACGGGCGATTTTG
TCAAAATGCTTCTACTGACCCGTCTTGAAACACGGACCAAGGAGTCTACCCAGTATGCGAGTGTTAGGGTGGAAAAACCCGAACGCGCA
ACGAAAGTGATTAGATGCCAAGCCGCAA--GGCCGCAGCACCAGCCAACCTTGACTCTTTGAGGAAGGGATTGAGCGGGAGCATATTAG
GTAGGACCCGAAAGATGGTGAACTACGCTTGAGTAGGGTGAAGCCAGGGGAAACTCTGGTGGAAGCTCGAAGCGATACTGACGTGCAAA
TCGTTCGTCAAACTTGAGTGTAGGGGCGAAAG

**>Metaradiophrya_lumbrici_KR_10_LT**

CGCTGAACTTAAGCATATCAGTAAGCGGAGGAAAAGAAACTAACAAGGATTGCTCCAGTAGCGGCGAGTGAACAGGCAAAAGCTCAAAT
TGAAAATCCGTGGCACA-AGCTATGGAATTGTAATCTATAGAGGTCTTCGGAAAGTACGAGTTTCGCACAAGTTCCTTGGAACTGGACG
TCATAGAGGGTGAAAACCCCGTACATGGCGAGACTTCGACTGTACGAAGGCTTTCGAAGAGTCGGGTTGTTTGGTATTGCAGCCCTAAA
TGGGAGATAAACTTCTTCTAAAGCTAAATATCTATGGGAAACCGATAGCGAACAAGTACTGCGAAGGAAAGATGAAAAGAACTTTGAAA
AGAGGGTTAAAAGACTTGAAACCGTTGAAAAGGAAGCGGTAGAAGAGTAATGATATCAGTTGCGATATACCTGCTATAGTGCGGACCCT
TGGGTCGTTTCGGACTTCATCGGACGGGCGATTTAATGCGTGACTGTGCTTTGTAGGTTTTTCGCAAATTGGTAGGCAAAATGAGGTTG
TGCAGGCGGAGTCAGTGGACTGGAGGACCCTTCATCGGAGTCAGTTCACAAAACGTCGGCGCAGCCTGAGGGGCCTACGGGCGATTTTG
TCAAAATGCTTCTACTGACCCGTCTTGAAACACGGACCAAGGAGTCTACCCAGTATGCGAGTGTTAGGGTGGAAAAACCCGAACGCGCA
ACGAAAGTGATTAGATGCCAAGCCGCAA--GGCCGCAGCACCAGCCAACCTTGACTCTTTGAGGAAGGGATTGAGCGGGAGCATATTAG
GTAGGACCCGAAAGATGGTGAACTACGCTTGAGTAGGGTGAAGCCAGGGGAAACTCTGGTGGAAGCTCGAAGCGATACTGACGTGCAAA
TCGTTCGTCAAACTTGAGTGTAGGGGCGAAAG

**>Metaradiophrya_lumbrici_JA2_25_LT**

CGCTGAACTTAAGCATATCAGTAAGCGGAGGAAAAGAAACTAACAAGGATTGCTCCAGTAGCGGCGAGTGAACAGGCAAAAGCTCAAAT
TGAAAATCCGTGGCACA-AGCTATGGAATTGTAATCTATAGAGGTCTTCGGAAAGTACGAGTTTCGCACAAGTTCCTTGGAACTGGACG
TCATAGAGGGTGAAAACCCCGTACATGGCGAGACTTCGACTGTACGAAGGCTTTCGAAGAGTCGGGTTGTTTGGTATTGCAGCCCTAAA
TGGGAGATAAACTTCTTCTAAAGCTAAATATCTATGGGAAACCGATAGCGAACAAGTACTGCGAAGGAAAGATGAAAAGAACTTTGAAA
AGAGGGTTAAAAGACTTGAAACCGTTGAAAAGGAAGCGGTAGAAGAGTAATGATATCAGTTGCGATATACCTGCTATAGTGCGGACCCT
TGGGTCGTTTCGGACTTCATCGGACGGGCGATTTAATGCGTGACTGTGCTTTGTAGGTTTTTCGCAAATTGGTAGGCAAAATGAGGTTG
TGCAGGCGGAGTCAGTGGACTGGAGGACCCTTCATCGGAGTCAGTTCACAAAACGTCGGCGCAGCCTGAGGGGCCTACGGGCGATTTTG
TCAAAATGCTTCTACTGACCCGTCTTGAAACACGGACCAAGGAGTCTACCCAGTATGCGAGTGTTAGGGTGGAAAAACCCGAACGCGCA
ACGAAAGTGATTAGATGCCAAGCCGCAA--GGCCGCAGCACCAGCCAACCTTGACTCTTTGAGGAAGGGATTGAGCGGGAGCATATTAG
GTAGGACCCGAAAGATGGTGAACTACGCTTGAGTAGGGTGAAGCCAGGGGAAACTCTGGTGGAAGCTCGAAGCGATACTGACGTGCAAA
TCGTTCGTCAAACTTGAGTGTAGGGGCGAAAG

**>Metaradiophrya_lumbrici_JA2_26_LT**

CGCTGAACTTAAGCATATCAGTAAGCGGAGGAAAAGAAACTAACAAGGATTGCTCCAGTAGCGGCGAGTGAACAGGCAAAAGCTCAAAT
TGAAAATCCGTGGCACA-AGCTATGGAATTGTAATCTATAGAGGTCTTCGGAAAGTACGAGTTTCGCACAAGTTCCTTGGAACTGGACG
TCATAGAGGGTGAAAACCCCGTACATGGCGAGACTTCGACTGTACGAAGGCTTTCGAAGAGTCGGGTTGTTTGGTATTGCAGCCCTAAA
TGGGAGATAAACTTCTTCTAAAGCTAAATATCTATGGGAAACCGATAGCGAACAAGTACTGCGAAGGAAAGATGAAAAGAACTTTGAAA
AGAGGGTTAAAAGACTTGAAACCGTTGAAAAGGAAGCGGTAGAAGAGTAATGATATCAGTTGCGATATACCTGCTATAGTGCGGACCCT
TGGGTCGTTTCGGACTTCATCGGACGGGCGATTTAATGCGTGACTGTGCTTTGTAGGTTTTTCGCAAATTGGTAGGCAAAATGAGGTTG
TGCAGGCGGAGTCAGTGGACTGGAGGACCCTTCATCGGAGTCAGTTCACAAAACGTCGGCGCAGCCTGAGGGGCCTACGGGCGATTTTG
TCAAAATGCTTCTACTGACCCGTCTTGAAACACGGACCAAGGAGTCTACCCAGTATGCGAGTGTTAGGGTGGAAAAACCCGAACGCGCA
ACGAAAGTGATTAGATGCCAAGCCGCAA--GGCCGCAGCACCAGCCAACCTTGACTCTTTGAGGAAGGGATTGAGCGGGAGCATATTAG
GTAGGACCCGAAAGATGGTGAACTACGCTTGAGTAGGGTGAAGCCAGGGGAAACTCTGGTGGAAGCTCGAAGCGATACTGACGTGCAAA
TCGTTCGTCAAACTTGAGTGTAGGGGCGAAAG

**>Metaradiophrya_speculorum_HkD_59_AT**

CGCTGAACTTAAGCATATCAGTAAGCGGAGGAAAAGAAACTAACAAGGATTGCTCCAGTAGCGGCGAGTGAACAGGCAAAAGCTCAAAT
TGAAAATCCGTGGCATATGGCTATGGAATTGTAATCTATAGAGGTCTTCGGAAGGTACGAGTTTCGCACAAGTTCCTTGGAACTGGACG
TCATAGAGGGTGAAAACCCCGTACATGGCGAGACTT-GACTGTACGAAGGCTTTCGAAGAGTCGGGTTGTTTGGTATTGCAGCCCTAAA
TGGGAGATAAACTTCTTCTAAAGCTAAATATCTATGGGAAACCGATAGCGAACAAGTACTGCGAAGGAAAGATGAAAAGAACTTTGAAA

AGAGGGTTAAAAGACTTGAAACCGTTGAAAAGGAAGCGGTAGAAGAGTAATG--ATCAGTTGCGATATACATGTTATAGTGCGGGCCCT
TGCGTCGTTTCGGACTTCATCGGACGGGCGATTCAATGAGTGACTGTGCTTTGCAGGTTTTTCGCAAATTGATAGGCAAAATGAGGGTG
TGCTGGCGGAGTCAGTGGACTGGAGGACCCTTCATCGGAGTCAGTTCACAAAACGGCGGCGCAGCCTGAGGGGCCTACGGGCGATTTTG
TCAAAATGCTTCTACTGACCCGTCTTGAAACACGGACCAAGGAGTCTACCCAGTATGCGAGTGTTAGGGTGGAAAAACCCGAACGCGCA
ACGAAAGTGATTAGATGCCAAGCCGCAA--GGCCGCAGCACCAGCCAACCTTGACTCTTTGAGGAAGGGATTGAGCGGGAGCATATTAG
GTAGGACCCGAAAGATGGTGAACTACGCTTGAGTAGGGTGAAGCCAGGGGAAACTCTGGTGGAAGCTCGAAGCGATACTGACGTGCAAA
TCGTTCGTCAAACTTGAGTGTAGGGGCGAAAG
**>Metaradiophrya_speculorum_HkD_60_AT**
CGCTGAACTTAAGCATATCAGTAAGCGGAGGAAAAGAAACTAACAAGGATTGCTCCAGTAGCGGCGAGTGAACAGGCAAAAGCTCAAAT
TGAAAATCCGTGGCATATGGCTATGGAATTGTAATCTATAGAGGTCTTCGGAAGGTACGAGTTTCGCACAAGTTCCTTGGAACTGGACG
TCATAGAGGGTGAAAACCCCGTACATGGCGAGACTT-GACTGTACGAAGGCTTTCGAAGAGTCGGGTTGTTTGGTATTGCAGCCCTAAA
TGGGAGATAAACTTCTTCTAAAGCTAAATATCTATGGGAAACCGATAGCGAACAAGTACTGCGAAGGAAAGATGAAAAGAACTTTGAAA
AGAGGGTTAAAAGACTTGAAACCGTTGAAAAGGAAGCGGTAGAAGAGTAATG--ATCAGTTGCGATATACATGTTATAGTGCGGGCCCT
TGCGTCGTTTCGGACTTCATCGGACGGGCGATTCAATGAGTGACTGTGCTTTGCAGGTTTTTCGCAAATTGATAGGCAAAATGAGGGTG
TGCTGGCGGAGTCAGTGGACTGGAGGACCCTTCATCGGAGTCAGTTCACAAAACGGCGGCGCACCCTGAGGGGCCTACGGGCGATTTTG
TCAAAATGCTTCTACTGACCCGTCTTGAAACACGGACCAAGGAGTCTACCCAGTATGCGAGTGTTAGGGTGGAAAAACCCGAACGCGCA
ACGAAAGTGATTAGATGCCAAGCCGCAA--GGCCGCAGCACCAGCCAACCTTGACTCTTTGAGGAAGGGATTGAGCGGGAGCATATTAG
GTAGGACCCGAAAGATGGTGAACTACGCTTGAGTAGGGTGAAGCCAGGGGAAACTCTGGTGGAAGCTCGAAGCGATACTGACGTGCAAA
TCGTTCGTCAAACTTGAGTGTAGGGGCGAAAG
**>Metaradiophrya_varians_BZ_12_EF**
CGCTGAACTTAAGCATATCAGTAAGCGGAGGAAAAGAAACTAACAAGGATTGCTCCAGTAGCGGCGAGTGAACAGGCAAAAGCTCAAAT
TGAAAATCCGTGGCACA-GGCTATGGAATTGTAATCTATAGAGGTCTTCGGAAGGCACGAGTTTCGCACAAGTTCCTTGGAACTGGACG
TCATAGAGGGTGAAAACCCCGTACATGGCGAGACTTCGGCTGTACGAAGGCTTTCGAAGAGTCGGGTTGTTTGGTATTGCAGCCCTAAA
TGGGAGATAAACTTCTTCTAAAGCTAAATATCTATGGGAAACCGATAGCGAACAAGTACTGCGAAGGAAAGATGAAAAGAACTTTGAAA
AGAGGGTTAAAAGACTTGAAACCGTTGAAAAGGAAGCGGTAGAAGAGTAATGATATCAGTTGTGTTATACCTGTTATAGAGCAGGCCCT
TGAATCGTTTCGGACTTCATCGGACGGGCGGTTCAATGTGTGACTGAACTTTACAGGTTTTTCGCAAATTGATAGGCAAAATGAGGTTG
TGCTGGCGGAGTCAGTGAATTGAAGGACCCTTCATCGGAGTCAGTTCACAAAACGTCGGTGCAGCCTGAGGGGCCTACGGGCGATTTTG
TCAAAATGCTTCTACTGACCCGTCTTGAAACACGGACCAAGGAGTCTACCCAGTATGCGAGTGTTAGGGTGGAAAAACCCGAACGCGCA
ACGAAAGTGATTAGATGCCAAGCCGAAA--GGCAGCAGCACCAGCCAACCTTGACTCTTTGAGGAAGGGATTGAGCGGGAGCATATTAG
GTAGGACCCGAAAGATGGTGAACTACGCTTGAGTAGGGTGAAGCCAGGGGAAACTCTGGTGGAAGCTCGAAGCGATACTGACGTGCAAA
TCGTTCGTCAAACTTGAGTGTAGGGGCGAAAG
**>Metaradiophrya_varians_BZ_14_EF**
CGCTGAACTTAAGCATATCAGTAAGCGGAGGAAAAGAAACTAACAAGGATTGCTCCAGTAGCGGCGAGTGAACAGGCAAAAGCTCAAAT
TGAAAATCCGTGGCACA-GGCTATGGAATTGTAATCTATAGAGGTCTTCGGAAGGCACGAGTTTCGCACAAGTTCCTTGGAACTGGACG
TCATAGAGGGTGAAAACCCCGTACATGGCGAGACTTCGGCTGTACGAAGGCTTTCGAAGAGTCGGGTTGTTTGGTATTGCAGCCCTAAA
TGGGAGATAAACTTCTTCTAAAGCTAAATATCTATGGGAAACCGATAGCGAACAAGTACTGCGAAGGAAAGATGAAAAGAACTTTGAAA
AGAGGGTTAAAAGACTTGAAACCGTTGAAAAGGAAGCGGTAGAAGAGTAATGATATCAGTTGTGTTATACCTGTTATAGAGCAGGCCCT
TGAATCGTTTCGGACTTCATCGGACGGGCGGTTCAATGTGTGACTGAACTTTACAGGTTTTTCGCAAATTGATAGGCAAAATGAGGTTG
TGCTGGCGGAGTCAGTGAATTGAAGGACCCTTCATCGGAGTCAGTTCACAAAACGTCGGTGCAGCCTGAGGGGCCTACGGGCGATTTTG
TCAAAATGCTTCTACTGACCCGTCTTGAAACACGGACCAAGGAGTCTACCCAGTATGCGAGTGTTAGGGTGGAAAAACCCGAACGCGCA
ACGAAAGTGATTAGATGCCAAGCCGAAA--GGCAGCAGCACCAGCCAACCTTGACTCTTTGAGGAAGGGATTGAGCGGGAGCATATTAG
GTAGGACCCGAAAGATGGTGAACTACGCTTGAGTAGGGTGAAGCCAGGGGAAACTCTGGTGGAAGCTCGAAGCGATACTGACGTGCAAA
TCGTTCGTCAAACTTGAGTGTAGGGGCGAAAG
**>Metaradiophrya_varians_JA1_19_EF**
CGCTGAACTTAAGCATATCAGTAAGCGGAGGAAAAGAAACTAACAAGGATTGCTCCAGTAGCGGCGAGTGAACAGGCAAAAGCTCAAAT
TGAAAATCCGTGGCACA-GGCTATGGAATTGTAATCTATAGAGGTCTTCGGAAGGCACGAGTTTCGCACAAGTTCCTTGGAACTGGACG
TCATAGAGGGTGAAAACCCCGTACATGGCGAGACTTCGGCTGTACGAAGGCTTTCGAAGAGTCGGGTTGTTTGGTATTGCAGCCCTAAA
TGGGAGATAAACTTCTTCTAAAGCTAAATATCTATGGGAAACCGATAGCGAACAAGTACTGCGAAGGAAAGATGAAAAGAACTTTGAAA
AGAGGGTTAAAAGACTTGAAACCGTTGAAAAGGAAGCGGTAGAAGAGTAATGATATCAGTTGTGTTATACCTGTTATAGAGCAGGCCCT
TGAATCGTTTCGGACTTCATCGGACGGGCGGTTCAATGTGTGACTGAACTTTACAGGTTTTTCGCAAATTGATAGGCAAAATGAGGTTG
TGCTGGCGGAGTCAGTGAATTGAAGGACCCTTCATCGGAGTCAGTTCACAAAACGTCGGTGCAGCCTGAGGGGCCTACGGGCGATTTTG
TCAAAATGCTTCTACTGACCCGTCTTGAAACACGGACCAAGGAGTCTACCCAGTATGCGAGTGTTAGGGTGGAAAAACCCGAACGCGCA
ACGAAAGTGATTAGATGCCAAGCCGAAA--GGCAGCAGCACCAGCCAACCTTGACTCTTTGAGGAAGGGATTGAGCGGGAGCATATTAG
GTAGGACCCGAAAGATGGTGAACTACGCTTGAGTAGGGTGAAGCCAGGGGAAACTCTGGTGGAAGCTCGAAGCGATACTGACGTGCAAA
TCGTTCGTCAAACTTGAGTGTAGGGGCGAAAG
**>Metaradiophrya_varians_JA1_22_EF**
CGCTGAACTTAAGCATATCAGTAAGCGGAGGAAAAGAAACTAACAAGGATTGCTCCAGTAGCGGCGAGTGAACAGGCAAAAGCTCAAAT
TGAAAATCCGTGGCACA-GGCTATGGAATTGTAATCTATAGAGGTCTTCGGAAGGCACGAGTTTCGCACAAGTTCCTTGGAACTGGACG
TCATAGAGGGTGAAAACCCCGTACATGGCGAGACTTCGGCTGTACGAAGGCTTTCGAAGAGTCGGGTTGTTTGGTATTGCAGCCCTAAA
TGGGAGATAAACTTCTTCTAAAGCTAAATATCTATGGGAAACCGATAGCGAACAAGTACTGCGAAGGAAAGATGAAAAGAACTTTGAAA
AGAGGGTTAAAAGACTTGAAACCGTTGAAAAGGAAGCGGTAGAAGAGTAATGATATCAGTTGTGTTATACCTGTTATAGAGCAGGCCCT
TGAATCGTTTCGGACTTCATCGGACGGGCGGTTCAATGTGTGACTGAACTTTACAGGTTTTTCGCAAATTGATAGGCAAAATGAGGTTG
TGCTGGCGGAGTCAGTGAATTGAAGGACCCTTCATCGGAGTCAGTTCACAAAACGTCGGTGCAGCCTGAGGGGCCTACGGGCGATTTTG
TCAAAATGCTTCTACTGACCCGTCTTGAAACACGGACCAAGGAGTCTACCCAGTATGCGAGTGTTAGGGTGGAAAAACCCGAACGCGCA
ACGAAAGTGATTAGATGCCAAGCCGAAA--GGCAGCAGCACCAGCCAACCTTGACTCTTTGAGGAAGGGATTGAGCGGGAGCATATTAG
GTAGGACCCGAAAGATGGTGAACTACGCTTGAGTAGGGTGAAGCCAGGGGAAACTCTGGTGGAAGCTCGAAGCGATACTGACGTGCAAA
TCGTTCGTCAAACTTGAGTGTAGGGGCGAAAG
**>Metaradiophrya_varians_BZkv_31_EF**
CGCTGAACTTAAGCATATCAGTAAGCGGAGGAAAAGAAACTAACAAGGATTGCTCCAGTAGCGGCGAGTGAACAGGCAAAAGCTCAAAT
TGAAAATCCGTGGCACA-GGCTATGGAATTGTAATCTATAGAGGTCTTCGGAAGGCACGAGTTTCGCACAAGTTCCTTGGAACTGGACG
TCATAGAGGGTGAAAACCCCGTACATGGCGAGACTTCGGCTGTACGAAGGCTTTCGAAGAGTCGGGTTGTTTGGTATTGCAGCCCTAAA

TGGGAGATAAACTTCTTCTAAAGCTAAATATCTATGGGAAACCGATAGCGAACAAGTACTGCGAAGGAAAGATGAAAAGAACTTTGAAA
AGAGGGTTAAAAGACTTGAAACCGTTGAAAAGGAAGCGGTAGAAGAGTAATGATATCAGTTGTGTTATACCTGTTATAGAGCAGGCCCT
TGAATCGTTTCGGACTTCATCGGACGGGCGGTTCAATGTGTGACTGAACTTTACAGGTTTTTCGCAAATTGATAGGCAAAATGAGGTTG
TGCTGGCGGAGTCAGTGAATTGAAGGACCCTTCATCGGAGTCAGTTCACAAAACGTCGGTGCAGCCTGAGGGGCCTACGGGCGATTTTG
TCAAAATGCTTCTACTGACCCGTCTTGAAACACGGACCAAGGAGTCTACCCAGTATGCGAGTGTTAGGGTGGAAAAACCCGAACGCGCA
ACGAAAGTGATTAGATGCCAAGCCGAAA--GGCAGCAGCACCAGCCAACCTTGACTCTTTGAGGAAGGGATTGAGCGGGAGCATATTAG
GTAGGACCCGAAAGATGGTGAACTACGCTTGAGTAGGGTGAAGCCAGGGGAAACTCTGGTGGAAGCTCGAAGCGATACTGACGTGCAAA
TCGTTCGTCAAACTTGAGTGTAGGGGCGAAAG

**>Metaradiophrya_varians_BZkv_32_EF**

CGCTGAACTTAAGCATATCAGTAAGCGGAGGAAAAGAAACTAACAAGGATTGCTCCAGTAGCGGCGAGTGAACAGGCAAAAGCTCAAAT
TGAAAATCCGTGGCACA-GGCTATGGAATTGTAATCTATAGAGGTCTTCGGAAGGCACGAGTTTCGCACAAGTTCCTTGGAACTGGACG
TCATAGAGGGTGAAAACCCCGTACATGGCGAGACTTCGGCTGTACGAAGGCTTTCGAAGAGTCGGGTTGTTTGGTATTGCAGCCCTAAA
TGGGAGATAAACTTCTTCTAAAGCTAAATATCTATGGGAAACCGATAGCGAACAAGTACTGCGAAGGAAAGATGAAAAGAACTTTGAAA
AGAGGGTTAAAAGACTTGAAACCGTTGAAAAGGAAGCGGTAGAAGAGTAATGATATCAGTTGTGTTATACCTGTTATAGAGCAGGCCCT
TGAATCGTTTCGGACTTCATCGGACGGGCGGTTCAATGTGTGACTGAACTTTACAGGTTTTTCGCAAATTGATAGGCAAAATGAGGTTG
TGCTGGCGGAGTCAGTGAATTGAAGGACCCTTCATCGGAGTCAGTTCACAAAACGTCGGTGCAGCCTGAGGGGCCTACGGGCGATTTTG
TCAAAATGCTTCTACTGACCCGTCTTGAAACACGGACCAAGGAGTCTACCCAGTATGCGAGTGTTAGGGTGGAAAAACCCGAACGCGCA
ACGAAAGTGATTAGATGCCAAGCCGAAA--GGCAGCAGCACCAGCCAACCTTGACTCTTTGAGGAAGGGATTGAGCGGGAGCATATTAG
GTAGGACCCGAAAGATGGTGAACTACGCTTGAGTAGGGTGAAGCCAGGGGAAACTCTGGTGGAAGCTCGAAGCGATACTGACGTGCAAA
TCGTTCGTCAAACTTGAGTGTAGGGGCGAAAG

**>Subanoplophrya_nodulata_PU_29_OT**

CGCTGAACTTAAGCATATCAGTAAGCGGAGGAAAAGAAACTAACAAGGATTGCCTCAGTAGCGGCGAGTGAACAGGCAAAAGCTCAAAT
TGAAAATCCATAATACATAGTTATGGAATTGTAATCTATAGAGATGATCGGAAGGTATGTGCTCTGTACAAGTTCTCTGGAACTAGACA
TCATAGAGGGTGATAATCCCGTATATGACAGAGCA-CGACTGTACGAGAATTTTCAAAGAGTCGGGTTGTTTGGTATTGCAGCCCTAAA
TGGGAGATAAACTTCTTCTAAAGCTAAATATTTATGGGAAACCGATAGCGAACAAGTACTGCGAAGGAAAGATGAAAAGAACTTTGAAA
ATAGGGTCAAAAGACTTGAAACCGTTGAAAAGGAAGCGGTAGGAGAGTAATAATATCAGTCGCTA-AAAGAAGTTCTAGCGTTAGCTTC
TGGAGTATTTAGGACCTTAAAAGCCTTGTGCTTCAGCAAGTAATAGCGCTTTGC-TGCCTTTTGCGAATTGATAGGCAAAATGAGGCTA
AGATGGCGGAGTCAGTGATTAGAAAGTTTTTCTA----------GTCACAAAACGCCATTTTAACCTGAGGGGCCTACGGGCGATTTTG
TCAAAAGGCTTCTACCGACCCGTCTTGAAACACGGACCAAGGAGTCTACCCAGTATGCAAGTGTTAGGGTGTAAAAACCCGAACGCACA
ACGAAAGTGA-GAGATGCCAAGCCGCAA--GGCCGCAGCACCAGCCAACCTTGAGTCTCTGACAAAGGGATTGAGCAAGAGCATATTAG
GTAGGACCCGAAAGATGGTGAACTACGCTTGAGTAGGGTGAAGCCAAAGGAAACTCTGGTGGAAGCTCGCAGCGATTCTGACGTGCAAA
TCGATCGTCAAACTTGAGTGTAGGGGCGAAAG

**>Subanoplophrya_nodulata_PU_30_OT**

CGCTGAACTTAAGCATATCAGTAAGCGGAGGAAAAGAAACTAACAAGGATTGCCTCAGTAGCGGCGAGTGAACAGGCAAAAGCTCAAAT
TGAAAATCCATAATACATAGTTATGGAATTGTAATCTATAGAGATGATCGGAAGGTATGTGCTCTGTACAAGTTCTCTGGAACTAGACA
TCATAGAGGGTGATAATCCCGTATATGACAGAGCA-CGACTGTACGAGAATTTTCAAAGAGTCGGGTTGTTTGGTATTGCAGCCCTAAA
TGGGAGATAAACTTCTTCTAAAGCTAAATATTTATGGGAAACCGATAGCGAACAAGTACTGCGAAGGAAAGATGAAAAGAACTTTGAAA
ATAGGGTCAAAAGACTTGAAACCGTTGAAAAGGAAGCGGTAGGAGAGTAATAATATCAGTCGCTA-AAAGAAGTTCTAGCGTTAGCTTC
TGGAGTATTTAGGACCTTAAAAGCCTTGTGCTTCAGCAAGTAATAGCGCTTTGC-TGCCTTTTGCGAATTGATAGGCAAAATGAGGCTA
AGATGGCGGAGTCAGGGATTAGAAAGTTTTTCTA----------GTCACAAAACGCCATTTTAACCTGAGGGGCCTACGGGCGATTTTG
TCAAAAGGCTTCTACCGACCCGTCTTGAAACACGGACCAAGGAGTCTACCCAGTATGCAAGTGTTAGGGTGTAAAAACCCGAACGCACA
ACGAAAGTGA-GAGATGCCAAGCCGCAA--GGCCGCAGCACCAGCCAACCTTGAGTCTCTGACAAAGGGATTGAGCAAGAGCATATTAG
GTAGGACCCGAAAGATGGTGAACTACGCTTGAGTAGGGTGAAGCCAAAGGAAACTCTGGTGGAAGCTCGCAGCGATTCTGACGTGCAAA
TCGATCGTCAAACTTGAGTGTAGGGGCGAAAG

# 16S rRNA gene alignment

**>Anoplophrya_allolobophorae_JA3_37_ACH**
AACGTTATTCGTAGTTACTGGGCGTATAGAATAAATAAGTAAAAATC-TAAACGA----G----TT----------------------
-TTGTTTATTTTTAAAAAATTAATAAATATTTTT--CATTTTTCTAATGTAATGATAAAATATAATAACATTATAGGGGTTTCTTGTGA
ATAAATAAATATTAATTTATATTGAATTATGAAGGTATAAAAATCAAATAGGATTCGGGACCCTAGTAGTTTATACAGTAAACAATGGT
--AATTA----------T-TAATATTAATTATCCGCTTAGGTAGTATAACCGCAAGGTTGAAATTTAACAGAATTGGCGGGAATTTAAT
TTAACGGTGGAGCATGTGGTTTAATACGATAATCCACGTAAAATCTTACCAGTGTGTT-TGTTT-ATTTAA-----TTTTTTTTTTATTA
GGAAAGTTATTATA-AACGTGTAGTATTGCATGGCTGTCGTCAGCTCGTGTTGTGAAATGTAAAATTAAGTTTTTTAACGAGTAAAATC
CCTAGTATTTTTATAATTATGAATTTTTATTTGTATTTAA-AAATACTCTTATATTTA---GGGTTGAAGTCAAGTCGGTATGATCTTT
GAATGCTGGGCTATACACGTGTTACAATGCTAATTACAAAAAATGGTTTTTTTGTGAAAATATACTATATTTTAAAAATTAGCAAAGTT
CAGATTATTTTTTGTAACTCAAAAGTATGAAGATGAAATCGTTAGTAATTGTAAATAATTATGTTACAGTGAATTATCAAATTAAATTT
TGTACACACTGCCC-ATCACGCTCGAAAAA-------CAAATTTAAT----------TTTTTTTTATTAGATAAACA----------CA
AAAGCTAACTGACGTTTTTGAACGTT----T--TTGTTTATTTAGTATTAATGATATTTT-----TTGTAATTTGAGTG
**>Anoplophrya_aporrectodeae_PUz_17_AT**
AACGTTATTCGTAATTACTGGGTGTATAGTATAAATAAACAAAAATA-TATGCGA----A----AT----------------------
-ACGCTTGATTTTTAAAAACTAATAAGTATTTTT--GATTTTTATAATGTAATGATAAAATATAATAACATTATGGGGATTTCTAAAAA
ATAAAT-GCTATTAGTTTATGTTGAATTATGAAGGTATAAAAATCAAATAGGATTCGGGACCCTAGTAGTTTATACAGTAAACAATGGC
--GGCCG----------A-TAACATCAGCTGCTCACTTAGGTAGTATAATCGCAAGATTGAAATTTAACAGAATTGGCGGGAATTTAAT
TTAACGGTGGAGCATGTGGTTTAATACGATAATCCACGTAAAACCTTACCAGTGTGGT-TATTT-ATTTAA-----TTTCTTTTTAAGA
ATAAAATTATTATA-AATGCGTAGTATTGCATGGCTGTCGTCAGCTCGTGTTGTGAAATGTAAAATTAAGTTTTTTAACGAGTAAAATC
CCTAATATTTATATAGTTATAAATTTTTATTTATACTTAA-AAATATTTTTATGTTTA---GGGTTGAAGTCAAGTCGGTATGATCTTT
GAATGCTGGGCTATACACGTGTTACAATGCTAATTACAAAAAATGGTTTTTTTGTAAAAATATACTATATTTAAAAAATTAGCAAAGTT
CAGATTATTTTTTGTAACTCAAAAATATGAAGATGAAATCGTTAGTAATTGTAAATAATTATGTTACAGTGAATTATCAAATTAAATTT
TGTACACACTGCCC-ATCACGCTCGGAAAA-------CAAA-TTGAT----------TTTTTTTTATTAAAAAAAACA----------TA
AAGAT--ATGGCGTATTTTTAATATT----T--TTGTTTATTTAGTATTGAATTTATATT-----TTGTAATTTGAGTG
**>Anoplophrya_aporrectodeae_PUz_40_AT**
AACGTTATTCGTAATTACTGGGTGTATAGTATAAATAAACAAAAATA-TATGCGA----A----AT----------------------
-ACGCTTGATTTTTAAAAACTAATAAGTATTTTT--GATTTTTATAATGTAATGATAAAATATAATAACATTATGGGGATTTCTAAAAA
ATAAAT-GCTATTAGTTTATGTTGAATTATGAAGGTATAAAAATCAAATAGGATTCGGGACCCTAGTAGTTTATACAGTAAACAATGGC
--GGCCG----------A-TAACATCAGCTGCTCACTTAGGTAGTATAATCGCAAGATTGAAATTTAACAGAATTGGCGGGAATTTAAT
TTAACGGTGGAGCATGTGGTTTAATACGATAATCCACGTAAAACCTTACCAGTGTGGT-TATTT-ATTTAA-----TTTCTTTTTAAGA
ATAAAATTATTATA-AATGCGTAGTATTGCATGGCTGTCGTCAGCTCGTGTTGTGAAATGTAAAATTAAGTTTTTTAACGAGTAAAATC
CCTAATATTTATATAGTTATAAATTTTTATTTATACTTAA-AAATATTTTTATGTTTA---GGGTTGAAGTCAAGTCGGTATGATCTTT
GAATGCTGGGCTATACACGTGTTACAATGCTAATTACAAAAAATGGTTTTTTTGTAAAAATATACTATATTTAAAAAATTAGCAAAGTT
CAGATTATTTTTTGTAACTCAAAAATATGAAGATGAAATCGTTAGTAATTGTAAATAATTATGTTACAGTGAATTATCAAATTAAATTT
TGTACACACTGCCC-ATCACGCTCGGAAAA-------CAAA-TTGAT----------TTTTTTTTATTAAAAAAAACA----------TA
AAGAT--ATGGCGTATTTTTAATATT----T--TTGTTTATTTAGTATTGAATTTATATT-----TTGTAATTTGAGTG
**>Anoplophrya_aporrectodeae_PUz_41_AT**
AACGTTATTCGTAATTACTGGGTGTATAGTATAAATAAACAAAAATA-TATGCGA----A----AT----------------------
-ACGCTTGATTTTTAAAAACTAATAAGTATTTTT--GATTTTTATAATGTAATGATAAAATATAATAACATTATGGGGATTTCTAAAAA
ATAAAT-GCTATTAGTTTATGTTGAATTATGAAGGTATAAAAATCAAATAGGATTCGGGACCCTAGTAGTTTATACAGTAAACAATGGC
--GGCCG----------A-TAACATCAGCTGCTCACTTAGGTAGTATAATCGCAAGATTGAAATTTAACAGAATTGGCGGGAATTTAAT
TTAACGGTGGAGCATGTGGTTTAATACGATAATCCACGTAAAACCTTACCAGTGTGGT-TATTT-ATTTAA-----TTTCTTTTTAAGA
ATAAAATTATTATA-AATGCGTAGTATTGCATGGCTGTCGTCAGCTCGTGTTGTGAAATGTAAAATTAAGTTTTTTAACGAGTAAAATC
CCTAATATTTATATAGTTATAAATTTTTATTTATACTTAA-AAATATTTTTATGTTTA---GGGTTGAAGTCAAGTCGGTATGATCTTT
GAATGCTGGGCTATACACGTGTTACAATGCTAATTACAAAAAATGGTTTTTTTGTAAAAATATACTATATTTAAAAAATTAGCAAAGTT
CAGATTATTTTTTGTAACTCAAAAATATGAAGATGAAATCGTTAGTAATTGTAAATAATTATGTTACAGTGAATTATCAAATTAAATTT
TGTACACACTGCCC-ATCACGCTCGGAAAA-------CAAA-TTGAT----------TTTTTTTTATTAAAAAAAACA----------TA
AAGAT--ATGGCGTATTTTTAATATT----T--TTGTTTATTTAGTATTGAATTTATATT-----TTGTAATTTGAGTG
**>Anoplophrya_lumbrici_RZ_6_LT**
AACGTTATTCGTAATAACTGAGTGTACTGGATAGGAATATATAAGTATTGGTTTATTTTA----AA----------------------
-ATACTAAACTGTTGTAAAATATAAATTATTTGT--AATTTGTAGAATGTAATGATAAAATATATTAACATTCTTTGGATTTCTATTAA
TCAAATATTTTTTATTTTTTGTAGATTTATGAAGGTATAATTATCAAATAGGATTCGGGACCCTAGTAGTTTATACAGTGAACAATGGC
--CGCTA----------T-TAATATAAACGGCTCACTTAGGTAGTACGATCGCAAGATTGAAATTTAACAGAATTGGCGGGAATTTAAT
TTAACGGTGGAACATGTGGTTTAATACGATAATCCACGTAAAAACTTACCAGTGTGCT-TTTTT-TATTTA-----AATTTTTTATTCT
TAGGCTTTAATATA-ACTATGTAGTATTGCATGGCTGTCGTCAGCTCGTGTTGTGAAATGTAAAATTAAGTTTTTTAACGAGTGAAATC
CCTGGTTTTTTTATATTTATAAACTATTGTTTATAATTAA-AAACA-CTATACGTTTA---GGGGTGAAGTCAAGTCTGTATGATCTTT
GAATGCTGGGCTACACACGTGTTACAATGGTAATTACAAAAAATGGTTTTTTTGTAAAAATTTACTATATTTTAAAGATTATCAATGTC
CGAATTATTTCTTGTAATTTAGAAATATTAAGATGAAATCGTTAGTAATTGTAAATTAGTATGTTACAGTGAATATTCAAATTAAATTT
TGTACACACTGCCC-ATCACGCTCGAAAAA-------TGAAATGCAT----------TTTATTTTAATAAAAACACAAGTAAAATTAAA
ATGTA--TTTGTTAGTGGTTAGCATGACTATTATTGTTTTTTTAGTAAAATAAAATTATT-----TTGTAATTTGAGTG
**>Anoplophrya_lumbrici_KR_9_LT**
AACGTTATTCGTAATAACTGAGTGTACTGGATAGGAATATATAAGTATTGGTTTATTTTA----AA----------------------
-ATACTAAACTGTTGTAAAATATAAATTATTTGT--AATTTGTAGAATGTAATGATAAAATATATTAACATTCTTTGGATTTCTATTAA
TCAAATATTTTTTATTTTTTGTAGATTTATGAAGGTATAATTATCAAATAGGATTCGGGACCCTAGTAGTTTATACAGTGAACAATGGC
--CGCTA----------T-TAATATAAACGGCTCACTTAGGTAGTACGATCGCAAGATTGAAATTTAACAGAATTGGCGGGAATTTAAT
TTAACGGTGGAACATGTGGTTTAATACGATAATCCACGTAAAAACTTACCAGTGTGCT-TTTTT-TATTTA-----AATTTTTTATTTT
TAGGCTTTAATATA-ACTATGTAGTATTGCATGGCTGTCGTCAGCTCGTGTTGTGAAATGTAAAATTAAGTTTTTTAACGAGTGAAATC
CCTGGTTTTTTTATATTTATAAACTATTGTTTATAATTAA-AAACA-CTATACGTTTA---GGGGTGAAGTCAAGTCTGTATGATCTTT
GAATGCTGGGCTACACACGTGTTACAATGGTAATTACAAAAAATGGTTTTTTTGTAAAAATTTACTATATTTTAAAGATTATCAATGTC

CGAATTATTTCTTGTAATTTAGAAATATTAAGATGAAATCGTTAGTAATTGTAAATTAGTATGTTACAGTGAATATTCAAATTAAATTT
TGTACACACTGCCC-ATCACGCTCGAAAAA-------TGAAATGCAT----------TTTATTTTAATAAAAACACAAGTAAAATTAAA
ATGTA--TTTGTTAGTGGTTAGCATGACTATTATTGTTTTTTTAGTAAAATAAAATTATT-----TTGTAATTTGAGTG

**>Anoplophrya_lumbrici_KR_11_LT**

AACGTTATTCGTAATAACTGAGTGTACTGGATAGGAATATATAAGTATTGGTTTATTTTA----AA-----------------------
-ATACTAAACTGTTGTAAAATATAAATTATTTGT--AATTTGTAGAATGTAATGATAAAATATATTAACATTCTTTGGATTTCTATTAA
TCAAATATTTTTTATTTTTTGTAGATTTATGAAGGTATAATTATCAAATAGGATTCGGGACCCTAGTAGTTTATACAGTGAACAATGGC
--CGCTA----------T-TAATATAAACGGCTCACTTAGGTAGTACGATCGCAAGATTGAAATTTAACAGAATTGGCGGGAATTTAAT
TTAACGGTGGAACATGTGGTTTAATACGATAATCCACGTAAAAACTTACCAGTGTGCT-TTTTT-TATTTA-----AATTTTTTATTTT
TAGGCTTTAATATA-ACTATGTAGTATTGCATGGCTGTCGTCAGCTCGTGTTGTGAAATGTAAAATTAAGTTTTTTAACGAGTGAAATC
CCTGGTTTTTTTATATTTATAAACTATTGTTTATAATTAA-AAACA-CTATACGTTTA---GGGGTGAAGTCAAGTCTGTATGATCTTT
GAATGCTGGGCTACACACGTGTTACAATGGTAATTACAAAAAATGGTTTTTTTGTAAAAATTTACTATATTTTAAAGATTATCAATGTC
CGAATTATTTCTTGTAATTTAGAAATATTAAGATGAAATCGTTAGTAATTGTAAATTAGTATGTTACAGTGAATATTCAAATTAAATTT
TGTACACACTGCCC-ATCACGCTCGAAAAA-------TGAAATGCAT----------TTTATTTTAATAAAAACACAAGTAAAATTAAA
ATGTA--TTTGTTAGTGGTTAGCATGACTATTATTGTTTTTTTAGTAAAATAAAATTATT-----TTGTAATTTGAGTG

**>Anoplophrya_octolasionis_MU_56_OL**

AACGTTATTCGCAATAACTGGGTGTACAGCATAAAAAAATATAAATA-CAAAAAATTATA----TA-----------------------
-GTTGTAGATTGTAAAAAAGTATTAAATATTTTT--AATTTTTATAAGGTAATGATAAAATATTTTAACATTATAATAATTTCTTAAAA
AAAAATATTTACTACTTTTTGTTTAATTATGAAGGTATAAATATCGAATAGGATTCGGGACCCTAGTAGTTTATACAGTAAAAAATGGT
--TATAA----------ATTAATATAAGTAACCCACTTAGGTAGTACGATCGCAAGATTGAAATTTAACAGAATTGGCGGGAATTTAAT
TTAACGGTGGAGCATGTGGTTTAATACGATAATCCACGTAAAATCTTACCAGTGTGTTGTAGTT-ATTTAA-----AGTTGATTTATTT
TTTGCTTTATTATA-ACTGTGTAGTATTGCATGGCTGTCGTCAGCTCGTGTTGTGAGATGTAAAATTAAGTTTTTTAACGAGTGGAATC
CCTGGTGTTTTTGTAATAAGGAA-TGTTATTTTTTATTAA-GAATATCTTTATGTTTA---GGGTTGAAGTCAAGTCGATATGATCTTT
GAATGCTGGGCTATACACGTGTTACATTGGTAATTACAAAAAATGGTTTTTTTGTAAAAATTTACTATATTTAAAAAATTATCAAGGTT
CGGATTATTTTTTGTAATTTAAAAGTATGAAGATGAAATCGTTAGTAATTGCAAATAAGTATGTTGTAGTGAATAATAAAATTAAATTC
TGTACACACTGCCC-ATCACGCTCGGAAAA-------TTAA-ATTAT----------TTTTTTTTATGAAAAAACCA----------TT
ATATT--TTTATATTTGAATAATTTTGTTCT--TTGGCTTTTTCGTTAAAAAAATATATT-----TTATGATTTGAGTG

**>Anoplophrya_octolasionis_MU_57_OL**

AACGTTATTCGCAATAACTGGGTGTACAGCATAAAAAAATATAAATA-CAAAAAATTATA----TA-----------------------
-GTTGTAGATTGTAAAAAAGTATTAAATATTTTT--AATTTTTATAAGGTAATGATAAAATATTTTAACATTATAATAATTTCTTAAAA
AAAAATATTTACTACTTTTTGTTTAATTATGAAGGTATAAATATCGAATAGGATTCGGGACCCTAGTAGTTTATACAGTAAAAAATGGT
--TATAA----------ATTAATATAAGTAACCCACTTAGGTAGTACGATCGCAAGATTGAAATTTAACAGAATTGGCGGGAATTTAAT
TTAACGGTGGAGCATGTGGTTTAATACGATAATCCACGTAAAATCTTACCAGTGTGTTGTAGTT-ATTTAA-----AGTTGATTTATTT
TTTGCTTTATTATA-ACTGTGTAGTATTGCATGGCTGTCGTCAGCTCGTGTTGTGAGATGTAAAATTAAGTTTTTTAACGAGTGGAATC
CCTGGTGTTTTTGTAATAAGGAA-TGTTATTTTTTATTAA-GAATATCTTTATGTTTA---GGGTTGAAGTCAAGTCGATATGATCTTT
GAATGCTGGGCTATACACGTGTTACATTGGTAATTACAAAAAATGGTTTTTTTGTAAAAATTTACTATATTTAAAAAATTATCAAGGTT
CGGATTATTTTTTGTAATTTAAAAGTATGAAGATGAAATCGTTAGTAATTGCAAATAAGTATGTTGTAGTGAATAATAAAATTAAATTC
TGTACACACTGCCC-ATCACGCTCGGAAAA-------TTAA-ATTAT----------TTTTTTTTATGAAAAAACCA----------TT
ATATT--TTTATATTTGAATAATTTTGTTCT--TTGGCTTTTTCGTTAAAAAAATATATT-----TTATGATTTGAGTG

**>Anoplophrya_octolasionis_MU_58_OL**

AACGTTATTCGCAATAACTGGGTGTACAGCATAAAAAAATATAAATA-CAAAAAATTATA----TA-----------------------
-GTTGTAGATTGTAAAAAAGTATTAAATATTTTT--AATTTTTATAAGGTAATGATAAAATATTTTAACATTATAATAATTTCTTAAAA
AAAAATATTTACTACTTTTTGTTTAATTATGAAGGTATAAATATCGAATAGGATTCGGGACCCTAGTAGTTTATACAGTAAAAAATGGT
--TATAA----------ATTAATATAAGTAACCCACTTAGGTAGTACGATCGCAAGATTGAAATTTAACAGAATTGGCGGGAATTTAAT
TTAACGGTGGAGCATGTGGTTTAATACGATAATCCACGTAAAATCTTACCAGTGTGTTGTAGTT-ATTTAA-----AGTTGATTTATTT
TTTGCTTTATTATA-ACTGTGTAGTATTGCATGGCTGTCGTCAGCTCGTGTTGTGAGATGTAAAATTAAGTTTTTTAACGAGTGGAATC
CCTGGTGTTTTTGTAATAAGGAA-TGTTATTTTTTATTAA-GAATATCTTTATGTTTA---GGGTTGAAGTCAAGTCGATATGATCTTT
GAATGCTGGGCTATACACGTGTTACATTGGTAATTACAAAAAATGGTTTTTTTGTAAAAATTTACTATATTTAAAAAATTATCAAGGTT
CGGATTATTTTTTGTAATTTAAAAGTATGAAGATGAAATCGTTAGTAATTGCAAATAAGTATGTTGTAGTGAATAATAAAATTAAATTC
TGTACACACTGCCC-ATCACGCTCGGAAAA-------TTAA-ATTAT----------TTTTTTTTATGAAAAAACCA----------TT
ATATT--TTTATATTTGAATAATTTTGTTCT--TTGGCTTTTTCGTTAAAAAAATATATT-----TTATGATTTGAGTG

**>Anoplophrya_vulgaris_BZ_13_EF**

AACGTTATTCGTATTTACTGGGTGTATAGTATAAATAAGTAAAGAAT--------TTATA----AA-----------------------
-TATATAAATTGTTAAAAAGTGCAAAAAATTCTTTAATTCTTTGTAGTGTAATGATAAAATATAGTAACACTTTAAGAATTTCTTTTAA
AAAAGTTTTTTTTACTTTTTTTTAATTTATGAAGGTATAAATATCGAATAGGATTCGGGACCCTAGTAGTTTATACAGTAAACAATGGC
--TTTTT----------T-TAATGTAAATAGCTCACTTAGGGAGTACGGTCGCAAGATTGAAATTTAACAGAATTGGCGGGAATTTAAT
TTAACGGTGGAACATGTGGTTTAATACGATAATCCACGTAAAATCTTACCAGTGTGTT-TAATT-TATTTA-----ATGTTAGTTATTT
TTAACCTTATTATAAATTATGTAGTATTGCATGGCTGTCGTCAGCTCGTGTTGTGAAATGTAAAATTAAGTTTTTTAACGAGTGCAATC
CCTAATATCTTTATACTGCTAAACTATGGTTTGTCTTTAC-AGGTA--TTTTTGTTCA---GGGTCGAAGTCAAGTCGGTATGGTCTTT
GAATGCTGGGCTACACACGTGTTACATTGAAAAATACAAAAAATGGTTTATTTGCGAGAATTTACTATATTTAAAAATTTTTCTAAGTT
CGAATTGTTTCTTGGAATTCAGAAGCATGAAGATGAAATCGTTAGTAATTGTAAATAAATATGTTACAGTGAATAATTAAATTAAATTT
TGTACACACTGCCC-ATCAGGCTCGAAAAG-------TATAGTGAAT----------TTTTTTATAAAAAACTTATG------------
AATTA--CTTGTAATGTATTGGATTA--TGTTAAGTGGTTTATA----AAAAAATAAATT-----TTGTAATTTGAGTG

**>Anoplophrya_vulgaris_JA1_18_EF**

AACGTTATTCGTATTTACTGGGTGTATAGTATAAATAAGTAAAGAAT--------TTATA----AA-----------------------
-TATATAAATTGTTAAAAAGTGCAAAAAATTCTTTAATTCTTTGTAGTGTAATGATAAAATATAGTAACACTTTAAGAATTTCTTTTAA
AAAAGTTTTTTTTACTTTTTTTTAATTTATGAAGGTATAAATATCGAATAGGATTCGGGACCCTAGTAGTTTATACAGTAAACAATGGC
--TTTTT----------T-TAATGTAAATAGCTCACTTAGGGAGTACGGTCGCAAGATTGAAATTTAACAGAATTGGCGGGAATTTAAT
TTAACGGTGGAACATGTGGTTTAATACGATAATCCACGTAAAATCTTACCAGTGTGTT-TAATT-TATTTA-----ATGTTAGTTATTT
TTAACCTTATTATAAATTATGTAGTATTGCATGGCTGTCGTCAGCTCGTGTTGTGAAATGTAAAATTAAGTTTTTTAACGAGTGCAATC
CCTAATATCTTTATACTGCTAAACTATGGTTTGTCTTTAC-AGGTA--TTTTTGTTCA---GGGTCGAAGTCAAGTCGGTATGGTCTTT

GAATGCTGGGCTACACACGTGTTACATTGAAAAATACAAAAAATGGTTTATTTGCGAGAATTTACTATATTTAAAAATTTTTCTAAGTT
CGAATTGTTTCTTGGAATTCAGAAGCATGAAGATGAAATCGTTAGTAATTGTAAATAAATATGTTACAGTGAATAATTAAATTAAATTT
TGTACACACTGCCC-ATCAGGCTCGAAAAG-------TATAGTGAAT----------TTTTTTATAAAAAACTTATG------------
AATTA--CTTGTAATGTATTGGATTA--TGTTAAGTGGTTTATA----AAAAAATAAATT-----TTGTAATTTGAGTG

**>Anoplophrya_vulgaris_JA1_20_EF**

AACGTTATTCGTATTTACTGGGTGTATAGTATAAATAAGTAAAGAAT--------TTATA----AA-----------------------
-TATATAAATTGTTAAAAAGTGCAAAAAATTCTTTAATTCTTTGTAGTGTAATGATAAAATATAGTAACACTTTAAGAATTTCTTTTAA
AAAAGTTTTTTTTACTTTTTTTTAATTTATGAAGGTATAAATATCGAATAGGATTCGGGACCCTAGTAGTTTATACAGTAAACAATGGC
--TTTTT----------T-TAATGTAAATAGCTCACTTAGGGAGTACGGTCGCAAGATTGAAATTTAACAGAATTGGCGGGAATTTAAT
TTAACGGTGGAACATGTGGTTTAATACGATAATCCACGTAAAATCTTACCAGTGTGTT-TAATT-TATTTA-----ATGTTAGTTATTT
TTAACCTTATTATAAATTATGTAGTATTGCATGGCTGTCGTCAGCTCGTGTTGTGAAATGTAAAATTAAGTTTTTTAACGAGTGCAATC
CCTAATATCTTTATACTGCTAAACTATGGTTTGTCTTTAC-AGGTA--TTTTTGTTCA---GGGTCGAAGTCAAGTCGGTATGGTCTTT
GAATGCTGGGCTACACACGTGTTACATTGAAAAATACAAAAAATGGTTTATTTGCGAGAATTTACTATATTTAAAAATTTTTCTAAGTT
CGAATTGTTTCTTGGAATTCAGAAGCATGAAGATGAAATCGTTAGTAATTGTAAATAAATATGTTACAGTGAATAATTAAATTAAATTT
TGTACACACTGCCC-ATCAGGCTCGAAAAG-------TATAGTGAAT----------TTTTTTATAAAAAACTTATG------------
AATTA--CTTGTAATGTATTGGATTA--TGTTAAGTGGTTTATA----AAAAAATAAATT-----TTGTAATTTGAGTG

**>Anoplophrya_vulgaris_JA1_21_EF**

AACGTTATTCGTATTTACTGGGTGTATAGTATAAATAAGTAAAGAAT--------TTATA----AA-----------------------
-TATATAAATTGTTAAAAAGTGCAAAAAATTCTTTAATTCTTTGTAGTGTAATGATAAAATATAGTAACACTTTAAGAATTTCTTTTAA
AAAAGTTTTTTTTACTTTTTTTTAATTTATGAAGGTATAAATATCGAATAGGATTCGGGACCCTAGTAGTTTATACAGTAAACAATGGC
--TTTTT----------T-TAATGTAAATAGCTCACTTAGGGAGTACGGTCGCAAGATTGAAATTTAACAGAATTGGCGGGAATTTAAT
TTAACGGTGGAACATGTGGTTTAATACGATAATCCACGTAAAATCTTACCAGTGTGTT-TAATT-TATTTA-----ATGTTAGTTATTT
TTAACCTTATTATAAATTATGTAGTATTGCATGGCTGTCGTCAGCTCGTGTTGTGAAATGTAAAATTAAGTTTTTTAACGAGTGCAATC
CCTAATATCTTTATACTGCTAAACTATGGTTTGTCTTTAC-AGGTA--TTTTTGTTCA---GGGTCGAAGTCAAGTCGGTATGGTCTTT
GAATGCTGGGCTACACACGTGTTACATTGAAAAATACAAAAAATGGTTTATTTGCGAGAATTTACTATATTTAAAAATTTTTCTAAGTT
CGAATTGTTTCTTGGAATTCAGAAGCATGAAGATGAAATCGTTAGTAATTGTAAATAAATATGTTACAGTGAATAATTAAATTAAATTT
TGTACACACTGCCC-ATCACGCTCGAAAAG-------TATAGTGAAT----------TTTTTTATAAAAAACTTATG------------
AATTA--CTTGTAATGTATTGGATTA--TGTTAAGTGGTTTATA----AAAAAATAAATT-----TTGTAATTTGAGTG

**>Anoplophrya_vulgaris_NG_27_DV**

AACGTTATTCGGATTTACTGGGTGTATAGTATAAATAAATAAAGAAT--------TTAAA----AA-----------------------
-TATATAAATTGTTAAAAAGTATAAAAAATTCTTTAATTCTTTGTAGTGTAATGATAAAATATAGCAACACTTTAAGAATTTCTTTTAA
AAAAGTTTTTTTTACTTTTTTTTCATTTATGAAGGTATAAGTATCAAATAGGATTCGGGACCCTAGTAGTTTATACAGTAAACAATGGT
TATTTTT----------T-TAATATAGATAACTCACTTAGGGAGTACGATCGCAAGATTGAAATTTAACAGAATTGGCGGGAATTTAAT
TTAACGGTGGAACATGTGGTTTAATACGATAATCCACGTAAAATCTTACCAGTGTGTT-TAATT-TATTTA-----ATATTAGGTATTT
CTAATTTTATTATAGATTGCGTAGTATTGCATGGCTGTCGTCAGCTCGTGTTGTGAAATGTAAAATTAAGTTTTTTAACGAGTGCAATC
CCTAATATCTTTATACTACTAAACAATTGTTTATTTTTAT-AGATA-TTTTTTGTTTA---GGGTTGAAGTCAAGTCGGTATGGTCTTT
GAATGCTGGGCTACACACGTGTTACATTGAAAAATACAAAAATTGGTTTCTTTGTGAAAATTTACTATATTTAAAAATTTTTCTAAGTT
CGAATTGTTTCTTGGAATTCAGAAGCATGAAGATGAAATCGTTAGTAATTGTAAATAAATATGTTACAGTGAATAATTAAATTAAATTT
TGTACACACTGCCC-ATCACGCTCGAAAAA-------TATAACGAAT----------TTTTTTATAAAAAATTTATG------------
GTTTA--ACTGTAGTTAATGGACCTA--CATTAAAGTTTTTATA----AAAAAATAAATT-----TTGTAATTTGAGTG

**>Anoplophrya_vulgaris_NG_28_DV**

AACGTTATTCGGATTTACTGGGTGTATAGTATAAATAAATAAAGAAT--------TTAAA----AA-----------------------
-TATATAAATTGTTAAAAAGTATAAAAAATTCTTTAATTCTTTGTAGTGTAATGATAAAATATAGCAACACTTTAAGAATTTCTTTTAA
AAAAGTTTTTTTTACTTTTTTTTCATTTATGAAGGTATAAGTATCAAATAGGATTCGGGACCCTAGTAGTTTATACAGTAAACAATGGT
TATTTTT----------T-TAATATAGATAACTCACTTAGGGAGTACGATCGCAAGATTGAAATTTAACAGAATTGGCGGGAATTTAAT
TTAACGGTGGAACATGTGGTTTAATACGATAATCCACGTAAAATCTTACCAGTGTGTT-TAATT-TATTTA-----ATATTAGGTATTT
CTAATTTTATTATAGATTGCGTAGTATTGCATGGCTGTCGTCAGCTCGTGTTGTGAAATGTAAAATTAAGTTTTTTAACGAGTGCAATC
CCTAATATCTTTATACTACTAAACAATTGTTTATTTTTAT-AGATA-TTTTTTGTTTA---GGGTTGAAGTCAAGTCGGTATGGTCTTT
GAATGCTGGGCTACACACGTGTTACATTGAAAAATACAAAAATTGGTTTCTTTGTGAAAATTTACTATATTTAAAAATTTTTCTAAGTT
CGAATTGTTTCTTGGAATTCAGAAGCATGAAGATGAAATCGTTAGTAATTGTAAATAAATATGTTACAGTGAATAATTAAATTAAATTT
TGTACACACTGCCC-ATCACGCTCGAAAAA-------TATAACGAAT----------TTTTTTATAAAAAATTTATG------------
GTTTA--ACTGTAGTTAATGGACCTA--CATTAAAGTTTTTATA----AAAAAATAAATT-----TTGTAATTTGAGTG

**>Metaradiophrya_chlorotica_JA2_1M_ACH**

AGCGTTGTTCGTAATTACTGAGTGTAACGCATAAAAAAGCATATATA-TATAACTATG------GA----------------------
-GTTATATATGATTTTAATATTTTTTTTTT-TTA--ATATTTTATAATGTAATGATAAAATATAATAACAATATAAGATTTTTT--TTA
TAAAAAAAAAAAATATTTAGGCATTATTATGAAGGTATAAGTATCAAATAGGATTAGGTACCCTAGTAGTTTTTACAGTAAAATAAGGT
--TATTTAAAT------T-TAACATAAATAACTTGCTTAGGTAGTACGATCGCAAGATTGAAATTTAACAGAATTGGCGGGAATTTAAT
TTAACGGTGGAGTATGTGGTTTAATTCGATAATCCACGAAAAATCTTACCAGTATGTT-TTTTAATTTTTA-----AATTTATTTATTG
TTTATTTAATAAATTAATTGGTAGTATTGCATGGCTGTCGTCAACTCGTGTTGTGAAATGTAAAATTAAGTTTTTTAACGAGTGAAATC
TCTAGTATTTTATTTTTTTA----T-TTAAATATAAAAAATACAAATATTGTTTGTTA---GAGCTGAAGTCAAGTCAGTATGATCTTT
GTATGCTGGGCTATACACGTGCTACAATGATAAAAACAAAGAATGTTTATAATGTAAATTAAAATTATTTTTTAAAAATTATCTAAGTA
CGGATTATTTTTTGTAATTTAAAAATATGAAGTTGAAATCGTTAGTAATTATAAATGAGTATGTTATAGTGAAAAATAAAATTAAATTT
TGTACACACTGCCCCATCACGCCTCGGAAAAATTTTTTATTTTTTT----------TTTAAAAATAAAATT--TTATTTAAAA---TA
ATTTT--AATATTTTTTTTTAAAATA------TATAAAATTTTTATTTTTTTTATATTATAAATAGGGTAATTTGAGTG

**>Metaradiophrya_chlorotica_JA2_2M_ACH**

AGCGTTGTTCGTAATTACTGAGTGTAACGCATAAAAAAGCATATATA-TATAACTATG------GA----------------------
-GTTATATATGATTTTAATATTTTTTTTTT-TTA--ATATTTTATAATGTAATGATAAAATATAATAACAATATAAGATTTTTT--TTA
TAAAAAAAAAAAATATTTAGGCATTATTATGAAGGTATAAGTATCAAATAGGATTAGGTACCCTAGTAGTTTTTACAGTAAAATAAGGT
--TATTTAAAT------T-TAACATAAATAACTTGCTTAGGTAGTACGATCGCAAGATTGAAATTTAACAGAATTGGCGGGAATTTAAT
TTAACGGTGGAGTATGTGGTTTAATTCGATAATCCACGAAAAATCTTACCAGTATGTT-TTTTAATTTTTA-----AATTTATTTATTG
TTTATTTAATAAATTAATTGGTAGTATTGCATGGCTGTCGTCAACTCGTGTTGTGAAATGTAAAATTAAGTTTTTTAACGAGTGAAATC

TCTAGTATTTTATTTTTTTA----T-TTAAATATAAAAAATACAAATATTGTTTGTTA---GAGCTGAAGTCAAGTCAGTATGATCTTT
GTATGCTGGGCTATACACGTGCTACAATGATAAAAACAAAGAATGTTTATAATGTAAATTAAAATTATTTTTTAAAAATTATCTAAGTA
CGGATTATTTTTTGTAATTTAAAAATATGAAGTTGAAATCGTTAGTAATTATAAATGAGTATGTTATAGTGAAAAATAAAATTAAATTT
TGTACACACTGCCCCATCACGCCTCGGAAAAATTTTTTTATTTTTTTT----------TTTAAAAATAAAATT--TTATTTAAAA---TA
ATTTT--AATATTTTTTTTTAAAATA------TATAAAATTTTATTTTTTTTATATTATAAATAGGGTAATTTGAGTG
**>Metaradiophrya_chlorotica_JA2_3M_ACH**
AGCGTTGTTCGTAATTACTGAGTGTAACGCATAAAAAAGCATATATA-TATAACTATG------GA------------------------
-GTTATATATGATTTTAATATTTTTTTTTTT-TTA--ATATTTTATAATGTAATGATAAAATATAATAACAATATAAGATTTTTT--TTA
TAAAAAAAAAAAATATTTAGGCATTATTATGAAGGTATAAGTATCAAATAGGATTAGGTACCCTAGTAGTTTTTACAGTAAAATAAGGT
--TATTTAAAT------T-TAACATAAATAACTTGCTTAGGTAGTACGATCGCAAGATTGAAATTTAACAGAATTGGCGGGAATTTAAT
TTAACGGTGGAGTATGTGGTTTAATTCGATAATCCACGAAAAATCTTACCAGTATGTT-TTTTAATTTTTA-----AATTTATTTATTG
TTTATTTAATAAATTAATTGGTAGTATTGCATGGCTGTCGTCAACTCGTGTTGTGAAATGTAAAATTAAGTTTTTTAACGAGTGAAATC
TCTAGTATTTTATTTTTTTA----T-TTAAATATAAAAAATACAAATATTGTTTGTTA---GAGCTGAAGTCAAGTCAGTATGATCTTT
GTATGCTGGGCTATACACGTGCTACAATGATAAAAACAAAGAATGTTTATAATGTAAATTAAAATTATTTTTTAAAAATTATCTAAGTA
CGGATTATTTTTTGTAATTTAAAAATATGAAGTTGAAATCGTTAGTAATTATAAATGAGTATGTTATAGTGAAAAATAAAATTAAATTT
TGTACACACTGCCCCATCACGCCTCGGAAAAATTTTTTTATTTTTTTT----------TTTAAAAATAAAATT--TTATTTAAAA---TA
ATTTT--AATATTTTTTTTTAAAATA------TATAAAATTTTATTTTTTTTATATTATAAATAGGGTAATTTGAGTG
**>Metaradiophrya_lumbrici_RZ_4_LT**
AGCGTTGTTCGTAATAACTGAGTGTATAGAATAAATAAACAAAAGTA-TTTAAATATTGATA--AA------------------------
-TATTTAATTGATATTAATATTATTTTTTTT-TTT--AAATGTTATATTGTAATGATAAAATATAATCACAATATAAAATTTTTTAAT-A
AATTATAAAATAATATTTAAGTATAATTATGAAGGTATAAGTAGCAAATAGGATTAGATACCCTAGTAGTTTTTACAGTAAACGATGGC
--CATATGTGTATATAAA-TAATATAAGTGGTTTACTTAGGTAGTATGATCGCAAGATTGAAATTTAACAGAATTGGCGGGAATTTAAT
TTAACGGTGGAGTATGTGGTTTAATTCGATAATCCACGAAAAATCTTACCAGTGTGGT-TTTTA-TATTTT-----TTTTTTTTTAAAA
AAAATAAATTAATATAATATGTAGTATTGCAGGGCTGTCGTCAACTCGGGTTGTGAAATGTAAAATTAAGTTTTTTAACGAGGGTAATC
TCTAAAA-----ATTTTATG----TTTTTATTAAAATAAATAACTATATTATTAATAAATAGAGCTGAAGTCAAGTCAGTATGATCTTA
GTATGCTGGGCTATACATGTGCTACAACGAAAAAAACAAAAAATGTTTTTAATGTGAATTGAAATTATTTTTTAAAAATTTTCTAAGTT
CGGATTATTTTTTGTAATTTAAAAATATGAAGTTGAAATCGTTAGTAATTGTAAATGAGTATGTTACAGTGAAAAATAAAATTAAATTT
TGTACACACTGCCC-ATCACGCTCGGAAAA-------TTAAATACTT----------ATTATTCAAAAAAATCTTTATTTAA-------
ATTTT--TATTTTTTTTTTAAAATAA------TAGGGGTTTT---TTTTTATATAAAATT-----TGGGGATTTGAGTG
**>Metaradiophrya_lumbrici_RZ_5_LT**
AGCGTTGTTCGTAATAACTGAGTGTATAGAATAAATAAACAAAAGTA-TTTAAATATTGATA--AA------------------------
-TATTTAATTGATATTAATATTATTTTTTTT-TTT--AAATGTTATATTGTAATGATAAAATATAATCACAATATAAAATTTTTTAAT-A
AATTATAAAATAATATTTAAGTATAATTATGAAGGTATAAGTAGCAAATAGGATTAGATACCCTAGTAGTTTTTACAGTAAACGATGGC
--CATATGTGTATATAAA-TAATATAAGTGGTTTACTTAGGTAGTATGATCGCAAGATTGAAATTTAACAGAATTGGCGGGAATTTAAT
TTAACGGTGGAGTATGTGGTTTAATTCGATAATCCACGAAAAATCTTACCAGTGTGGT-TTTTA-TATTTT-----TTTTTTTTTAAAA
AAAATAAATTAATATAATATGTAGTATTGCAGGGCTGTCGTCAACTCGGGTTGTGAAATGTAAAATTAAGTTTTTTAACGAGGGTAATC
TCTAAAA-----ATTTTATG----TTTTTATTAAAATAAATAACTATATTATTAATAAATAGAGCTGAAGTCAAGTCAGTATGATCTTA
GTATGCTGGGCTATACATGTGCTACAACGAAAAAAACAAAAAATGTTTTTAATGTGAATTGAAATTATTTTTTAAAAATTTTCTAAGTT
CGGATTATTTTTTGTAATTTAAAAATATGAAGTTGAAATCGTTAGTAATTGTAAATGAGTATGTTACAGTGAAAAATAAAATTAAATTT
TGTACACACTGCCC-ATCACGCTCGGAAAA-------TTAAATACTT----------ATTATTCAAAAAAATCTTTATTTAA-------
ATTTT--TATTTTTTTTTTAAAATAA------TAGGGGTTTT---TTTTTATATAAAATT-----TGGGGATTTGAGTG
**>Metaradiophrya_lumbrici_KR_8_LT**
AGCGTTGTTCGTAATAACTGAGTGTATAGAATAAATAAACAAGAGTA-TTTAAATATTGATA--AA------------------------
-TATTTAATTGATATTAATATTATTTTTTTT-TTT--AAATGTTATATTGTAATGATAAAATATAATCACAATATAAAATTTTTTAAT-A
AATTATAAAATAATATTTAAGTATAATTATGAAGGTATAAGTAGCAAATAGGATTAGATACCCTAGTAGTTTTTACAGTAAACAATGAC
--CATATGTGTATATAAA-TAATATAAGTGGTTTACTTAGGTAGTATGATCGCAAGATTGAAATTTAACAGAATTGGCGGGAATTTAAT
TTAACGGTGGAGTATGTGGTTTAATTCGATAATCCACGAAAAATCTTACCAGTGTGGT-TTTTA-TATTTT-----TTTTTTTTTAAAA
AAAATAAATTAATATAATATGTAGTATTGCAGGGCTGTCGTCAACTCGGGTTGTGAAATGTAAAATTAAGTTTTTTAACGAGGGCAATC
TCTAAAA-----ATTTTATG----TTTTTATTAAAATAAATAACTACATTATTAATAAATAGAGCTGAAGTCAAGTCAGTATGATCTTA
GTATGCTGGGCTATACATGTGCTACAACGAAAAAAACAAAAAATGTTTTTAATGTGAATTGAAATTATTTTTTAAAAATTTTCTAAGTT
CGGATTATTTTTTGTAATTTAAAAATATGAAGTTGAAATCGTTAGTAATTGTAAATGAGTATGTTACAGTGAAAAATAAAATTAAATTT
TGTACACACTGCCC-ATCACGCTCGGAAAA-------TTAAATACTT----------ATTATTCAAAAAAATCTTTATTTAA-------
ATTTT--TATTTTTTTTTTAAAATAA------TAGGGGTTTT---TTTTTATATAAAATT-----TGGGGATTTGAGTG
**>Metaradiophrya_lumbrici_KR_10_LT**
AGCGTTGTTCGTAATAACTGAGTGTATAGAATAAATAAACAAGAGTA-TTTAAATATTGATA--AA------------------------
-TATTTAATTGATATTAATATTATTTTTTTT-TTT--AAATGTTATATTGTAATGATAAAATATAATCACAATATAAAATTTTTTAAT-A
AATTATAAAATAATATTTAAGTATAATTATGAAGGTATAAGTAGCAAATAGGATTAGATACCCTAGTAGTTTTTACAGTAAACAATGAC
--CATATGTGTATATAAA-TAATATAAGTGGTTTACTTAGGTAGTATGATCGCAAGATTGAAATTTAACAGAATTGGCGGGAATTTAAT
TTAACGGTGGAGTATGTGGTTTAATTCGATAATCCACGAAAAATCTTACCAGTGTGGT-TTTTA-TATTTT-----TTTTTTTTTAAAA
AAAATAAATTAATATAATATGTAGTATTGCAGGGCTGTCGTCAACTCGGGTTGTGAAATGTAAAATTAAGTTTTTTAACGAGGGCAATC
TCTAAAA-----ATTTTATG----TTTTTATTAAAATAAATAACTACATTATTAATAAATAGAGCTGAAGTCAAGTCAGTATGATCTTA
GTATGCTGGGCTATACATGTGCTACAACGAAAAAAACAAAAAATGTTTTTAATGTGAATTGAAATTATTTTTTAAAAATTTTCTAAGTT
CGGATTATTTTTTGTAATTTAAAAATATGAAGTTGAAATCGTTAGTAATTGTAAATGAGTATGTTACAGTGAAAAATAAAATTAAATTT
TGTACACACTGCCC-ATCACGCTCGGAAAA-------TTAAATACTT----------ATTATTCAAAAAAATCTTTATTTAA-------
ATTTT--TATTTTTTTTTTAAAATAA------TAGGGGTTTT---TTTTTATATAAAATT-----TGGGGATTTGAGTG
**>Metaradiophrya_lumbrici_JA2_25_LT**
AGCGTTGTTCGTAATAACTGAGTGTATAGAATAAATAAACAAAAGTA-TTTAAATATTGATA--AA------------------------
-TATTTAATTGATATTAATATTATTTTTTTT-TTT--AAATGTTATATTGTAATGATAAAATATAATCACAATATAAAATTTTTTAAT-A
AATTATAAAATAATATTTAAGTATAATTATGAAGGTATAAGTAGCAAATAGGATTAGATACCCTAGTAGTTTTTACAGTAAACGATGGC
--CATATGTGTATATAAA-TAATATAAGTGGTTTACTTAGGTAGTATGATCGCAAGATTGAAATTTAACAGAATTGGCGGGAATTTAAT
TTAACGGTGGAGTATGTGGTTTAATTCGATAATCCACGAAAAATCTTACCAGTGTGGT-TTTTA-TATTTT-----TTTTTTTTTAAAA

AAAATAAATTAATATAATATGTAGTATTGCAGGGCTGTCGTCAACTCGGGTTGTGAAATGTAAAATTAAGTTTTTTAACGAGGGTAATC
TCTAAAA-----ATTTTATG----TTTTTATTAAAATAAATAACTATATTATTAATAAATAGAGCTGAAGTCAAGTCAGTATGATCTTA
GTATGCTGGGCTATACATGTGCTACAACGAAAAAAACAAAAAATGTTTTAATGTGAATTGAAATTATTTTTTAAAAATTTTCTAAGTT
CGGATTATTTTTTGTAATTTAAAAATATGAAGTTGAAATCGTTAGTAATTGTAAATGAGTATGTTACAGTGAAAAATAAAATTAAATTT
TGTACACACTGCCC-ATCACGCTCGGAAAA-------TTAAATACTT----------ATTATTCAAAAAAATCTTTATTTAA-------
ATTTT--TATTTTTTTTTTAAAATAA------TAGGGGTTTT---TTTTTATATAAAATT-----TGGGGATTTGAGTG
**>Metaradiophrya_lumbrici_JA2_26_LT**
AGCGTTGTTCGTAATAACTGAGTGTATAGAATAAATAAACAAAAGTA-TTTAAATATTGATA--AA-----------------------
-TATTTAATTGATATTAATATTATTTTTTT-TTT--AAATGTTATATTGTAATGATAAAATATAATCACAATATAAAATTTTTTAAT-A
AATTATAAAATAATATTTAAGTATAATTATGAAGGTATAAGTAGCAAATAGGATTAGATACCCTAGTAGTTTTTACAGTAAACGATGGC
--CATATGTGTATATAAA-TAATATAAGTGGTTTACTTAGGTAGTATGATCGCAAGATTGAAATTTAACAGAATTGGCGGGAATTTAAT
TTAACGGTGGAGTATGTGGTTTAATTCGATAATCCACGAAAAATCTTACCAGTGTGGT-TTTTA-TATTTT-----TTTTTTTTTAAAA
AAAATAAATTAATATAATATGTAGTATTGCAGGGCTGTCGTCAACTCGGGTTGTGAAATGTAAAATTAAGTTTTTTAACGAGGGTAATC
TCTAAAA-----ATTTTATG----TTTTTATTAAAATAAATAACTATATTATTAATAAATAGAGCTGAAGTCAAGTCAGTATGATCTTA
GTATGCTGGGCTATACATGTGCTACAACGAAAAAAACAAAAAATGTTTTAATGTGAATTGAAATTATTTTTTAAAAATTTTCTAAGTT
CGGATTATTTTTTGTAATTTAAAAATATGAAGTTGAAATCGTTAGTAATTGTAAATGAGTATGTTACAGTGAAAAATAAAATTAAATTT
TGTACACACTGCCC-ATCACGCTCGGAAAA-------TTAAATACTT----------ATTATTCAAAAAAATCTTTATTTAA-------
ATTTT--TATTTTTTTTTTAAAATAA------TAGGGGTTTT---TTTTTATATAAAATT-----TGGGGATTTGAGTG
**>Metaradiophrya_speculorum_HkD_59_AT**
AGCGTTGTTCGTAATGACTGAGTATATTGTATAAGAAAATAATGATG-CGTGTAAACAAATTATTT-----------------------
-TATACGTTTGTTTATAATATTTTTTTTTTTAATT--AAATTCTGTATTGTAATGATAAAATATAATAACAGTGCAGGATTTTTTTATAG
AATTAAAAATTAATATTTTTATATTATTATGAAGGTATAATTATCGAATAGGATTAGGGACCCTAGTAGTTTTTACAGTAAACAATGGC
--TATTTAAAT------T-TAATGTTATTAGCTTACTTAGGTAGTACGATCGCAAGGTTGAAATTTAACAGAATTGGCGGGAATTTAAT
TTAACGGTGGAGTATGTGGTTTAATACGATAATCCACGTAAAATCTTACCAGTGTGTT-TTTTG-TGATTT-----AATTTCATTAATT
GGAGTATAATAGCGCAATGAGTAGTATTGCATGGCTGTCGTCAACTCGTGTTGTGAAATGTAAAATTAAGTTTTTTAACGAGTGCAATC
TTTGGTATTTATATTAATTG----TATAATGTATAATTAA-AAAAATATTAGTAATAAGT-GAGCTGAAGTCAAGTCGGTATGATCTTT
GTATGCTGGGCTACACACGTGCTACAATGAAAAAAACAAAGAACGGTTTTTATGTGAATATAAACTATCTTTAAAAAATTTTCAAAGTT
CGGATTATTTTTTGAAATTTAAAAGTATGAAGGTGAAATCGTTAGTAATTGTAAATGAGTATGTTACGGTGAAAAATAAAATTAAATTT
TGTACACACTGCCC-ATCACGCTCGGAAAA-------TTAATTGTAT----------TGTATAAATAAAAAT--TT------TG---TG
GTATA--TTTGTATTTTTTGAAAATA------GGCATTTTTTTTATTTTTCTTTTATAAT-----TGGTGATTTGAGTG
**>Metaradiophrya_speculorum_HkD_60_AT**
AGCGTTGTTCGTAATGACTGAGTATATTGTATAAGAAAATAATGATG-CGTGTAAACAAATTATTT-----------------------
-TATACGTTTGTTTATAATATTTTTTTTTTTAATT--AAATTCTGTATTGTAATGATAAAATATAATAACAGTGCAGGATTTTTTTATAG
AATTAAAAATTAATATTTTTATATTATTATGAAGGTATAATTATCGAATAGGATTAGGGACCCTAGTAGTTTTTACAGTAAACAATGGC
--TATTTAAAT------T-TAATGTTATTAGCTTACTTAGGTAGTACGATCGCAAGGTTGAAATTTAACAGAATTGGCGGGAATTTAAT
TTAACGGTGGAGTATGTGGTTTAATACGATAATCCACGTAAAATCTTACCAGTGTGTT-TTTTG-TGATTT-----AATTTCATTAATT
GGAGTATAATAGCGCAATGAGTAGTATTGCATGGCTGTCGTCAACTCGTGTTGTGAAATGTAAAATTAAGTTTTTTAACGAGTGCAATC
TTTGGTATTTATATTAATTG----TATAATGTATAATTAA-AAAAATATTAGTAATAAGT-GAGCTGAAGTCAAGTCGGTATGATCTTT
GTATGCTGGGCTACACACGTGCTACAATGAAAAAAACAAAGAACGGTTTTTATGTGAATATAAACTATCTTTAAAAAATTTTCAAAGTT
CGGATTATTTTTTGAAATTTAAAAGTATGAAGGTGAAATCGTTAGTAATTGTAAATGAGTATGTTACGGTGAAAAATAAAATTAAATTT
TGTACACACTGCCC-ATCACGCTCGGAAAA-------TTAATTGTAT----------TGTATAAATAAAAAT--TT------TG---TG
GTATA--TTTGTATTTTTTGAAAATA------GGCATTTTTTTTATTTTTCTTTTATAAT-----TGGTGATTTGAGTG
**>Metaradiophrya_varians_BZ_12_EF**
AGCGTTATTCTAATTAACTGAGTGTATAGTATAAAATAATAAATAAA-TATATGTATGTA----TA-----------------------
-TATATATATGATTTTAATATTATTAGTTT-TTT--AAATTTTATATTGTAATGATAAAATATTTTGACAATATATGGTTTTCTAATTT
AAAAAATTTTTAATATTTTTTTATTATTATGAAGGTAAAAATATCAAATAGGATTAGGGACCCTAGTAGTTTTTACAGTAAAAAATAAC
--TAAATATGA------T-TAATATAATTAGTTTACTTAGGTAGTACTTTCGCAAGATTGAAATTTAACAGAATTGGCGGGAATTTAAT
TTAACGGTGGAGTATGTGGTTTAATATGATAATCCACGTAAAAACTTACCAATGTGTT-TTATATTTATTT-----AATTTTTATAAAT
TAAAATTAATATAATATTTAGTAGTATTGCATGGCTGTCGTCAACTCGTGTTGTGAGATGTAGAATTAGGTTTCTTAACGAGTGCAATC
TTTTATATTTAATTTATCTA----TATTATATAGATTAAACAAAAATATTATTAATAA---AAGTTGAAGTCAAGTCGGTATGATCTTT
GTATGTTGGGCTACACACGTGCTACAATGGTAAAAACAAAAAATGGTTTTTATGTAAATATAAACTATCTTTAAAAAATTATCTAAGTT
CGGATTATTTTTTGTAATTTAAAAATATGAAGTTGAAATCGTTAGTAATTATAAATGAGTATGTTATAGTGAAAAATAAAATTAAATTT
TGTACACACTGCCC-ATCACGCTCGTAGAA-------TTAATTTTAT----------ATTTTGAATAAAGTT--T--------------
ATATA--TATATATTTTCTAAAATAA------ATATATTTTTATTTTTTTTTATTTTAAT-----TAGTAATTTGAGTG
**>Metaradiophrya_varians_BZ_14_EF**
AGCGTTATTCTAATTAACTGAGTGTATAGTATAAAATAATAAATAAA-TATATGTATGTA----TA-----------------------
-TATATATATGATTTTAATATTATTAGTTT-TTT--AAATTTTATATTGTAATGATAAAATATTTTGACAATATATGGTTTTCTAATTT
AAAAAATTTTTAATATTTTTTTATTATTATGAAGGTAAAAATATCAAATAGGATTAGGGACCCTAGTAGTTTTTACAGTAAAAAATAAC
--TAAATATGA------T-TAATATAATTAGTTTACTTAGGTAGTACTTTCGCAAGATTGAAATTTAACAGAATTGGCGGGAATTTAAT
TTAACGGTGGAGTATGTGGTTTAATATGATAATCCACGTAAAAACTTACCAATGTGTT-TTATATTTATTT-----AATTTTTATAAAT
TAAAATTAATATAATATTTAGTAGTATTGCATGGCTGTCGTCAACTCGTGTTGTGAGATGTAGAATTAGGTTTCTTAACGAGTGCAATC
TTTTATATTTAATTTATCTA----TATTATATAGATTAAACAAAAATATTATTAATAA---AAGTTGAAGTCAAGTCGGTATGATCTTT
GTATGTTGGGCTACACACGTGCTACAATGGTAAAAACAAAAAATGGTTTTTATGTAAATATAAACTATCTTTAAAAAATTATCTAAGTT
CGGATTATTTTTTGTAATTTAAAAATATGAAGTTGAAATCGTTAGTAATTATAAATGAGTATGTTATAGTGAAAAATAAAATTAAATTT
TGTACACACTGCCC-ATCACGCTCGTAGAA-------TTAATTTTAT----------ATTTTGAATAAAGTT--T--------------
ATATA--TATATATTTTCTAAAATAA------ATATATTTTTATTTTTTTTTATTTTAAT-----TAGTAATTTGAGTG
**>Metaradiophrya_varians_JA1_19_EF**
AGCGTTATTCTAATTAACTGAGTGTATAGTATAAAATAATAAATAAA-TATATGTATGTA----TA-----------------------
-TATATATATGATTTTAATATTATTAGTTT-TTT--AAATTTTATATTGTAATGATAAAATATTTTGACAATATATGGTTTTCTAATTT
AAAAAATTTTTAATATTTTTTTATTATTATGAAGGTAAAAATATCAAATAGGATTAGGGACCCTAGTAGTTTTTACAGTAAAAAATAAC
--TAAATATGA------T-TAATATAATTAGTTTACTTAGGTAGTACTTTCGCAAGATTGAAATTTAACAGAATTGGCGGGAATTTAAT

TTAACGGTGGAGTATGTGGTTTAATATGATAATCCACGTAAAAACTTACCAATGTGTT-TTATATTTATTT-----AATTTTTATAAAT
TAAAATTAATATAATATTTAGTAGTATTGCATGGCTGTCGTCAACTCGTGTTGTGAGATGTAGAATTAGGTTTCTTAACGAGTGCAATC
TTTTATATTTAATTTATCTA----TATTATATAGATTAAACAAAAATATTATTAATAA---AAGTTGAAGTCAAGTCGGTATGATCTTT
GTATGTTGGGCTACACACGTGCTACAATGGTAAAAACAAAAAATGGTTTTTATGTAAATATAAACTATCTTTAAAAAATTATCTAAGTT
CGGATTATTTTTTGTAATTTAAAAATATGAAGTTGAAATCGTTAGTAATTATAAATGAGTATGTTATAGTGAAAAATAAAATTAAATTT
TGTACACACTGCCC-ATCACGCTCGTAGAA-------TTAATTTTAT----------ATTTGAATAAAGTT--T---------------
ATATA--TATATATTTTCTAAAATAA------ATATATTTTTTATTTTTTTTTATTTTAAT-----TAGTAATTTGAGTG

**>Metaradiophrya_varians_JA1_22_EF**

AGCGTTATTCTAATTAACTGAGTGTATAGTATAAAATAATAAATAAA-TATATGTATGTA----TA-----------------------
-TATATATATGATTTTAATATTATTAGTTT-TTT--AAATTTTATATTGTAATGATAAAATATTTTGACAATATATGGTTTTCTAATTT
AAAAAATTTTTAATATTTTTTTATTATTATGAAGGTAAAAATATCAAATAGGATTAGGGACCCTAGTAGTTTTTACAGTAAAAAATAAC
--TAAATATGA------T-TAATATAATTAGTTTACTTAGGTAGTACTTTCGCAAGATTGAAATTTAACAGAATTGGCGGGAATTTAAT
TTAACGGTGGAGTATGTGGTTTAATATGATAATCCACGTAAAAACTTACCAATGTGTT-TTATATTTATTT-----AATTTTTATAAAT
TAAAATTAATATAATATTTAGTAGTATTGCATGGCTGTCGTCAACTCGTGTTGTGAGATGTAGAATTAGGTTTCTTAACGAGTGCAATC
TTTTATATTTAATTTATCTA----TATTATATAGATTAAACAAAAATATTATTAATAA---AAGTTGAAGTCAAGTCGGTATGATCTTT
GTATGTTGGGCTACACACGTGCTACAATGGTAAAAACAAAAAATGGTTTTTATGTAAATATAAACTATCTTTAAAAAATTATCTAAGTT
CGGATTATTTTTTGTAATTTAAAAATATGAAGTTGAAATCGTTAGTAATTATAAATGAGTATGTTATAGTGAAAAATAAAATTAAATTT
TGTACACACTGCCC-ATCACGCTCGTAGAA-------TTAATTTTAT----------ATTTGAATAAAGTT--T---------------
ATATA--TATATATTTTCTAAAATAA------ATATATTTTTTATTTTTTTTTATTTTAAT-----TAGTAATTTGAGTG

**>Metaradiophrya_varians_BZkv_31_EF**

AGCGTTATTCTAATTAACTGAGTGTATAGTATAAAATAATAAATAAA-TATATGTATGTA----TA-----------------------
-TATATATATGATTTTAATATTATTAGTTT-TTT--AAATTTTATATTGTAATGATAAAATATTTTGACAATATATGGTTTTCTAATTT
AAAAAATTTTTAATATTTTTTTATTATTATGAAGGTAAAAATATCAAATAGGATTAGGGACCCTAGTAGTTTTTACAGTAAAAAATAAC
--TAAATATGA------T-TAATATAATTAGTTTACTTAGGTAGTACTTTCGCAAGATTGAAATTTAACAGAATTGGCGGGAATTTAAT
TTAACGGTGGAGTATGTGGTTTAATATGATAATCCACGTAAAAACTTACCAATGTGTT-TTATATTTATTT-----AATTTTTATAAAT
TAAAATTAATATAATATTTAGTAGTATTGCATGGCTGTCGTCAACTCGTGTTGTGAGATGTAGAATTAGGTTTCTTAACGAGTGCAATC
TTTTATATTTAATTTATCTA----TATTATATAGATTAAACAAAAATATTATTAATAA---AAGTTGAAGTCAAGTCGGTATGATCTTT
GTATGTTGGGCTACACACGTGCTACAATGGTAAAAACAAAAAATGGTTTTTATGTAAATATAAACTATCTTTAAAAAATTATCTAAGTT
CGGATTATTTTTTGTAATTTAAAAATATGAAGTTGAAATCGTTAGTAATTATAAATGAGTATGTTATAGTGAAAAATAAAATTAAATTT
TGTACACACTGCCC-ATCACGCTCGTAGAA-------TTAATTTTAT----------ATTTGAATAAAGTT--T---------------
ATATA--TATATATTTTCTAAAATAA------ATATATTTTTTATTTTTTTTTATTTTAAT-----TAGTAATTTGAGTG

**>Metaradiophrya_varians_BZkv_32_EF**

AGCGTTATTCTAATTAACTGAGTGTATAGTATAAAATAATAAATAAA-TATATGTATGTA----TA-----------------------
-TATATATATGATTTTAATATTATTAGTTT-TTT--AAATTTTATATTGTAATGATAAAATATTTTGACAATATATGGTTTTCTAATTT
AAAAAATTTTTAATATTTTTTTATTATTATGAAGGTAAAAATATCAAATAGGATTAGGGACCCTAGTAGTTTTTACAGTAAAAAATAAC
--TAAATATGA------T-TAATATAATTAGTTTACTTAGGTAGTACTTTCGCAAGATTGAAATTTAACAGAATTGGCGGGAATTTAAT
TTAACGGTGGAGTATGTGGTTTAATATGATAATCCACGTAAAAACTTACCAATGTGTT-TTATATTTATTT-----AATTTTTATAAAT
TAAAATTAATATAATATTTAGTAGTATTGCATGGCTGTCGTCAACTCGTGTTGTGAGATGTAGAATTAGGTTTCTTAACGAGTGCAATC
TTTTATATTTAATTTATCTA----TATTATATAGATTAAACAAAAATATTATTAATAA---AAGTTGAAGTCAAGTCGGTATGATCTTT
GTATGTTGGGCTACACACGTGCTACAATGGTAAAAACAAAAAATGGTTTTTATGTAAATATAAACTATCTTTAAAAAATTATCTAAGTT
CGGATTATTTTTTGTAATTTAAAAATATGAAGTTGAAATCGTTAGTAATTATAAATGAGTATGTTATAGTGAAAAATAAAATTAAATTT
TGTACACACTGCCC-ATCACGCTCGTAGAA-------TTAATTTTAT----------ATTTGAATAAAGTT--T---------------
ATATA--TATATATTTTCTAAAATAA------ATATATTTTTTATTTTTTTTTATTTTAAT-----TAGTAATTTGAGTG

**>Subanoplophrya_nodulata_PU_29_OT**

AGTGTTATTTGTATTTATTAAGTAAAAAACATAAAAATGCACAAAACTCAATGTTATTTATAATAAGTACAATTTATTGTATAATAAAA
TAACATTGTTATTTAAAAATTTAAAATTATATTA--TTTTTTTATTATATAGTGATTAAATACAATAATATAATAATAAAATCT---AA
TAGTATAATTTAAAATTTATGTAGTCTTATGAAAGTATACTCATTGAATAGGATTAGAGACCCTAGTAATGTATACCGTAAACTAATAT
--ATTTT----------ATAAATATA-------TACTTAGGTAGTATTTTCGCAAGATTGAAATTTAACAGAATTGGCGGGAA-TTAAT
ACAGTGGTGGAATATGTAATTTAATACGATAATCCACGTAAAAACTTACCAGTGTGTAAAAATATTATTTAAAAATAAAGTTTTTATTT
AAAATAT---------TTTTTTAGTATTGCATGGCTGTCGTCAATCCATGTTGTAAGATATATAATTTATTTATATTAAATGGATGTAA
CTTTAATTTTTAAACAAGATA---ATTTTAATAAATTATTTATATATTAATTATGTTA---TAAATGAAGTCAAGTCTTTATGGTCTAT
GTACACTGGGCTACTTATGTATTACAATATTAAAAACAAAATAATAAAATTATGTAAATAATTTTTATTTTAAAAA------TTAATAA
CGAACTGTACATTGGAA--TATGTACATGAAGTTGGAATCACTAGTAATTGTAATTAATTATGTTACAATGAATG--TATATTTTATTT
TGCACACACTGCCC-ATCACACTTGAAAAA-------TTATTATTGTAGCGTATATATTTATATAATAAAATATTTAATC---------
-------TTATTATTTTTTAAATGTA-------------------ATACTCTATTATAAG-----TATTGATTTAAGTG

**>Subanoplophrya_nodulata_PU_30_OT**

AGTGTTATTTGTATTTATTAAGTAAAAAACATAAAAATGCACAAAACTCAATGTTATTTATAATAAGTACAATTTATTGTATAATAAAA
TAACATTGTTATTTAAAAATTTAAAATTATATTA--TTTTTTTATTATATAGTGATTAAATACAATAATATAATAATAAAATCT---AA
TAGTATAATTTAAAATTTATGTAGTCTTATGAAAGTATACTCATTGAATAGGATTAGAGACCCTAGTAATGTATACCGTAAACTAATAT
--ATTTT----------ATAAATATA-------TACTTAGGTAGTATTTTCGCAAGATTGAAATTTAACAGAATTGGCGGGAA-TTAAT
ACAGTGGTGGAATATGTAATTTAATACGATAATCCACGTAAAAACTTACCAGTGTGTAAAAATATTATTTAAAAATAAAGTTTTTATTT
AAAATAT---------TTTTTTAGTATTGCATGGCTGTCGTCAATCCATGTTGTAAGATATATAATTTATTTATATTAAATGGATGTAA
CTTTAATTTTTAAACAAGATA---ATTTTAATAAATTATTTATATATTAATTATGTTA---TAAATGAAGTCAAGTCTTTATGGTCTAT
GTACACTGGGCTACTTATGTATTACAATATTAAAAACAAAATAATAAAATTATGTAAATAATTTTTATTTTAAAAA------TTAATAA
CGAACTGTACATTGGAA--TATGTACATGAAGTTGGAATCACTAGTAATTGTAATTAATTATGTTACAATGAATG--TATATTTTATTT
TGCACACACTGCCC-ATCACACTTGAAAAA-------TTATTATTGTAGCGTATATATTTATATAATAAAATATTTAATC---------
-------TTATTATTTTTTAAATGTA-------------------ATACTCTATTATAAG-----TATTGATTTAAGTG

## Cytochrome *c* oxidase subunit I alignment

**>Anoplophrya_allolobophorae_JA3_37_ACH**
GGTTCCAAAGATGTTGCATACCCCAGGTTAAACAGTATAGCTTTCTGATTGCAACCCTGTGCATTTTTAGTCGTAGTTAAATTGGCGTT
TTTAAGACCGCAATATTGAAAATATTATGATGACACTGAAATTTATATAAACTTTTTAAAAAAAATAAAGGAATCTAAGTTGCTAGCCA
ACCTTAATTATGAATCGTCTTATGATGTTAATTTATTTAAAAAAACTAAAATAAATTTAGTGTTTTGAGATTCTTTTTTTTTA---TAT
TGTGAAAGCTCATACTTATTTTTTTCTAAATTATCTAACTATAGAAGAAAAAAATTATATTTTTCTAAATGCTCAAATAGAAACTCTGT
AGTAACTGGTTGGACGTTTATAACCCCGTTTACATCCAATACTAAATTTTCTGGTGTAGGTTCTCAGGATATTTTACTTATTGGTGTTG
TTTTTGTTGGTTTTACAACAACATTGGGTTTTGTAAATTTATTGATTACCCGTAGAACTTTATGTATGCCTGGATTGCGTAATCGCCGA
ATATTGTTGCCCTTTGTTACAATTACACTACTCTTGTCGTTACGAATGTTAGTAATAATTACCCCAGTTTTAAGTGCCGCGATGATAAT
GATGTTGTTAGACCGTCATTGGGCAACTTCATTTTTTGATTTTGCATATGGAGGTGATCCAATTTTATCTCAGCATTTATTTTGATTTT
TCGGTCACCCT
**>Anoplophrya_vulgaris_NG_27_DV**
GGTTCCAAAGATGTTGCATATCCACGATTGAATAGCATTGCGTTTTGATTGCAACCGTGTGCGTTTATTGTGGTAGTAAAGATGGCATT
TCTTAGACCACAGTATTGACAATATTATAATGATATAGATTTTTTTATTTTATTTTTAGAAAAAACAAAACAAATAAATTATTTTAATA
ATTTTTGGTTACAAAACAATATAAATAATAATGTTGAGAAAAAATTTAAAATAAATTTATTATTTTGAGATTCATTTTTTTAT---TAC
TGTGAGTCTTCATATTTATTTTTTTTTAAACTTTTTAATTATAGAAGAAAAAAATTATATTTTTCAAAATGCTCAAATCGAAATGCAGT
CATTACCGGATGAACATTTATTACACCATTTACGTCTAATACCAAATATTCAGCAGTTGGGGGCCAAGATATTTTATTAATTGGTGTTG
TTTTTGTTGGTTTTGTGTCAACGTTAGGATTTGTAAATTTATTAATTACTCGTAGAACTCTTTGTATGCCTGGATTACGAAATCGACGA
ATTTTATTGCCATTTGTTACAATTACAATTTTATTATCTCTCCGCATGCTAGTATTAATTACTCCAGTTTTAAGCGCCGCAATGATTAT
GATGTTTTTAGATAGACATTGAAATACTTCATTTTTTGATTTTGCATATGGTGGTGATCCAATTTTATCACAGCATTTATTTTGATTTT
TCGGTCACCCT
**>Anoplophrya_vulgaris_NG_28_DV**
GGTTCCAAAGATGTTGCATATCCACGATTGAATAGCATTGCGTTTTGATTGCAACCGTGTGCGTTTATTGTGGTAGTAAAGATGGCATT
TCTTAGACCACAGTATTGACAATATTATAATGATATAGATTTTTTTATTTTATTTTTAGAAAAAACAAAACAAATAAATTATTTTAATA
ATTTTTGGTTACAAAACAATATAAATAATAATGTTGAGAAAAAATTTAAAATAAATTTATTATTTTGAGATTCATTTTTTTAT---TAC
TGTGAGTCTTCATATTTATTTTTTTTTAAACTTTTTAATTATAGAAGAAAAAAATTATATTTTTCAAAATGCTCAAATCGAAATGCAGT
CATTACCGGATGAACATTTATTACACCATTTACGTCTAATACCAAATATTCAGCAGTTGGGGGCCAAGATATTTTATTAATTGGTGTTG
TTTTTGTTGGTTTTGTGTCAACGTTAGGATTTGTAAATTTATTAATTACTCGTAGAACTCTTTGTATGCCTGGATTACGAAATCGACGA
ATTTTATTGCCATTTGTTACAATTACAATTTTATTATCTCTCCGCATGCTAGTATTAATTACTCCAGTTTTAAGCGCCGCAATGATTAT
GATGTTTTTAGATAGACATTGAAATACTTCATTTTTTGATTTTGCATATGGTGGTGATCCAATTTTATCACAGCATTTATTTTGATTTT
TCGGTCACCCT
**>Anoplophrya_lumbrici_RZ_6_LT**
GGTTCCAAAGATGTTGCATACCCCAGGTTGAATAGTATCGCTTTTTGGCTGCAGCCATGCGCATTTCTAGTTGTAGTGAAATTAGCATT
TTTACGGCCCCAGTATTGAAAGTACTATGATGATCTTGATTTTTTTATAACATTTTTGAATAAATATAAAATAGATGGTAAAGTTAATT
CGCCTACTGTGTTATCCGGCGGTGGGCAATCATATCAACAACGGTTGAACATAAATTTATTTTTTTGGGATACTATTTATCAA---TAT
TGTGAGTGAGGTTATTTTTTTTTTAATAATTGGTCTAACTACAGGCGTAAAAAGTTATATTTTTCTAAATGTTCAAACAGAAATTCTGT
TGTAACAGGGTGAACTTTTATTACTCCATTTACTTCGGATACTAGATTTTCTGGTGTAGGGTCACAAGATATCTTACTTATTGGTGTTG
TTTTTGTTGGGTTTACAACAACAATTGGCTTTGTTAACTTACTTGTTACGCGCAGAACTCTTTGTATGCCTGGGTTACGGAATCGTAGA
ATATTACTGCCCTTTGTTACTATTACATTACTTTTGTCTCTGCGAATGCTTGTAATCATAACTCCAGTATTAAGTGCTGCGATGATTAT
GATGCTTCTTGACCGTCATTGGGGGACTTCCTTTTTTGATTTTTCATATGGAGGAGATCCGATTTTGTCACAGCATTTGTTTTGATTTT
TCGGTCACCCT
**>Anoplophrya_lumbrici_KR_9_LT**
GGTTCCAAAGATGTTGCATACCCCAGGTTGAATAGTATCGCTTTTTGGCTGCAGCCATGCGCATTTCTAGTTGTAGTGAAATTAGCATT
TTTACGGCCCCAGTATTGAAAGTACTATGATGATCTTGATTTTTTTATAACATTTTTGAATAAATATAAAATAGATGGTAAAGTTAATT
CGCCTACTGTGTTATCCGGCGGTGGGCAATCATATCAACAACGGTTGAACATAAATTTATTTTTTTGGGATACTATTTATCAA---TAT
TGTGAGTGAGGTTATTTTTTTTTTAATAATTGGTCTAACTACAGGCGTAAAAAGTTATATTTTTCTAAATGTTCAAACAGAAATTCTGT
TGTAACAGGGTGAACTTTTATTACTCCATTTACTTCGGATACTAGATTTTCTGGTGTAGGGTCACAAGATATCTTACTTATTGGTGTTG
TTTTTGTTGGGTTTACAACAACAATTGGCTTTGTTAACTTACTTGTTACGCGCAGAACTCTTTGTATGCCTGGGTTACGGAATCGTAGA
ATATTACTGCCCTTTGTTACTATTACATTACTTTTGTCTCTGCGAATGCTTGTAATCATAACTCCAGTATTAAGTGCTGCGATGATTAT
GATGCTTCTTGACCGTCATTGGGGGACTTCCTTTTTTGATTTTTCATATGGAGGAGATCCGATTTTGTCACAGCATTTGTTTTGATTTT
TCGGTCACCCT
**>Anoplophrya_lumbrici_KR_11_LT**
GGTTCCAAAGATGTTGCATACCCCAGGTTGAATAGTATCGCTTTTTGGCTGCAGCCATGCGCATTTCTAGTTGTAGTGAAATTAGCATT
TTTACGGCCCCAGTATTGAAAGTACTATGATGATCTTGATTTTTTTATAACATTTTTGAATAAATATAAAATAGATGGTAAAGTTAATT
CGCCTACTGTGTTATCCGGCGGTGGGCAATCATATCAACAACGGTTGAACATAAATTTATTTTTTTGGGATACTATTTATCAA---TAT
TGTGAGTGAGGTTATTTTTTTTTTAATAATTGGTCTAACTACAGGCGTAAAAAGTTATATTTTTCTAAATGTTCAAACAGAAATTCTGT
TGTAACAGGGTGAACTTTTATTACTCCATTTACTTCGGATACTAGATTTTCTGGTGTAGGGTCACAAGATATCTTACTTATTGGTGTTG
TTTTTGTTGGGTTTACAACAACAATTGGCTTTGTTAACTTACTTGTTACGCGCAGAACTCTTTGTATGCCTGGGTTACGGAATCGTAGA
ATATTACTGCCCTTTGTTACTATTACATTACTTTTGTCTCTGCGAATGCTTGTAATCATAACTCCAGTATTAAGTGCTGCGATGATTAT
GATGCTTCTTGACCGTCATTGGGGGACTTCCTTTTTTGATTTTTCATATGGAGGAGATCCGATTTTGTCACAGCATTTGTTTTGATTTT
TCGGTCACCCT
**>Anoplophrya_octolasionis_MU_56_OL**
GGTTCCAAAGATGTTGCTTATCCACGCATAAATAGTATTGCTTTCTGATTGCAACCATGCGCGTTTTTAGTTGTTGTTAAAATGGCTTT
TTTACGCCCACAATATTGAAAATATTATGATGATGTAGATTTTTTTGTTTTTTTTTTAAAAAAGTAAAAAATTTTGAAATTTCTAAGA
ATAGTATATCTAATAATACAACATCCGCTTTTTTTTTGGATAAAAATAAATTAAATTTATTTTTTTTAGATTCATTTTTAAAA---TAT
TGTGAATACTCTTATCTTTTTTTCAGTAATTTAACATCTTATAGAAGAAAAAAATTATATTTTTCAAAATGCTCTAACAGAAACTCTGT
TGTCACTGGATGAACATTTATAACACCGTTTACATCTAACACGAAATATTCTGGAGTGGGCTCTCAAGATATTTTATTAATTGGGGTTG
TTTTTGTTGGCTTTACAACTACTATAGGTTTTGTAAATTTATTAATAACTAGACGCACGCTGTGCATGCCGGGTCTCCGTAATCGTAGG
ATACTTCTACCCTTTATTACGATTACACTTTTACTGTCTTTAAGAATGTTAGTAATTATTACGCCAGTTCTTAGTGCAGCTATGATTAT
GATGCTTCTCGATAGGCACTGGTGTACTTCATTTTTTGATTTTGCTTACGGTGGAGATCCGATATTATCTCAGCATCTGTTTTGATTTT

TCGGTCACCCT
**>Anoplophrya_octolasionis_MU_57_OL**
GGTTCCAAAGATGTTGCTTATCCACGCATAAATAGTATTGCTTTCTGATTGCAACCATGCGCGTTTTAGTTGTTGTTAAAATGGCTTT
TTTACGCCCACAATATTGAAAATATTATGATGATGTAGATTTTTTTGTTTTTTTTTTTAAAAAAGTAAAAAATTTTGAAATTTCTAAGA
ATAGTATATCTAATAATACAACATCCGCTTTTTTTTTGGATAAAAATAAATTAAATTTATTTTTTTTAGATTCATTTTTAAAA---TAT
TGTGAATACTCTTATCTTTTTTTCAGTAATTTAACATCTTATAGAAGAAAAAAATTATATTTTTCAAAATGCTCTAACAGAAACTCTGT
TGTCACTGGATGAACATTTATAACACCGTTTACATCTAACACGAAATATTCTGGAGTGGGCTCTCAAGATATTTTATTAATTGGGGTTG
TTTTTGTTGGCTTTACAACTACTATAGGTTTTGTAAATTTATTAATAACTAGACGCACGCTGTGCATGCCGGGTCTCCGTAATCGTAGG
ATACTTCTACCCTTTATTACGATTACACTTTTACTGTCTTTAAGAATGTTAGTAATTATTACGCCAGTTCTTAGTGCAGCTATGATTAT
GATGCTTCTCGATAGGCACTGGTGTACTTCATTTTTTGATTTTGCTTACGGTGGAGATCCGATATTATCTCAGCATCTGTTTTGATTTT
TCGGTCACCCT
**>Anoplophrya_octolasionis_MU_58_OL**
GGTTCCAAAGATGTTGCTTATCCACGCATAAATAGTATTGCTTTCTGATTGCAACCATGCGCGTTTTAGTTGTTGTTAAAATGGCTTT
TTTACGCCCACAATATTGAAAATATTATGATGATGTAGATTTTTTTGTTTTTTTTTTTAAAAAAGTAAAAAATTTTGAAATTTCTAAGA
ATAGTATATCTAATAATACAACATCCGCTTTTTTTTTGGATAAAAATAAATTAAATTTATTTTTTTTAGATTCATTTTTAAAA---TAT
TGTGAATACTCTTATCTTTTTTTCAGTAATTTAACATCTTATAGAAGAAAAAAATTATATTTTTCAAAATGCTCTAACAGAAACTCTGT
TGTCACTGGATGAACATTTATAACACCGTTTACATCTAACACGAAATATTCTGGAGTGGGCTCTCAAGATATTTTATTAATTGGGGTTG
TTTTTGTTGGCTTTACAACTACTATAGGTTTTGTAAATTTATTAATAACTAGACGCACGCTGTGCATGCCGGGTCTCCGTAATCGTAGG
ATACTTCTACCCTTTATTACGATTACACTTTTACTGTCTTTAAGAATGTTAGTAATTATTACGCCAGTTCTTAGTGCAGCTATGATTAT
GATGCTTCTCGATAGGCACTGGTGTACTTCATTTTTTGATTTTGCTTACGGTGGAGATCCGATATTATCTCAGCATCTGTTTTGATTTT
TCGGTCACCCT
**>Maupasella_mucronata_KDo_33_ET**
GGTTCCAAAGATGTTGCGTACCCAAGGCTGAACAGTATCGCTTTTTGACTACAGCCAGTAGCGCTAGTAGCAGTTCTTAAGACAATAAT
TTTACGACAACAATTTTGAACTAACAAAAACATGCCAGAAAGTTATTTCAACTATTTAAAAAAAATCAATGATAATACGACTGCTACAC
CAGTAAGAATAAATACAAGG--------------------------CGTGTTTTAATAGACTGGTGGTATTGTAGAATACAAGCACAG
ACTGACTTTTATTGATTTTTTGTATCACAAAAAACTAAAAATCTAACAAAAAAATTATACATGTCTAAATACAGCGACAGAGCAGCCGT
TACCACAGGATGGACTTTTGTGACACCATTTACGTCTAGCACAAAATATTCTAGCGTTGGCGCACAAGATATGTTATTATTGGGTGTAG
TTTTCGTAGGAGTGACTAGTACTGTTTCGTTCGTGAATTTACTAATAACTAAAAGAACCTTATGTATGCCTGGTATAAGATCACGTCGC
GTACTGCTACCCTTCATAACCATAAATATTTTGTTGTCGCTACGTATGTTGGCTATAATAACACCTGTATTAAGCGCGGCTATGATAAT
GATGCTTCTTGATAGACATTGGAACACTTCCTTCTTTGATTTTACATACGGAGGAGATCCTATACTATCACAACATTTATTTTGATTTT
TCGGTCACCCT
**>Maupasella_mucronata_KDo_34_ET**
GGTTCCAAAGATGTTGCGTACCCAAGGCTGAACAGTATCGCTTTTTGACTACAGCCAGTAGCGCTAGTAGCAGTTCTTAAGACAATAAT
TTTACGACAACAATTTTGAACTAACAAAAACATGCCAGAAAGTTATTTCAACTATTTAAAAAAAATCAATGATAATACGACTGCTACAC
CAGTAAGAATAAATACAAGG--------------------------CGTGTTTTAATAGACTGGTGGTATTGTAGAATACAAGCACAG
ACTGACTTTTATTGATTTTTTGTATCACAAAAAACTAAAAATCTAACAAAAAAATTATACATGTCTAAATACAGCGACAGAGCAGCCGT
TACCACAGGATGGACTTTTGTGACACCATTTACGTCTAGCACAAAATATTCTAGCGTTGGCGCACAAGATATGTTATTATTGGGTGTAG
TTTTCGTAGGAGTGACTAGTACTGTTTCGTTCGTGAATTTACTAATAACTAAAAGAACCTTATGTATGCCTGGTATAAGATCACGTCGC
GTACTGCTACCCTTCATAACCATAAATATTTTGTTGTCGCTACGTATGTTGGCTATAATAACACCTGTATTAAGCGCGGCTATGATAAT
GATGCTTCTTGATAGACATTGGAACACTTCCTTCTTTGATTTTACATACGGAGGAGATCCTATACTATCACAACATTTATTTTGATTTT
TCGGTCACCCT
**>Maupasella_mucronata_KDo_35_ET**
GGTTCCAAAGATGTTGCGTACCCAAGGCTGAACAGTATCGCTTTTTGACTACAGCCAGTAGCGCTAGTAGCAGTTCTTAAGACAATAAT
TTTACGACAACAATTTTGAACTAACAAAAACATGCCAGAAAGTTATTTCAACTATTTAAAAAAAATCAATGATAATACGACTGCTACAC
CAGTAAGAATAAATACAAGG--------------------------CGTGTTTTAATAGACTGGTGGTATTGTAGAATACAAGCACAG
ACTGACTTTTATTGATTTTTTGTATCACAAAAAACTAAAAATCTAACAAAAAAATTATACATGTCTAAATACAGCGACAGAGCAGCCGT
TACCACAGGATGGACTTTTGTGACACCATTTACGTCTAGCACAAAATATTCTAGCGTTGGCGCACAAGATATGTTATTATTGGGTGTAG
TTTTCGTAGGAGTGACTAGTACTGTTTCGTTCGTGAATTTACTAATAACTAAAAGAACCTTATGTATGCCTGGTATAAGATCACGTCGC
GTACTGCTACCCTTCATAACCATAAATATTTTGTTGTCGCTACGTATGTTGGCTATAATAACACCTGTATTAAGCGCGGCTATGATAAT
GATGCTTCTTGATAGACATTGGAACACTTCCTTCTTTGATTTTACATACGGAGGAGATCCTATACTATCACAACATTTATTTTGATTTT
TCGGTCACCCT
**>Maupasella_mucronata_KDo_36_ET**
GGTTCCAAAGATGTTGCGTACCCAAGGCTGAACAGTATCGCTTTTTGACTACAGCCAGTAGCGCTAGTAGCAGTTCTTAAGACAATAAT
TTTACGACAACAATTTTGAACTAACAAAAACATGCCAGAAAGTTATTTCAACTATTTAAAAAAAATCAATGATAATACGACTGCTACAC
CAGTAAGAATAAATACAAGG--------------------------CGTGTTTTAATAGACTGGTGGTATTGTAGAATACAAGCACAG
ACTGACTTTTATTGATTTTTTGTATCACAAAAAACTAAAAATCTAACAAAAAAATTATACATGTCTAAATACAGCGACAGAGCAGCCGT
TACCACAGGATGGACTTTTGTGACACCATTTACGTCTAGCACAAAATATTCTAGCGTTGGCGCACAAGATATGTTATTATTGGGTGTAG
TTTTCGTAGGAGTGACTAGTACTGTTTCGTTCGTGAATTTACTAATAACTAAAAGAACCTTATGTATGCCTGGTATAAGATCACGTCGC
GTACTGCTACCCTTCATAACCATAAATATTTTGTTGTCGCTACGTATGTTGGCTATAATAACACCTGTATTAAGCGCGGCTATGATAAT
GATGCTTCTTGATAGACATTGGAACACTTCCTTCTTTGATTTTACATACGGAGGAGATCCTATACTATCACAACATTTATTTTGATTTT
TCGGTCACCCT
**>Metaradiophrya_chlorotica_JA2_1M_ACH**
GGTTCCAAAGATGTTGCTTATCCAAGATTAAATAGTATTGCATTTTGATTGCAACCATGTGCTTTTATTGTAGTAGTTAAAATGGCATT
TTTGCGAACGCAGTATTGAAAATATTATGATGATATGGAATTTTTTGTATTTTTTTTTAACAAATTGAAAAAAAAAGAATTACAATTAA
AAAACAATATAGATGCGTCTAATTCAATTTGATTAAATTCTAAAATACCAAGTCATTTGTATTTATGAGATACATTTTTTAAT---TAT
TGTGAAAATTTTTTTTTATTTTTTAAACGTACTTCTGAATACAGAAGAAAAAAATTATATTTTTCTAAATGTTCTAATAGAAACGCAGT
TGTAACAGGATGAACATTTATTACTCCATTTACATCTAGTGTTAGGTTTACTAGTGTAGGTTCTCAAGATATTTTGCTAATAGGTGTTG
TTTTTGTAGGATTTACCAGTACAATTGCATTTGTAAATTTGTTAATAACCAGAAGAACATTATGTATGCCTGGAATTAGAAATAGGCGA
ATTCTTTTACCGTTTGTTTCGATTAGTATATTATTATCATTAAGGATGTTAGTTATTATTACTCCTGTATTAAGTGCAGCTATGATTAT
GATGTTATTAGATCGGCATTGAGCAACTTCATTTTTTGATTTTGCATATGGTGGTGATCCAATTTTGTCTCAGCATCTTTTCTGATTTT
TCGGTCACCCT

**>Metaradiophrya_chlorotica_JA2_2M_ACH**
GGTTCCAAAGATGTTGCTTATCCAAGATTAAATAGTATTGCATTTTGATTGCAACCATGTGCTTTTATTGTAGTAGTTAAAATGGCATT
TTTGCGAACGCAGTATTGAAAATATTATGATGATATGGAATTTTTTGTATTTTTTTTTAACAAATTGAAAAAAAAAGAATTACAATTAA
AAAACAATATAGATGCGTCTAATTCAATTTGATTAAATTCTAAAATACCAAGTCATTTGTATTTATGAGATACATTTTTTAAT---TAT
TGTGAAAATTTTTTTTTATTTTTTAAACGTACTTCTGAATACAGAAGAAAAAAATTATATTTTTCTAAATGTTCTAATAGAAACGCAGT
TGTAACAGGATGAACATTTATTACTCCATTTACATCTAGTGTTAGGTTTACTAGTGTAGGTTCTCAAGATATTTTGCTAATAGGTGTTG
TTTTTGTAGGATTTACCAGTACAATTGCATTTGTAAATTTGTTAATAACCAGAAGAACATTATGTATGCCTGGAATTAGAAATAGGCGA
ATTCTTTTACCGTTTGTTTCGATTAGTATATTATTATCATTAAGGATGTTAGTTATTATTACTCCTGTATTAAGTGCAGCTATGATTAT
GATGTTATTAGATCGGCATTGAGCAACTTCATTTTTTGATTTTGCATATGGTGGTGATCCAATTTTGTCTCAGCATCTTTTCTGATTTT
TCGGTCACCCT
**>Metaradiophrya_chlorotica_JA2_3M_ACH**
GGTTCCAAAGATGTTGCTTATCCAAGATTAAATAGTATTGCATTTTGATTGCAACCATGTGCTTTTATTGTAGTAGTTAAAATGGCATT
TTTGCGAACGCAGTATTGAAAATATTATGATGATATGGAATTTTTTGTATTTTTTTTTAACAAATTGAAAAAAAAAGAATTACAATTAA
AAAACAATATAGATGCGTCTAATTCAATTTGATTAAATTCTAAAATACCAAGTCATTTGTATTTATGAGATACATTTTTTAAT---TAT
TGTGAAAATTTTTTTTTATTTTTTAAACGTACTTCTGAATACAGAAGAAAAAAATTATATTTTTCTAAATGTTCTAATAGAAACGCAGT
TGTAACAGGATGAACATTTATTACTCCATTTACATCTAGTGTTAGGTTTACTAGTGTAGGTTCTCAAGATATTTTGCTAATAGGTGTTG
TTTTTGTAGGATTTACCAGTACAATTGCATTTGTAAATTTGTTAATAACCAGAAGAACATTATGTATGCCTGGAATTAGAAATAGGCGA
ATTCTTTTACCGTTTGTTTCGATTAGTATATTATTATCATTAAGGATGTTAGTTATTATTACTCCTGTATTAAGTGCAGCTATGATTAT
GATGTTATTAGATCGGCATTGAGCAACTTCATTTTTTGATTTTGCATATGGTGGTGATCCAATTTTGTCTCAGCATCTTTTCTGATTTT
TCGGTCACCCT
**>Metaradiophrya_lumbrici_RZ_4_LT**
GGTTCCAAAGATGTTGCTTACCCTCGATTAAATAGTATTGCGTTTTGATTACAACCATGTGCTTTTATTGTGGTGGTAAAAATGGCTTT
TTTGCGTACTCAATATTGGAAATATTATGATGATATGGAGTTTTTTGTTTTTTTTTTTGATAAATTAAAAAAAAATAAAAACCAACTTA
TCATAACATATGAAAGAAGTAATAACGAATGATTGACTCCGAAAAATCCTAATTATTTATATTTTTGGGATAGTTTTTTTAAT---TAT
GGCGAAAATTTTTTTTTTTTTTTTAATAGATCATCGGCATATAGGCGAAAAAAATTATATTTTTCTAAATGTTCTAATAGAAATGCAGT
AGTAACGGGATGAACTTTTATTACTCCATTTACATCTAATACTAGATTTACAAGTGTTGGTTCTCAAGATATTTTATTAATTGGAGTCG
TGTTTGTAGGTTTTACAAGTACGATTGCGTTTGTTAATTTATTAATAACACGACGAACCTTGTGTATGCCAGGAATTAGAAATCGTCGT
ATTCTTTTGCCGTTTGTTTCTATTAGTTTACTTTTATCTTTGCGGATGTTAATCATAATTACCCCAGTTTTAAGTGCCGCTATGATAAT
GATGCTTTTAGATAGACATTGAGCCACTTCTTTTTTTGATTTTGCGTATGGGGGAGATCCTATTTTATCACAGCATTTATTTTGATTTT
TCGGTCACCCT
**>Metaradiophrya_lumbrici_RZ_5_LT**
GGTTCCAAAGATGTTGCTTACCCTCGATTAAATAGTATTGCGTTTTGATTACAACCATGTGCTTTTATTGTGGTGGTAAAAATGGCTTT
TTTGCGTACTCAATATTGGAAATATTATGATGATATGGAGTTTTTTGTTTTTTTTTTTGATAAATTAAAAAAAAATAAAAACCAACTTA
TCATAACATATGAAAGAAGTAATAACGAATGATTGACTCCGAAAAATCCTAATTATTTATATTTTTGGGATAGTTTTTTTAAT---TAT
GGCGAAAATTTTTTTTTTTTTTTTAATAGATCATCGGCATATAGGCGAAAAAAATTATATTTTTCTAAATGTTCTAATAGAAATGCAGT
AGTAACAGGATGAACTTTTATTACTCCATTTACATCTAATACTAGATTTACAAGTGTTGGTTCTCAAGATATTTTATTAATTGGAGTCG
TGTTTGTGGGTTTTACAAGTACGATTGCGTTTGTTAATTTATTAATAACCCGACGAACCTTGTGTATGCCAGGAATTAGAAATCGTCGT
ATTCTTTTGCCGTTTGTTTCTATTAGTTTACTTTTATCTTTGCGGATGTTAATCATAATTACCCCAGTTTTAAGTGCCGCTATGATAAT
GATGCTTTTAGATAGACATTGAGCCACTTCTTTTTTTGATTTTGCGTATGGGGGAGATCCTATTTTATCACAGCATTTATTTTGATTTT
TCGGTCACCCT
**>Metaradiophrya_lumbrici_KR_8_LT**
GGTTCCAAAGATGTTGCTTACCCTCGATTAAATAGTATTGCGTTTTGATTACAACCATGTGCTTTTATTGTGGTGGTAAAAATGGCTTT
TTTGCGTACTCAATATTGGAAATATTATGATGATATGGAGTTTTTTGTTTTTTTTTTTGATAAATTAAAAAAAAATAAAAACCAACTTA
TCATAACATATGAAAGAAGTAATAACGAATGATTGACTCCGAAAAATCCTAATTATTTATATTTTTGGGATAGTTTTTTTAAT---TAT
GGTGAAAATTTTTTTTTTTTTTTTAATAGATCGTCGGCGTATAGACGAAAAAAATTATATTTTTCTAAATGTTCTAATAGAAATGCAGT
AGTAACGGGATGAACTTTTATTACTCCATTTACATCTAATACTAGATTTACAAGTGTTGGTTCTCAAGATATTTTATTAATTGGAGTCG
TGTTTGTAGGTTTTACAAGTACGATTGCGTTTGTTAATTTATTAATAACCCGACGAACCTTGTGTATGCCAGGAATTAGAAATCGTCGT
ATTCTTTTGCCGTTTGTTTCTATTAGTTTACTTTTATCTTTGCGGATGTTAATCATAATTACCCCAGTTTTAAGTGCTGCTATGATAAT
GATGCTTTTAGATAGACATTGAGCCACTTCTTTTTTTGATTTTGCGTATGGGGGAGATCCTATTTTATCACAGCATTTATTTTGATTTT
TCGGTCACCCT
**>Metaradiophrya_lumbrici_KR_10_LT**
GGTTCCAAAGATGTTGCTTACCCTCGATTAAATAGTATTGCGTTTTGATTACAACCATGTGCTTTTATTGTGGTGGTAAAAATGGCTTT
TTTGCGTACTCAATATTGGAAATATTATGATGATATGGAGTTTTTTGTTTTTTTTTTTGATAAATTAAAAAAAAATAAAAACCAACTTA
TCATAACATATGAAAGAAGTAATAACGAATGATTGACTCCGAAAAATCCTAATTATTTATATTTTTGGGATAGTTTTTTTAAT---TAT
GGGGAAAATTTTTTTTTTTTTTTTAATAGATCGTCGGCGTATAGACGAAAAAAATTATATTTTTCTAAATGTTCTAATAGAAATGCAGT
AGTAACAGGATGAACTTTTATTACTCCATTTACATCTAATACTAGATTTACAAGTGTTGGTTCTCAAGATATTTTATTAATTGGAGTCG
TGTTTGTAGGTTTTACAAGTACGATTGCGTTTGTTAATTTATTAATAACACGACGAACCTTGTGTATGCCAGGAATTAGAAATCGTCGT
ATTCTTTTGCCGTTTGTTTCTATTAGTTTACTTTTATCTTTGCGGATGTTAATCATAATTACCCCAGTTTTAAGTGCTGCTATGATAAT
GATGCTTTTAGATAGACATTGAGCCACTTCTTTTTTTGATTTTGCGTATGGGGGAGATCCTATTTTATCACAGCATTTATTTTGATTTT
TCGGTCACCCT
**>Metaradiophrya_lumbrici_JA2_25_LT**
GGTTCCAAAGATGTTGCTTACCCTCGATTAAATAGTATTGCGTTTTGATTACAACCATGTGCTTTTATTGTGGTGGTAAAAATGGCTTT
TTTGCGTACTCAATATTGGAAATATTATGATGATATGGAGTTTTTTGTTTTTTTTTTTGATAAATTAAAAAAAAATAAAAACCAACTTA
TCATAACATATGAAAGAAGTAATAACGAATGATTGACTCCGAAAAATCCTAATTATTTATATTTTTGGGATAGTTTTTTTAAT---TAT
GGCGAAAATTTTTTTTTTTTTTTTAATAGATCATCGGCTTATAGGCGAAAAAAATTATATTTTTCTAAATGTTCTAATAGAAATGCAGT
AGTAACGGGATGAACTTTTATTACTCCATTTACATCTAATACTAGATTTACAAGTGTTGGTTCTCAAGATATTTTATTAATTGGAGTCG
TGTTTGTGGGTTTTACAAGTACGATTGCGTTTGTTAATTTATTAATAACCCGACGAACCTTGTGTATGCCAGGAATTAGAAATCGTCGT
ATTCTTTTGCCGTTTGTTTCTATTAGTTTACTTTTATCTTTGCGGATGTTAATCATAATTACCCCAGTTTTAAGTGCCGCTATGATAAT
GATGCTTTTAGATAGACATTGAGCCACTTCTTTTTTTGATTTTGCGTATGGGGGAGATCCTATTTTATCACAGCATTTATTTTGATTTT
TCGGTCACCCT

**>Metaradiophrya_lumbrici_JA2_26_LT**
GGTTCCAAAGATGTTGCTTACCCTCGATTAAATAGTATTGCGTTTTGATTACAACCATGTGCTTTTATTGTGGTGGTAAAAATGGCTTT
TTTGCGTACTCAATATTGGAAATATTATGATGATATGGAGTTTTTTGTTTTTTTTTTTGATAAATTAAAAAAAAATAAAAACCAACTTA
TCATAACATATGAAAGAAGTAATAACGAATGATTGACTCCGAAAAATCCTAATTATTTATATTTTTGGGATAGTTTTTTTAAT---TAT
GGCGAAAATTTTTTTTTTTTTTTTAATAGATCATCGGCATATAGGCGAAAAAAATTATATTTTTCTAAATGTTCTAATAGAAATGCAGT
AGTAACGGGATGAACTTTTATTACTCCATTTACATCTAATACTAGATTTACAAGTGTTGGTTCTCAAGATATTTTATTAATTGGAGTCG
TGTTTGTGGGTTTTACAAGTACGATTGCGTTTGTTAATTTATTAATAACCCGACGAACCTTGTGTATGCCAGGAATTAGAAATCGTCGT
ATTCTTTTGCCGTTTGTTTCTATTAGTTTACTTTTATCTTTGCGGATGTTAATCATAATTACCCCAGTTTTAAGTGCCGCTATGATAAT
GATGCTTTTAGATAGACATTGAGCCACTTCTTTTTTTGATTTTGCGTATGGGGGAGATCCTATTTTATCACAGCATTTATTTTGATTTT
TCGGTCACCCT
**>Metaradiophrya_speculorum_HkD_59_AT**
GGTTCCAAAGATGTTGCGTACCCGCGGCTTAATTGTATTGCGTTTTGATTGCAACCTTGCGCATTTCTTGTAGTAGTCAAAATGGGATT
TTTGCGCGCGCAATATTGAAAGTATTATGATGACATTGAATTTTTTATGTTTTTTTTTAATAAGATAAAATTGAATGGCAATTCTACAG
TAGAGAGTAAGCTTAATAATAGTGCTGCTTTTTTAGACTTTAAAGCACCGCTATATTTACGATTGTGGGATTCTTTTTTTTTA---TAT
TGTGAAAATTTTTTTATTTTTTTTAATCGAAATTCTGCGTATAGGAGAAAAAAATTATACTTTACAAAATGCTCTAACCGAAATGCTGT
AGTGACTGGATGGACCTTTATAACACCGTTTACGTCTAGTACACGGTTTACTAGTGTCGGTTCTCAAGATATTTTATTAATAGGTGTTG
TGTTTGTTGGTTTTACTAGTACGATAGCGTTTGTTAATTTGTTAATAACTCGTCGAACTTTATGTATGCCTGGTATTAGGAATAGACGA
ATCTTATTGCCATTTATTACGATTAGTTTACTTTTGTCTTTGCGTATGCTAGTAATTATTACGCCTGTTTTGAGTGCAGCAATGATAAT
GATGCTTTTAGATAGGCATTGGGCTACGGCATTTTTTGATTTTGCGTATGGTGGTGATCCTATATTGTCGCAGCATATGTTTTGATTTT
TCGGTCACCCT
**>Metaradiophrya_speculorum_HkD_60_AT**
GGTTCCAAAGATGTTGCGTACCCGCGGCTTAATTGTATTGCGTTTTGATTGCAACCTTGCGCATTTCTTGTAGTAGTCAAAATGGGATT
TTTGCGCGCGCAATATTGAAAGTATTATGATGACATTGAATTTTTTATGTTTTTTTTTAATAAGATAAAATTGAATGGCAATTCTACAG
TAGAGAGTAAGCTTAATAATAGTGCTGCTTTTTTAGACTTTAAAGCACCGCTATATTTACGATTGTGGGATTCTTTTTTTTTA---TAT
TGTGAAAATTTTTTTATTTTTTTTAATCGAAATTCTGCGTATAGGAGAAAAAAATTATACTTTACAAAATGCTCTAACCGAAATGCTGT
AGTGACTGGATGGACCTTTATAACACCGTTTACGTCTAGTACACGGTTTACTAGTGTCGGTTCTCAAGATATTTTATTAATAGGTGTTG
TGTTTGTTGGTTTTACTAGTACGATAGCGTTTGTTAATTTGTTAATAACTCGTCGAACTTTATGTATGCCTGGTATTAGGAATAGACGA
ATCTTATTGCCATTTATTACGATTAGTTTACTTTTGTCTTTGCGTATGCTAGTAATTATTACGCCTGTTTTGAGTGCAGCAATGATAAT
GATGCTTTTAGATAGGCATTGGGCTACGGCATTTTTTGATTTTGCGTATGGTGGTGATCCTATATTGTCGCAGCATATGTTTTGATTTT
TCGGTCACCCT
**>Metaradiophrya_varians_BZ_12_EF**
GGTTCCAAAGATGTTGCATATCCACGTTTAAATAGTATTGCATTTTGATTACAACCTTGTGCTTTTTTAGTAGTCGTAAAAATGGCATT
TTTAAAATCTCAATATTGAAAATATTATGATGATATTGAATTTTTTATTTCTTTTCTTAATAAATTAAAATATACCAATATAAATATTA
ATAATAATTTTAAATTAGAAGTTCAAAAACCATTA------AAGAAAAAATATGAATATGTATTTATGAAATGATATTTTTTAAT---TAC
TGTGAAAATTTTTTTTATTTTTTTCACGTACGTCTTCATATCGACGAAAGAAACTTTATTACTCAAAATGTTCAAATCGTAATGCAGT
TATAACTGGATGAACATTTATAACACCATTTACATCTAGTACTCGATATAGTAGTACAGGATCTCAAGATATATTACTTATTGGGGTTG
TATTTGTTGGTTTTACTAGTACTGTTGCTTTTGTAAATTTATTAATAACAAGACGTACCTTATGTATGCCAGGTATTCGAAATCGTCGA
ATTTTATTACCTTTTGTAACTATTAGTATGTTATTATCTTTAAGAATGTTAGTAATTATTACTCCTGTTTTAAGTGCCGCAATGATTAT
GATGTTATTAGATAGACATTTTGGAACATCCTTTTTTGATTTTGCGTATGGTGGTGATCCAATTTTATCTCAGCATTTATTTTGATTTT
TCGGTCACCCT
**>Metaradiophrya_varians_BZ_14_EF**
GGTTCCAAAGATGTTGCATATCCACGTTTAAATAGTATTGCATTTTGATTACAACCTTGTGCTTTTTTAGTAGTCGTAAAAATGGCTTT
TTTAAAATCTCAATATTGAAAATATTATGATGATATTGAATTTTTTATTTCTTTTCTTAATAAATTAAAATATACCAATATAAATATTA
ATAATAATTTTAAATTAGAAGTTCAAAAACCATTA------AAGAAAAAATATGAATATGTATTTATGAAATGATATTTTTTAAT---TAC
TGTGAAAATTTTTTTTATTTTTTTCACGTACGTCTTCATATCGACGAAAGAAACTTTATTACTCAAAATGTTCAAATCGTAATGCAGT
TATAACTGGATGAACATTTATAACACCATTTACATCTAGTACTCGATATAGTAGTACAGGATCTCAAGATATATTACTTATTGGGGTTG
TATTTGTTGGTTTTACTAGTACTGTTGCTTTTGTAAATTTATTAATAACAAGACGTACCTTATGTATGCCAGGTATTCGAAATCGTCGA
ATTTTATTACCTTTTGTAACTATTAGTATGTTATTATCTTTAAGAATGTTAGTAATTATTACTCCTGTTTTAAGTGCGGCAATGATTAT
GATGTTATTAGATAGACATTTTGGAACATCCTTTTTTGATTTTGCGTATGGTGGTGATCCAATTTTATCTCAGCATTTATTTTGATTTT
TCGGTCACCCT
**>Metaradiophrya_varians_JA1_19_EF**
GGTTCCAAAGATGTTGCATATCCACGTTTAAATAGTATTGCATTTTGATTACAACCTTGTGCTTTTTTAGTAGTCGTAAAAATGGCATT
TTTAAAATCTCAATATTGAAAATATTATGATGATATTGAATTTTTTATTTCTTTTCTTAATAAATTAAAATATACCAATATAAATATTA
ATAATAATTTTAAATTAGAAGTTCAAAAACCATTA------AAGAAAAAATATGAATATGTATTTATGAAATGATATTTTTTAAT---TAC
TGTGAAAATTTTTTTTATTTTTTTCACGTACGTCTTCATATCGACGAAAGAAACTTTATTACTCAAAATGTTCAAATCGTAATGCAGT
TATAACTGGATGAACATTTATAACACCATTTACATCTAGTACTCGATATAGTAGTACAGGATCTCAAGATATATTACTTATTGGGGTTG
TATTTGTTGGTTTTACTAGTACTGTTGCTTTTGTAAATTTATTAATAACAAGACGTACCTTATGTATGCCAGGTATTCGAAATCGTCGA
ATTTTATTACCTTTTGTAACTATTAGTATGTTATTATCTTTAAGAATGTTAGTAATTATTACTCCTGTTTTAAGTGCCGCAATGATTAT
GATGTTATTAGATAGACATTTTGGAACATCCTTTTTTGATTTTGCGTATGGTGGTGATCCAATTTTATCTCAGCATTTATTTTGATTTT
TCGGTCACCCT
**>Metaradiophrya_varians_JA1_22_EF**
GGTTCCAAAGATGTTGCATATCCACGTTTAAATAGTATTGCATTTTGATTACAACCTTGTGCTTTTTTAGTAGTCGTAAAAATGGCATT
TTTAAAATCTCAATATTGAAAATATTATGATGATATTGAATTTTTTATTTCTTTTCTTAATAAATTAAAATATACCAATATAAATATTA
ATAATAATTTTAAATTAGAAGTTCAAAAACCATTA------AAGAAAAAATATGAATATGTATTTATGAAATGATATTTTTTAAT---TAC
TGTGAAAATTTTTTTTATTTTTTTCACGTACGTCTTCATATCGACGAAAGAAACTTTATTACTCAAAATGTTCAAATCGTAATGCAGT
TATAACTGGATGAACATTTATAACACCATTTACATCTAGTACTCGATATAGTAGTACAGGATCTCAAGATATATTACTTATTGGGGTTG
TATTTGTTGGTTTTACTAGTACTGTTGCTTTTGTAAATTTATTAATAACAAGACGTACCTTATGTATGCCAGGTATTCGAAATCGTCGA
ATTTTATTACCTTTTGTAACTATTAGTATGTTATTATCTTTAAGAATGTTAGTAATTATTACTCCTGTTTTAAGTGCCGCAATGATTAT
GATGTTATTAGATAGACATTTTGGAACATCCTTTTTTGATTTTGCGTATGGTGGTGATCCAATTTTATCTCAGCATTTATTTTGATTTT
TCGGTCACCCT

**>Metaradiophrya_varians_BZkv_31_EF**
GGTTCCAAAGATGTTGCATATCCACGTTTAAATAGTATTGCATTTTGATTACAACCTTGTGCTTTTTTAGTAGTCGTAAAAATGGCATT
TTTAAAATCTCAATATTGAAAATATTATGATGATATTGAATTTTTTATTTCTTTTCTTAATAAATTAAAATATACCAATATAAATATTA
ATAATAATTTTAAATTAGAAGTTCAAAAACCATTA------AAGAAAAAATATGAATATGTATTTATGAAATGATATTTTTTAAT---TAC
TGTGAAAATTTTTTTTTATTTTTTTCACGTACGTCTTCATATCGACGAAAGAAACTTTATTACTCAAAATGTTCAAATCGTAATGCAGT
TATAACTGGATGAACATTTATAACACCATTTACATCTAGTACTCGATATAGTAGTACAGGATCTCAAGATATATTACTTATTGGGGTTG
TATTTGTTGGTTTTACTAGTACTGTTGCTTTTGTAAATTTATTAATAACAAGACGTACCTTATGTATGCCAGGTATTCGAAATCGTCGA
ATTTTATTACCTTTTGTAACTATTAGTATGTTATTATCTTTAAGAATGTTAGTAATTATTACTCCTGTTTTAAGTGCCGCAATGATTAT
GATGTTATTAGATAGACATTTTGGAACATCCTTTTTTGATTTTGCGTATGGTGGTGATCCAATTTTATCTCAGCATTTATTTTGATTTT
TCGGTCACCCT

**>Metaradiophrya_varians_BZkv_32_EF**
GGTTCCAAAGATGTTGCATATCCACGTTTAAATAGTATTGCATTTTGATTACAACCTTGTGCTTTTTTAGTAGTCGTAAAAATGGCATT
TTTAAAATCTCAATATTGAAAATATTATGATGATATTGAATTTTTTATTTCTTTTCTTAATAAATTAAAATATACCAATATAAATATTA
ATAATAATTTTAAATTAGAAGTTCAAAAACCATTA------AAGAAAAAATATGAATATGTATTTATGAAATGATATTTTTTAAT---TAC
TGTGAAAATTTTTTTTTATTTTTTTCACGTACGTCTTCATATCGACGAAAGAAACTTTATTACTCAAAATGTTCAAATCGTAATGCAGT
TATAACTGGATGAACATTTATAACACCATTTACATCTAGTACTCGATATAGTAGTACAGGATCTCAAGATATATTACTTATTGGGGTTG
TATTTGTTGGTTTTACTAGTACTGTTGCTTTTGTAAATTTATTAATAACAAGACGTACCTTATGTATGCCAGGTATTCGAAATCGTCGA
ATTTTATTACCTTTTGTAACTATTAGTATGTTATTATCTTTAAGAATGTTAGTAATTATTACTCCTGTTTTAAGTGCCGCAATGATTAT
GATGTTATTAGATAGACATTTTGGAACATCCTTTTTTGATTTTGCGTATGGTGGTGATCCAATTTTATCTCAGCATTTATTTTGATTTT
TCGGTCACCCT

## REFERENCES


Folmer, O., Black, M., Hoeh, W., Lutz, R., and Vrijenhoek, R. (1994). DNA primers for amplification of mitochondrial cytochrome *c* oxidase subunit I from diverse metazoan invertebrates. *Mol. Mar. Biol. Biotechnol.* 3, 294–299.

Kolicka, M. (2019). New *Chaetonotus* (*Wolterecka*) *semovitus* sp. nov. (Gastrotricha: Chaetonotida: Chaetonotidae) from a palm house in Vienna (Austria). *Ann. Zool.* 69, 447–475. doi: 10.3161/00034541ANZ2019.69.2.011.

Medlin, L., Elwood, H. J., Stickel, S., and Sogin, M. L. (1988). The characterization of enzymatically amplified eukaryotic 16S-like rRNA-coding regions. *Gene* 71, 491–499. doi: 10.1016/0378-1119(88)90066-2

Miao, M., Warren, A., Song, W., Wang, S., Shang, H., and Chen, Z. (2008). Analysis of the internal transcribed spacer 2 (ITS2) region of scuticociliates and related taxa (Ciliophora, Oligohymenophorea) to infer their evolution and phylogeny. *Protist* 159, 519–533. doi: 10.1016/j.protis.2008.05.002

Obert, T., and Vďačný, P. (2019). Integrative taxonomy of five astome ciliates (Ciliophora, Astomatia) isolated from earthworms in Central Europe. *Eur. J. Taxon*. 559, 1–37. doi: 10.5852/ejt.2019.559

Obert, T., and Vďačný, P. (2020). Delimitation of five astome ciliate species isolated from the digestive tube of three ecologically different groups of lumbricid earthworms, using the internal transcribed spacer region and the hypervariable D1/D2 region of the 28S rRNA gene. *BMC Evol. Biol*. 20, e37. doi: 10.1186/s12862-020-1601-2

Pawlowski, J. (2000). Introduction to the molecular systematics of foraminifera. *Micropaleontology* 46 (Suppl. 1), 1–12.

Pérez-Losada, M., Ricoy, M., Marshall, J. C., and Domínguez, J. (2009). Phylogenetic assessment of the earthworm *Aporrectodea caliginosa* species complex (Oligochaeta: Lumbricidae) based on mitochondrial and nuclear DNA sequences. *Mol. Phylogenet. Evol.* 52, 293–302. doi: 10.1016/j.ympev.2009.04.003

Rataj, M., and Vďačný, P. (2020). Multi-gene phylogeny of *Tetrahymena* refreshed with three new histophagous species invading freshwater planarians. *Parasitol. Res*. 119, 1523–1545. doi: 10.1007/s00436-020-06628-0

Strüder-Kypke, M. C., and Lynn, D. H. (2010). Comparative analysis of the mitochondrial cytochrome *c* oxidase subunit I (COI) gene in ciliates (Alveolata, Ciliophora) and evaluation of its suitability as a biodiversity marker. *System. Biodivers*. 8, 131–148. doi: 10.1080/14772000903507744

van Hoek, A. H. A. M., Akhmanova, A. S., Huynen, M. A., and Hackstein, J. H. P. (2000). A mitochondrial ancestry of the hydrogenosomes of *Nyctotherus ovalis*. *Mol. Biol. Evol.* 17, 202–206. doi: 10.1093/oxfordjournals.molbev.a026234

Vďačný, P., Bourland, W. A., Orsi, W., Epstein, S. S., and Foissner, W. (2011). Phylogeny and classification of the Litostomatea (Protista, Ciliophora), with emphasis on free-living taxa and the 18S rRNA gene. *Mol. Phylogenet. Evol.* 59, 510–522. doi: 10.1016/j.ympev.2011.02.016
